# Second-Generation Anti-Tubercular Squaramides Targeting Complex V of the Respiratory Chain of *Mycobacterium tuberculosis* Displaying Enhanced Metabolic Stability

**DOI:** 10.1021/acs.jmedchem.5c03274

**Published:** 2026-04-23

**Authors:** Nada Mosallam, Paul M. O’Neill, Monika Lisauskaitė, Christopher M. Woodley, Alison Ardrey, Laura N. Jeffreys, Ilinca Memelis, Deepak Almeida, Jin Lee, Paul J. Converse, Daire Cantillon, Eric L. Nuermberger, Dirk Bald, Giancarlo A. Biagini, W. David Hong, Neil G. Berry, Gemma L. Nixon

**Affiliations:** † Department of Chemistry, 4591 University of Liverpool, Liverpool L69 7ZD, U.K.; ‡ Centre for Drugs & Diagnostics, Department of Tropical Disease Biology, 9655 Liverpool School of Tropical Medicine, Liverpool L3 5QA, U.K.; § Centre for Tuberculosis Research, Liverpool School of Tropical Medicine, Liverpool L3 5QA, U.K.; ∥ Center for Tuberculosis Research, Department of Medicine, 1466 Johns Hopkins University School of Medicine, Baltimore, Maryland 21287, United States; ⊥ Department of A-LIFE, AIMMS, 1190 Faculty of Science, Vrije Universiteit Amsterdam De Boelelaan 1108, Amsterdam 1081 HZ, The Netherlands

## Abstract

Squaramides (SQA) were reported as potent antituberculosis drugs through inhibition of the mycobacterial enzyme adenosine triphosphate (ATP) synthase, which is critical for ATP synthesis. However, squaramide compounds showed high metabolic clearance (CL) despite their promising potency and selectivity. Herein, we describe lead optimization efforts to improve the potency and metabolic stability of previous lead **1f**. Multiple squaramide analogues exhibited improved potency. The most potent analogue **20j** expressed enhanced potency of 51 nM and moderate metabolic stability. SQA **6k** displayed optimum balance of potency and metabolic stability both *in vitro* and *in vivo*. Notably, against bedaquiline-resistant *Rv0678* mutants, a modest 2-fold increase in **6k** minimum inhibitory concentration (MIC) was observed—reversed by complementation—versus a 16-fold shift in bedaquiline (BDQ) MIC. In a chronic tuberculosis (TB) mouse model, **6k** coadministered with 1-aminobenzotriazole (ABT) exhibited bactericidal activity. These findings provide key strategies for improving potency and pharmacokinetic properties toward a tractable preclinical candidate.

## Graphical abstract

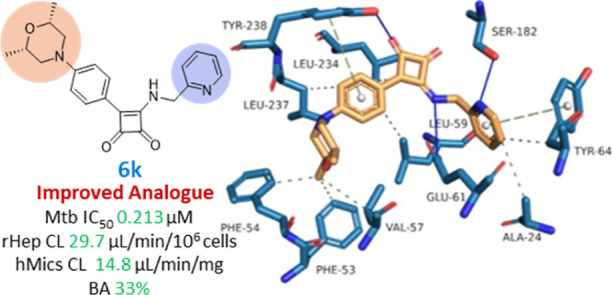

## Introduction

Tuberculosis (TB) is an ancient disease that has caused a tremendous number of deaths in mankind. The disease is caused primarily by *Mycobacterium tuberculosis* (Mtb) and is transmitted through inhalation of infected airborne droplets, when an infected patient coughs or sneezes.^1,2^ TB affects approximately 10 million people each year and the number has been rising since 2021.^3^ Most patients initially have latent TB where the host responds to infection by remodeling the site of infection into a cellular mass, tubercle or granuloma, which has given the disease its name.^4^ The progression of infection from the latent to active phase depends primarily on the host’s immune response; therefore, individuals with compromised immune systems are at greater risk of developing the disease.^5^ One of the major risk factors of TB is infection with human immunodeficiency virus (HIV), which accounts for 12% of all new active TB cases and 25% of all TB-related deaths.^6^ Other risk factors include poor nutrition, cancer, excessive use of alcohol, indoor air pollution, smoking, type-2 diabetes mellitus, and stress.^4^

Despite the high morbidity and mortality, TB is preventable and can be cured with therapeutic treatment. The current TB treatment protocol consists of a six-month combination course of the four first-line drugs: isoniazid **1a** (INH), rifampicin **1b** (RIF), pyrazinamide **1c** (PZA), and ethambutol **1d** (EMB) (Chart 1).^7,8^ The first two months are called the intensive phase where all the four drugs are taken daily, while the last four months are called the continuation phase where only RIF and INH are taken (Chart 1).^8^ The full duration of the treatment is critical for the effective and complete eradication of TB bacilli. More recently, the World Health Organization (WHO) has endorsed a shortened four-month regimen that combines INH, rifapentine **1g**, PZA, and moxifloxacin **1h** for eligible patients with drug-susceptible pulmonary TB (≥12 years, ≥40 kg, no drug resistance), as well as a four-month regimen for children with non-severe TB consisting of two months of INH, RIF, PZA, EMB followed by two months of INH and RIF. Failure to respond to these first-line drugs requires reassessment for drug resistance and treatment with second or even third line drugs.^9^ For this drug-resistant TB, including multidrug-resistant (MDR) and extensively drug-resistant (XDR) a more complex regimen with drug combination is required for 6–9 months.^8^

**Chart 1:**
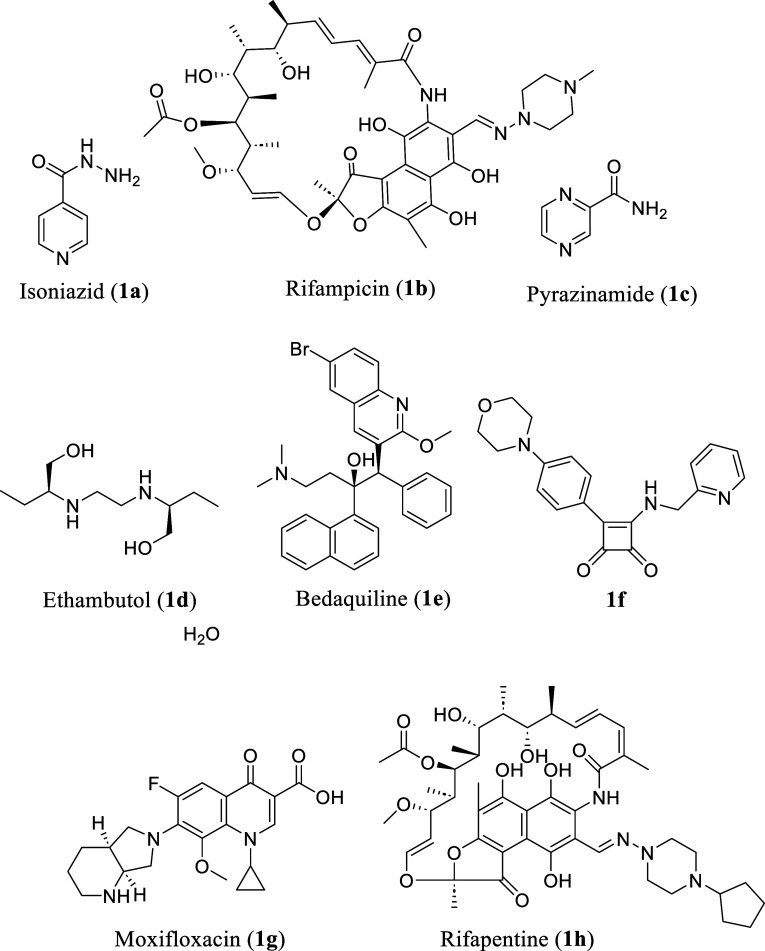
First-Line Anti-TB Drugs and Squaramide Lead Compound

Rising cases of MDR and XDR-TB threaten global efforts to reduce the disease burden.^10,11^ The declining effectiveness of current treatments highlights the need for novel drugs with new targets that bypass existing resistance mechanisms. Inhibiting critical elements of the *M. tuberculosis* respiratory chain has proven effective in eradicating both active and dormant bacteria.

Novel candidates should also possess better safety and tolerability profiles and compatibility with other anti-TB drugs as well as concomitant HIV chemotherapeutics.^8^

Bedaquiline (**1e**) is approved as part of combination therapy for the treatment of MDR and XDR pulmonary tuberculosis in adults and children ≥5 years of age (≥15 kg), targeting the ATP synthase in *M. tuberculosis* (Chart 1).^8,12,13^ Bedaquiline displays high selectivity against mycobacterial ATP synthase over the human homologue.^14^ It has been postulated that ATP synthesis through oxidative phosphorylation is essential regardless of the metabolic state of the mycobacteria (during active or slow-growing “dormant” state).^13^ Moreover, targeting additional components of the electron transport chain (such as cytochrome *bc*1:*aa*3, type I nicotinamide adenine dinucleotide (NADH) dehydrogenase (NDH-1), succinate dehydrogenase, type II NADH dehydrogenase (NDH-2), cytochrome *bd*, and fumarate reductase) have been shown to be synergistic and an attractive approach for combination therapy.^15,16^ Bedaquiline is generally well tolerated, but it carries a risk of QT interval prolongation, which can predispose to arrhythmias, and should be used with electrocardiogram monitoring. Other adverse effects include hepatotoxicity, nausea, arthralgia, and headache, but serious events are uncommon when appropriately monitored.^13,17^

In an attempt to find an alternative chemotype that overcomes the limitations of bedaquiline, Tantry et al.^17^ described a novel class of ATP synthase inhibitors, identified through high through put screening, with a squaramide core moiety. The lead compound of the series (**1f**) (Chart 1) exhibits potent mycobacterial killing through ATP synthase inhibition. Interestingly, initial in silico docking studies performed with the squaramide series suggest that binding to the ATP synthase occurs at a unique site distinct from that of bedaquiline.^17^ Moreover, structural studies of BDQ and the lead SQA **1f** using cryogenic electron microscopy (Cryo-EM) of *Mycobacterium smegmatis* (Msm) ATP synthase revealed that BDQ binds mostly in the c-ring and the a-c interface forming multiple hydrophobic interactions.^18^ However, the SQA **1f** binds to a distinct site at the interface between the a- and c-subunits within the aqueous cytosolic half channel of the ATP synthase enzyme forming several hydrogen bonds.^19^ This unique binding site of squaramide **1f** may explain its activity against certain BDQ-resistant mycobacterial strains, its lack of uncoupling effect on the proton motive force, and lack of restoration of ATP hydrolysis at high concentrations as seen with bedaquiline.^19^ This shows that the directional inhibition of the SQA **1f** favors ATP synthesis over hydrolysis, highlighting the mechanistic differences from bedaquiline.^19^ While **1f** displayed good in vitro activity, it also displayed high mouse metabolic clearance in vitro and due to this instability, it was tested in mice using ABT, a pan cytochrome P450 inhibitor. ABT can boost the concentrations of metabolically unstable molecules in mice, therefore maximizing drug exposure for proof-of-concept studies.^20^ Prior to engagement in lead optimization, we profiled **1f** against human liver microsomes and rat hepatocytes and these studies revealed less than optimal metabolic stability profiles. These studies underlined the cross-species metabolic liability of this class of compound with optimization of metabolic stability, a key aim of this research. Here we describe structure activity studies toward the improvement of the metabolic stability of the squaramide class, without compromising antimycobacterial potency.

## Results and Discussion

### Chemistry

#### Left-Hand Side Modifications

StarDrop analysis revealed predicted metabolic liabilities on both the right-hand side (RHS) and left-hand side (LHS) of the squaramide starting point (**1f**) (for full analysis see the Supporting Information).^21^ Our investigation began with the modification of the LHS of the SQA lead compound with substitutions made on both the aryl and morpholine rings. The RHS was kept constant (2-aminomethylpyridine) to enable matched pair analysis with the starting point.

Preparation of the target squaramides, following the original reported synthetic strategy, resulted in very low yields and a difficult purification process (Scheme 1).^17^

**Scheme 1:**
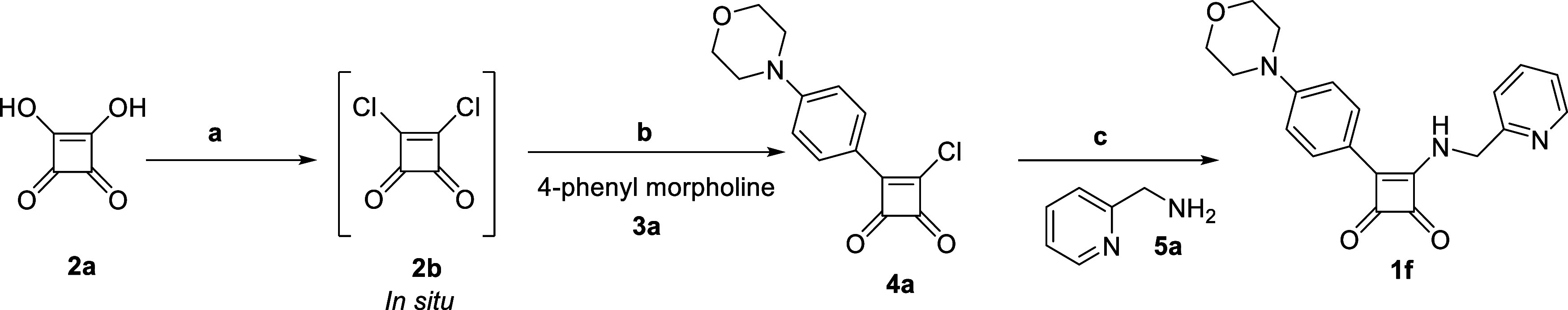
Original Synthetic Route as Developed by Tantry et al.^17^ ^a^

a Reagent and conditions: (a) SOCl_2_, DMF, Toluene, 80 °C, 3 h; (b) AlCl_3_, DCM, 0 °C then rt, 2 h; (c) TEA, 1,4-Dioxane, 0 °C to rt, 0.5 h.

For this reason, an alternative synthesis was investigated. The dichlorosquaric acid starting material was first prepared via squaric acid chlorination using SOCl_2_/DMF and was isolated with good yields (55–77%).^22^ It was then allowed to react with different prepared/commercial phenylmorpholine derivatives (Scheme S1, Table S1)^23^ to achieve the subsequent monochloro squaramides **4a**–**s**. These intermediate SQAs were prepared either using toluene and heat to afford **4a–c** and **4j–s**, where morpholine or other heterocyclic ring alterations were incorporated^24^ or via Friedel-Craft’s alkylation employing AlCl_3_ and CHCl_3_ to afford **4d**–**i** (Scheme 2, Table 1).^25^ The toluene method was found to be more successful for the synthesis of heterocyclic modified analogues particularly with additional solvent dilution, which reduced side products’ formation and facilitated product purification.

**Scheme 2:**
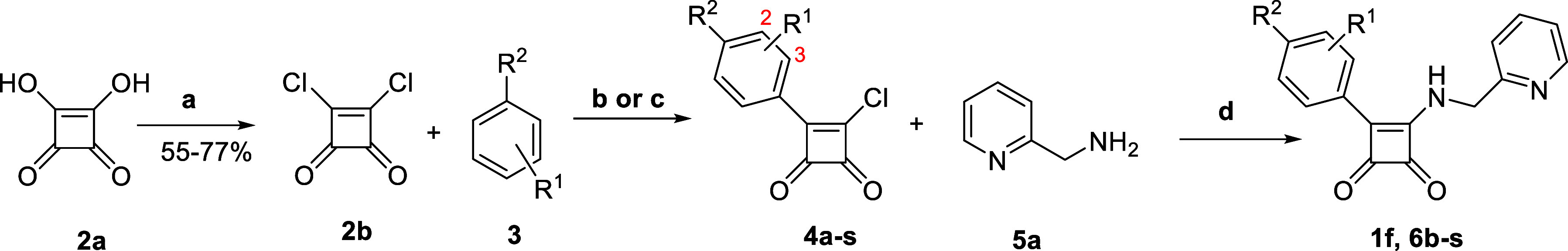
Preparation of the Novel Squaramide Derivatives **1f, 6b–s** Using the Modified Synthetic Route^a^

a Reagent and conditions: (a) SOCl_2_, DMF, Toluene, 80 °C, 3 h; (b) Toluene, 80 °C, 3 h; (c) AlCl_3_, CHCl_3_, 10 °C then rt, 3 h; (d) TEA, 1,4-Dioxane, 0 °C to rt, 1–3 h.

**Table 1:**
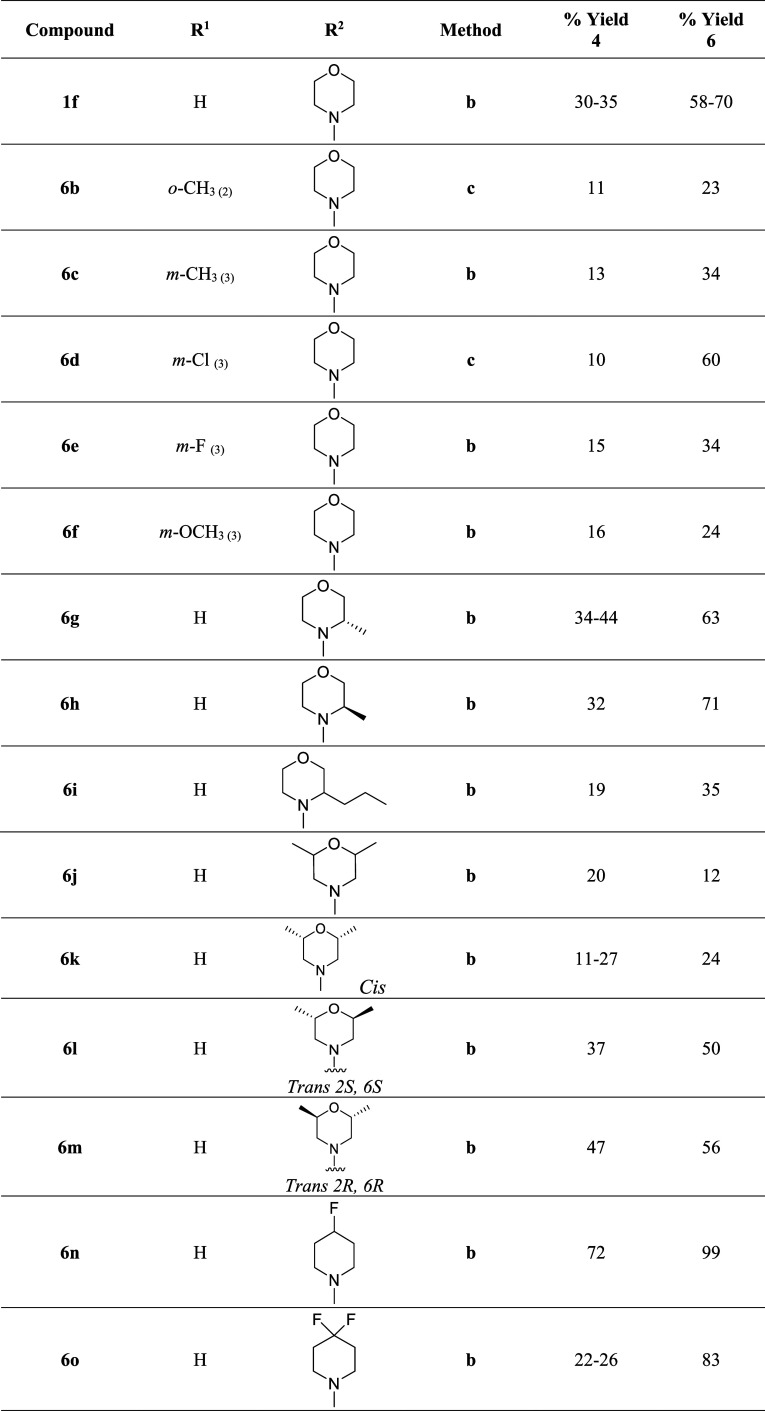

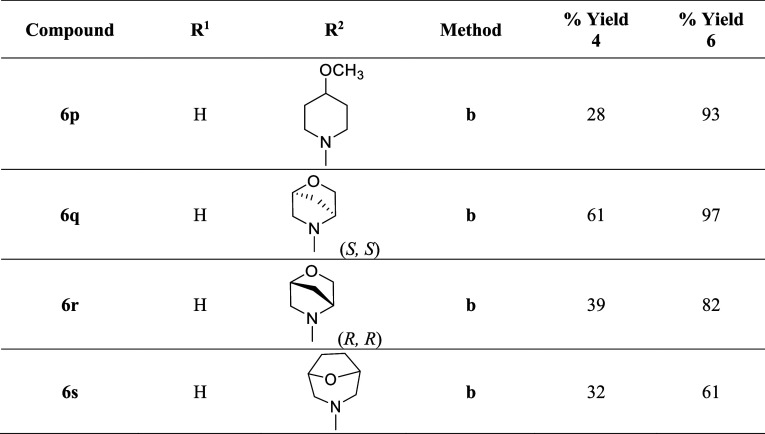
Yields for the Synthesis of Squaramides **1f**, **6b**–**s**

Reaction of the synthesized squaramide intermediates **4a**–**s** with amine **5a** in the presence of TEA was performed to afford the final targets **1f, 6b–s**. This method has been reported in literature and afforded the target compounds **1f, 6b–s** with acceptable to good yields (15–84%), which compensates for the lower yields of the previous intermediate preparation step.^17^ It also employs an easier purification technique via water trituration and subsequent filtration, which reduces any product loss.

As noted, we examined the input of singular substitution on the aromatic ring (**6b**–**f**) to examine how conformational changes (due to a change in torsion angle) could influence both metabolism and antitubercular activity (**6b**–**f**). In addition, the morpholine ring system was substituted at the metabolically prone alpha heteroatom (O/N) position to increase steric protection by a combination of single, disubstitution or by bridging the ring system (See Table 1).^26^ **6n** and **6o** employed fluorine in place of oxygen to see if this isosteric/bioisosteric approach was tolerated. Due to a lack of commercial availability or expensive cost of goods, all phenyl morpholine/piperidines were synthesized by Buchwald–Hartwig amination (See the Supporting Information).

#### RHS Modifications

With the LHS modified, we turned to the RHS of the molecule to trial some simple modifications. To improve reaction yields an alternative route for target synthesis was employed. This route was then used successfully for further target preparation.

Phenyllithium **7** was initially utilized and coupled with diisopropyl squarate and then substituted with 2-picolyl amine to give the SQA **10** (Scheme 3).^27,28^ However, for morpholine-substituted targets a halogen-lithium exchange reaction was required, followed by coupling with diisopropyl squarate and the loss of isopropanol to yield the intermediate **12** (Scheme 3).^29^ One-pot synthesis of these two steps was chosen, as it provided better yields and easier purification. Amination of this isopropoxy intermediate **12** was then performed conveniently using various amines. It should be noted that this route could not be applied to LHS modified analogues due to the low yields of the first step involving lithium halogen exchange and the degradation of the resulting intermediate.

**Scheme 3:**
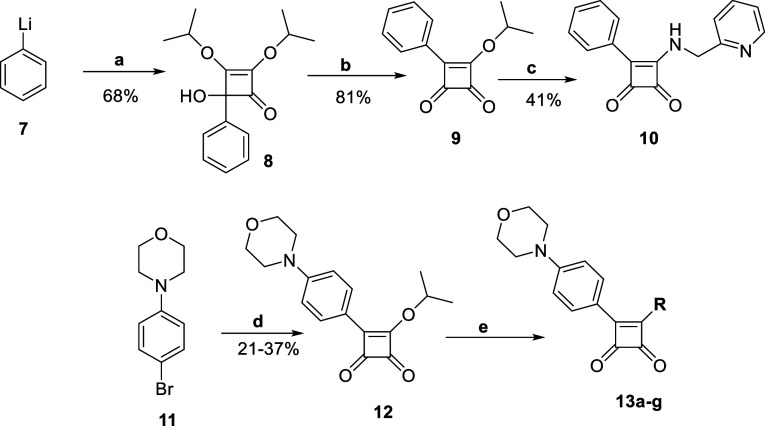
Synthesis of the Squaramide Analogues **10** and **13a**–**g** Using the New Synthetic Route^a^

a Reagent and conditions: (a) 1. Diisopropyl squarate, anhydrous THF, −78 °C, 2 h, 2. H_2_O, −78 °C, 0.25 h; (b) Conc. HCl, 0 °C to rt, 1 h; (c) MeOH, rt, 0.5 h; (d) 1. *t*-BuLi, anhydrous THF, −78 °C, 2.5 h, 2. Diisopropyl squarate, rt, 18 h, 3. H_2_O, −78 °C, 0.25 h; 4. Conc. HCl, 0 °C to rt, 1 h; (e) MeOH, rt, 2–18 h. See Table 2 for R groups.

In addition to evaluating pyridyl analogues (**13a**–**f**), we also included a pyrimidine analogue (**13g**). Amination of the isopropoxy intermediate **12** was then employed using different amines to afford the new RHS targets **13a**–**g** (Scheme 3, Table 2).^27,28^

**Table 2:**
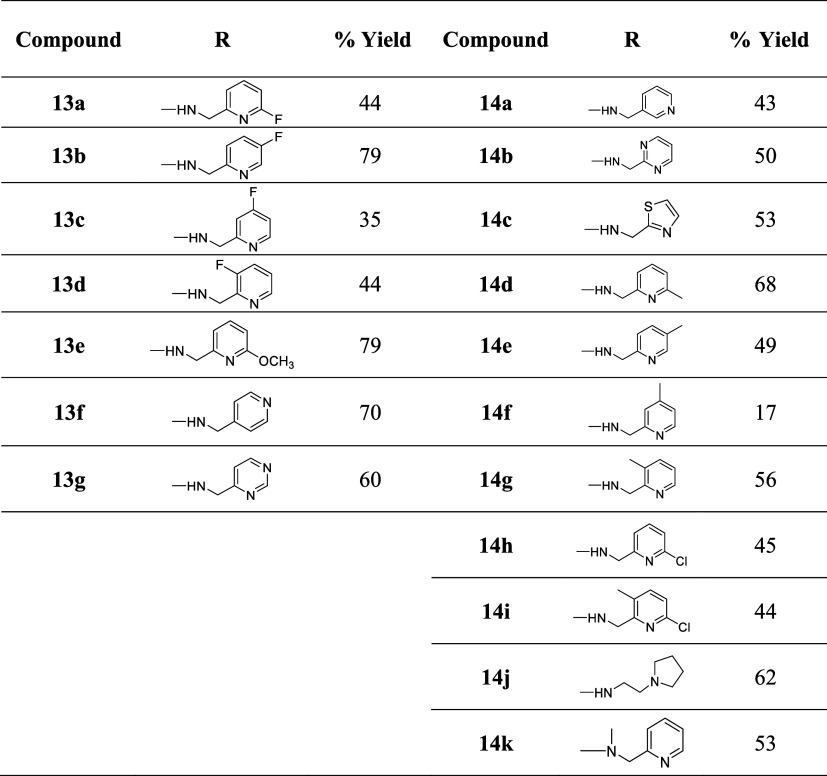
Yields for the Synthesis of Compounds **13a**–**g** and **14a**–**k**

To compare between the original and the new route in terms of yields and purity, several other RHS-modified analogues were also synthesized following the amended original synthetic approach.

Squaramide analogues **14a**–**g** were synthesized following Scheme 1 using 3-chloro-4-(4-morpholinophenyl)­cyclobut-3-ene-1,2-dione **4a**. The SQA intermediate **4a** was then allowed to react with the amines to afford **14a**–**k** in good yields (Scheme S2, Table 2).

While most of the pyridine amines required could be purchased, some of them had limited commercial availability. Therefore, their synthesis from suitable starting materials was performed (Schemes S3, S4 and Table S2).^30,31^

#### Merging RHS/LHS Modifications

To assess the possible synergistic effects of the most potent substituents, the best RHS and LHS substitutions in terms of activity and metabolic stability were combined. Accordingly, novel analogues with both modifications were prepared. However, the newly developed synthetic route in Scheme 3 could not be applied to other LHS derivatives either due to the very poor yields obtained or difficulty in isolating the products.^27,28^ Therefore, the original route was used for the synthesis of analogues with both LHS and RHS alterations (Scheme S5, Table 3).

**Table 3:**
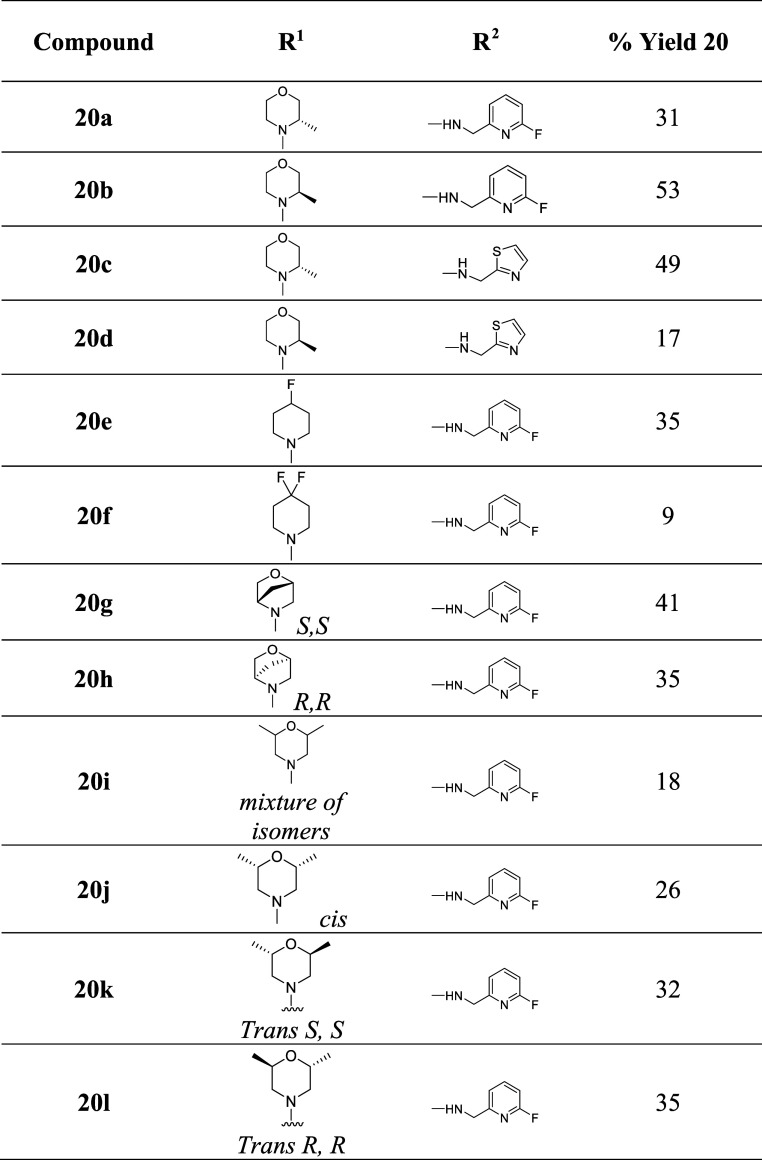
Yields for the Synthesis of Compounds **20a**–**l**

### Biological and Pharmacokinetic Evaluation

#### In Vitro Testing against *M. tuberculosis* H37Rv and DMPK Profiling

All analogues were tested in vitro against *M. tuberculosis* H37Rv, with selected promising compounds and then subjected to drug metabolism and pharmacokinetic testing (DMPK) (Table 4). Compounds with low or absent activity were evaluated only once during the initial screening and were not pursued further.

**Table 4:**
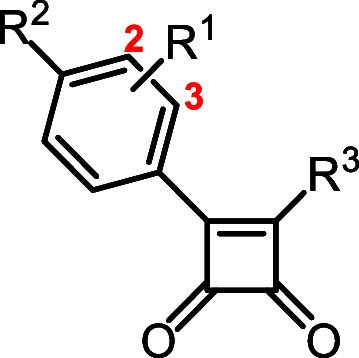

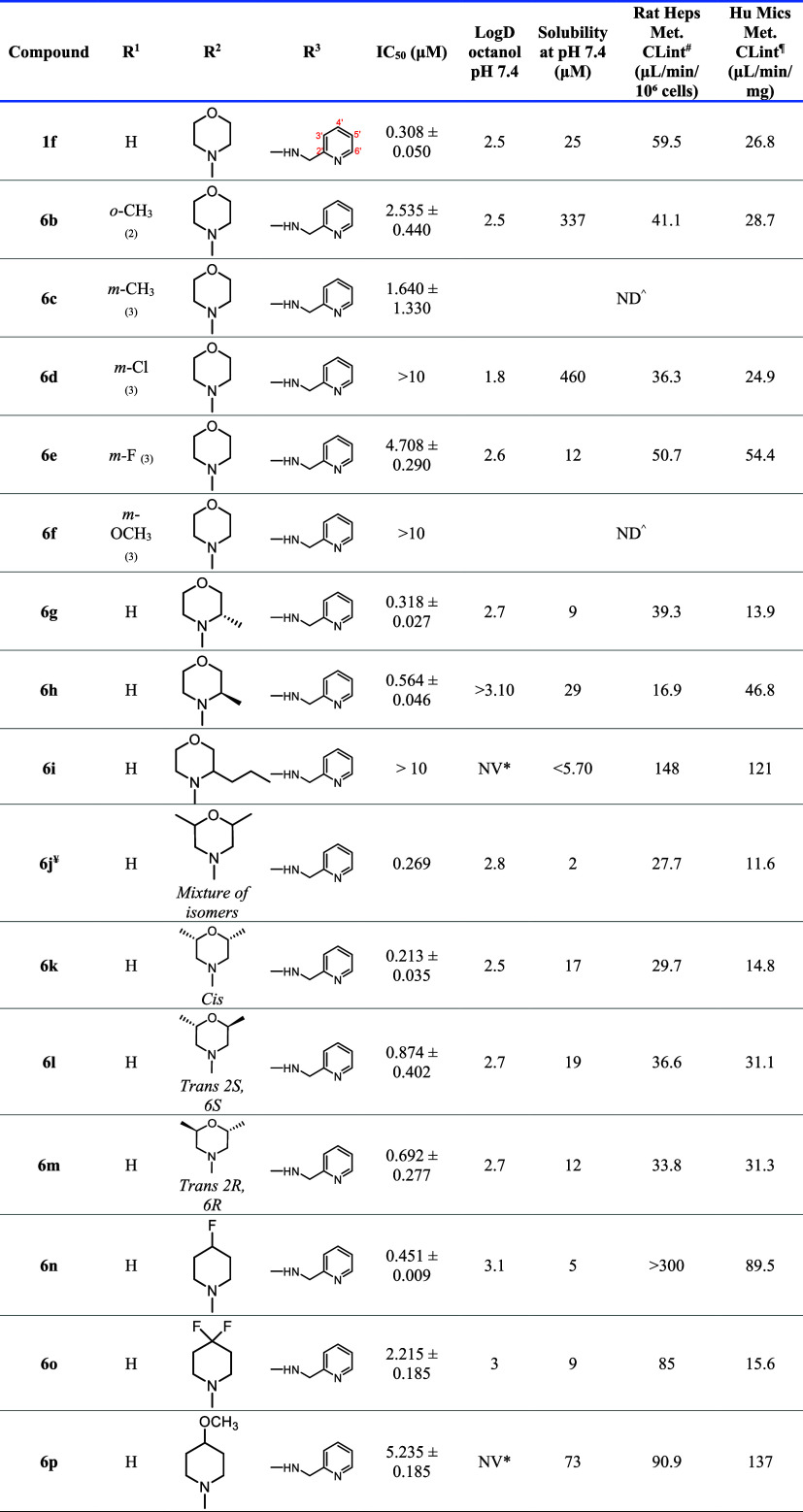

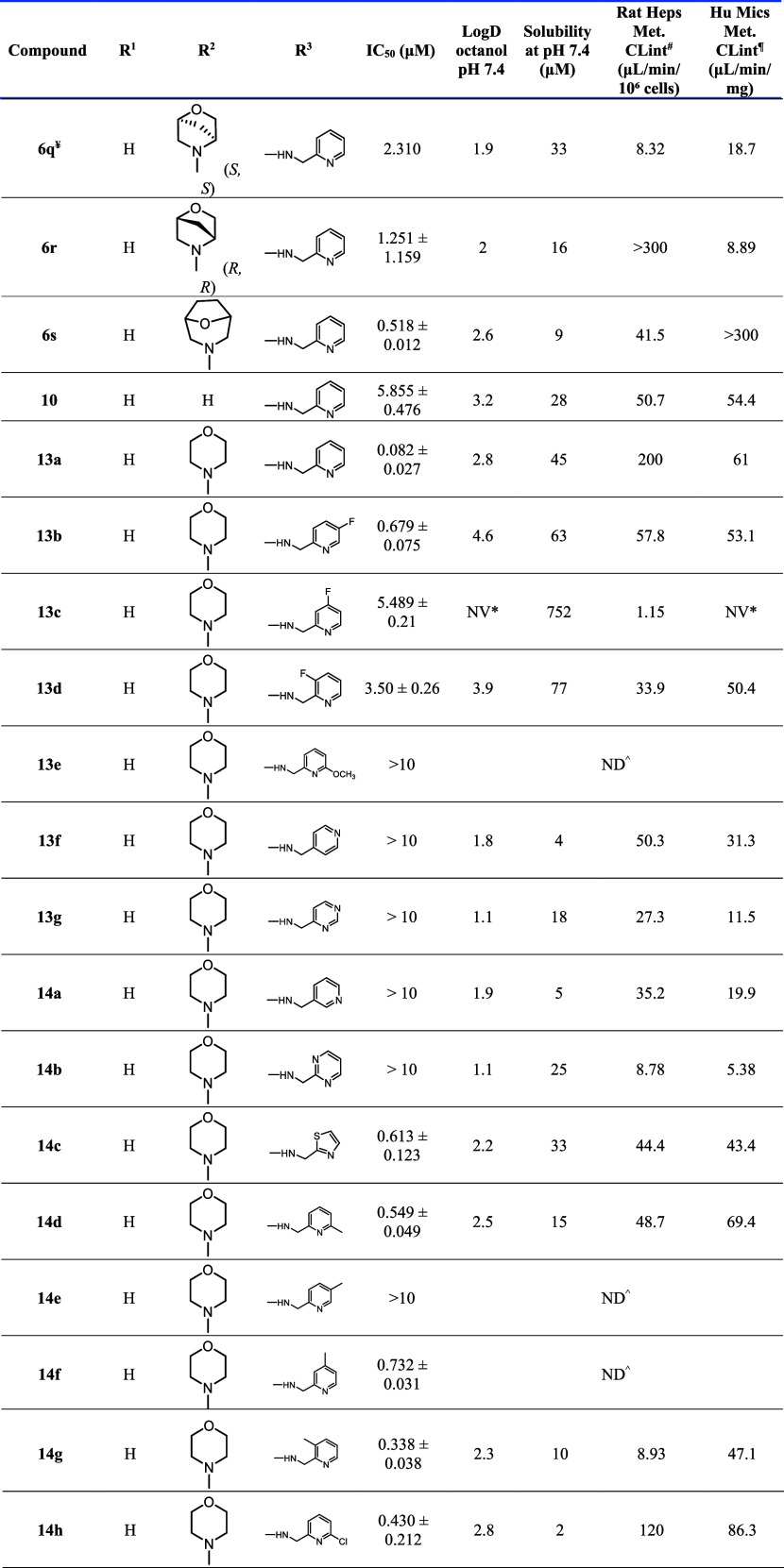

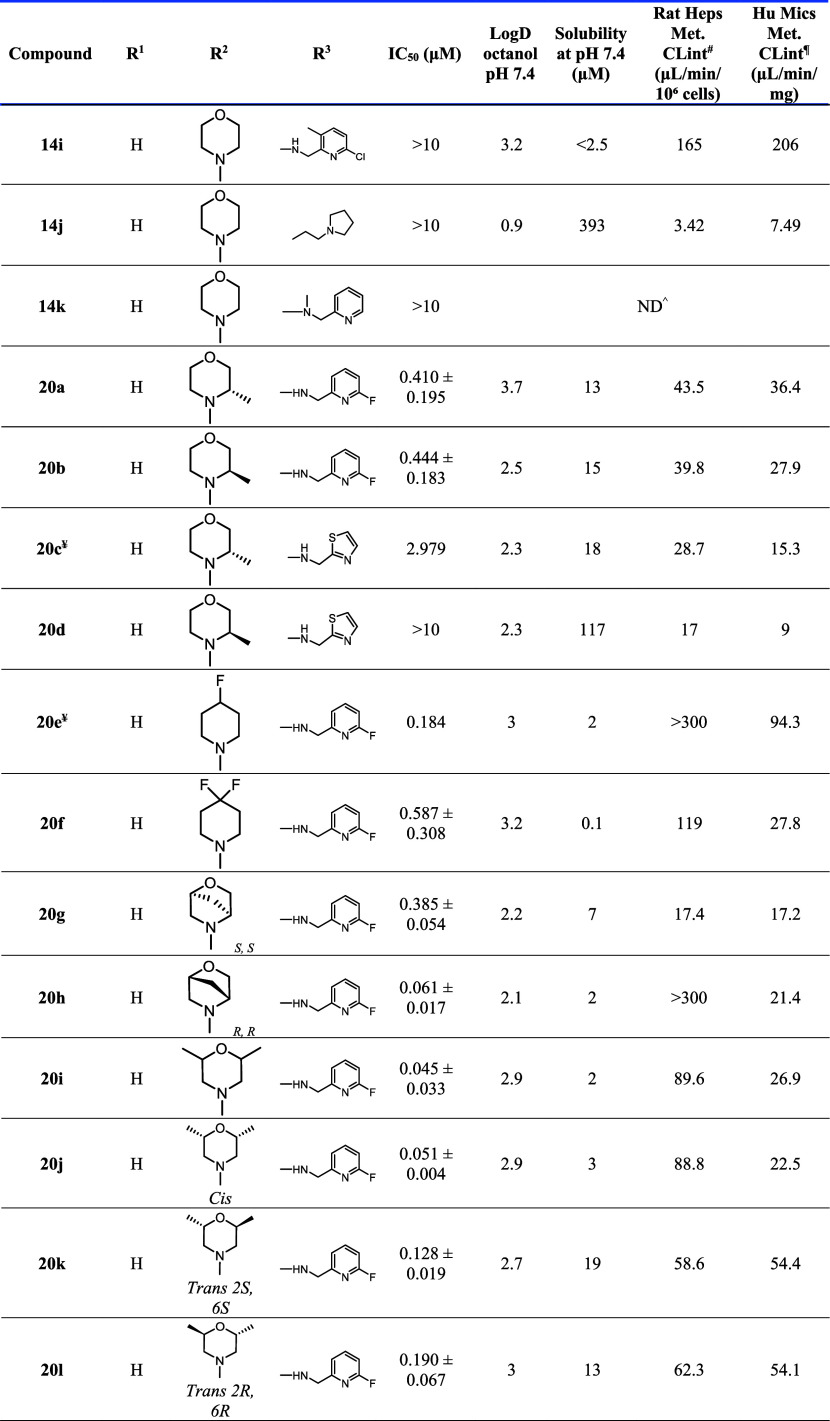
In Vitro Anti-TB Activity and DMPK Testing of the Prepared Squaramide Derivatives^a^

a ND^: not determined, NV*: No volume, Rat Met. CL_int_^#^: rat hepatocytes metabolic clearance, Hu Mics Met. CL_int_^¶^: Human microsomes metabolic clearance. **IC_50_ values were determined from in vitro dose–response assays. Values represent mean ± SEM from two biological replicates, each performed in triplicate. Values denoted ^¥^ are from a single initial screen (performed in triplicate) and were not repeated because of the racemic mixture for **6j**, reduced isomer potency for **6q**, low potency of **20c** and high clearance of **20e**. Intrinsic clearance values of 1–20 μL/min/mg are generally considered indicative of good metabolic stability, values of 20–60 μL/min/mg are classified as moderate, and values >60 μL/min/mg are regarded as high clearance and therefore metabolically unstable, consistent with established drug discovery benchmarks and literature precedent.^36,37^ While these cut-offs were initially defined according to Esaki et al.,^38^ they also align with commonly applied industry frameworks for interpreting in vitro intrinsic clearance data and its translation to in vivo hepatic clearance. In the context of tuberculosis drug development, clinical pharmacokinetic data indicate that first-line agents such as rifampicin and isoniazid display relatively modest systemic clearance (e.g., rifampicin ∼4.5 L/h; isoniazid ∼25–62 L/h depending on acetylator status), suggesting that rapid metabolic turnover is not a feature of many clinically successful TB therapies.^39^ Therefore, compounds exhibiting intrinsic clearance values >60 μL/min/mg would be expected to show reduced in vivo stability compared to lower clearance analogues in this series.

In vitro testing for the hit compound **1f** was first performed to validate potency, where it displayed very good inhibitory activity against whole cell *M. tuberculosis* screening half maximal inhibitory concentration (IC_50_) of 0.308 μM (Table 4). However, DMPK profiling of **1f** revealed a high metabolic clearance in both rat hepatocytes and human microsomes.

#### LHS Modifications

LHS-modified analogues were then prepared **1f, 6b–s,** and **10** (Table 4). Incorporation of the methyl group to position **2** of the phenyl ring significantly reduced activity (**6b**) (IC_50_ = 2.535 μM) while the methyl group on position **3** seems to be more tolerated (**6c**) (IC_50_ = 1.640 μM) (Table 4). The dramatic reduction in activity of **6b** could be attributed to steric clashes between the methyl group and the target protein. This directed our attention to other substitutions in the same position that could be tolerated such as fluoro, chloro, or methoxy groups. Chloro substitution at position **3** (**6d**) of the phenyl ring led to loss of activity. On the other hand, fluoro substitution at the same position (**6e**) was tolerated (IC_50_ = 4.708 μM) compared to the chloro analogue **6d** (IC_50_ > 10 μM) probably due to its smaller atomic size (Table 4). The methoxy-substituted analogue (**6f**) on position **3** showed similar results to the chloro derivative (**6d**) which further supports the steric clash hypothesis and limits the possibility of derivatization at this position (Table 4). Unfortunately, all the phenyl ring substitutions showed high metabolic clearance compared to the lead compound.

This directed our efforts into morpholine ring substitutions, which seem to offer better metabolic stability with maintenance of the activity of the lead compound. Methylated morpholines are known to display good metabolic stability owing to their blockage of biodegradation next to the heteroatom.^32,33^ This effect was observed when examining clearance rates for **6g**–**h**. Monomethylated compounds **6g** and **6h** showed good antitubercular activity with IC_50_ values of 0.318 and 0.564 μM, respectively (Table 4). The *S*–Me morpholine analogue displayed higher activity than its *R*-Me congener. Additionally, **6g** showed better metabolic stability in human microsomes, while **6h** was more stable in rat hepatocytes. Propyl (**6i**) morpholine was also prepared, where **6i** was both inactive and metabolically unstable (Table 4).

2,6-Dimethyl substitution provided the more stable analogue **6j** (contains a mixture of isomers) (Table 4). This analogue also expressed very good potency upon initial screening (0.26 μM) similar to the lead **1f**. Based on this promising result, we then examined all possible stereoisomers at the 2,6-position (**6k**–**m**) (Table 4). The *cis* isomer **6k** displayed optimum TB inhibitory activity (IC_50_ = 0.213 μM) similar to the mixture of isomers **6j** with a comparable DMPK profile. However, both *trans* isomers **6l** and **6m** had slightly higher clearance rates compared to their *cis* congener **6k**, but still lower than the lead compound **1f** in rat hepatocytes. The *trans* 2*S*, 6*S* isomer **6l** displayed marginally lower potency to *trans* 2*R*, 6*R* stereoisomer **6m**, whereas both *trans* isomers **6l**–**m** displayed lower activity compared to their *cis* congener **6k**. Both *trans* isomers also exhibited higher metabolic clearance particularly in human microsomes. This difference in potency and metabolic stability between the *cis* and *trans* isomers could be due to stereochemistry-dependent variation in target binding and susceptibility to metabolic enzymes.^34,35^

Our next approach was to consider morpholine ring replacements. Various 6-, 7-, and 8-membered heterocyclic rings were examined (Table 4). All the analogues had a hydrogen bond acceptor. The 4-fluoro piperidine analogue **6n** exhibited very good potency of 0.451 μM while the difluoropiperidine **6o** displayed a lower potency of 2.215 μM, along with high metabolic clearance in rat hepatocytes (Table 4). Similarly, the methoxy piperidine derivative **6p** displayed metabolic instability and reduced inhibitory activity (IC_50_ = 5.235 μM). The 7-membered heterocylces *S*- (**6q**) and *R*- (**6r**) oxa-azabicyclo[2.2.1]­heptane phenyl squaramides also were investigated. **6q** displayed reduced activity in the initial screening (IC_50_ = 2.310 μM), whereas **6r** exhibited improved activity of 1.251 μM (Table 4). However, the *S* derivative **6q** was more metabolically stable than its *R* congener despite being less active against TB.

An 8-membered heterocycle was also prepared in the hope of enhancing metabolic stability. The SQA with 8-oxa-3-azabicyclo[3.2.1]­octane (**6s**) elicited good TB inhibitory activity (IC_50_ = 0.518 μM) but unfortunately suffered from high metabolic clearance especially in human microsomes. This could be attributed to the exposed aliphatic carbons which might be more vulnerable to metabolism.

Removal of the morpholine ring in compound **10** reduced the activity to 5.855 μM and resulted in higher clearance values; therefore, no additional analogues were pursued without this key group (Table 4).

#### RHS Modifications

In terms of RHS modifications **13a**–**g** and **14a**–**k** (Table 4), fluorination at different positions of the pyridine ring were performed where the squaramide analogue **13a** exhibited the most potent activity of 0.082 μM, which is significantly more active than the lead SQA **1f**. This could be due to the electronic and steric effects of fluorine atom, where it can form an electrostatic interaction with the protein and may result in better target engagement.^40^ Compound **13b** exhibited potent activity (IC_50_ = 0.679 μM) while compounds **13c** and **13d** showed moderate TB killing effects of 5.489 and 3.50 μM, respectively. With regards to the metabolic stability, compounds **13b** and **13d** had slightly high clearance rates. However, we failed to create an analytical method in human microsomes for **13c**. Unfortunately, the potent derivative **13a** showed a very high clearance rate in both rat hepatocytes and human microsomes. Methoxy pyridine substituents were also investigated, but the prepared analogue **13e** showed no inhibitory activity against TB.

Pyridine ring methylation was also evaluated to identify the best tolerated positions. Indeed, methyl substitutions at positions **3′**, **4′** and **6′** of the pyridine ring were very well tolerated and showed good results **14d**–**g**, with position **3′** being the best one (**14g**, IC_50_ = 0.338 μM) (Table 4). However, methyl substitution at position **5′** resulted in loss of activity (**14e**, IC_50_ > 10 μM).

Analogues incorporating pyrimidine rings or substituted at different positions on the pyridine ring (**13f**–**g** and **14a**–**b**) showed no activity against TB (Table 4). This suggests that additional or modified substitutions of this nature may disrupt the proposed interaction of the lone pair on nitrogen with Serine 182 in the docked protein–ligand structure.

Pyridine ring replacement with thiazole **14c** resulted in good potency (IC_50_ = 0.613 μM) and improved metabolic stability. The 6-chloropyridine analogue **14h** exhibited lower potency (IC_50_ = 0.430 μM) compared to the fluoro analogue **13a** (IC_50_ = 0.082 μM) and a higher clearance rate in human microsomes. To combine the best RHS substituents in terms of activity, a squaramide with 3-methyl, 6-chloropyridine **14i** was prepared. Unfortunately, the compound was both inactive and metabolically unstable. This could be due to steric hindrance around the ring nitrogen, potentially impairing key binding interactions or altering the conformational preference of the scaffold.

Replacing the pyridine ring with pyrrolidine moiety **14j** completely abolished the activity, which suggests that ring aromaticity and planarity are essential for binding with the target protein.

Furthermore, we aimed to evaluate the NH-methyl linker substituent tolerability. Since Tantry et al.^17^ reported that *C*-methylation of the aminomethyl linker adjacent to the pyridine ring was not tolerated, *N*-alkylation of the squaramide amine was not investigated. Therefore, an *N*-methyl aminopyridine analogue **14k** was prepared (Table 4). If tolerated we hypothesized that this could act as a suitable handle for macrocyclization to explore improved binding affinity though conformational-locking.^41^ Unfortunately, *N*-methylation resulted in loss of activity in **14k**, suggesting the importance of the N–H for activity.

#### Merging RHS/LHS Modifications

The final part of our analysis combined metabolically stable LHS substituents with the most potent RHS derivatives **20a**–**l** (Table 4). The best LHS derivatives in terms of potency and metabolic stability were **6g**–**h**, **6j**–**o** and **6q**–**r**. The most potent RHS analogue is **13a** with 3.8-fold improvement in TB inhibition and the most stable one was **14c**. Therefore, analogues with the fluoropyridine or thiazole RHS and methyl morpholine or fluoropiperidine LHS were targeted for synthesis.

Adding the metabolically more stable (*S*)-3-methyl-4-phenylmorpholine to the most potent fluoropyridine substituent significantly boosted the stability of **20a,** which exhibited moderate clearance rates compared to its congener without the methyl morpholine substitution **13a** while maintaining good activity of 0.410 μM (Table 4).

The *R*-methyl morpholine derivative was also coupled with the 6-fluoro pyridine RHS to form the target analogue **20b**. The compound displayed good activity of 0.444 μM, similar to its *S*-methyl stereoisomer with comparable clearance rates. On the other hand, coupling the methyl morpholine substitution with the RHS thiazole ring led to dramatic reduction in activity for **20c** (IC_50_ = 2.979 μM) in initial screening, whereas **20d** lost its activity although the only difference is in their stereochemistry. This suggests steric or conformational disruption of key binding interactions. The complete loss of activity in the corresponding stereoisomer **20d** indicates a strong stereochemical requirement within the binding pocket.

Since the 4-fluoropiperidine derivative (**6n**) exhibited good activity, it was also combined with the most active fluoropyridine to evaluate the possible synergistic effect on potency. Indeed, combining both substituents resulted in the superior activity of **20e** (IC_50_ = 0.184 μM) upon initial screening. Unfortunately, the compound was metabolically unstable. Similarly, installing 6-fluoropyridine RHS to the difluoropiperidine LHS resulted in a remarkable enhancement in TB inhibitory activity of **20f** by almost 4-fold (IC_50_ = 0.587 μM). Despite good potency, this analogue also exhibited a high clearance rate in rat hepatocytes. This clearly highlights the prominent effects of incorporating a fluorine atom to the SQA compounds.^42,43^

Adding the 6-fluoropyridine RHS to the oxazabicycloheptanes **6q** and **6r** resulted in compounds **20g** and **20h,** respectively, which were significantly more potent than their nonfluorinated counterparts. (1*S*, 4*S*-5-methyl-2-oxa-5-azabicyclo­[2.2.1]­heptane) **20g** maintained a similar inhibitory activity (IC_50_ = 0.385 μM) to the lead compound **1f** while displaying improved metabolic resistance, as seen from its lower clearance rates in human microsomes and rat hepatocytes. On the other hand, the 1*R*, 4*R* enantiomer **20h** exhibited a 20-fold increase in the TB inhibitory activity (IC_50_ = 0.061 μM) compared to the nonfluorinated congener **6r** and is considered one of our most potent SQAs in our series (Table 4). Interestingly, this underlines the exceptional potency improvement of 6-fluoro pyridine. The difference in potency of **20g**–**h** indicates a strong stereochemical preference within the binding pocket. Despite the low clearance rate of **20h** in human microsomes, it was rapidly cleared in vitro from rat hepatocytes.

From the 2,6-dimethyl morpholine analogues **20i**–**l**, both the mixture of isomers **20i** and the *cis* analogue **20j** displayed remarkable activity with IC_50_ values of 45 and 51 nM, respectively (Table 4). Not only did the SQAs **20i**–**j** exhibit superior potency but they also provided compounds that are relatively stable to metabolism in human microsomes. Unfortunately, **20i**–**j** displayed a relatively high elimination rate in rat hepatocytes. Similarly, the *trans* isomers **20k**–**l** provided enhanced potency against whole cell TB; however, they were rapidly cleared from both rat hepatocytes and human microsomes.

In summary, we achieved modest synergistic effects produced from combining the more favorable LHS modifications with the RHS 6-fluoropyridine. Morpholine ring alterations and alkylation significantly increased the compounds’ resistance to biodegradation, while the 6-fluoropyridine remarkably enhanced the analogues’ TB inhibitory activity to the nanomolar range.

The optimum SQA analogue in terms of potency and anti-TB activity was selected to be **6k**, where it maintained the original lead **1f**′s potent TB inhibitory activity while improving the metabolic stability. For this reason, **1f** and **6k** were selected for evaluation in the in vivo TB mouse models. It was therefore essential to test the clearance rates of both **1f** and **6k** in mouse liver microsomes to confirm the metabolic resistance of **6k** ahead of the in vivo investigation. **6k** displayed nearly 2-fold improvement in metabolic stability compared to **1f**, as evident from the moderate clearance of **6k** compared to the high clearance rate of **1f** (Table 5). This confirmed the suitability of **6k** for in vivo mouse models. Additionally, permeability studies were evaluated for **6k** where it exhibited high permeability and a poor efflux rate (Table S5).

**Table 5:** In Vitro Intrinsic Clearance in Mouse Liver Microsomes of the Squaramides **1f** and **6k**

compound	*T*_1/2_(min)	CL_int_ (mL/min/kg)
**1f**	23.28	234.46
**6k**	40.56	134.57

#### Structure–Activity Relationship

The structure–activity relationship (SAR) of the prepared derivatives and their biological activity is summarized in Figure 1. In terms of LHS modifications, substitution of the heterocyclic ring was more tolerated and resulted in better potency against *Mycobacterium tuberculosis*. Dimethylation of the morpholine ring at the 2- and 6-positions provided the best analogue of the series in terms of activity and metabolic stability. Methylation *ortho* to the morpholine nitrogen or the bioisosteric replacement of morpholine with other 6-, 7- or 8-membered heterocycles was well tolerated, with morpholine and 4-fluoropiperidine being the most potent. *Ortho*-methylation of the phenyl ring relative to the heterocycle reduced activity, whereas *meta*-methyl and *meta*-fluoro substitution were better tolerated (CH_3_ > F). For the RHS alterations, we confirmed the necessity of the pyridine nitrogen and its position for antitubercular activity as reported by Tantry et al.,^17^ where changing the nitrogen position led to loss of activity. We also found that incorporation of extra nitrogen atoms into the ring completely abolished activity. The NH was also essential for activity, where *N*-alkylation abolished activity. Substitution on the pyridine ring was both position- and substituent-dependent. Chloro or fluoro groups *ortho* to pyridine nitrogen gave the best activity compared to methyl group (*F* > Cl > Me), whereas the methoxy group abolished activity. Halogenation and methylation at other positions gave weak-to-moderate activity. Surprisingly, introduction of a methyl group *meta* to the pyridine nitrogen led to loss of activity, likely due to steric or conformational interference with optimal binding.

**Figure 1:**
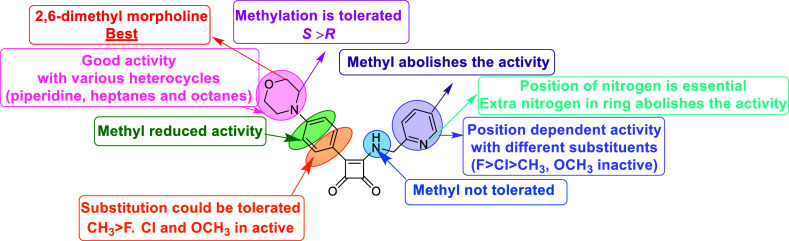
Structure–activity relationship of the prepared squaramides.

#### In Vitro Testing against the Bedaquiline-Resistant *Rv0678* Mutant Strain

Additionally, we evaluated the in vitro activity of **6k** against the wild-type*M. tuberculosis* H37Rv and an isogenic BDQ-resistant mutant with an *Rv0678* mutation using the resazurin microtiter assay (REMA) broth microdilution method (Table 6). Clinical BDQ resistance has been associated with mutations in the transcriptional regulator *Rv0678*, leading to upregulation of the MmpS5–MmpL5 efflux pump.^44^ However, resistance to the squaramides was linked to mutations in the ATP synthase a- and c-subunits, with no evidence for involvement of efflux-mediated mechanism.^17^ Indeed, the *Rv0678* mutation was associated with a 16-fold increase in BDQ minimum inhibitory concentration (MIC) that was reversed by complementation. In contrast, the *Rv0678* mutation was associated with only a 2-fold increase in **6k** MIC that was reversed by complementation. This shows the potential activity of **6k** against BDQ-resistant TB strains and its lower susceptibility to efflux-mediated resistance, providing a basis for further optimization of this SQA series.

**Table 6:** In Vitro MIC of **6k** and BDQ Using the REMA Method

drug	MIC (μg/mL)	REMA method: day 7 MIC (μg/mL)		
		H3Rv	Rv0678	complemented
**6k**	128–0.0078	1	2	1
**BDQ**	64–0.0078	0.125	2	0.06

### Computational Studies

#### Homology Modeling, Molecular Dynamics Simulations, and Molecular Docking

A recently published cryogenic electron microscopy (cryo-EM) structure of *M. smegmatis* ATP synthase in complex with **1f** provides insights into the binding mode of squaramides.^19^ To gain further insight into the mode of binding of our squaramides in Mtb ATP synthase we opted to produce a homology model based on this cryo-EM structure. We aimed to use this structure to gain further insights into squaramide binding to ATP synthase by MD studies and use the refined binding mode from the MD simulations in molecular docking studies to better understand the SAR of our compounds.

We built a homology model using the protein sequences for a-, b-, b-delta- and c-subunits of Mtb ATP synthase using the SWISS-model Web server.^45^ We used the published structure of squaramide bound Msm ATP-synthase as a user-template structure.^19^ The global mean quality estimate was found to be relatively low (0.31); however this is based solely on the target sequence and template quality and is negatively affected by the poor coverage of b- and b-delta-subunits in the chosen template.^45^ The QMEANDisCo metric assesses the quality of the model by comparison with the template structure, giving an indication of the produced model quality considering global and per-residue model quality.^46^ For the produced model, this was found to be acceptable at 0.73 ± 0.05. Other cryo-EM structures of Msm ATP synthase are available with greater coverage of the target sequence;^47^ however we determined that using a squaramide-bound structure as a template would produce a higher quality starting pose for refinement by MD methods.

Inspection of the cryo-EM structure showed that carboxylate groups of residues Asp32 and Glu65, both in the c-subunit (Figure S1A), are found to be in proximity consistent with a hydrogen bond existing between the residues—supporting this is the presence of an area of electron density on the cryo-EM mapping between these residues (Figure S1B). Glu65 is known to be involved in the transfer of protons between the c- and a-subunits,^48^ so we considered the protonation states of each of these residues. We therefore docked **1f** into our homology model with three protonation states; doubly deprotonated (ddp), Asp32 protonated (Y32–H), and Glu61 protonated (E61-H) protonation states. The three generated complexes were used in 150 ns MD simulations in singleton runs to monitor the distance between Asp32 and Glu61. We observed that only for the E61-H protonation state the distance between Asp32 and Glu61 remained stable from the starting conformation (Figure S2). We therefore consider this protonation state to be a reasonable approximation of the native protonation state of this dyad under the cryo-EM conditions.

Additional two MD simulations of the E61-H protonation state were carried out and used to calculate Molecular Mechanics/Poisson–Boltzmann Surface Area (MM/PBSA) binding free energy for the final 20 ns of the simulation and to determine a lowest energy binding mode.^49,50^ A summary of MM/PBSA binding free energies and contributions of polar, nonpolar, and molecular mechanics components is provided in Table S3. Run 2 was found to possess the lowest calculated binding free energy (−147.4 ± 12.6 kJ/mol), and run 3 was found to be of statistically comparable energy (−146.913 ± 11.875 kJ/mol, independent *t*-test *p*-value = 0.84), while run 1 was found to converge to a significantly less energetically favorable conformation (−103.958 ± 12.997 kJ/mol, independent *t*-test *p*-value <0.001). Root Mean Square Deviation calculations on each run confirmed that each simulation converged to a stable conformation in the last 20 ns of simulations (Figure S3), and in each simulation the ligand remained stable within the interface between the a- and c-subunits. Each trajectory was analyzed by agglomerative clustering and interaction analysis to determine stable interactions between protein and ligand (Table S4). While interactions observed in run 2 are shown in Figure 2, interactions observed in run 3 should be considered as possible binding interactions due to the similar predicted end-state binding energy compared to run 2, and less weight should be placed on interactions observed only in run 1.

**Figure 2:**
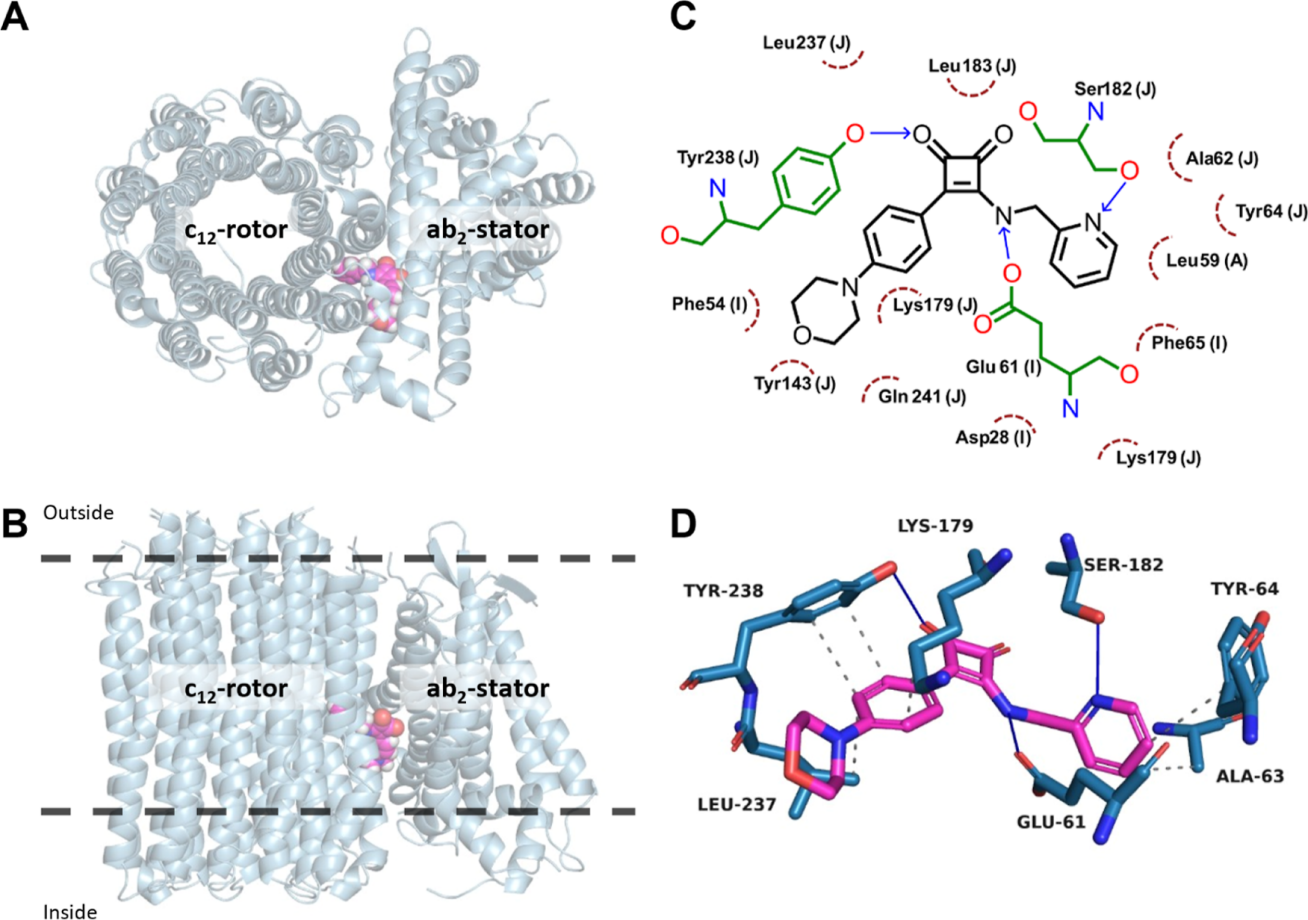
(A/B) PyMOL representation of the end state of the MD simulation of the complex between Mtb ATP-synthase (blue cartoon) and squaramide **1f** (magenta spheres). The protein is viewed from the inside to the outside (A) and along the membrane (B). The position of the protein in the lipid bilayer is represented by dashed black lines. Initial atomic positions were obtained from docking **1f** into a homology model based on protein data bank: 8G07. (C) 2D representations of noncovalent interactions between **1f** with binding site residues. Binding site residues are shown in green, hydrophobic interacting residues are depicted by red dotted arcs, hydrogen bond donors are depicted as blue arrows that point from donor to acceptor. The chain of the interacting amino acid residues is given in parentheses. (D) Interaction analyses of end-state binding modes of **1f** (magenta sticks) in Mtb ATP synthase. Binding site residues are shown as blue sticks, hydrogen bonds are shown as blue lines, hydrophobic interactions are shown as dotted gray lines. Interactions were detected using the Protein Ligand Interaction Profiler tool.^51^

Consistent with the original cryo-EM structure PDB: 8G07, we observe that hydrogen bonding interactions between the squaramide carbonyl with Tyr238 of the a-subunit and the squaramide NH with Glu61 of the c-ring are stable throughout the trajectories in run 2 (Table S4), forming a stable bridge between the two subunits. While a hydrogen bond with Arg186 (a-subunit) is observed in the cryo-EM structure, this is only observed in the first half of the simulation in run 2—this is likely due to the flexibility of the Arg side-chain, and the energetic preference of formation of a salt–bridge interaction with Glu61 of an adjacent c-subunit. These interactions solidify the importance of the squaramide core for the activity of these compounds, including the importance of the free hydrogen bond donor; in compound **14k** with an alkylated squaramide amine, activity is abolished. The morpholine ring of **1f** is observed to bind within a solvent-exposed pocket defined by Phe54 of the c-ring, and Tyr143, Lys 179, and Gln241 of the a-subunit forming primarily hydrophobic interactions. In run 2 we also see Gln241 forming a transient H-bonding interaction with the morpholine oxygen, which may contribute to the preference of morpholine-like moieties in this position. The linking aromatic ring between the squaramide and the morpholine binds deeper within the binding pocket and is observed to form hydrophobic interactions with the residues defining the pocket. The restricted nature of the binding pocket toward the squaramide core explains the observed lack of tolerance for bulky substituents at the positions of the ring closest to the core, and tolerance at the more solvent exposed side.

Lastly, we see that the pyridine binds within a pocket defined by Tyr64 and Ala62 of the a-subunit, and Leu59 and Phe65 of the c-ring form primarily hydrophobic interactions—this pocket is located between two adjacent c-subunits with little room for ligand growth, consistent with the lack of tolerance for bulky substituents. Note that while interactions with Tyr64 and Phe65 were identified as hydrophobic, they may also form π–π interactions, but these were not identified by our sampling methods. We see from our SAR that movement of the pyridine nitrogen from the meta-position reduces activity—a rationale for this is provided by the hydrogen bonding interaction between the ligand and the side chain of Ser182 (a-subunit) by the pyridine nitrogen. This interaction is observed in runs 2 and 3 in 23.3% and 57.9% of frames, respectively. Furthermore, we saw that 2-fluoro pyridyl analogues **13a** and **20j** had enhanced potency compared to the unsubstituted pyridine—this may be explained by enhancement of hydrogen bond strength between the pyridine nitrogen and Ser182 through conjugation of fluorine nonbonding electrons. This conjugation would not be observed in the 3- and 5-fluoro pyridyl analogue **13b/d,** consistent with their reduced activity compared to **13a**. The same cannot apply to the 4-fluoropyridyl analogue **13c**; however the increased solubility of this compound compared to **13a** suggests 4-fluoro pyridyl substitution has significant effects on the physicochemical properties of this analogue and may not be tolerated in the binding pocket.

These studies suggest that compared to Msm, the binding mode of **1f** in Mtb ATP synthase is likely to be unchanged and is consistent with the observed SAR of our compounds. The observed network of stable interactions bridging the a- and c-subunits is supportive of the prevention of c-ring rotation by SQA compounds, in alignment with the findings of Courbon et al.^19^ The end-state structure from run 2 was therefore taken as a candidate for molecular docking to further investigate the SAR of our compounds.

To compare our MD-derived binding mode with our observed SAR we docked the two most potent molecules in this work, compounds **20h** and **20j**, into the end state of our MD trajectory (Figure 3). Both **20h** and **20j** contain the 2-fluoropyridine moiety shown to enhance potency in our SAR exploration. The results of the docking are consistent with this in that the hydrogen bond between the pyridyl ring and Ser182 remains present in both structures. Interaction analysis by Protein–Ligand Interaction Profiler (PLIP)^51^ identified a further halogen bonding interaction with the Glu61 backbone carbonyl, also consistent with enhanced potency. Our SAR identified modification of the LHS morpholine was tolerated and, in **20h** and **20j**, observed to enhance potency compared to the parent unsubstituted morpholine. Our modeling supports this—docked poses of **20h** and **20j** identify a hydrophobic binding pocket defined by Phe53, Phe54 and Val57 not interacted with in our MD simulations. The bridged morpholine in **20h** mediates hydrophobic interaction with Phe54, while the 2,6-dimethylmorpholine forms a network of hydrophobic interactions with Phe53, Phe54, and Val57. The observed preference for *R, R*-bridged morpholine and *cis*-dimethyl morpholine in **20h** and **20j**, respectively, may be due to a conformational preference amenable to binding in this hydrophobic pocket. A further π-stacking interaction was detected between **20j** and Tyr238, supportive of its stronger binding—this LHS binding mode is identical to the pyridine analogue **6k** used in in vivo experiments (Figure 3). It should be noted that no difference in binding mode was observed between the *cis* isomer **6k** and both *trans* isomers **6l**–**m**.

**Figure 3:**
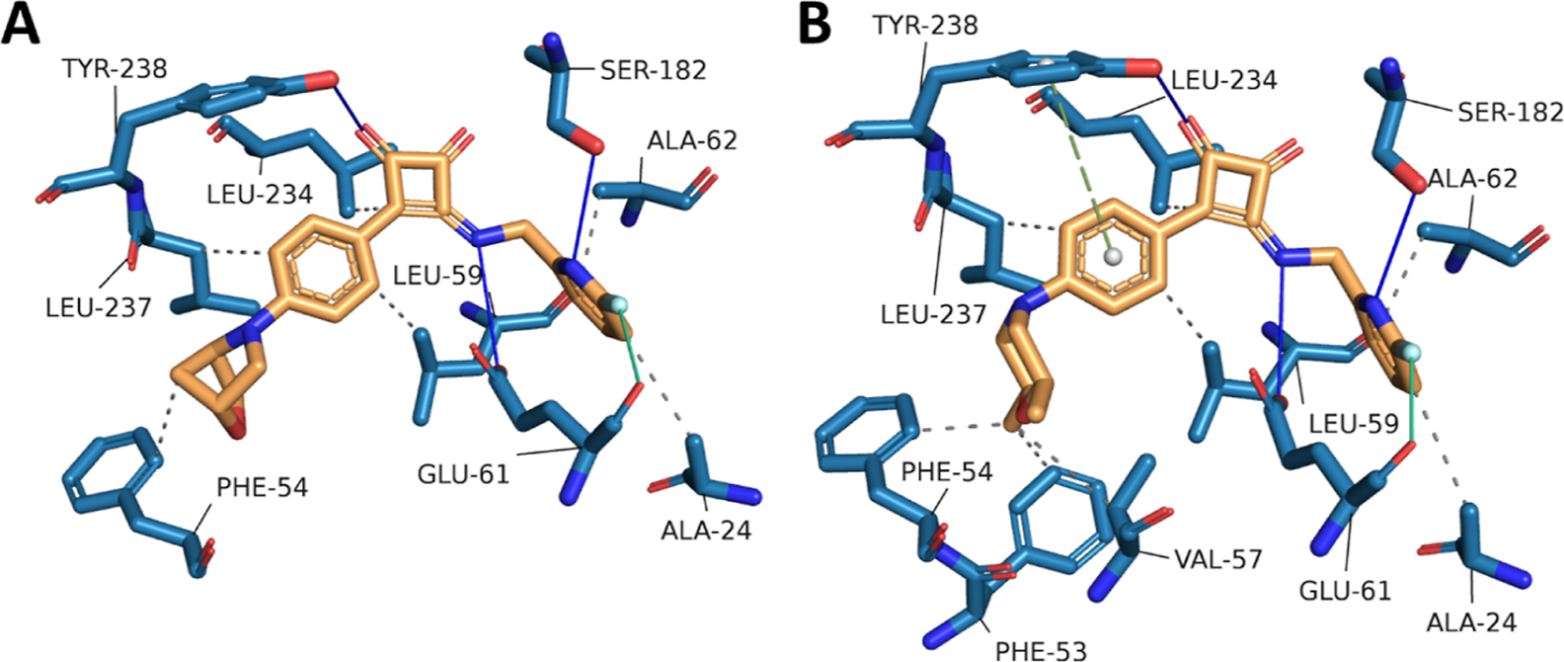
Docked poses of potent squaramide compounds **20h** (A) and **20j** (B), based on PDB: 8G07. Binding site residues are shown as blue sticks, hydrogen bonds are shown as blue lines, hydrophobic interactions are shown as dotted gray lines, halogen bonding interactions are shown as green lines, and π-stacking interactions are shown as olive dashed lines. Interactions were detected using the Protein Ligand Interaction Profiler tool.^51^

Our SAR also showed reduced potency when these compounds were methylated on the LHS aryl proximal to the morpholine (**6b**) and when proximal to the squaramide core (**6c**). Similarly, methylation of the RHS pyridyl at the 2-position (**14d**) was found to reduce activity while methylation at the 3-position (**14e**) abolished activity. We attempted to rationalize this by docking these compounds into our model; however, all compounds were predicted to be tolerated in the binding site (Figure S4). Interaction analyses showed predicted hydrophobic contacts between the methyl in each structure with surrounding residues. This contrasts with the observed SAR and may represent a limitation of the static docking approach used.

We also compared the binding modes of bedaquiline and the squaramide **1f** in our model (Figure 4). BDQ was found to occupy a lipophilic pocket forming stable hydrophobic interactions, consistent with its bulky diaryl scaffold. In contrast, the SQA **1f** is predicted to adopt a more polar binding mode, allowing hydrogen-bonding interactions with surrounding residues while also occupying an adjacent hydrophobic subpocket.

**Figure 4:**
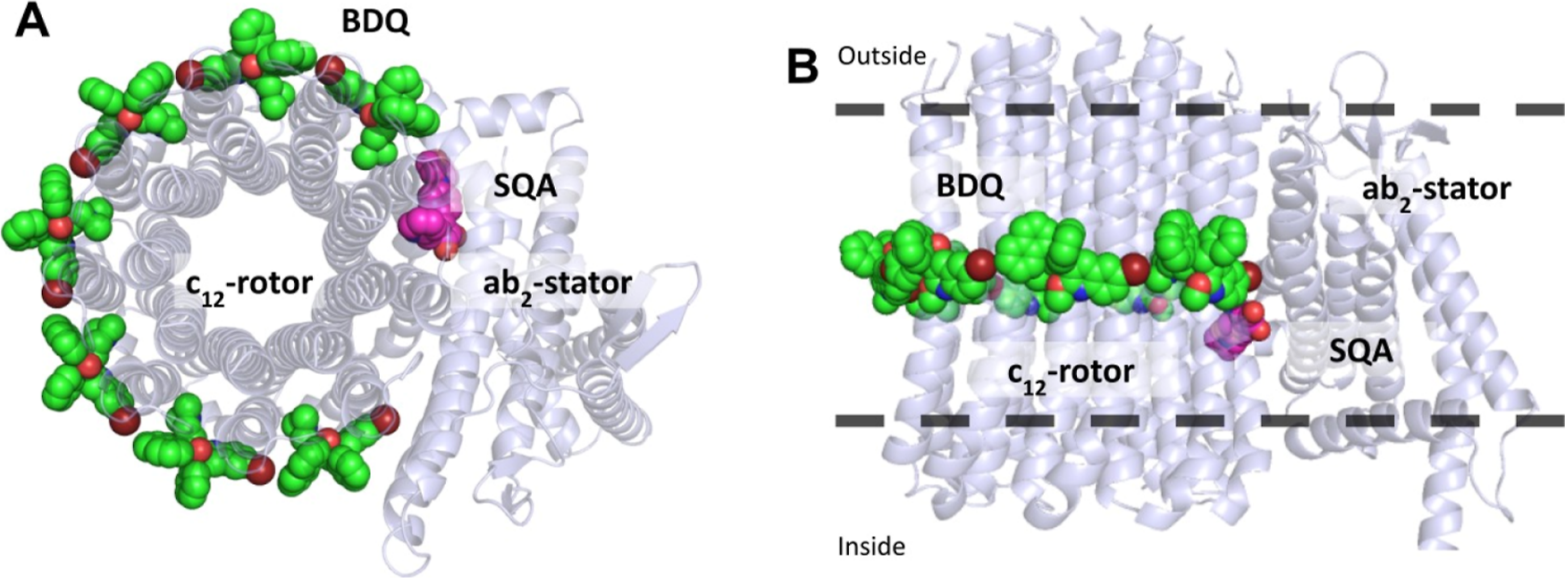
(A/B) PyMOL representation of the superposition of Mtb ATP-synthase (blue cartoon) in complex with bedaquiline (green spheres, PDB: 4V1F), and with squaramide **1f** (magenta spheres, homology models based on PBD: 8G07). The protein is viewed from the inside to the outside (A) and along the membrane (B). The position of the protein in the lipid bilayer is represented by dashed black lines.

### Target Engagement Studies

#### ATP Synthase Enzyme Inhibition Assays

In the previously reported data Tantry et al.^17^ generated drug-resistant mutant strains against the lead SQA **1f** and performed whole genome sequencing to identify resistance-associated mutations. Only five main mutations were discovered, three were mapped to a-subunit of ATP synthase, and the other two belonged to the subunit c of the enzyme.^17^ These studies suggested that both a- and c-subunits of ATP synthase played a major role in SQA binding with the enzyme. It also confirmed the proposed mechanism of action of squaramides; that is, via inhibition of ATP synthase complex upon plugging its rotation.^17^

To confirm target engagement of our newly synthesized squaramide analogues we employed biochemical assays to evaluate ATP synthase inhibition.

The first assay measured ATP synthesis inhibition to determine whether key compounds interfered with any component of the respiratory chain or specifically with the ATP synthase complex. Compounds showing activity in this assay were then assessed for their effect on oxygen consumption, which differentiates between general respiratory chain inhibition (i.e., blocking electron transport before ATP synthase) and direct inhibition of ATP synthase. ATP synthase inhibitors are expected to decrease ATP production without significantly altering oxygen consumption.

These assays utilized isolated membrane vesicles from *M. smegmatis*, which is widely used for studying membrane-associated processes. *M. smegmatis* closely resembles *M. tuberculosis* in cell wall composition, ATP synthase structure, and overall metabolism, making it a robust surrogate for biochemical and cell-based assays.^52,53^

##### Inhibition of ATP Production Using NADH as a Substrate

Two target squaramides **20a**–**b** were initially evaluated for their effect on ATP inhibition at an early stage in the project, owing to their balanced potency and metabolic stability profiles. We used two known ATP synthase inhibitors, BDQ and dicyclohexylcarbodiimide (DCCD), for comparison.^13,54^ All tested compounds were utilized at a 3 μM concentration using *M. smegmatis* membrane vesicles employing NADH as a substrate. NADH is utilized as a substrate for the NADH dehydrogenase type II (NDH-2) and is responsible for transferring electrons to menaquinone with the oxidation of NADH to NAD^+^.^55^ **20a**–**b** were evaluated for their effect on ATP synthesis utilizing NADH as a substrate (Figure 5).

**Figure 5:**
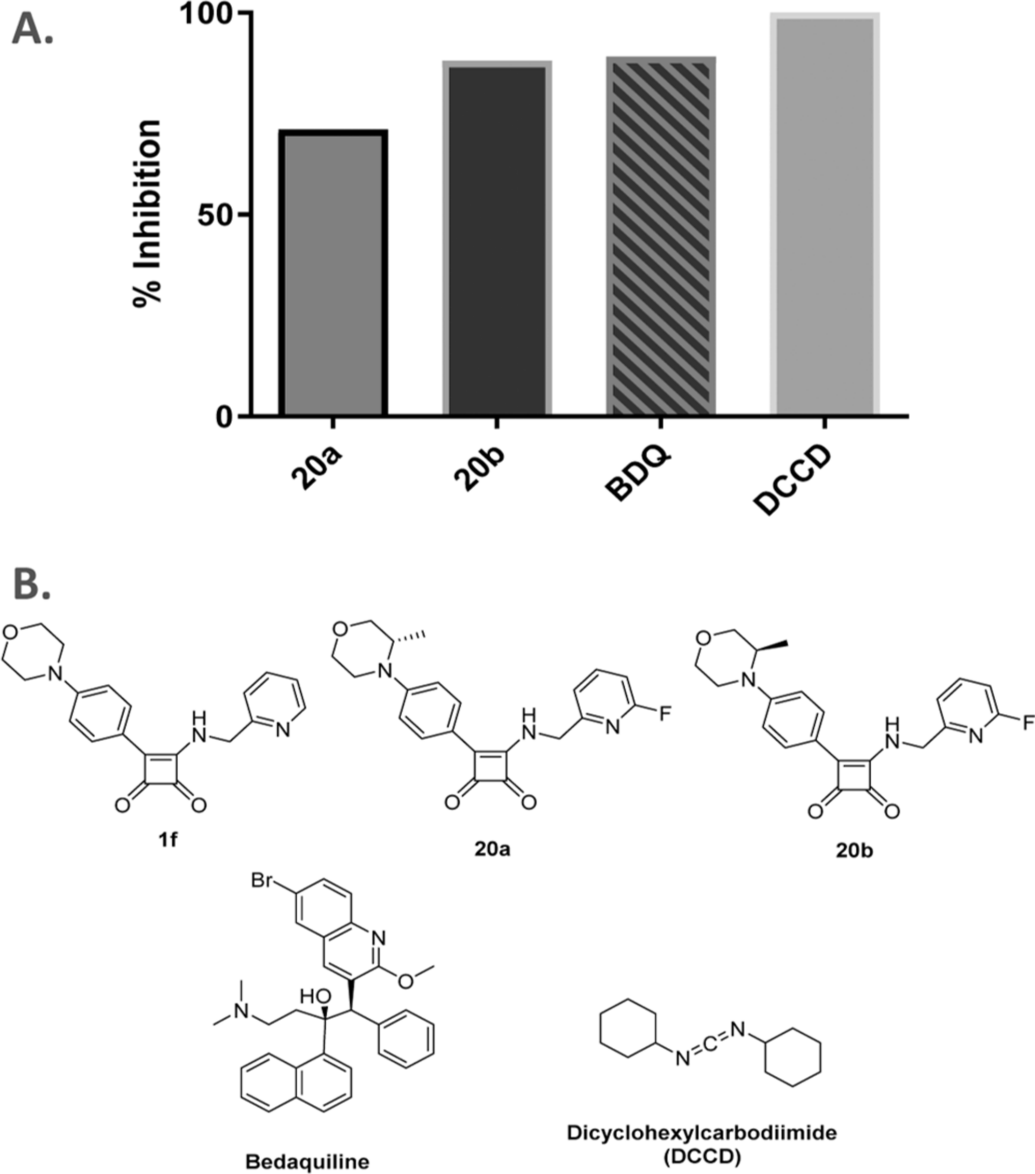
(A) Effect of **20a**–**b** on ATP production at a 3 μM concentration using NADH as a substrate. (B) Structure of tested compounds **20a**–**b**, **1f,** and the controls bedaquiline and DCCD.

DCCD was found to reduce ATP production by 50% and was taken as the benchmark for 100% inhibition to reduce the effects of background signal in this assay. Both compounds **20a** and **20b** displayed significant inhibitory effect on ATP synthesis of 71% and 88%, respectively, comparable to BDQ at the same concentration for **20b** and slightly lower than BDQ in case of **20a** (Figure 5). As BDQ is reported to inhibit ATP synthase virtually completely at lower micromolar or even submicromolar concentrations, these results confirm the activity of the analogues **20a** and **20b** on ATP production.^56,57^

##### Effect of Squaramide Compounds on Oxygen Consumption

SQAs **1f** and **20a**–**b** were investigated in an oxygen consumption assay to determine whether these compounds inhibit components of the mycobacterial respiratory chain, e.g., NDH-2, the cytochrome *bc:aa3* complex or cytochrome *bd* (Figure 6). While NDH-2 accepts electrons from NADH and transfers them onto menaquinone, the cytochrome *bc:aa3* complex or cytochrome *bd* represent two alternative routes for transferring the electrons from menaquinol onto molecular oxygen, reducing it to water.^55^ A selective ATP synthase inhibitor would not interact with NDH-2, the cytochrome *bc:aa3* complex, or cytochrome *bd* and therefore, they would not affect oxygen consumption by the mycobacterial membrane. In this assay KCN was used a positive control as it inhibits electron flow in the cytochrome *bc:aa3* complex and cytochrome *bd*.^58^ BDQ was also included as a negative control since its mechanism of action has been defined as an ATP synthase inhibitor.

**Figure 6:**
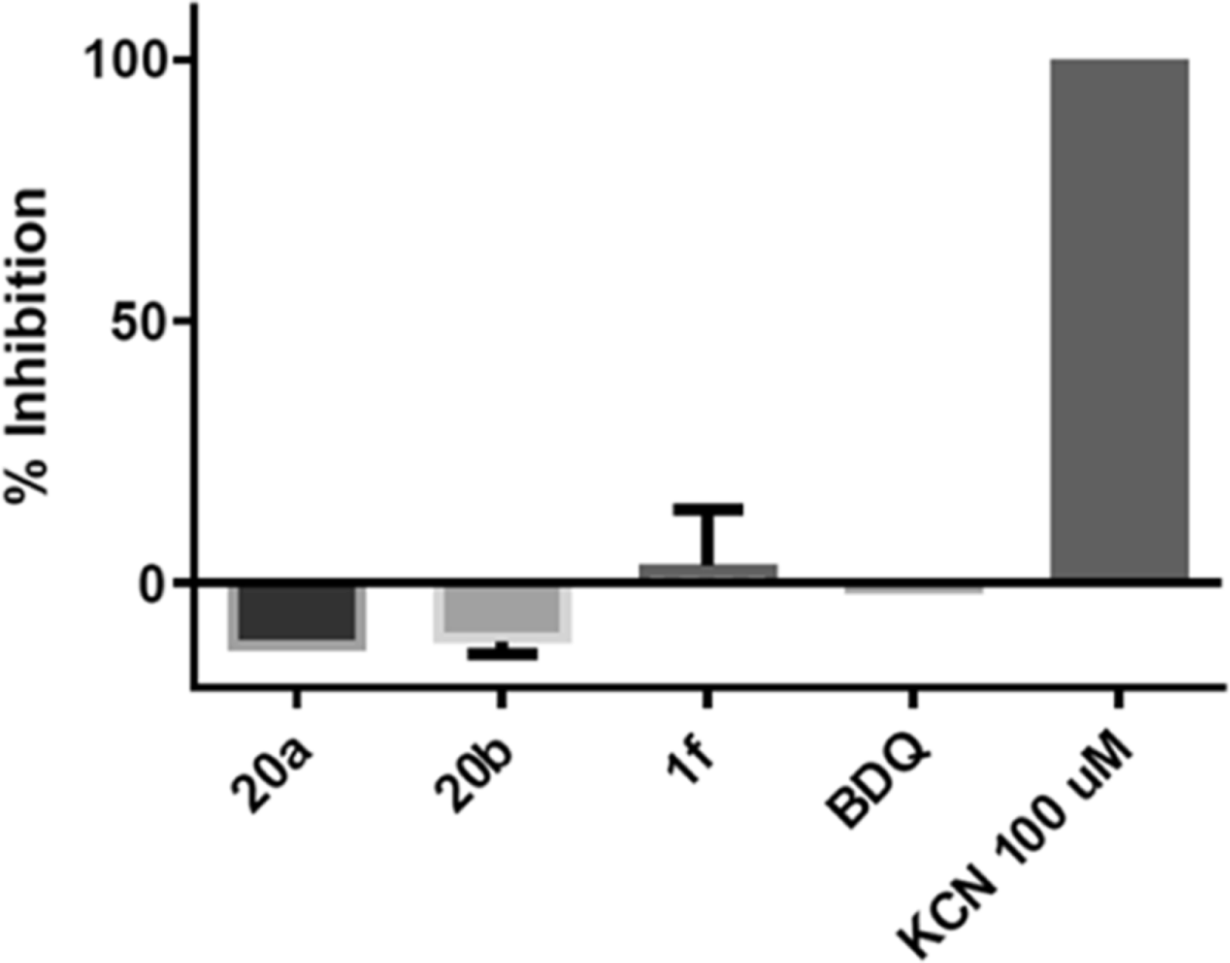
Inhibitory effect of **20a**–**b** and **1f** on oxygen consumption at 3 μM concentrations.

We found that **1f** and **20a**–**b** displayed no significant effect on oxygen consumption, supporting that these compounds do not inhibit any component of the respiratory chain and therefore confirms that ATP inhibition is due to binding with the ATP synthase enzyme complex rather than other respiratory proteins (Figure 6).

While insignificant, compounds **20a**–**b** show some moderate activation of oxygen consumption, which may indicate some uncoupling effects (Figure 6). Therefore, additional testing is required to rule out their uncoupling behavior.

Taken together, the combination of robust ATP inhibition with no significant inhibition on oxygen consumption provides compelling evidence that **20a**–**b** selectively target the ATP synthase complex rather than other components of the respiratory chain.

### In Vivo Pharmacokinetic and Pharmacodynamic Profiling

Drug exposure of the developed squaramide analogues was evaluated where the SQA **6k** was selected as it displayed good activity and metabolic stability. The lead analogue **1f** was tested alongside our developed derivatives for comparison. The novel SQA **6k** increased area under the curve (AUC_last_) to almost double that for the lead derivative **1f** at 1 mg/kg dosage (Figure S5, Table S6). Similarly, when tested at 20 mg/kg, **6k** displayed significant enhancement in bioavailability of 33%, which is also almost double that of **1f** (18.6%) (Figure S5, Table S7). Unfortunately, the analogue **20j** showed no improvement against **1f** (Table S7). Since **6k** exhibited the best in vivo pharmacokinetic profile, it was then tested at a higher dose of 100 mg/kg with and without ABT (Figure S6, Table S8). ABT is a pan cytochrome p450 inhibitor and is expected to reduce the metabolism of **6k** which could further improve its pharmacokinetic properties. This could be useful in our proof-of-concept testing in mouse models. Interestingly the (AUC_last_) of **6k** increased 10-fold with ABT as compared to without ABT at 100 mg/kg (Figures S6–S7, Table S8). In contrast, the reported exposure levels for **1f** displayed 300-fold increase in free plasma concentration when coadministered with 100 mg/kg ABT.^17^ This confirms the metabolic stability of our analogue **6k** compared to the lead **1f**.

Since the SQA **6k** displayed improved exposure compared to **1f**, it was tested in vivo in both acute and chronic TB mouse models to investigate its pharmacodynamic effects compared to **1f**. Both squaramides **6k** and **1f** were administered with ABT to enhance drug exposure. An initial experiment was conducted using the subacute mouse model of TB, which involves a lethal infectious challenge, where **6k** prevented death in two of the 5 mice. The lung colony-forming unit (CFU) counts from the mice succumbing early to infection indicated bacteriostatic activity (i.e., significantly lower lung CFU counts) compared to untreated controls and resulted in an 0.5 log_10_ CFU reduction in mice completing 4 weeks of treatment compared to baseline (D0), suggesting modest bactericidal effects with a longer treatment duration (Figure S8, Table S9). In contrast, when **1f** was evaluated in an acute TB mouse model at a dose of 100 mg/kg in combination with ABT over a 2 week treatment period, it allowed an increase in CFU compared to baseline; and at a higher **1f** dose of 200 mg/kg with ABT, only a modest reduction of <0.5 log_10_ CFU was observed.^17^ This suggests limited dose dependence and is consistent with its reported metabolic instability. Although a direct comparison cannot be achieved due to the different models and treatment regimens used, the in vivo efficacy of **6k** confirms its metabolic stability and exposure profile.

A follow-up experiment was then performed to further confirm the activity of **6k** and compare it to that of **1f**. This second study used the chronic mouse infection model of TB, in which untreated mice or mice treated with agents that have limited activity against actively multiplying bacteria and/or delayed onset of bactericidal effects will not succumb to the infection. The mice were infected with *M. tuberculosis* and then were given the treatment 6 weeks later. The treatment lasted for 4 weeks for all tested compounds. BDQ was bactericidal in this model, which served as a useful model to compare the activity of the two squaramides. The number of CFU implanted and the number present at the start of treatment were counted.

At the end of 4 weeks of treatment, the mean (±SD) CFU count in untreated mice was 5.72 ± 0.20 log_10_ CFU/lung, indicating a relatively stable chronic infection with all the untreated mice surviving until the end of experiment (Figure 7, Table S10). Mice treated with BDQ at 25 mg/kg had a significantly lower mean CFU count, at 3.76 ± 0.20 log_10_ CFU/lung, compared to untreated controls (*p* < 0.0001). Mice treated with **6k** alone at 100 mg/kg had a mean log_10_ CFU/lung of 5.97 ± 0.09, which was not significantly different from that of untreated controls (*p* = 0.1486) (Figure 7, Table S10). In mice treated with **6k** (100 mg/kg) + **ABT** (100 mg/kg) or **1f** (100 mg/kg) + **ABT** (100 mg/kg), the mean CFU counts were 5.18 ± 0.17 and 5.22 ± 0.20 log_10_ CFU/lung, respectively; and both means were significantly lower when compared to untreated controls (*p* = 0.0004 and *p* = 0.0012, respectively) (Figure 7, Table S10), indicating a modest bactericidal effect for both compounds administered with ABT. However, the mean CFU counts in the **6k** + **ABT** and **1f** + **ABT** arms were not significantly different from each other (*p* = 0.99).

**Figure 7:**
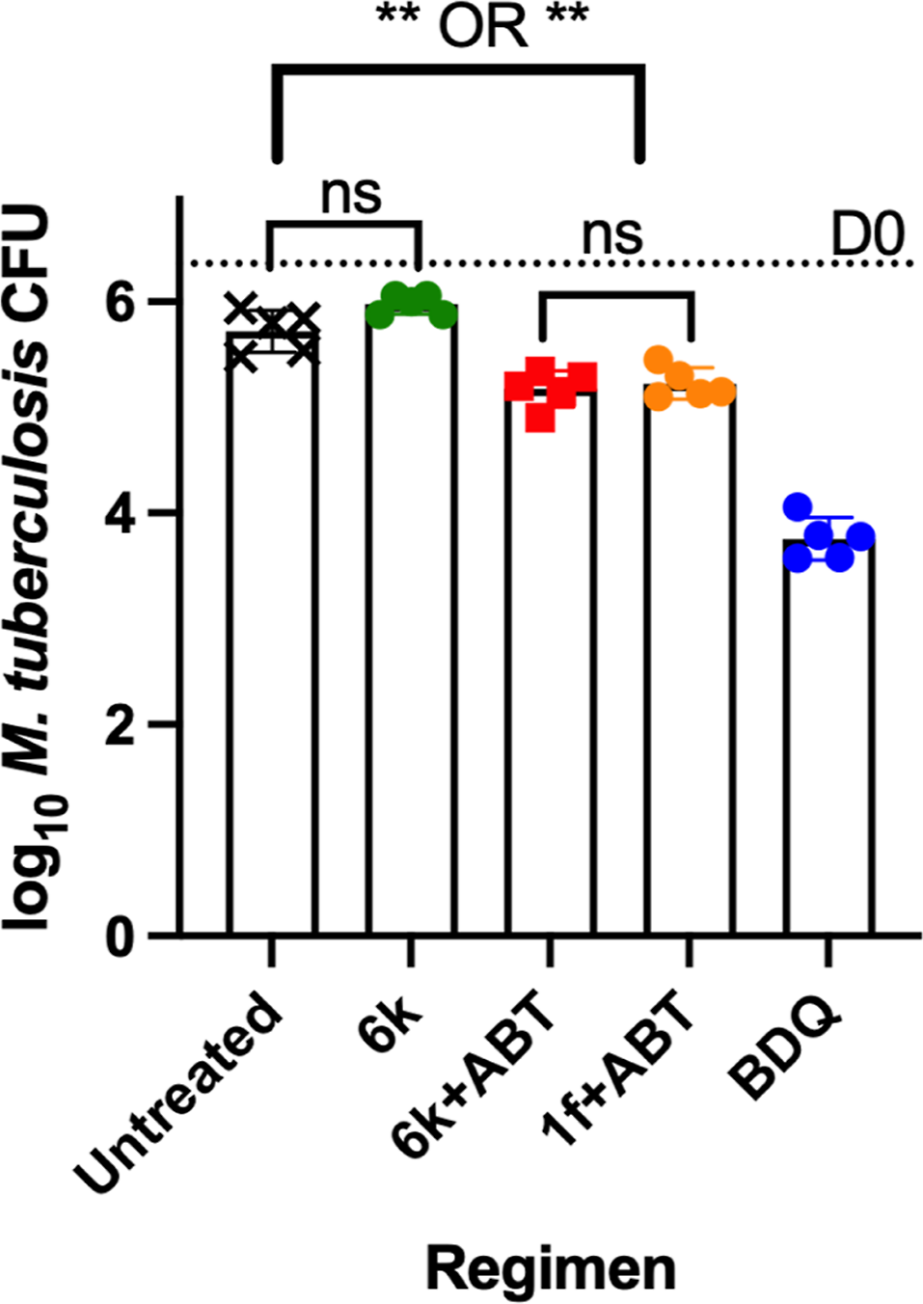
Mean log_10_ CFU/lung in mice at the end of 4 weeks of treatment. Black symbols, untreated controls; green, **6k** alone; red, **6k** + ABT; orange, **1f** + ABT; blue, BDQ alone. **1f**, **6k,** and ABT were used at 100 mg/kg and **BDQ** was used at 25 mg/kg. All drugs were administered daily 5 days per week. ABT was given 2 h before **1f** and **6k** **p = 0.0012, Untreated *v/s* **1f** + ABT; *p* = 0.0004, untreated v/s **6k** + ABT.

## Conclusion

In summary, the SQA **1f** was previously reported as a mycobacterial ATP synthase inhibitor with potent anti-TB activity but moderate-to-poor metabolic stability.^17^ Herein, the original squaramide series has been expanded and several more potent inhibitors were discovered alongside derivatives with maintained activity and improved metabolic properties. Docking studies were performed using our newly built homology model and the recently published cryo-EM structure of Msm ATP synthase, which confirmed that our compounds display a similar binding mode to **1f** and modeling could rationalize the observed SAR profile. The selected lead SQAs of the series exhibit excellent ATP synthase enzyme inhibition with no inhibition of respiratory chain components. In vivo PK demonstrated that **6k** achieved improved exposure but **6k** did not by itself produce a bactericidal effect. When coadministered with ABT, **6k** exposure was bactericidal, and at 100 mg/kg (with ABT) **6k** and **1f** were similarly active. This comparison was made at a single dose; an in vivo dose–response (ED_50_/IC_50_) was not performed, so relative potency outside this ABT-boosted condition remains unresolved. Overall, the improved DMPK of **6k** did not translate into superior efficacy under the conditions tested, underscoring the need for further optimization to address metabolic liabilities and enhance exposure, particularly in light of activity against BDQ-resistant strains.

## Experimental Section

### Microbiology Methods (Alamar Blue Assay)

The IC_50_ Assay is done in two parts: In the first stage, the IC_50_ plates are set up under sterile conditions in a biological safety cabinet. The second stage includes the inoculation of the plates with Mtb, which is performed in accordance with the Containment Suite Guidelines. Stage 1: Plate preparation. Stock solutions for drug and controls were prepared as a 10 mM stock solution of the drug in DMSO. For a starting concentration of 20 μM, the 10 mM stock solution was diluted 1 in 25 in DMSO to 400 μM. [20 μL (10 mM) + 480 μL (DMSO)], the 400 μM solution was then serially diluted to 1 in 2 [(100 μL compound) + (100 μL DMSO)] in a 96-well plate. 2.5 μL of each dilution was added in triplicate to wells containing 47.5 μL of Mtb culture media. For the positive control (isoniazid) preparation, 50 μL of Isoniazid (162 μM in media) and 50 μL of Mtb inoculum (81 μM in well) were added to the wells. However, for the negative media control, 50 μL of Mtb media (+5% DMSO) was used in addition to 50 μL of Mtb inoculum. 200 μL of water was also added to the perimeter well to prevent the plates from drying out during the incubation period. The positive isoniazid control was prepared as a 10 mM Stock Solution that was diluted 1 in 61.728 in Mtb media to give a 162 μM working stock solution. An isoniazid control IC_50_ assay is also run alongside test compounds, where the 162 μM working Stock Solution was serially diluted 1 in 3 in (100 μL of working solution +200 μL of media) Mtb media. Each compound is plated in triplicate (i.e., 3 × 50 μL). Stage 2: Plate inoculation with TB. A 2–3 week-old culture was used, and its optical density (OD) was measured. Since the OD should be between 0.6 and 1.0. The cultures were then diluted accordingly with Mtb culture media to give an OD 0.3; then 1 mL was taken and then added to 29 mL of Mtb culture media. 50 μL of inoculum was added to all wells except those containing water. The plates were then covered with a lid, placed into sealable plastic bags, and then were put into a sealable plastic container and incubated at 37 °C for 7 days. On the seventh day, Alamar Blue dye was added to the plates. The Alamar Blue solution was prepared by mixing Alamar Blue with 10% Tween in a falcon tube, where it was diluted by mixing one part 10% Tween and two parts Alamar Blue. 20 μL of Alamar Blue working solution was then added to the wells using a multichannel pipet. No Alamar Blue was added to the well containing water. The plates were then returned to the bag and placed in the incubator at 37 °C overnight. The plates were collected the next day (day 8) and 50 μL of 10% paraformaldehyde solution was added to all wells of each plate. The plates were then placed back in the bag and put in the incubator for at least 2 h. Following that, the plates were read using a plate reader and the results were then given as % Mtb Activity.

### Pharmacodynamic Studies in Mice

(A) Chronic murine infection model Female BALB/c mice were aerosol-infected with low-dose aerosol infection to implant about 100 CFU of *M. tuberculosis* H37Rv on D-42. Treatment started 6 weeks later (D0) and lasted for 1 month (4 weeks). Untreated mice were sacrificed for lung CFU counts on D-41 and on D0 to determine the number of CFU implanted and the number present at the start of treatment, respectively. Treated mice were sacrificed after 4 weeks of treatment with the remaining untreated mice to determine the effect of treatment. (B) Drug preparation and administration to mice: Doses tested were (in mg/kg): **BDQ** (25), **1f** (100) and **6k** (100). The volume of administration was 0.2 mL. Mice weighed about 20 g. **BDQ** was prepared in acidified hydroxypropyl-β-cyclodextrin solution, ABT was prepared in 0.5% methylcellulose, **1f** and **6k** were prepared in carboxymethyl cellulose (0.5%), benzyl alcohol (0.5%), Tween 80 (0.4%), and NaCl (0.9%), with stirring and sonication. All drugs were administered once daily by gavage. ABT was dosed 2 h before **1f** and **6k**. All animal procedures were approved by the Johns Hopkins University Animal Care and Use Committee (ACUC; protocol numbers MO21M389 and MO24M289) and were conducted in accordance with relevant national and international guidelines. The Johns Hopkins animal care program complies with the Animal Welfare Act regulations and Public Health Service Policy and is accredited by the Association for Assessment and Accreditation of Laboratory Animal Care (AAALAC) International. (C) Evaluation of drug efficacy in mice: Efficacy determinations were based on lung CFU counts after 4 weeks of treatment. To determine CFU counts, lung homogenates were plated in serial 10-fold dilutions on selective 7H11 plates supplemented with 10% oleic acid–albumin–dextrose–catalase. Charcoal (0.4%)-containing plates were used to mitigate effects of drug carryover. (D) Statistical analysis: Group means were compared using one-way ANOVA with Tukey’s post-test to control for multiple comparisons.

### General Chemistry Methods

All commercially available chemicals were purchased from Sigma-Aldrich, Fluorochem, Manchester Organics, Enamine, Apollo Scientific, or Alfa Aesar and were used with no further purification.

^1^H and ^13^C NMR spectra were recorded using a Bruker 400 MHz NMR spectrometer. Chemical shifts are reported as parts per million (ppm) on the δ scale relative to the internal standard, tetramethylsilane (TMS). Coupling constants (*J*) are reported in Hertz (Hz). The residual solvent signals were used as references and the chemical shifts converted to the TMS scale. Analytical thin-layer chromatography was performed on Merck silica gel 60 F_254_ plates. Visualization was achieved via short wave UV light. Flash chromatography was performed on Sigma-Aldrich silica gel 60 Å (230–400 mesh particle size) by a standard technique. All mass spectrometry and elemental microanalysis was done at the Analytical services in the Chemistry department of the University of Liverpool. Mass spectrometry data were recorded using Electron spray ionization (ES+) or Chemical ionization (CI+) using an Agilent QTOF 6540 mass spectrometer using methanol or acetonitrile as the solvent. UV detector recorded signals at 254 nm. Melting points were recorded manually using a Stuart melting point apparatus (SMP3) and results given in degrees Celsius (°C). HPLC was carried out using an Agilent 1200 HPLC equipped with a ZORBAX Eclipse Plus C18 column (4.6 mm × 10 mm, 3.5 μm) at 25 °C. The flow rate was 1 mL/min for 17 min using MeCN/water with compounds dissolved in methanol. A UV detector recorded signals at 254 nm. Method: hold at 2% MeCN for 1 min, then 2–98% MeCN for 11 min, then hold at 98% MeCN for 3 min, then from 98 to 2% MeCN for 1 min, then hold at 2% MeCN until the run finishes. All compounds were ≥95% pure by HPLC analysis and/or elemental analysis except **6j** and **20d**, none of which are critical for the SAR. Optical rotations were measured on Bellingham + Stanley Single Wavelength Polarimeter ADP450, [α]_D_ was calculated using the following formula [α]_D_ = 100 α/l c, where α is the reading from the instrument, positive or negative, l is the length of the sample tube in dm (1 dm = 10 cm), and c is the concentration of the sample in g 100 mL^–1^. The sample tube length of this polarimeter was 2.5 cm (0.25 dm).

^1^H and ^13^C NMR spectra for all final compounds and HPLC chromatograms of lead compounds are provided in the Supporting Information (Figures S16–S64).

### General Procedures

The general procedures and analysis for the synthesis of the LHS substituents **3b**–**s**, the intermediate squaramides **4a**–**s**, compounds **8–10,** and the RHS amines **16**, **19a**–**g** are included in the Supporting Information.

### General Procedure A for Synthesis of Analogues **6a–s**

The chloro squarate derivative **4a**–**s** (1.0 equiv) was dissolved in 1,4-dioxane and cooled to 0 °C. Triethylamine (1.5 equiv or 2.5 equiv if amine salt is used) was added dropwise followed by addition of amine (1.5 equiv). The solution was then warmed to room temperature and allowed to stir for 1–3 h. The reaction mixture was concentrated in vacuo, washed with water, filtered, and dried under vacuum. The product was purified using flash column chromatography on silica gel using MeOH/DCM as an eluent system to afford products **6a**–**s**.

#### 3-(4-Morpholinophenyl)-4-((pyridin-2-ylmethyl)­amino)­cyclobut-3-ene-1,2-dione (**1f/6a**)

Yellow solid, 58–70% yield (0.07–0.17 g). ^1^H NMR (400 MHz, DMSO): δ 9.40 (s, 1H), 8.56 (d, *J* = 4.2 Hz, 1H), 7.96 (d, *J* = 8.9 Hz, 2H), 7.82 (td, *J* = 7.7, 1.7 Hz, 1H), 7.44 (d, *J* = 7.7 Hz, 1H), 7.33 (dd, *J* = 7.2, 5.0 Hz, 1H), 7.08 (d, *J* = 8.9 Hz, 2H), 5.01 (s, 2H), 3.84–3.67 (m, 4H), 3.32–3.27 (m, 4H). ^13^C NMR (101 MHz, DMSO): δ 191.98, 189.76, 178.49, 163.87, 157.38, 152.50, 149.36, 137.00, 128.51, 122.75, 121.91, 120.23, 66.42, 49.40, 47.56. HRMS (ES+) *m*/*z*: calcd for C_20_H_20_N_3_O_3_ [M + H]^+^: 350.1499; found, 350.1508 (Diff −2.49 ppm). IR ν_max_/cm^–1^: (solid) 3264 (s), 3.50 (m), 2947 (m), 1776 (m), 1707 (s), 1617 (s), 1586 (s), 1344 (s), 1235 (s). Anal. Calcd for C_20_H_19_N_3_O_3_: C, 68.75; H, 5.48; N, 12.03. Found C, 68.43; H, 5.35; N, 11.81. Purity HPLC 95.86%, *R*_*t*_ = 6.99 min.

#### 3-(3-Methyl-4-morpholinophenyl)-4-((pyridin-2-ylmethyl)­amino)­cyclobut-3-ene-1,2-dione (**6b**)

Brown solid, 23% yield (0.023 g). ^1^H NMR (400 MHz, DMSO): δ 9.50 (t, *J* = 5.9 Hz, 1H), 8.58–8.55 (m, 1H), 7.91 (dd, *J* = 8.4, 1.9 Hz, 1H), 7.86 (d, *J* = 1.5 Hz, 1H), 7.82 (td, *J* = 7.8, 1.9 Hz, 1H), 7.45 (d, *J* = 7.8 Hz, 1H), 7.36–7.31 (m, 1H), 7.14 (d, *J* = 8.4 Hz, 1H), 5.03 (d, *J* = 5.9 Hz, 2H), 3.81–3.67 (m, 4H), 2.97–2.88 (m, 4H), 2.32 (s, 3H). ^13^C NMR (101 MHz, DMSO): δ 193.14, 189.42, 179.22, 162.76, 157.70, 153.82, 149.76, 137.56, 132.33, 129.48, 125.77, 124.10, 123.20, 122.10, 119.28, 66.85, 51.77, 18.40. ^13^C NMR (101 MHz, DMSO): δ 193.14, 189.42, 179.22, 162.76, 157.70, 153.82, 149.76, 137.56, 132.33, 129.48, 125.77, 124.10, 123.20, 122.10, 119.28, 66.85, 51.77, 49.31, 18.40. HRMS (ES+) *m*/*z*: calcd for C_21_H_22_N_3_O_3_ [M + H]^+^: 364.1656; found, 364.1665 (Diff −2.47 ppm). mp 100–102 °C. Purity HPLC 95.11%, *R*_*t*_ = 7.83 min.

#### 3-(2-Methyl-4-morpholinophenyl)-4-((pyridin-2-ylmethyl)­amino)­cyclobut-3-ene-1,2-dione (**6c**)

Dark yellow solid, 34% yield (0.043 g). ^1^H NMR (400 MHz, DMSO): δ 9.64 (t, *J* = 6.4 Hz, 1H), 9.22 (t, *J* = 6.0 Hz, 1H), 8.56 (d, *J* = 4.3 Hz, 1H), 7.82 (td, *J* = 7.7, 1.5 Hz, 1H), 7.43 (d, *J* = 7.7 Hz, 1H), 7.38–7.29 (m, 2H), 6.96–6.81 (m, 2H), 4.96 (d, *J* = 6.4 Hz, 2H), 3.77–3.71 (m, 4H), 3.26–3.19 (m, 4H), 2.41 (s, 3H). ^13^C NMR (101 MHz, DMSO): δ 193.82, 188.92, 180.64, 167.40, 157.77, 152.57, 149.64, 138.62, 137.59, 129.09, 123.14, 121.99, 119.40, 116.53, 111.89, 66.38, 49.18, 47.87, 21.63. HRMS (ES+) *m*/*z*: calcd for C_21_H_22_N_3_O_3_ [M + H]^+^: 364.1656; found, 364.1659 (Diff −0.79 ppm). mp 110–112 °C. Purity HPLC 95.20%, *R*_*t*_ = 7.06 min.

#### 3-(2-Chloro-4-morpholinophenyl)-4-((pyridin-2-ylmethyl)­amino)­cyclobut-3-ene-1,2-dione (**6d**)

Dark yellow solid, 60% yield (0.086 g). ^1^H NMR (400 MHz, DMSO): δ 9.03 (t, *J* = 6.3 Hz, 1H), 8.57–8.54 (m, 1H), 7.82 (td, *J* = 7.8, 1.8 Hz, 1H), 7.67 (d, *J* = 8.8 Hz, 1H), 7.41 (d, *J* = 7.8 Hz, 1H), 7.34–7.30 (m, 1H), 7.12 (d, *J* = 2.5 Hz, 1H), 7.05 (dd, *J* = 8.8, 2.5 Hz, 1H), 4.98 (d, *J* = 6.3 Hz, 2H), 3.76–3.72 (m, 4H), 3.30–3.26 (m, 4H). ^13^C NMR (101 MHz, DMSO): δ 193.96, 188.42, 179.74, 162.92, 157.64, 153.40, 149.67, 137.51, 132.74, 130.50, 123.08, 121.84, 117.92, 114.95, 113.05, 66.22, 49.22, 47.50. HRMS (ES+) *m*/*z*: calcd for C_20_H_19_ClN_3_O_3_ [M + H]^+^: 384.1109; found, 384.1121 (Diff: −3.12 ppm). Mp 142–144 °C. Anal. Calcd for C_20_H_18_^35^ClN_3_O_3_ (+0.25H_2_O): C, 61.86; H, 4.80; N, 10.82. Found C, 61.96; H, 4.79; N, 10.61.

#### 3-(2-Fluoro-4-morpholinophenyl)-4-((pyridin-2-ylmethyl)­amino)­cyclobut-3-ene-1,2-dione (**6e**)

Dark yellow crystals, 34% yield (0.02 g). ^1^H NMR (400 MHz, CDCl_3_): δ 8.93 (s, 1H), 8.61 (d, *J* = 4.7 Hz, 1H), 8.39 (t, *J* = 8.9 Hz, 1H), 7.72 (tt, *J* = 6.1, 3.1 Hz, 1H), 7.68–7.61 (m, 1H), 7.33 (d, *J* = 7.7 Hz, 1H), 6.72 (dd, *J* = 8.9, 2.4 Hz, 1H), 6.60 (dd, *J* = 15.6, 2.4 Hz, 1H), 5.14 (d, *J* = 5.3 Hz, 2H), 3.90–3.77 (m, 4H), 3.40–3.16 (m, 4H). ^13^C NMR (101 MHz, CDCl_3_): δ 184.71, 177.29, 169.27, 155.05, 149.39, 146.31, 137.15, 130.20, 122.93, 122.04, 110.30, 100.67, 66.38, 48.20, 47.35. HRMS (ES+) *m*/*z*: calcd for C_20_H_19_FN_3_O_3_ [M + H]^+^: 368.1405; found, 368.1409 (Diff: −1.08 ppm). mp 174–176 °C. Purity HPLC 98.08%, *R*_*t*_ = 7.56 min.

#### 3-(2-Methoxy-4-morpholinophenyl)-4-((pyridin-2-ylmethyl)­amino)­cyclobut-3-ene-1,2-dione (**6f**)

Yellow solid, 24% yield (0.048 g). ^1^H NMR (400 MHz, DMSO): δ 8.63–8.59 (m, 1H), 8.30 (t, *J* = 5.9 Hz, 1H), 8.16 (d, *J* = 8.8 Hz, 1H), 7.84 (td, *J* = 7.8, 1.8 Hz, 1H), 7.42 (d, *J* = 7.8 Hz, 1H), 7.38–7.31 (m, 1H), 6.68 (dd, *J* = 8.8, 2.2 Hz, 1H), 6.60 (d, *J* = 2.2 Hz, 1H), 5.03 (d, *J* = 5.9 Hz, 2H), 3.97 (s, 3H), 3.78–3.72 (m, 4H), 3.37–3.33 (m, 4H). ^13^C NMR (101 MHz, DMSO): δ 191.81, 189.24, 177.96, 162.02, 157.59, 157.17, 155.18, 149.48, 137.58, 129.67, 123.06, 121.81, 109.66, 107.00, 97.58, 66.32, 56.47, 48.28, 47.51, 31.30, 22.83. HRMS (ES+) *m*/*z*: calcd for C_21_H_21_N_3_NaO_4_ [M + Na]^+^: 402.1424; found, 402.1438 (Diff: −3.48 ppm). IR ν_max_/cm^–1^: (solid) 3338 (s), 3093 (s), 2927 (m), 1767 (s), 1708 (s), 1591 (s), 1567 (s), 1305 (s), 1248 (s). mp 212–214 °C. Anal. Calcd for C_21_H_21_N_3_O_4_: C, 66.48; H, 5.58; N, 11.08. Found C, 66.32; H, 5.86; N, 10.24. Purity HPLC 96.87%, *R*_*t*_ = 7.89 min.

#### (*S*)-3-(4-(3-Methylmorpholino)­phenyl)-4-(pyridin-2-ylmethylamino)­cyclobut-3-ene-1,2-dione (**6g**)

Orange solid, 63% yield (0.016 g). ^1^H NMR (400 MHz, DMSO): δ 9.36 (s, 1H), 8.57–8.53 (m, 1H), 7.95 (d, *J* = 9.0 Hz, 2H), 7.81 (td, *J* = 7.7, 1.8 Hz, 1H), 7.48–7.40 (m, 1H), 7.35–7.26 (m, 1H), 7.01 (d, *J* = 9.0 Hz, 2H), 5.00 (s, 2H), 4.14–4.04 (m, 1H), 3.95 (dd, *J* = 11.3, 3.4 Hz, 1H), 3.74 (d, *J* = 11.3 Hz, 1H), 3.68 (dd, *J* = 11.3, 2.6 Hz, 1H), 3.55–3.50 (m, 2H), 3.06 (tt, *J* = 10.7, 5.3 Hz, 1H), 1.08 (d, *J* = 6.6 Hz, 3H). ^13^C NMR (101 MHz, DMSO): δ 189.50, 188.70, 178.47, 163.52, 161.69, 157.90, 151.58, 149.71, 137.54, 128.63, 123.14, 122.01, 113.92, 71.01, 66.51, 49.24, 48.69, 41.33, 33.04, 12.08. HRMS (ES+) *m*/*z*: calcd for C_21_H_22_N_3_O_3_ [M + H]^+^: 364.1656; found, 364.166 (Diff −1.07 ppm). mp 189–190 °C. Anal. Calcd for C_21_H_21_N_3_O_3_(+0.25 H_2_O): C, 68.56; H, 5.89; N, 11.42. Found C, 68.50; H, 5.81; N, 11.15. Purity HPLC 95.11%, *R*_*t*_ = 7.57 min.

#### (*R*)-3-(4-(3-Methylmorpholino)­phenyl)-4-((pyridin-2-ylmethyl)­amino)­cyclobut-3-ene-1,2-dione (**6h**)

Yellow solid, 71% yield (0.047 g). ^1^H NMR (400 MHz, DMSO): δ 9.36 (s, 1H), 8.57–8.54 (m, 1H), 7.95 (d, *J* = 9.0 Hz, 2H), 7.82 (td, *J* = 7.7, 1.8 Hz, 1H), 7.43 (d, *J* = 7.7 Hz, 1H), 7.36–7.29 (m, 1H), 7.01 (d, *J* = 9.0 Hz, 2H), 5.01 (d, *J* = 3.7 Hz, 2H), 4.16–4.04 (m, 1H), 3.96 (dd, *J* = 11.2, 3.4 Hz, 1H), 3.74 (d, *J* = 11.2 Hz, 1H), 3.69 (dd, *J* = 11.2, 2.7 Hz, 1H), 3.55 (td, *J* = 11.6, 2.7 Hz, 1H), 3.51–3.44 (m, 1H), 3.07 (td, *J* = 12.3, 3.8 Hz, 1H), 1.09 (d, *J* = 6.6 Hz, 3H). ^13^C NMR (101 MHz, DMSO): δ 192.12, 189.48, 178.45, 163.53, 157.93, 151.56, 149.72, 137.52, 128.63, 123.13, 122.02, 119.03, 113.91, 71.01, 66.52, 49.25, 48.69, 41.34, 12.09. HRMS (ES+) *m*/*z*: calcd for C_21_H_22_N_3_O_3_ [M + H]^+^: 364.1656; found, 364.1657 (Diff −0.37 ppm). IR ν_max_/cm^–1^: (solid) 3257 (s), 3020 (m), 2963 (m), 2939 (s), 1769 (m), 1705 (s), 1615 (s), 1581 (s), 1344 (s), 1227 (s). mp 189–192 °C. Anal. Calcd for C_21_H_21_N_3_O_3_: C, 69.41; H, 5.82; N, 11.56. Found C, 68.94; H, 5.81; N, 11.72. [α]_D_ = +28 (*c* = 1, MeOH). Purity HPLC 100%, *R*_*t*_ = 7.65 min.

#### 3-(4-(3-Propylmorpholino)­phenyl)-4-((pyridin-2-ylmethyl)­amino)­cyclobut-3-ene-1,2-dione (**6i**)

Dark orange solid in a 35% yield (0.014 g). ^1^H NMR (400 MHz, DMSO): δ 9.34 (t, *J* = 6.1 Hz, 1H), 8.57–8.53 (m, 1H), 7.94 (d, *J* = 9.0 Hz, 2H), 7.81 (td, *J* = 7.8, 1.7 Hz, 1H), 7.43 (d, *J* = 7.8 Hz, 1H), 7.35–7.29 (m, 1H), 6.99 (d, *J* = 9.0 Hz, 2H), 5.00 (dd, *J* = 6.1, 1.7 Hz, 1H), 3.97–3.82 (m, 4H), 3.64–3.54 (m, 2H), 3.16–3.04 (m, 1H), 1.38–1.16 (m, 4H), 0.86 (t, *J* = 7.0 Hz, 3H). ^13^C NMR (101 MHz, DMSO): δ 192.00, 189.51, 178.39, 163.56, 157.91, 151.76, 149.71, 137.54, 128.67, 123.14, 122.02, 118.67, 113.74, 68.05, 66.41, 53.16, 49.23, 41.74, 28.73, 19.71, 14.46. HRMS (ES+) *m*/*z*: calcd for C_23_H_26_N_3_O_3_ [M + H]^+^: 392.1969; found, 392.1967 (Diff: 0.5 ppm). Anal. Calcd for C_23_H_25_N_3_O_3_: C, 70.57; H, 6.44; N, 10.73. Found C, 69.28; H, 6.38; N, 10.30. Purity HPLC 95.75%, *R*_*t*_ = 8.63 min.

#### 3-(4-(2,6-Dimethylmorpholino)­phenyl)-4-((pyridin-2-ylmethyl)­amino)­cyclobut-3-ene-1,2-dione (**6j**)

Yellow solid, 12% yield (0.007 g). ^1^H NMR (400 MHz, DMSO): δ 9.39 (s, 1H), 8.57–8.52 (m, 1H), 7.94 (d, *J* = 8.9 Hz, 2H), 7.81 (td, *J* = 7.7, 1.8 Hz, 1H), 7.43 (d, *J* = 7.7 Hz, 1H), 7.35–7.29 (m, 1H), 7.07 (d, *J* = 8.9 Hz, 2H), 5.01 (s, 2H), 4.17–4.14 (m, 2H), 3.85–3.77 (m, 4H), 1.17 (d, *J* = 6.2 Hz, 6H). Due to insufficient signal intensity, a ^13^C NMR spectrum could not be obtained; however, the structure is supported by ^1^H NMR and high-resolution mass spectrometry data. HRMS (ES+) *m*/*z*: calcd for C_22_H_24_N_3_O_3_ [M + H]^+^: 378.1812; found, 378.1813 (Diff: −0.26 ppm).

#### 3-(4-(*Cis* 2,6-Dimethylmorpholino)­phenyl)-4-((pyridin-2-ylmethyl) amino)­cyclobut-3-ene-1,2-dione (**6k**)

Yellow solid, 24% yield (0.035 g). ^1^H NMR (400 MHz, DMSO): δ 9.38 (s, 1H), 8.58–8.54 (m, 1H), 7.95 (d, *J* = 8.8 Hz, 2H), 7.82 (td, *J* = 7.8, 1.6 Hz, 1H), 7.44 (d, *J* = 7.8 Hz, 1H), 7.35–7.29 (m, 1H), 7.07 (d, *J* = 8.8 Hz, 2H), 5.01 (s, 2H), 3.73–3.62 (m, 2H), 2.39 (t, *J* = 11.4 Hz, 2H), 1.18 (d, *J* = 6.1 Hz, 6H). ^13^C NMR (101 MHz, DMSO): δ 192.21, 189.48, 178.63, 171.64, 158.30, 149.72, 137.52, 128.49, 123.13, 122.03, 114.36, 79.10, 71.30, 52.51, 19.25. mp 237–239 °C. HRMS (ES+) *m*/*z*: calcd for C_22_H_24_N_3_O_3_ [M + H]^+^: 378.1812; found, 378.1810 (Diff: 0.53 ppm). Purity HPLC 99.86%, *R*_*t*_ = 8.08 min.

#### Synthesis of 3-(4-((*2S*,*6S*)-2,6-Dimethylmorpholino)­phenyl)-4-((pyridin-2-ylmethyl) amino)­cyclobut-3-ene-1,2-dione (**6l**)

Orange solid, 50% yield (0.08 g). ^1^H NMR (400 MHz, DMSO): δ 9.36 (t, *J* = 6.0 Hz, 1H), 8.56 (d, *J* = 4.0 Hz, 1H), 7.94 (d, *J* = 8.8 Hz, 2H), 7.82 (td, *J* = 7.8, 1.6 Hz, 1H), 7.44 (d, *J* = 7.8 Hz, 1H), 7.37–7.29 (m, 1H), 7.03 (d, *J* = 8.8 Hz, 2H), 5.01 (d, *J* = 6.0 Hz, 2H), 4.11–4.01 (m, 2H), 3.42 (dd, *J* = 12.4, 2.7 Hz, 2H), 3.09 (dd, *J* = 12.4, 6.5 Hz, 2H), 1.19 (d, *J* = 6.3 Hz, 6H). ^13^C NMR (101 MHz, DMSO): δ 192.07, 189.49, 178.40, 163.50, 157.93, 152.84, 149.72, 137.53, 128.58, 123.14, 122.03, 119.01, 113.95, 66.03, 51.83, 49.23, 18.29. HRMS (ES+) *m*/*z*: calcd for C_22_H_24_N_3_O_3_ [M + H]^+^: 378.1812; found, 378.1814 (Diff: −0.53 ppm). mp 210–212 °C. Purity HPLC 95.03%, *R*_*t*_ = 7.93 min.

#### 3-(4-((2*R*,6*R*)-2,6-Dimethylmorpholino)­phenyl)-4-((pyridin-2-ylmethyl) amino)­cyclobut-3-ene-1,2-dione (**6m**)

Orange solid, 56% yield (0.107 g). ^1^H NMR (400 MHz, DMSO): δ 9.36 (t, *J* = 6.0 Hz, 1H), 8.56 (d, *J* = 4.5 Hz, 1H), 7.94 (d, *J* = 8.7 Hz, 2H), 7.82 (t, *J* = 7.7 Hz, 1H), 7.43 (d, *J* = 7.7 Hz, 1H), 7.35–7.30 (m, 1H), 7.03 (d, *J* = 8.7 Hz, 2H), 5.01 (d, *J* = 6.0 Hz, 2H), 4.11–4.02 (m, 2H), 3.42 (dd, *J* = 12.4, 2.5 Hz, 2H), 3.09 (dd, *J* = 12.4, 6.5 Hz, 2H), 1.19 (d, *J* = 6.3 Hz, 6H). ^13^C NMR (101 MHz, DMSO): δ 192.07, 189.49, 178.40, 163.51, 157.93, 152.84, 149.72, 137.53, 128.58, 123.14, 122.03, 119.01, 113.94, 66.03, 51.83, 49.24, 18.29. HRMS (ES+) *m*/*z*: calcd for C_22_H_24_N_3_O_3_ [M + H]^+^: 378.1812; found, 378.1811 (Diff: 0.26 ppm). mp 208–210 °C. Purity HPLC 97.69%, *R*_*t*_ = 7.92 min.

#### 3-(4-(4-Fluoropiperidin-1-yl)­phenyl)-4-((pyridin-2-ylmethyl)­amino)­cyclobut-3-ene-1,2-dione (**6n**)

Dark yellow solid of **6n** in a 99% yield (0.178 g). ^1^H NMR (400 MHz, DMSO): δ 9.37 (t, *J* = 6.2 Hz, 1H), 8.57–8.54 (m, 1H), 7.94 (d, *J* = 9.0 Hz, 2H), 7.82 (td, *J* = 7.8, 1.8 Hz, 1H), 7.43 (d, *J* = 7.8 Hz, 1H), 7.35–7.30 (m, 1H), 7.09 (d, *J* = 9.0 Hz, 2H), 5.01 (d, *J* = 6.2 Hz, 2H), 4.98–4.92 (m, 0.5H), 4.88–4.79 (m, 0.5H), 3.68–3.50 (m,21H), 3.40–3.35 (m, 2H), 2.05–1.89 (m, 2H), 1.83–1.68 (m, 2H). ^13^C NMR (101 MHz, DMSO): δ 192.15, 189.48, 178.45, 163.47, 157.93, 152.04, 149.72, 137.52, 128.64, 123.13, 122.01, 119.08, 114.68, 88.91 (d, *J* = 169.5 Hz), 49.24, 44.01 (d, *J* = 6.7 Hz), 30.80 (d, *J* = 19.1 Hz). HRMS (ES+) *m*/*z*: calcd for C_21_H_21_FN_3_O_2_ [M + H]^+^: 366.1612; found, 366.1623 (Diff: −3.00 ppm). mp 197–198 °C. Anal. Calcd for C_21_H_20_FN_3_O_2_: C, 69.03; H, 5.52; N, 11.50. Found C, 69.29; H, 5.70; N, 11.37.

#### 3-(4-(4,4-Difluoropiperidin-1-yl)­phenyl)-4-((pyridin-2-ylmethyl)­amino)­cyclobut-3-ene-1,2-dione (**6o**)

Orange solid, 83% yield (0.12 g). ^1^H NMR (400 MHz, DMSO): δ 9.40 (t, *J* = 5.5 Hz, 1H), 8.56 (d, *J* = 4.4 Hz, 1H), 7.96 (d, *J* = 8.8 Hz, 2H), 7.82 (td, *J* = 7.7, 1.2 Hz, 1H), 7.43 (d, *J* = 7.7 Hz, 1H), 7.37–7.29 (m, 1H), 7.14 (d, *J* = 8.8 Hz, 2H), 5.01 (d, *J* = 5.5 Hz, 2H), 3.58–3.51 (m, 4H), 2.11–1.97 (m, 4H). ^13^C NMR (101 MHz, DMSO): δ 192.30, 189.47, 178.55, 163.27, 157.87, 151.29, 149.72, 137.53, 128.61, 123.14, 122.03, 119.68, 115.10, 49.24, 44.74 (t, *J* = 4.8 Hz), 33.18 (t, *J* = 22.5 Hz). HRMS (ES+) *m*/*z*: calcd for C_21_H_20_F_2_N_3_O_2_ [M + H]^+^: 384.1519; found, 384.1521 (Diff: −0.78 ppm). IR ν_max_/cm^–1^: (solid) 3257 (m), 2927 (m), 2849 (s), 1769 (s), 1708 (s), 1618 (s), 1583 (s), 1345 (s), 1231 (s). mp 203–205 °C. Purity HPLC 95.21%, *R*_*t*_ = 8.63 min.

#### 3-(4-(4-Methoxypiperidin-1-yl)­phenyl)-4-((pyridin-2-ylmethyl)­amino)­cyclobut-3-ene-1,2-dione (**6p**)

Dark yellow solid, 93% yield (0.097 g). ^1^H NMR (400 MHz, DMSO): δ 9.35 (s, 1H), 8.57–8.54 (m, 1H), 7.93 (d, *J* = 9.0 Hz, 2H), 7.81 (td, *J* = 7.8, 1.7 Hz, 1H), 7.43 (d, *J* = 7.8 Hz, 1H), 7.37–7.25 (m, 1H), 7.06 (d, *J* = 9.0 Hz, 2H), 5.01 (d, *J* = 3.8 Hz, 2H), 3.77–3.61 (m, 2H), 3.46–3.38 (m, 1H), 3.33 (s, 3H), 3.19–2.96 (m, 2H), 1.97–1.87 (m, 2H), 1.56–1.42 (m, 2H). ^13^C NMR (101 MHz, DMSO): δ 192.06, 189.49, 178.39, 163.57, 157.95, 152.32, 149.71, 137.51, 128.64, 123.12, 122.00, 118.79, 114.53, 75.61, 55.39, 49.24, 45.02, 30.27. HRMS (ES+) *m*/*z*: calcd for C_22_H_24_N_3_O_3_ [M + H]^+^: 378.1812; found, 378.1826 (Diff: −3.70 ppm). IR ν_max_/cm^–1^: (solid) 3068 (m), 2919 (s), 1762 (m), 1704 (s), 11,586 (s), 1566 (s), 1210 (s), 1197 (s). mp 195–197 °C. Anal. Calcd for C_22_H_23_N_3_O_3_: C, 70.01; H, 6.14; N, 11.13. Found C, 70.25; H, 6.40; N, 10.99.

#### 3-(4-((1*S*,4*S*)-2-Oxa-5-azabicyclo­[2.2.1]­heptan-5-yl)­phenyl)-4-((pyridin-2-ylmethyl)­amino)­cyclobut-3-ene-1,2-dione (**6q**)

Orange solid, 97% yield (0.06 g). ^1^H NMR (400 MHz, DMSO): δ 9.29 (t, *J* = 5.7 Hz, 1H), 8.57–8.54 (m, 1H), 7.92 (d, *J* = 8.8 Hz, 2H), 7.81 (td, *J* = 7.8, 1.8 Hz, 1H), 7.43 (d, *J* = 7.8 Hz, 1H), 7.35–7.30 (m, 1H), 6.76 (d, *J* = 8.8 Hz, 2H), 5.01 (d, *J* = 5.7 Hz, 2H), 4.72 (d, *J* = 34.0 Hz, 2H), 3.79 (d, *J* = 6.6 Hz, 1H), 3.64 (d, *J* = 7.4 Hz, 1H), 3.56–3.52 (m, 1H), 3.11 (d, *J* = 9.5 Hz, 1H), 1.92 (dd, *J* = 24.2, 9.5 Hz, 2H). ^13^C NMR (101 MHz, DMSO): δ 191.62, 189.49, 178.12, 163.96, 158.01, 149.70, 149.29, 137.51, 128.80, 123.10, 121.98, 117.68, 112.92, 75.97, 72.48, 58.04, 57.37, 49.22, 36.78. mp 208–210 °C. Purity HPLC 98.07%, *R*_*t*_ = 6.77 min. [α]_D_ = −213 (*c* = 0.3, MeOH).

#### 3-(4-((1*R*,4*R*)-2-Oxa-5-azabicyclo­[2.2.1]­heptan-5-yl)­phenyl)-4-((pyridin-2-ylmethyl) amino)­cyclobut-3-ene-1,2-dione (**6r**)

Orange solid, 82% yield (0.11 g). ^1^H NMR (400 MHz, DMSO): δ 9.29 (t, *J* = 6.0 Hz, 1H), 8.56 (d, *J* = 4.8 Hz, 1H), 7.93 (d, *J* = 8.7 Hz, 2H), 7.82 (td, *J* = 7.7, 1.5 Hz, 1H), 7.43 (d, *J* = 7.7 Hz, 1H), 7.32 (dd, *J* = 7.0, 5.2 Hz, 1H), 6.76 (d, *J* = 8.7 Hz, 2H), 5.01 (d, *J* = 6.0 Hz, 2H), 3.79 (d, *J* = 7.2 Hz, 1H), 3.65 (d, *J* = 7.2 Hz, 1H), 3.54 (d, *J* = 9.4 Hz, 1H), 3.12 (d, *J* = 9.8 Hz, 1H), 1.99–1.84 (m, 2H). ^13^C NMR (101 MHz, DMSO): δ 191.63, 189.49, 178.12, 163.96, 158.01, 149.70, 149.29, 137.51, 128.80, 123.10, 121.98, 117.69, 112.92, 75.97, 72.48, 58.04, 57.37, 49.22, 36.78. HRMS (ES+) *m*/*z*: calcd for C_21_H_20_N_3_O_3_ [M + H]^+^: 362.1499; found, 362.1506 (Diff: −1.9 ppm). mp 213–215 °C. Anal. Calcd for C_21_H_19_N_3_O_3_: C, 69.79; H, 5.30; N, 11.63. Found C, 69.07; H, 5.30; N, 11.60. Purity HPLC 95.93%, *R*_*t*_ = 6.76 min. [α]_D_ = +232 (*c* = 0.5, MeOH).

#### 3-(4-(8-Oxa-3-azabicyclo­[3.2.1]­octan-3-yl)­phenyl)-4-((pyridin-2-ylmethyl)­amino)­cyclobut-3-ene-1,2-dione (**6s**)

Orange solid in a 61% yield (0.05 g). ^1^H NMR (400 MHz, DMSO): δ 9.36 (t, *J* = 5.0 Hz, 1H), 8.56 (d, *J* = 4.3 Hz, 1H), 7.94 (d, *J* = 8.8 Hz, 2H), 7.82 (td, *J* = 7.7, 1.2 Hz, 1H), 7.43 (d, *J* = 7.7 Hz, 1H), 7.35–7.30 (m, 1H), 6.97 (d, *J* = 8.8 Hz, 2H), 5.01 (d, *J* = 5.0 Hz, 2H), 4.46 (s, 2H), 3.62–3.55 (m, 2H), 2.97–2.89 (m, 2H), 1.89–1.76 (m, 4H). ^13^C NMR (101 MHz, DMSO): δ 192.07, 189.49, 178.43, 163.55, 157.91, 153.21, 149.72, 137.53, 128.39, 123.14, 122.02, 119.15, 113.37, 73.24, 52.34, 49.24, 31.15. HRMS (ES+) *m*/*z*: calcd for C_22_H_22_N_3_O_3_ [M + H]^+^: 376.1656; found, 376.1659 (Diff: −0.79 ppm). mp 229–231 °C. Purity HPLC 95.08%, *R*_*t*_ = 7.49 min.

### 3-Isopropoxy-4-(4-morpholinophenyl)­cyclobut-3-ene-1,2-dione (**12**)

A solution of 4-(4-bromophenyl)­morpholine **11** (0.3 g, 1.24 mmol, 1 equiv) in anhydrous THF (12 mL) was cooled to −78 °C, then *t*-BuLi in pentane (1.7 M, 1.46 mL, 2.48 mmol, 2 equiv) was added slowly. The resulting yellow solution was allowed to warm to room temperature slowly and stir for another 2.5 h. The solution was recooled down to −78 °C. A suspension of diisopropyl squarate (0.49 g, 2.48 mmol, 2 equiv) in anhydrous THF (16 mL) was added slowly. The resulting yellow mixture was allowed to warm to room temperature slowly and allowed to stir overnight (18h). The reaction mixture was cooled in an ice bath, and water (approx. 15 mL) was added to quench the reaction. The mixture was allowed to stir for another 15 min. Concentrated hydrochloric acid (approx. 2 mL) was added and the mixture was allowed to stir for an hour (followed by TLC). Then it was neutralized with saturated sodium bicarbonate solution and extracted with ethyl acetate (3 × 20 mL), the combined organic layers were washed with brine, dried over MgSO_4_, filtered, and concentrated to a yellow oil. The crude product was purified by column chromatography using 5–40% ethyl acetate in hexane to afford product **12** as a yellow solid in a 21–37% yield (0.13–0.137 g). ^1^H NMR (400 MHz, CDCl_3_): δ 7.98 (d, *J* = 8.6 Hz, 2H), 6.92 (d, *J* = 8.6 Hz, 2H), 5.65–5.53 (m, 1H), 3.92–3.79 (m, 4H), 3.39–3.28 (m, 4H), 1.54 (d, *J* = 6.2 Hz, 6H). ^13^C NMR (101 MHz, CDCl_3_): δ 193.45, 192.07, 190.99, 173.76, 153.76, 129.72, 118.43, 113.82, 79.27, 66.49, 47.26, 23.05. HRMS (ES+) *m*/*z*: calcd for C_17_H_19_NO_4_Na [M + Na]^+^: 324.1206; found, 324.1209 (Diff: −0.92 ppm).

### General Procedure B for the Synthesis of Analogues **13a–g**

A mixture of 3-isopropoxy-4-(4-morpholinophenyl)­cyclobut-3-ene-1,2-dione (**12**) (1 equiv), methanamine salts (**16a**–**g**) (1 equiv), and triethyl amine (1–2 equiv) in anhydrous MeOH (3 mL) was allowed to stir for 3 h. The product was filtered to isolate products **13a**–**g** with/without further purification being required (see details below).

#### 3-(((6-Fluoropyridin-2-yl)­methyl)­amino)-4-(4-morpholinophenyl)­cyclobut-3-ene-1,2-dione (**13a**)

A mixture of 3-isopropoxy-4-(4-morpholinophenyl)­cyclobut-3-ene-1,2-dione (**12**) (1 equiv), (6-fluoropyridin-2-yl) methanamine salt (**16a**) (1 equiv), and triethyl amine (2 equiv) in anhydrous MeOH (3 mL) was allowed to stir for 3 h. The product was filtered to give **13a** as a light-yellow solid in a 42% yield (0.01 g) and no further purification was required. ^1^H NMR (400 MHz, DMSO): δ 9.42 (t, *J* = 6.1 Hz, 1H), 8.02 (dd, *J* = 16.0, 8.2 Hz, 1H), 7.96 (d, *J* = 8.9 Hz, 2H), 7.41 (dd, *J* = 7.4, 2.1 Hz, 1H), 7.12 (dd, *J* = 8.2, 2.1 Hz, 1H), 7.08 (d, *J* = 8.9 Hz, 2H), 4.96 (d, *J* = 6.1 Hz, 2H), 3.80–3.70 (m, 4H), 3.33–3.27 (m, 4H). ^13^C NMR (101 MHz, DMSO): δ 189.48, 178.48, 162.45 (d, *J* = 231.2 Hz), 160.33, 157.13 (d, *J* = 13.1 Hz), 152.84, 144.24, 143.51 (d, *J* = 7.7 Hz), 128.50, 125.39, 119.89 (d, *J* = 3.7 Hz), 119.66, 114.36, 108.74 (d, *J* = 37.0 Hz), 66.31, 47.36, 46.15. HRMS (ES+) *m*/*z*: calcd for C_20_H_18_FN_3_O_3_Na [M + Na]^+^: 390.1224; found, 390.1227 (Diff: −0.76 ppm). IR ν_max_/cm^–1^: (solid) 3019 (s), 2923 (m), 1786 (m), 1705 (s), 1589 (s), 1506 (s), 1220 (s), 1202 (s). mp >280 °C. Purity HPLC 99.43%, *R*_*t*_ = 8.54 min.

#### 3-(((5-Fluoropyridin-2-yl)­methyl)­amino)-4-(4-morpholinophenyl)­cyclobut-3-ene-1,2-dione (**13b**)

A mixture of 3-isopropoxy-4-(4-morpholinophenyl)­cyclobut-3-ene-1,2-dione (**12**) (1 equiv), (5-fluoropyridin-2-yl)­methanamine salt (**16b**) (1 equiv), and triethyl amine (2 equiv). The product was filtered to give **13b** as a yellow solid in a 79% yield (0.024 g) and no further purification was required. ^1^H NMR (400 MHz, DMSO): δ 9.39 (t, *J* = 5.4 Hz, 1H), 8.56 (d, *J* = 2.8 Hz, 1H), 7.95 (d, *J* = 8.8 Hz, 2H), 7.76 (td, *J* = 8.6, 2.8 Hz, 1H), 7.54 (dd, *J* = 8.6, 4.5 Hz, 1H), 7.07 (d, *J* = 8.8 Hz, 2H), 5.01 (d, *J* = 5.4 Hz, 2H), 3.78–3.70 (m, 4H), 3.31–3.29 (m, 4H). ^13^C NMR (101 MHz, DMSO): δ 191.91, 189.46, 178.48, 175.25, 158.89 (d, *J* = 252.9 Hz), 152.80 (d, *J* = 2.7 Hz), 137.68 (d, *J* = 23.9 Hz), 128.44, 124.39 (d, *J* = 18.3 Hz), 123.62 (d, *J* = 4.4 Hz), 119.74, 114.35, 111.59, 66.31, 47.37. HRMS (ES+) *m*/*z*: calcd for C_20_H_18_FN_3_O_3_Na [M + Na]^+^: 390.1224; found, 390.1223 (Diff: 0.26 ppm). mp 219–221 °C. Purity HPLC 96.29%, *R*_*t*_ = 8.26 min.

#### 3-(((4-Fluoropyridin-2-yl)­methyl)­amino)-4-(4-morpholinophenyl)­cyclobut-3-ene-1,2-dione (**13c**)

A mixture of 3-isopropoxy-4-(4-morpholinophenyl)­cyclobut-3-ene-1,2-dione (**12**) (1 equiv), (4-fluoropyridin-2-yl)­methanamine salt (**16c**) (1 equiv), and triethyl amine (2 equiv). The crude product was purified by flash column chromatography on silica gel using 3% MeOH/DCM as an eluent system to afford **13c** as a yellow solid in a 35% yield (0.008 g). ^1^H NMR (400 MHz, DMSO): δ 9.36 (t, *J* = 6.2 Hz, 1H), 8.60 (dd, *J* = 8.8, 5.7 Hz, 1H), 7.96 (d, *J* = 9.0 Hz, 2H), 7.41 (dd, *J* = 10.1, 2.4 Hz, 1H), 7.31–7.25 (m, 1H), 7.08 (d, *J* = 9.0 Hz, 2H), 5.03 (d, *J* = 6.2 Hz, 2H), 3.84–3.61 (m, 4H), 3.32–3.27 (m, 4H). ^13^C NMR (101 MHz, DMSO): δ 192.20, 189.53, 178.61, 169.02 (d, *J* = 263.0 Hz), 163.49, 161.66 (d, *J* = 6.4 Hz), 152.80, 152.63 (d, *J* = 7.0 Hz), 128.48, 119.76, 114.35, 110.98 (d, *J* = 16.5 Hz), 109.81 (d, *J* = 17.4 Hz), 66.31, 48.97, 47.37. HRMS (ES+) *m*/*z*: calcd for C_20_H_19_FN_3_O_3_ [M + H]^+^: 368.1405; found 368.1405 (Diff: −0.09 ppm). mp 232–235 °C. Purity HPLC 95.08%, *R*_*t*_ = 7.99 min.

#### 3-(((3-Fluoropyridin-2-yl)­methyl)­amino)-4-(4-morpholinophenyl)­cyclobut-3-ene-1,2-dione (**13d**)

A mixture of 3-isopropoxy-4-(4-morpholinophenyl)­cyclobut-3-ene-1,2-dione (**12**) (1 equiv), (3-fluoropyridin-2-yl)­methanamine salt **16d** (1 equiv), and triethyl amine (2 equiv). The reaction mixture was allowed to stir for 4 h, and the product was filtered to give **13d** as a yellow solid in a 44% yield (0.020 g) and no further purification was required. ^1^H NMR (400 MHz, DMSO): δ 9.31 (s, 1H), 8.42 (d, *J* = 4.0 Hz, 1H), 7.94 (d, *J* = 8.6 Hz, 2H), 7.84–7.73 (m, 1H), 7.49–7.43 (m, 1H), 7.07 (d, *J* = 8.6 Hz, 2H), 5.11 (s, 2H), 3.77–3.72 (m, 4H), 3.31–3.30 (m, 4H). Due to insufficient signal intensity, a ^13^C NMR spectrum could not be obtained; however, the structure is supported by ^1^H NMR and high-resolution mass spectrometry data. HRMS (ES+) *m*/*z*: calcd for C_20_H_18_FN_3_O_3_Na [M + Na]^+^: 390.1224; found, 390.1229 (Diff: −1.28 ppm). mp 222–224 °C. Anal. Calcd for C_20_H_18_FN_3_O_3_(+0.25H_2_O): C, 64.60; H, 5.01; N, 11.30. Found C, 64.48; H, 5.18; N, 11.60.

#### 3-(((6-Methoxypyridin-2-yl)­methyl)­amino)-4-(4-morpholinophenyl) Cyclobut-3-ene-1,2-dione (**13e**)

A mixture of 3-isopropoxy-4-(4-morpholinophenyl)­cyclobut-3-ene-1,2-dione (**12**) (1 equiv), (6-methoxypyridin-2-yl)­methanamine salt (**16e**) (1 equiv), and triethyl amine (2 equiv) in anhydrous MeOH (3 mL). The crude product was purified by flash column chromatography on silica gel using 3% MeOH/DCM as an eluent system to afford **13e** as a yellow solid in a 30% yield (0.017 g). ^1^H NMR (400 MHz, DMSO): δ 9.31 (t, *J* = 6.2 Hz, 1H), 7.89–7.80 (m, 1H), 7.08 (d, *J* = 8.6 Hz, 2H), 7.00 (d, *J* = 7.4 Hz, 1H), 6.83 (d, *J* = 7.4 Hz, 1H), 6.66 (d, *J* = 8.6 Hz, 2H), 4.91 (d, *J* = 6.2 Hz, 2H), 3.83 (s, 3H), 3.75–3.72 (m, 4H), 3.31–3.27 (m, 4H). ^13^C NMR (101 MHz, DMSO): δ 192.24, 189.61, 179.05, 163.66, 157.84, 155.70, 140.33, 139.95, 128.38, 119.77, 114.39, 113.35, 108.77, 66.32, 53.93, 53.32, 47.39. HRMS (ES+) *m*/*z*: calcd for C_21_H_22_N_3_O_4_ [M + H]^+^: 380.1605; found, 380.1604 (Diff: 0.26 ppm). Purity HPLC 95.45%, *R*_*t*_ = 8.94 min.

#### 3-(4-Morpholinophenyl)-4-((pyridin-4-ylmethyl)­amino)­cyclobut-3-ene-1,2-dione (**13f**)

A mixture of 3-isopropoxy-4-(4-morpholinophenyl)­cyclobut-3-ene-1,2-dione (**12**) (1 equiv) and pyridin-4-ylmethanamine (1 equiv). The product was filtered to provide **13f** as a yellow solid in a 70% yield (0.016 g) with no further purification required. ^1^H NMR (400 MHz, DMSO): δ 9.38 (s, 1H), 8.59–8.52 (m, 2H), 7.95 (d, *J* = 9.0 Hz, 2H), 7.39 (d, *J* = 5.9 Hz, 2H), 7.08 (d, *J* = 9.0 Hz, 2H), 4.93 (s, 2H), 3.80–3.69 (m, 4H), 3.32–3.22 (m, 4H). ^13^C NMR (101 MHz, DMSO): δ 192.13, 189.40, 178.16, 163.79, 152.89, 150.33, 147.82, 128.51, 122.70, 119.64, 114.36, 66.31, 47.35, 46.66. HRMS (ES+) *m*/*z*: calcd for C_20_H_20_N_3_O_3_ [M + H]^+^: 350.1499; found, 350.1500 (Diff: −0.18 ppm). mp 255–257 °C. Anal. Calcd for C_20_H_19_N_3_O_3_: C, 68.75; H, 5.48; N, 12.03. Found C, 68.43; H, 5.36; N, 11.80.

#### 3-(4-Morpholinophenyl)-4-((pyrimidin-4-ylmethyl)­amino)­cyclobut-3-ene-1,2-dione (**13g**)

A mixture of 3-isopropoxy-4-(4-morpholinophenyl)­cyclobut-3-ene-1,2-dione (**12**) (1 equiv) and pyrimidin-4-ylmethanamine (1 equiv). The reaction mixture was allowed to stir for 18 h. The product was purified using flash column chromatography on silica gel using 5% MeOH/DCM as an eluent system afford **13g** as a light-yellow solid in a 60% yield (0.014 g). ^1^H NMR (400 MHz, DMSO): δ 9.38 (s, 1H), 9.16 (d, *J* = 1.1 Hz, 1H), 8.80 (d, *J* = 5.2 Hz, 1H), 7.95 (d, *J* = 9.0 Hz, 2H), 7.82 (d, *J* = 9.0 Hz, 2H), 7.60 (dd, *J* = 5.2, 1.1 Hz, 1H), 5.01 (s, 2H), 3.78–3.64 (m, 4H), 3.31–3.27 (m, 4H). ^13^C NMR (101 MHz, DMSO): δ 189.57, 187.73, 158.84, 158.13, 129.21, 128.52, 125.85, 120.72, 119.65, 117.53, 114.38, 114.26, 66.31, 47.35, 47.00. HRMS (ES+) *m*/*z*: calcd for C_19_H_18_N_4_O_3_Na [M + Na]^+^: 373.1271; found, 373.1277 (Diff: −1.60 ppm). mp 253–255 °C. Purity HPLC 95.48%, *R*_*t*_ = 7.04 min.

### General Procedure C for Synthesis of Analogues **14a–k**

3-Chloro-4-(4-morpholinophenyl)­cyclobut-3-ene-1,2-dione **4a** (1.0 equiv) was dissolved in 1,4-dioxane (28 mL/mmol) and cooled to 0 °C. Triethylamine (TEA) (1.5 equiv) was added dropwise followed by the addition of pyridin-3-ylmethanamine (1.5 equiv). The solution was then warmed to room temperature and allowed to stir for 1–3 h. The reaction mixture was concentrated in vacuo and extracted with DCM. The products were purified using flash column chromatography on silica gel to afford **14a**–**k**.

#### 3-(4-Morpholinophenyl)-4-((pyridin-3-ylmethyl)­amino)­cyclobut-3-ene-1,2-dione (**14a**)

Prepared according to general procedure C using **4a** (1 equiv), pyridin-3-ylmethanamine (1.5 equiv), and TEA (1.5 equiv). The product was purified using flash column chromatography on silica gel using 3–5% MeOH/DCM as an eluent system to afford **14a** as yellow solid in a 43% yield (0.07 g). ^1^H NMR (400 MHz, DMSO): δ 9.35 (t, *J* = 6.1 Hz, 1H), 8.62 (d, *J* = 1.7 Hz, 1H), 8.53 (dd, *J* = 4.8, 1.4 Hz, 1H), 7.92 (d, *J* = 9.0 Hz, 2H), 7.81 (dt, *J* = 7.8, 1.7 Hz, 1H), 7.42 (dd, *J* = 7.8, 4.8 Hz, 1H), 7.07 (d, *J* = 9.0 Hz, 2H), 4.94 (d, *J* = 6.1 Hz, 2H), 3.79–3.65 (m, 4H), 3.31–3.21 (m, 4H). ^13^C NMR (101 MHz, DMSO): δ 192.25, 189.30, 177.84, 163.67, 152.84, 149.50, 149.26, 136.00, 134.46, 128.45, 124.24, 119.66, 114.33, 66.31, 47.34, 45.47. HRMS (ES+) *m*/*z*: calcd for C_20_H_20_N_3_O_3_ [M + H]^+^: 350.1499; found, 350.1503 (Diff: −1.14 ppm). mp >270 °C. Purity HPLC 95.28%, *R*_*t*_ = 6.05 min.

#### 3-(4-Morpholinophenyl)-4-((pyrimidin-2-ylmethyl)­amino)­cyclobut-3-ene-1,2-dione (**14b**)

Prepared according to general procedure C using **4a** (1 equiv), pyrimidin-2-ylmethanamine hydrochloride salt (1.5 equiv), and TEA (2.5 equiv). The product was purified using flash column chromatography on silica gel using 5% MeOH/DCM as an eluent system to afford **14b** as a dark orange solid in a 50% yield (0.05 g). ^1^H NMR (400 MHz, DMSO): δ 9.39 (t, *J* = 6.2 Hz, 1H), 8.82 (d, *J* = 4.9 Hz, 2H), 7.96 (d, *J* = 9.0 Hz, 2H), 7.46 (t, *J* = 4.9 Hz, 1H), 7.09 (d, *J* = 9.0 Hz, 2H), 5.09 (d, *J* = 6.2 Hz, 2H), 3.78–3.72 (m, 4H), 3.32–3.27 (m, 4H). ^13^C NMR (101 MHz, DMSO): δ 192.28, 188.65, 183.44, 179.14, 166.64, 158.12, 152.81, 128.41, 120.66, 119.74, 114.41, 112.50, 66.32, 49.60, 47.36. HRMS (ES+) *m*/*z*: calcd for C_19_H_18_N_4_O_3_Na [M + Na]^+^: 373.1271; found, 373.1274 (Diff: −0.80 ppm). mp 270–272 °C. Purity HPLC 95.62%, *R*_*t*_ = 7.13 min.

#### 3-(4-Morpholinophenyl)-4-((thiazol-2-ylmethyl)­amino)­cyclobut-3-ene-1,2-dione (**14c**)

Prepared according to general procedure C using **4a** (1 equiv), thiazol-2-ylmethanamine (1.5 equiv), and TEA (1.5 equiv). The product **14c** was isolated as a yellow solid in a 53% yield (0.048 g). ^1^H NMR (400 MHz, DMSO): δ 9.62 (t, *J* = 6.1 Hz, 1H), 7.95 (d, *J* = 9.0 Hz, 2H), 7.82 (d, *J* = 3.2 Hz, 1H), 7.73 (d, *J* = 3.2 Hz, 1H), 7.08 (d, *J* = 9.0 Hz, 2H), 5.21 (d, *J* = 6.2 Hz, 2H), 3.77–3.73 (m, 4H), 3.32–3.29 (m, 4H); ^13^C NMR (101 MHz, DMSO): δ 191.86, 189.49, 178.17, 167.87, 164.07, 152.96, 143.26, 128.57, 121.39, 119.38, 114.35, 66.30, 47.30, 45.32. HRMS (ES+) *m*/*z*: calcd for C_18_H_18_N_3_O_3_S [M + H]+: 356.1063; found, 356.1072. IR ν_max_/cm^–1^: (solid) 3255 (s), 1763 (s), 1574 (s) and 1237 (s). mp 238–240 °C. Anal. Calcd for C_18_H_17_N_3_O_3_S (+0.75 H_2_O) C, 58.60; H, 5.05; N, 11.39; S, 9.02. Found: C, 58.35; H, 4.68; N, 11.00; S, 8.76.

#### 3-(((6-Methylpyridin-2-yl)­methyl)­amino)-4-(4-morpholinophenyl)­cyclobut-3-ene-1,2-dione (**14d**)

Prepared according to general procedure C using **4a** (1 equiv), (6-methylpyridin-2-yl)­methanamine (1.5 equiv), and TEA (1.5 equiv). The product **14d** was isolated as a yellow solid in a 68% yield (0.17g). ^1^H NMR (400 MHz, DMSO): δ 9.41 (s, 1H), 7.97 (d, *J* = 8.8 Hz, 2H), 7.69 (t, *J* = 7.7 Hz, 1H), 7.20 (dd, *J* = 13.5, 7.7 Hz, 2H), 7.07 (d, *J* = 8.9 Hz, 2H), 4.95 (s, 2H), 3.83–3.66 (m, 4H), 3.31–3.25 (m, 4H), 2.55–2.49 (m, 3H). ^13^C NMR (101 MHz, DMSO): δ 192.24, 189.45, 178.39, 163.41, 158.09, 157.30, 152.80, 137.80, 128.46, 122.42, 119.78, 118.89, 114.37, 66.32, 49.39, 47.39, 24.47. HRMS (ES+) *m*/*z*: calcd for C_21_H_22_N_3_O_3_ [M + H]+: 364.1656; found, 364.1657. IR ν_max_/cm^–1^: (solid) 3222 (s), 1768 (s), 1567 (s) and 1237 (s). mp 215–217 °C. Anal. Calcd for C_21_H_21_N_3_O_3_ C, 69.41; H, 5.82; N, 11.56. Found: C, 69.32; H, 6.08; N, 11.33.

#### 3-(((5-Methylpyridin-2-yl)­methyl)­amino)-4-(4-morpholinophenyl)­cyclobut-3-ene-1,2-dione (**14e**)

Prepared according to general procedure C using **4a** (1 equiv), (5-methylpyridin-2-yl)­methanamine (1.5 equiv), and TEA (1.5 equiv). The product **14e** was isolated as a brown solid in a 49% yield (0.054 g). ^1^H NMR (400 MHz, DMSO): δ 9.38 (t, *J* = 6.1 Hz, 1H), 8.39 (s, 1H), 7.96 (d, *J* = 8.9 Hz, 2H), 7.62 (dd, *J* = 8.0, 1.7 Hz, 1H), 7.32 (d, *J* = 7.9 Hz, 1H), 7.07 (d, *J* = 9.0 Hz, 2H), 4.96 (d, *J* = 6.2 Hz, 2H), 3.80–3.68 (m, 4H), 3.31–3.24 (m, 4H), 2.29 (s, 3H); ^13^C NMR (101 MHz, DMSO): δ 192.26, 189.45, 178.46, 163.30, 155.01, 152.77, 149.91, 137.79, 132.25, 128.42, 121.55, 119.80, 114.36, 66.32, 49.02, 47.38, 18.04; HRMS (ES+) *m*/*z*: calcd for C_21_H_22_N_3_O_3_ [M + H]+: 364.1656; found, 364.1664. IR ν_max_/cm^–1^: (solid) 3152 (s), 1766 (s), 1562 (s) and 1242 (s); mp 196–199 °C. Anal. Calcd for C_21_H_21_N_3_O_3_ C, 69.41; H, 5.82; N, 11.56. Found: C, 69.37; H, 6.08; N, 11.38.

#### 3-(((4-Methylpyridin-2-yl)­methyl)­amino)-4-(4-morpholinophenyl)­cyclobut-3-ene-1,2-dione (**14f**)

Prepared according to general procedure C using **4a** (1 equiv), (4-methylpyridin-2-yl)­methanamine (1.5 equiv), and TEA (1.5 equiv). The crude product was purified by flash column chromatography on silica gel using 2% MeOH in DCM as an eluent system to afford **14f** as a yellow solid in a 17% yield (0.031 g). ^1^H NMR (400 MHz, DMSO): δ 9.42 (t, *J* = 6.1 Hz, 1H), 8.45 (d, *J* = 5.0 Hz, 1H), 8.01 (d, *J* = 8.8 Hz, 2H), 7.32 (s, 1H), 7.21 (d, *J* = 4.8 Hz, 1H), 7.13 (d, *J* = 8.9 Hz, 2H), 5.02 (d, *J* = 6.1 Hz, 2H), 3.84–3.77 (m, 4H), 3.37–3.29 (m, 4H), 2.38 (s, 3H); ^13^C NMR (101 MHz, DMSO): δ 192.28, 189.47, 178.47, 163.28, 157.67, 152.78, 149.44, 148.24, 128.44, 123.96, 122.64, 119.83, 114.37, 66.32, 49.22, 47.39, 20.96. HRMS (ES+) *m*/*z*: calcd for C21H_22_N_3_O_3_ [M + H]+: 364.1656; found, 364.1656. IR ν_max_/cm^–1^: (solid) 3156 (s), 1771 (s), 1577 (s) and 1238 (s). mp 227–230 °C. Anal. Calcd for C_21_H_21_N_3_O_3_ (+0.5 H_2_O) C, 67.73; H, 5.59; N, 11.28. Found: C, 67.53; H, 5.89; N, 10.78.

#### 3-(((3-Methylpyridin-2-yl)­methyl)­amino)-4-(4-morpholinophenyl)­cyclobut-3-ene-1,2-dione (**14g**)

Prepared according to general procedure C using **4a** (1 equiv), (3-methylpyridin-2-yl)­methanamine (1.5 equiv), and TEA (1.5 equiv). The product **14g** was isolated as a yellow solid in a 56% yield (0.053 g). ^1^H NMR (400 MHz, DMSO): δ 9.24 (t, *J* = 5.9 Hz, 1H), 8.37 (d, *J* = 4.3 Hz, 1H), 7.97 (d, *J* = 8.8 Hz, 2H), 7.62 (d, *J* = 7.5 Hz, 1H), 7.24 (dd, *J* = 7.5, 4.8 Hz, 1H), 7.08 (d, *J* = 8.9 Hz, 2H), 5.02 (d, *J* = 6.0 Hz, 2H), 3.78–3.73 (m, 4H), 3.31–3.26 (m, 4H), 2.35 (s, 3H); ^13^C NMR (101 MHz, DMSO): δ 192.45, 189.43, 178.88, 163.06, 155.25, 152.74, 146.86, 138.37, 131.02, 128.40, 123.03, 119.91, 114.39, 66.33, 47.41, 46.74, 17.88. HRMS (ES+) *m*/*z*: calcd for C21H_22_N_3_O_3_ [M + H]+: 364.1656; found, 364.1661. IR ν_max_/cm^–1^: (solid) 3162 (s), 1768 (s), 1580 (s) and 1240 (s). mp 217–220 °C. Anal. Calcd for C_21_H_21_N_3_O_3_ (+0.5 H_2_O) C, 67.73; H, 5.59; N, 11.28. Found: C, 67.80; H, 6.00; N, 11.32.

#### 3-(((6-Chloropyridin-2-yl)­methyl)­amino)-4-(4-morpholinophenyl)­cyclobut-3-ene-1,2-dione (**14h**)

Prepared according to general procedure C using **4a** (1 equiv), (6-chloropyridin-2-yl)­methanamine salt **16f** (1.5 equiv), and TEA (2.5 equiv). The product was purified by flash column chromatography on silica gel using 2–3% MeOH/DCM as an eluent system to afford **14h** as an orange solid in a 45% yield (0.05 g). ^1^H NMR (400 MHz, DMSO): δ 9.42 (t, *J* = 6.2 Hz, 1H), 7.95 (d, *J* = 9.0 Hz, 2H), 7.89 (t, *J* = 7.8 Hz, 1H), 7.46 (dd, *J* = 7.8, 2.3 Hz, 2H), 7.08 (d, *J* = 9.0 Hz, 2H), 4.97 (d, *J* = 6.2 Hz, 2H), 3.79–3.70 (m, 4H), 3.32–3.27 (m, 4H). ^13^C NMR (101 MHz, DMSO): δ 192.10, 189.51, 178.44, 163.66, 159.19, 152.87, 150.28, 141.23, 128.52, 123.64, 121.28, 119.63, 114.37, 66.31, 48.67, 47.35. HRMS (ES+) *m*/*z*: calcd for C_20_H_18_^35^ClN_3_O_3_Na [M + Na]^+^: 406.0929; found, 406.0928 (Diff: 0.25 ppm). mp 258–260 °C. Purity HPLC 95.16%, *R*_*t*_ = 8.84 min.

#### 3-(((6-Chloro-3-methylpyridin-2-yl)­methyl)­amino)-4-(4-morpholinophenyl)­cyclobut-3-ene-1,2-dione (**14i**)

Prepared according to general procedure C using **4a** (1 equiv), (6-chloro-3-methylpyridin-2-yl)­methanamine salt **16g** (1.5 equiv), and TEA (2.5 equiv). The crude product was washed with water, filtered, air-dried, and then purified by flash column chromatography on silica gel using 1% MeOH/DCM as an eluent system to afford **14i** as a brown solid in a 45% yield (0.05 g). ^1^H NMR (400 MHz, DMSO): δ 9.27 (t, *J* = 5.1 Hz, 1H), 7.97 (d, *J* = 8.7 Hz, 2H), 7.71 (d, *J* = 8.0 Hz, 1H), 7.36 (d, *J* = 8.0 Hz, 1H), 7.08 (d, *J* = 8.7 Hz, 2H), 4.99 (d, *J* = 5.1 Hz, 2H), 3.80–3.71 (m, 4H), 3.32–3.26 (m, 4H), 2.34 (s, 3H). ^13^C NMR (101 MHz, DMSO): δ 192.30, 189.44, 178.72, 163.32, 156.29, 152.82, 147.42, 142.17, 130.80, 128.50, 123.36, 119.74, 114.39, 66.32, 47.38, 46.47, 17.13. HRMS (ES+) *m*/*z*: calcd for C_21_H_20_^35^ClN_3_O_3_Na [M + Na]^+^: 420.1085; found, 420.1079 (Diff: 1.43 ppm). Purity HPLC 96.18%, *R*_*t*_ = 9.50 min.

#### 3-(4-Morpholinophenyl)-4-((2-(pyrrolidin-1-yl)­ethyl)­amino)­cyclobut-3-ene-1,2-dione (**14j**)

Prepared according to general procedure C using **4a** (1 equiv), (2-(pyrrolidin-1-yl)­ethan-1-amine) (1.5 equiv), and TEA (1.5 equiv). The crude product was washed with water, filtered, and dried to afford a dark yellow solid of **14j** in a 62% yield (0.23 g) with no further purification required. ^1^H NMR (400 MHz, DMSO): δ 8.88 (s, 1H), 7.92 (d, *J* = 9.0 Hz, 2H), 7.06 (d, *J* = 9.0 Hz, 2H), 3.79 (t, *J* = 6.3 Hz, 2H), 3.77–3.71 (m, 4H), 3.38–3.22 (m, 4H), 2.68 (t, *J* = 6.3 Hz, 2H), 2.52–2.49 (m, 4H), 1.70–1.64 (m, 4H). ^13^C NMR (101 MHz, DMSO): δ 192.23, 189.17, 178.04, 162.96, 152.70, 128.25, 119.88, 114.37, 66.32, 56.59, 54.09, 47.40, 43.74, 23.62. HRMS (ES+) *m*/*z*: calcd for C_20_H_26_N_3_O_3_ [M + H]^+^: 356.1969; found, 356.1973 (Diff: −1.12 ppm). IR ν_max_/cm^–1^: (solid) 3005 (s), 2923 (m), 2867 (s), 1769 (m), 1707 (s), 1588 (s), 1505 (s), 1280 (s), 1220 (s). mp 190–192 °C. Purity HPLC 95.54%, *R*_*t*_ = 5.70 min.

#### 3-(Methyl­(pyridin-2-ylmethyl)­amino)-4-(4-morpholinophenyl)­cyclobut-3-ene-1,2-dione (**14k**)

Prepared according to general procedure C using **4a** (1 equiv), (*N*-methyl-1-(pyridin-2-yl)­methanamine) (1.5 equiv), and TEA (1.5 equiv). The crude product was washed using a DCM/H_2_O mixture. The product was purified using flash column chromatography on silica gel using 5% MeOH/DCM as an eluent system to afford **13f** as brown crystals in 53% yield (0.08 g). ^1^H NMR (400 MHz, DMSO): δ 8.59 (s, 1H), 7.84 (td, *J* = 7.7, 1.6 Hz, 1H), 7.61 (d, *J* = 7.7 Hz, 1H), 7.52 (d, *J* = 6.4 Hz, 1H), 7.46 (d, *J* = 7.0 Hz, 1H), 7.40–7.32 (m, 1H), 7.06 (d, *J* = 8.0 Hz, 1H), 6.94 (d, *J* = 6.4 Hz, 1H), 5.11 (s, 1H), 4.79 (s, 1H), 3.84–3.62 (m, 4H), 3.28–3.18 (m, 4H), 2.51–2.49 (m, 3H). ^13^C NMR (101 MHz, DMSO): δ 192.00, 185.28, 179.90, 164.34, 147.19, 137.76, 136.07, 136.00, 129.64, 126.66, 122.25, 118.81, 114.40, 66.35, 47.56, 31.16, 15.63. Due to low signal intensity, the ^13^C NMR spectrum was weak; nonetheless, the observed chemical shifts were consistent with the proposed structure. HRMS (ES+) *m*/*z*: calcd for C_21_H_21_N_3_O_3_Na [M + Na]^+^: 386.1475; found, 386.1484 (Diff: −2.33 ppm). mp 190–193 °C. Anal. Calcd for C_21_H_21_N_3_O_3_ (+H_2_O): C, 66.13; H, 6.08; N, 11.02. Found C, 65.94; H, 5.63; N, 10.74.

### General Procedure D for the Synthesis of Analogues **20a–l**

**4g–h, 4j–o, 4q–r** (1.0 equiv) were dissolved in 1,4-dioxane (28 mL/mmol) and cooled to 0 °C. Triethylamine (1.5–2.5 equiv) was added dropwise followed by the addition of amine/amine salts **19a** (1.5 equiv). The solution was then warmed to room temperature and allowed to stir for 3 h. The reaction mixture was concentrated under reduced pressure. The product was purified by flash column chromatography on silica gel to afford **20a**–**l**.

#### (*S*)-3-(((6-Fluoropyridin-2-yl)­methyl)­amino)-4-(4-(3-methylmorpholino)­phenyl)­cyclobut-3-ene-1,2-dione (**20a**)

Prepared following general procedure D using (*S*)-3-chloro-4-(4-(3-methylmorpholino)­phenyl) cyclobut-3-ene-1,2-dione **4g** (1.0 equiv), (6-fluoropyridin-2-yl)­methanamine salt **19a** (1.5 equiv), and TEA (2.5 equiv). The crude product was purified by flash column chromatography on silica gel using 5% MeOH/DCM as an eluent system to afford **20a** as an orange solid in a 31% yield (0.058 g). ^1^H NMR (400 MHz, DMSO): δ 9.37 (s, 1H), 8.02 (dd, *J* = 15.9, 8.1 Hz, 1H), 7.95 (d, *J* = 9.0 Hz, 2H), 7.41 (dd, *J* = 7.4, 2.3 Hz, 1H), 7.12 (dd, *J* = 8.1, 2.3 Hz, 1H), 7.02 (d, *J* = 9.0 Hz, 2H), 4.96 (s, 2H), 4.15–4.06 (m, 1H), 3.96 (dd, *J* = 11.3, 3.6 Hz, 1H), 3.77–3.72 (m, 1H), 3.69 (dd, *J* = 11.3, 2.8 Hz, 1H), 3.56 (dd, *J* = 12.1, 2.8 Hz, 1H), 3.53–3.45 (m, 1H), 3.08 (td, *J* = 12.1, 3.6 Hz, 1H), 1.09 (d, *J* = 6.6 Hz, 3H). ^13^C NMR (101 MHz, DMSO): δ 191.97, 189.49, 178.38, 163.77, 163.03 (d, *J* = 233.3 Hz), 157.19 (d, *J* = 14.9 Hz), 151.62, 143.50 (d, *J* = 7.7 Hz), 128.69, 119.87 (d, *J* = 3.8 Hz), 118.91, 113.89, 108.73 (d, *J* = 36.8 Hz), 71.00, 66.51, 48.67, 48.38, 41.32, 12.11. HRMS (ES+) *m*/*z*: calcd for C_21_H_21_FN_3_O_3_ [M + H]^+^: 382.1561; found, 382.1567 (Diff: −1.57 ppm). mp 204–206 °C. Purity HPLC 98.13%, *R*_*t*_ = 8.82 min.

#### (*R*)-3-(((6-Fluoropyridin-2-yl)­methyl)­amino)-4-(4-(3-methylmorpholino)­phenyl)­cyclobut-3-ene-1,2-dione (**20b**)

Prepared according to general procedure D using (*R*)-3-chloro-4-(4-(3-methylmorpholino)­phenyl)­cyclobut-3-ene-1,2-dione **4h** (1.0 equiv), (6-fluoropyridin-2-yl)­methanamine salt **19a** (1.5 equiv), and TEA (2.5 equiv). The crude product was purified by flash column chromatography on silica gel using 3% MeOH/DCM as an eluent system to afford **20b** as an orange solid in a 53% yield (0.09 g). ^1^H NMR (400 MHz, DMSO): δ 9.37 (t, *J* = 6.0 Hz, 1H), 8.02 (dd, *J* = 15.9, 8.2 Hz, 1H), 7.95 (d, *J* = 9.0 Hz, 2H), 7.41 (dd, *J* = 7.4, 2.3 Hz, 1H), 7.12 (dd, *J* = 8.2, 2.3 Hz, 1H), 7.02 (d, *J* = 9.0 Hz, 2H), 4.96 (d, *J* = 6.0 Hz, 2H), 4.15–4.06 (m, 1H), 3.96 (dd, *J* = 11.3, 3.6 Hz, 1H), 3.74 (d, *J* = 11.3 Hz, 1H), 3.69 (dd, *J* = 11.3, 2.8 Hz, 1H), 3.56 (dd, *J* = 12.1, 2.8 Hz, 1H), 3.53–3.49 (m, 1H), 3.08 (td, *J* = 12.1, 3.6 Hz, 1H), 1.09 (d, *J* = 6.6 Hz, 3H). ^13^C NMR (101 MHz, DMSO): δ 191.98, 189.51, 178.40, 166.03 (d, *J* = 224.3 Hz), 164.29 (d, *J* = 13.3 Hz), 163.78, 151.64, 143.52 (d, *J* = 7.7 Hz), 128.70, 119.89 (d, *J* = 3.9 Hz), 118.92, 113.91, 108.74 (d, *J* = 36.9 Hz), 71.01, 66.51, 48.67, 41.32, 12.11. HRMS (ES+) *m*/*z*: calcd for C_21_H_21_FN_3_O_3_ [M + H]^+^: 382.1561; found, 382.1564 (Diff: −0.78 ppm). mp 205–207 °C. Purity HPLC 95.44%, *R*_*t*_ = 8.83 min.

#### (*S*)-3-(4-(3-Methylmorpholino)­phenyl)-4-((thiazol-2-ylmethyl)­amino)­cyclobut-3-ene-1,2-dione (**20c**)

Prepared according to general procedure D using (*S*)-3-chloro-4-(4-(3-methylmorpholino)­phenyl)­cyclobut-3-ene-1,2-dione **4g** (1.0 equiv), thiazol-2-ylmethanamine, (1.5 equiv), and TEA (1.5 equiv). The crude product was purified by flash column chromatography on silica gel using 5% MeOH/DCM as an eluent system to afford **20c** as a dark red solid in a 49% yield (0.198 g). ^1^H NMR (400 MHz, DMSO): δ 9.59 (s, 1H), 7.94 (d, *J* = 8.9 Hz, 2H), 7.81 (d, *J* = 3.2 Hz, 1H), 7.73 (d, *J* = 3.2 Hz, 1H), 7.02 (d, *J* = 8.9 Hz, 2H), 5.21 (s, 2H), 4.15–4.06 (m, 1H), 3.96 (dd, *J* = 11.1, 3.6 Hz, 1H), 3.74 (d, *J* = 11.1 Hz, 1H), 3.69 (dd, *J* = 11.1, 2.9 Hz, 1H), 3.56 (dd, *J* = 11.9, 2.9 Hz, 1H), 3.52–3.46 (m, 1H), 3.08 (td, *J* = 11.9, 3.6 Hz, 1H), 1.09 (d, *J* = 6.6 Hz, 3H). ^13^C NMR (101 MHz, DMSO): δ 191.70, 189.49, 178.04, 167.92, 164.23, 151.74, 143.25, 128.76, 121.37, 118.63, 113.87, 70.99, 66.50, 48.64, 45.32, 41.29, 12.16. HRMS (ES+) *m*/*z*: calcd for C_19_H_20_N_3_O_3_S [M + H]^+^: 370.1220; found, 370.1224 (Diff: −1.08 ppm). IR ν_max_/cm^–1^: (solid) 3253 (m), 2969 (s), 2930 (s), 1768 (m), 1706 (s), 1578 (s), 1494 (s), 1323 (s), 1229 (s). mp 202–204 °C. Purity HPLC 95.68%, *R*_*t*_ = 8.17 min.

#### (*R*)-3-(4-(3-Methylmorpholino)­phenyl)-4-((thiazol-2-ylmethyl)­amino)­cyclobut-3-ene-1,2-dione (**20d**)

Prepared according to general procedure D using (*R*)-3-chloro-4-(4-(3-methylmorpholino)­phenyl)­cyclobut-3-ene-1,2-dione **4h** (1.0 equiv), thiazol-2-ylmethanamine (1.5 equiv), and TEA (1.5 equiv). The crude product was purified by flash column chromatography on silica gel using 3% MeOH/DCM as an eluent system to afford **20d** as a brown solid in a 17% yield (0.01 g). ^1^H NMR (400 MHz, DMSO): δ 9.59 (t, *J* = 6.2 Hz, 1H), 7.94 (d, *J* = 8.9 Hz, 2H), 7.82 (d, *J* = 3.2 Hz, 1H), 7.73 (d, *J* = 3.2 Hz, 1H), 7.02 (d, *J* = 8.9 Hz, 2H), 5.21 (d, *J* = 6.2 Hz, 2H), 4.15–4.06 (m, 1H), 3.96 (dd, *J* = 11.1, 3.6 Hz, 1H), 3.74 (d, *J* = 11.1 Hz, 1H), 3.69 (dd, *J* = 11.1, 2.8 Hz, 1H), 3.56 (dd, *J* = 12.0, 2.8 Hz, 1H), 3.53–3.45 (m, 1H), 3.08 (td, *J* = 12.0, 3.6 Hz, 1H), 1.09 (d, *J* = 6.6 Hz, 3H). ^13^C NMR (101 MHz, DMSO): δ 189.51, 181.53, 178.05, 174.83, 167.94, 164.24, 143.26, 128.76, 121.39, 118.64, 113.88, 100.03, 70.99, 66.51, 48.65, 45.33, 41.29, 12.17. HRMS (ES+) *m*/*z*: calcd for C_19_H_20_N_3_O_3_S [M + H]^+^: 370.1220; found, 370.1220 (Diff: 0.01 ppm).

#### 3-(4-(4-Fluoropiperidin-1-yl)­phenyl)-4-(((6-fluoropyridin-2-yl)­methyl)­amino)­cyclobut-3-ene-1,2-dione (**20e**)

Prepared according to general procedure D using 3-chloro-4-(4-(4-fluoropiperidin-1-yl)­phenyl)­cyclobut-3-ene-1,2-dione **4n** (1.0 equiv), (6-fluoropyridin-2-yl)­methanamine salt **19a** (1.5 equiv), and TEA (2.5 equiv). The crude product was purified by flash column chromatography on silica gel using 3% MeOH/DCM as an eluent system to afford **20e** as a yellow solid in a 35% yield (0.035 g). ^1^H NMR (400 MHz, DMSO): δ 9.38 (s, 1H), 8.01 (dd, *J* = 15.8, 8.2 Hz, 1H), 7.94 (d, *J* = 9.0 Hz, 2H), 7.40 (dd, *J* = 7.4, 2.4 Hz, 1H), 7.14–7.08 (m, 3H), 4.96 (s, 2H), 4.87–4.80 (m, 1H), 3.62–3.53 (m, 2H), 3.43–3.37 (m, 2H), 2.05–1.88 (m, 2H), 1.83–1.69 (m, 2H). ^13^C NMR (101 MHz, DMSO): δ 189.49, 182.95, 178.38, 163.03 (d, *J* = 238.1 Hz), 161.46, 157.33 (d, *J* = 15.8 Hz), 152.08, 143.51 (d, *J* = 7.9 Hz), 128.70, 128.22, 119.60 (d, *J* = 3.8 Hz), 114.66, 108.72 (d, *J* = 36.8 Hz), 88.91 (d, *J* = 169.8 Hz), 54.45, 48.37, 30.79 (d, *J* = 19.4 Hz). HRMS (ES+) *m*/*z*: calcd for C_21_H_20_F_2_N_3_O_2_ [M + H]^+^: 384.1518; found, 384.1512 (Diff: 1.56 ppm). mp >300 °C. Purity HPLC 95.16%, *R*_*t*_ = 9.45 min.

#### (*S*)-3-(((6-Fluoropyridin-2-yl)­methyl)­amino)-4-(4-(3-methylmorpholino)­phenyl)­cyclobut-3-ene-1,2-dione (**20f**)

Prepared according to general procedure D using 3-chloro-4-(4-(4,4-difluoropiperidin-1-yl)­phenyl)­cyclobut-3-ene-1,2-dione **4o** (1.0 equiv), (6-fluoropyridin-2-yl)­methanamine salt **19a** (1.5 equiv), and TEA (2.5 equiv). The crude product was purified by flash column chromatography on silica gel using 3% MeOH/DCM as an eluent system and then using 2% MeOH/CHCl_3_. The product was then collected by recrystallization from CHCl_3_ to provide **20f** as a yellow solid in a 9% yield (0.04 g). ^1^H NMR (400 MHz, DMSO): δ 9.41 (t, *J* = 5.5 Hz, 1H), 8.02 (dd, *J* = 15.9, 8.1 Hz, 1H), 7.96 (d, *J* = 8.8 Hz, 2H), 7.43–7.38 (m, 1H), 7.15 (d, *J* = 8.8 Hz, 2H), 7.13–7.09 (m, 1H), 4.96 (d, *J* = 5.5 Hz, 2H), 3.59–3.51 (m, 4H), 2.11–1.97 (m, 4H). ^13^C NMR (101 MHz, DMSO): δ 192.16, 189.47, 178.49, 163.51, 163.03 (d, *J* = 237.4 Hz), 157.13 (d, *J* = 12.8 Hz), 151.34, 143.50 (d, *J* = 8.0 Hz), 128.67, 123.19, 119.88 (d, *J* = 3.8 Hz), 115.08, 108.74 (d, *J* = 36.7 Hz), 48.38, 44.72 (t, *J* = 4.9 Hz), 33.19 (t, *J* = 22.7 Hz). HRMS (ES+) *m*/*z*: calcd for C_21_H_18_F_3_N_3_O_2_Na [M + Na]^+^: 424.1243; found, 424.1242 (Diff: 0.24 ppm). mp 240–242 °C. Anal. Calcd for C_21_H_18_F_3_N_3_O_2_: C, 62.84; H, 4.52; N, 10.47. Found C, 62.41; H, 4.43; N, 10.41. Purity HPLC 96.29%, *R*_*t*_ = 11.08 min.

#### 3-(4-((1*S*,4*S*)-2-Oxa-5-azabicyclo­[2.2.1]­heptan-5-yl)­phenyl)-4-(((6-fluoropyridin-2-yl)­methyl)­amino)­cyclobut-3-ene-1,2-dione (**20g**)

Prepared according to general procedure D using 3-(4-((1*S*,4*S*)-2-oxa-5-azabicyclo­[2.2.1]­heptan-5-yl)­phenyl)-4-chloro cyclobut-3-ene-1,2-dione **4q** (1.0 equiv), (6-fluoropyridin-2-yl)­methanamine salt **19a** (1.5 equiv), and TEA (2.5 equiv). The product was purified by flash column chromatography on silica gel using 2% MeOH/CHCl_3_ as an eluent system. The crude product was then collected by recrystallization from DCM to provide **20g** as a yellow solid in a 26% yield (0.06 g). ^1^H NMR (400 MHz, DMSO): δ 9.31 (s, 1H), 8.02 (q, *J* = 8.0 Hz, 1H), 7.92 (d, *J* = 8.5 Hz, 2H), 7.40 (d, *J* = 7.0 Hz, 1H), 7.12 (d, *J* = 8.2 Hz, 1H), 6.77 (d, *J* = 8.5 Hz, 2H), 4.96 (s, 2H), 4.78 (s, 1H), 4.69 (s, 1H), 3.79 (d, *J* = 7.3 Hz, 1H), 3.65 (d, *J* = 7.4 Hz, 1H), 3.54 (d, *J* = 9.7 Hz, 1H), 3.12 (d, *J* = 9.9 Hz, 1H), 1.96 (d, *J* = 9.6 Hz, 1H), 1.90 (d, *J* = 9.8 Hz, 1H). ^13^C NMR (101 MHz, DMSO): δ 191.51, 189.51, 178.06, 163.03 (d, *J* = 236.7 Hz), 157.29 (d, *J* = 12.8 Hz), 149.35, 143.49 (d, *J* = 7.8 Hz), 128.86, 119.84 (d, *J* = 3.9 Hz), 117.58, 112.93, 108.70 (d, *J* = 37.0 Hz), 75.97, 72.49, 58.04, 57.38, 48.37, 36.78. HRMS (ES+) *m*/*z*: calcd for C_21_H_18_FN_3_O_3_Na [M + Na]^+^: 402.1224; found, 402.1231 (Diff: −1.74 ppm). IR ν_max_/cm^–1^: (solid) 3206 (s), 3163 (s), 2927 (m), 2854 (s), 1768 (s), 1704 (s), 1602 (s), 1558 (s), 1356 (s), 1198 (s). mp 268–270 °C. Anal. Calcd for C_21_H_18_FN_3_O_3_: C, 66.48; H, 4.78; N, 11.08. Found C, 66.28; H, 4.67; N, 10.93. Purity HPLC 98.98%, *R*_*t*_ = 8.82 min.

#### 3-(4-((1*R*,4*R*)-2-Oxa-5-azabicyclo­[2.2.1]­heptan-5-yl)­phenyl)-4-(((6-fluoropyridin-2-yl)­methyl)­amino)­cyclobut-3-ene-1,2-dione (**20h**)

Prepared according to general procedure D using 3-(4-((1*R*,4*R*)-2-oxa-5-azabicyclo­[2.2.1]­heptan-5-yl)­phenyl)-4-chloro cyclobut-3-ene-1,2-dione **4r** (1.0 equiv), (6-fluoropyridin-2-yl)­methanamine salt **19a** (1.5 equiv), and TEA (2.5 equiv). The crude product was purified by flash column chromatography on silica gel using 3% MeOH/CHCl_3_ as an eluent system to afford **20h** as a bright yellow solid in a 35% yield (0.08 g). ^1^H NMR (400 MHz, DMSO): δ 9.31 (t, *J* = 6.0 Hz, 1H), 8.01 (q, *J* = 8.1 Hz, 1H), 7.92 (d, *J* = 8.6 Hz, 2H), 7.42–7.37 (m, 1H), 7.11 (dd, *J* = 8.1, 1.6 Hz, 1H), 6.77 (d, *J* = 8.6 Hz, 2H), 4.96 (d, *J* = 6.0 Hz, 2H), 4.77 (s, 1H), 4.68 (s, 1H), 3.79 (d, *J* = 7.3 Hz, 1H), 3.65 (d, *J* = 7.3 Hz, 1H), 3.54 (d, *J* = 9.6 Hz, 1H), 3.12 (d, *J* = 9.6 Hz, 1H), 1.93 (dd, *J* = 24.1, 9.6 Hz, 2H). ^13^C NMR (101 MHz, DMSO): δ 191.48, 189.54, 178.09, 163.04 (d, *J* = 236.8 Hz), 157.28 (d, *J* = 12.7 Hz), 149.37, 143.50 (d, *J* = 7.7 Hz), 128.87, 119.84 (d, *J* = 3.9 Hz), 117.57, 112.93, 108.71 (d, *J* = 36.7 Hz), 75.98, 72.50, 58.04, 57.38, 48.36, 36.78. HRMS (ES+) *m*/*z*: calcd for C_21_H_18_FN_3_O_3_Na [M + Na]^+^: 402.1224; found, 402.1226 (Diff: −0.49 ppm). mp 263–265 °C. Anal. Calcd for C_21_H_18_FN_3_O_3_(+0.25H_2_O): C, 65.70; H, 4.86; N, 10.95. Found C, 65.76; H, 4.77; N, 10.69.

#### 3-(4-(2,6-Dimethylmorpholino)­phenyl)-4-(((6-fluoropyridin-2-yl)­methyl)­amino)­cyclobut-3-ene-1,2-dione (**20i**)

Prepared according to general procedure D using 3-chloro-4-(4-(2,6-dimethylmorpholino)­phenyl)­cyclobut-3-ene-1,2-dione **4j** (1.0 equiv), (6-fluoropyridin-2-yl)­methanamine salt **19a** (1.5 equiv), and TEA (2.5 equiv). The crude product was purified by flash column chromatography on silica gel using 3% MeOH/DCM as an eluent system to afford **20i** as an orange solid in an 18% yield (0.02 g). ^1^H NMR (400 MHz, DMSO): δ 9.40 (t, *J* = 6.1 Hz, 1H), 8.01 (q, *J* = 8.2 Hz, 1H), 7.94 (d, *J* = 8.8 Hz, 2H), 7.40 (dd, *J* = 7.3, 1.5 Hz, 1H), 7.12 (dd, *J* = 8.2, 1.9 Hz, 1H), 7.08 (d, *J* = 8.8 Hz, 2H), 4.96 (d, *J* = 6.1 Hz, 2H), 3.82 (d, *J* = 11.8 Hz, 2H), 3.73–3.62 (m, 2H), 3.46–3.42 (m, 2H), 1.18 (d, *J* = 6.1 Hz, 6H). ^13^C NMR (101 MHz, DMSO): δ 192.04, 189.52, 178.46, 163.67, 163.05 (d, *J* = 236.5 Hz), 157.15 (d, *J* = 12.6 Hz), 152.43, 143.53 (d, *J* = 7.6 Hz), 128.56, 119.89 (d, *J* = 4.0 Hz), 119.37, 114.36, 108.75 (d, *J* = 36.7 Hz), 71.30, 52.48, 48.37, 19.24. HRMS (ES+) *m*/*z*: calcd for C_22_H_22_FN_3_O_3_Na [M + Na]^+^: 418.1537; found, 418.1544 (Diff: −1.67 ppm). mp 218–220 °C. Purity HPLC 95.07%, *R*_*t*_ = 9.69 min.

#### 3-(4-(*Cis*-2,6-Dimethylmorpholino)­phenyl)-4-(((6-fluoropyridin-2-yl)­methyl)­amino)­cyclobut-3-ene-1,2-dione (**20j**)

Prepared according to general procedure D using 3-chloro-4-(4-(*Cis* 2,6-dimethylmorpholino)­phenyl)­cyclobut-3-ene-1,2-dione **4k** (1.0 equiv), (6-fluoropyridin-2-yl)­methanamine salt **19a** (1.5 equiv), and TEA (2.5 equiv). The crude product was purified by flash column chromatography on silica gel using 1% MeOH/DCM as an eluent system to afford **20j** as a yellow solid in a 26% yield (0.03 g). ^1^H NMR (400 MHz, DMSO): δ 9.40 (s, 1H), 8.02 (dd, *J* = 15.9, 8.3 Hz, 1H), 7.95 (d, *J* = 8.8 Hz, 2H), 7.41 (dd, *J* = 7.2, 1.5 Hz, 1H), 7.12 (dd, *J* = 8.3, 1.9 Hz, 1H), 7.08 (d, *J* = 8.8 Hz, 2H), 4.96 (s, 2H), 3.83 (d, *J* = 11.6 Hz, 2H), 3.73–3.63 (m, 2H), 2.39 (t, *J* = 11.6 Hz, 2H), 1.18 (d, *J* = 6.2 Hz, 6H). ^13^C NMR (101 MHz, DMSO): δ 192.04, 189.49, 178.43, 163.65, 163.03 (d, *J* = 237.5 Hz), 157.16 (d, *J* = 13.1 Hz), 152.41, 143.51 (d, *J* = 7.7 Hz), 128.55, 119.89 (d, *J* = 3.6 Hz), 119.38, 114.35, 108.74 (d, *J* = 36.8 Hz), 71.29, 52.48, 48.38, 19.25. HRMS (ES+) *m*/*z*: calcd for C_22_H_22_FN_3_O_3_Na [M + Na]^+^: 418.1537; found, 418.1538 (Diff: −0.24 ppm). mp 269–271 °C. Anal. Calcd for C_22_H_22_FN_3_O_3_: C, 66.82; H, 5.61; N, 10.63. Found C, 66.37; H, 5.79; N, 9.83. Purity HPLC 99.60%, *R*_*t*_ = 9.64 min.

#### 3-(4-((*Trans*-2*S*,6*S*)-2,6-Dimethylmorpholino)­phenyl)-4-(((6-fluoropyridin-2-yl)­methyl) amino)­cyclobut-3-ene-1,2-dione (**20k**)

Prepared according to general procedure D using 3-chloro-4-(4-((*Trans* 2*S*,6*S*)-2,6-dimethylmorpholino)­phenyl)­cyclobut-3-ene-1,2-dione **4l** (1.0 equiv), (6-fluoropyridin-2-yl)­methanamine salt **19a** (1.5 equiv), and TEA (2.5 equiv). The crude product was purified by flash column chromatography on silica gel using 4% MeOH/DCM as an eluent system to afford **20k** as a yellow solid in 32% yield (0.06 g). ^1^H NMR (400 MHz, DMSO): δ 9.37 (s, 1H), 8.02 (q, *J* = 8.1 Hz, 1H), 7.94 (d, *J* = 8.8 Hz, 2H), 7.40 (dd, *J* = 7.4, 1.9 Hz, 1H), 7.12 (dd, *J* = 8.1, 1.9 Hz, 1H), 7.04 (d, *J* = 8.8 Hz, 2H), 4.96 (s, 2H), 4.11–4.02 (m, 2H), 3.43 (dd, *J* = 12.5, 2.6 Hz, 2H), 3.10 (dd, *J* = 12.5, 6.5 Hz, 2H), 1.19 (d, *J* = 6.3 Hz, 6H). ^13^C NMR (101 MHz, DMSO): δ 191.91, 189.50, 178.33, 163.75, 163.03 (d, *J* = 237.5 Hz), 152.88, 143.51 (d, *J* = 7.7 Hz), 128.63, 119.88 (d, *J* = 3.8 Hz), 118.87, 113.92, 108.73 (d, *J* = 36.6 Hz), 66.03, 51.81, 48.37, 18.29. HRMS (ES+) *m*/*z*: calcd for C_22_H_22_FN_3_O_3_Na [M + Na]^+^: 418.1537; found, 418.1539 (Diff: −0.48 ppm). mp 236–238 °C. Anal. Calcd for C_22_H_22_FN_3_O_3_: C, 66.82; H, 5.61; N, 10.63. Found C, 66.46; H, 5.45; N, 10.39. [α]_D_ = −96 (*c* = 0.5, MeOH). Purity HPLC 99.46%, *R*_*t*_ = 10.32 min.

#### 3-(4-((2*R*,6*R*)-2,6-Dimethylmorpholino)­phenyl)-4-(((6-fluoropyridin-2-yl)­methyl)­amino)­cyclobut-3-ene-1,2-dione (**20l**)

Prepared according to general procedure D using 3-chloro-4-(4-((*Trans* 2*R*,6*R*)-2,6-dimethylmorpholino)­phenyl)­cyclobut-3-ene-1,2-dione **4m** (1.0 equiv), (6-fluoropyridin-2-yl)­methanamine salt **19a** (1.5 equiv), and TEA (2.5 equiv). The crude product was purified by flash column chromatography on silica gel using 2% MeOH/DCM as an eluent system to afford **20l** as a yellow solid in 25% yield (0.05 g). ^1^H NMR (400 MHz, DMSO): δ 9.37 (s, 1H), 8.02 (q, *J* = 8.1 Hz, 1H), 7.94 (d, *J* = 8.8 Hz, 2H), 7.43–7.38 (m, 1H), 7.12 (dd, *J* = 8.1, 1.8 Hz, 1H), 7.04 (d, *J* = 8.8 Hz, 2H), 4.96 (s, 2H), 4.11–4.02 (m, 2H), 3.43 (dd, *J* = 12.4, 2.9 Hz, 2H), 3.10 (dd, *J* = 12.4, 6.4 Hz, 2H), 1.19 (d, *J* = 6.4 Hz, 6H). ^13^C NMR (101 MHz, DMSO): δ 191.90, 189.50, 178.33, 163.75, 163.03 (d, *J* = 237.3 Hz), 157.18 (d, *J* = 13.0 Hz), 152.88, 143.50 (d, *J* = 7.9 Hz), 128.63, 119.87 (d, *J* = 3.9 Hz), 118.87, 113.92, 108.73 (d, *J* = 36.9 Hz). HRMS (ES+) *m*/*z*: calcd for C_22_H_22_FN_3_O_3_Na [M + Na]^+^: 418.1537; found, 418.1537 (Diff: −0.03 ppm). mp 235–237 °C. Purity HPLC 100%, *R*_*t*_ = 9.29 min. [α]_D_ = +56 (c = 0.5, MeOH).

## Supplementary Material









## Notes

Molecular formula strings and associated biochemical data (IC_50_ values of SQAs against *M. tuberculosis*, log D (octanol, pH 7.4), Solubility (pH 7.4), and clearance) (CSV)
*M. tuberculosis* ATP Synthase F0 homology model (PDB)
Additional synthetic procedures; experimental and computational studies; in vitro permeability data for **6k**; in silico DMPK screening; in vivo PK and PD results; and ^1^H and ^13^C NMR spectra and HPLC chromatograms for lead compounds (DOCX)
*M. tuberculosis* ATP Synthase in complex with SQA in the end state (PDB)

## #

Manchester Institute of Biotechnology, Department of Chemistry, University of Manchester, United Kingdom, M1 7DN

## Abbreviations

ABT: aminobenzotriazole
ATP: adenosine triphosphate
AUC: area under the curve.
BDQ: bedaquiline
CFU: colony-forming unit
CL: clearance
Cryo-EM: cryogenic electron microscopy
DMPK: drug metabolism and pharmacokinetics
EMB: ethambutol
ETC: electron transport chain
GMQE: global mean quality estimate
HIV: human immunodeficiency virus
HPLC: high performance liquid chromatography
IC_50_: half maximal inhibitory concentration
INH: isoniazid
LHS: left-hand side
MD: molecular dynamics
MDR: multi drug resistant
MIC: minimum inhibitory concentration
MM/PBSA: molecular mechanics/poisson-boltzman surface area
Msm: *Mycobacterium smegmatis*
Mtb: *Mycobacterium tuberculosis*
NADH: nicotinamide adenine dinucleotide
NDH1: NADH dehydrogenase type-1
NDH-2: type II NADH dehydrogenase
NMR: nuclear magnetic resonance
PDB: protein data bank
PLIP: protein–ligand interaction profiler
PMF: proton-motive force
PZA: pyrazinamide
REMA: resazurin microtiter assay
RHS: right-hand side
RIF: rifampicin
RMSD: root-mean-square deviation
SAR: structure–activity relationship
SQAs: squaramides
TB: tuberculosis
WHO: World Health Organization
XDR: extensive-drug resistance
