# Supplementary material for: Second-Generation Anti-Tubercular Squaramides Targeting Complex V of the Respiratory Chain of Mycobacterium tuberculosis Displaying Enhanced Metabolic Stability
Source: J Med Chem. 2026 Apr 23;69(9):10213–39. doi: 10.1021/acs.jmedchem.5c03274 (PMC13181789; doi:10.1021/acs.jmedchem.5c03274)
Supplement: Supplementary file 3 [file jm5c03274_si_003.docx]

**Supporting Information (SI)**

**Second Generation Anti-Tubercular Squaramides targeting Complex V of the Respiratory Chain of Mycobacterium *tuberculosis* displaying Enhanced Metabolic Stability**

Nada Mosallam^a^, Paul M. O’Neill^a^, Monika Lisauskaitė^a^, Christopher M. Woodley^a^, Alison Ardrey^b^, Laura N. Jeffreys^b¥^, Ilinca Memelis^b,c^, Deepak Almeida^d^, Jin Lee^d^, Paul J. Converse^d^, Daire Cantillon^b,c^, Eric L. Nuermberger^d^, Dirk Bald^e^, Giancarlo A. Biagini^b^, W. David Hong^a^, Neil G. Berry^a^ and Gemma L. Nixon^a^*

^a^Department of Chemistry, University of Liverpool, L69 7ZD, Liverpool, UK

^b^Centre for Drugs & Diagnostics, Department of Tropical Disease Biology, Liverpool School of Tropical Medicine, Liverpool L3 5QA, UK

^c^Centre for Tuberculosis Research, Liverpool School of Tropical Medicine, Liverpool L3 5QA, UK

^d^Center for Tuberculosis Research, Department of Medicine, Johns Hopkins University School of Medicine, Baltimore, Maryland, USA, 21287

^e^Department of A-LIFE, AIMMS, Faculty of Science, Vrije Universiteit Amsterdam, De Boelelaan 1108, 1081 HZ Amsterdam, The Netherlands

**Table of contents**

| **Content** | **Page** |
| --- | --- |
| 1. **Synthesis of left-hand side (LHS) morpholines** | S2 |
| 1. **Synthesis of right-hand side (RHS) modified SQAs using the modified reported synthetic route** | S3 |
| 1. **Synthesis of RHS amines** | S3 |
| 1. **Synthesis of Combined RHS and LHS modifications** | S5 |
| 1. **Computational Studies** | S5 |
| 1. **Measured permeability data** | S12 |
| 1. ***In vivo* Pharmacokinetic data** | S12 |
| 1. ***In vivo* Pharmcodynamic data** | S15 |
| 1. **Predicted drug metabolism pharmacokinetic (DMPK) data** | S17 |
| 1. **Additional experimental** | S23 |
| **Synthesis of 3,4-dichlorocyclobut-3-ene-1,2-dione 2b** | S23 |
| **Synthesis of LHS derivatives 3b-s** | S24 |
| **Synthesis of intermediates 4a-s** | S28 |
| **Synthesis of derivatives 8-10 and 16** | S32 |
| **Synthesis of RHS derivatives 18a-g and 19a-g** | S34 |
| 1. **References** | S37 |
| 1. **NMR Spectra and HPLC chromatograms for squaramide compounds** | S40 |

1. **Synthesis of LHS morpholines**

Solvent free Buchwald-Hartwig amination was used to provide the heterocyclic phenyl derivatives **3b-s** with good yields and purity (**Scheme 1**, **Table 1**).^1^ Using bromo or iodo phenyl rings have been found to provide better yields than their chloro alternatives.

**Scheme S1.** Preparation of the new LHS modified SQA

*Reagent and conditions:* (a) Pd(OAc)_2_, RuPhos, NaO*t*Bu, 110 ℃, 18-48h.

**Table S1.** Yields for the synthesis of compounds **3b-s**

| **Compound** | **R^1^** | **R^2^** | **% Yield** | **Compound** | **R^1^** | **R^2^** | **% Yield** |  |
| --- | --- | --- | --- | --- | --- | --- | --- | --- |
| **3b** | *o*-CH_3_ |  | 78 | **3k** | H | *cis* | 81-88 |  |
| **3c** | *m*-CH_3_ |  | 35-78 | **3l** | H | *Trans 2S,6S* | 87 |  |
| **3d** | *m-*Cl |  | 57 | **3m** | H | *Trans 2R,6R* | 92 |  |
| **3e** | *m*-F |  | 59-84 | **3n** | H |  | 72 |  |
| **3f** | *m*-OCH_3_ |  | 78 | **3o** | H |  | 61 |  |
| **3g** | H |  | 45-64 | **3p** | H |  | 60 |  |
| **3h** | H |  | 79 | **3q** | H |  *(S,S)* | 31-46 |  |
| **3i** | H |  | 36 | **3r** | H |  (*R,R*) | 67-78 |  |
| **3j** | H |  | 52 (*cis*) | **3s** | H |  | 48 |  |

1. **Synthesis of RHS modified SQAs using the modified reported synthetic route**

**Scheme S2.** Preparation of new RHS targets using the modified original synthetic route

*Reagent and conditions:* (a) SOCl_2_, DMF, Toluene, 60 ℃, 6h; (b) Toluene, 80 ℃, 3h; (c) Amine, TEA, 1,4-Dioxane, 0 ℃ to rt, 1-3h

1. **Synthesis of RHS amines**

Nitrile pyridines were identified as the most promising alternative starting material to prepare amino pyridines, due to their low cost and their simpler synthetic strategies. NaBH_4_/I_2_ system was employed for nitrile reduction (**Scheme S3**).^2^ The reaction allowed a successful conversion of **15** to its corresponding amine **16** in a 34% yield with no further purification required.

**Scheme S3.** Preparation of the aminopyridine derivative **16**

*Reagent and conditions:* (a) 1. NaBH_4_, I_2_, THF, 0 ℃ for 2.5h, then 70 ℃, 3h; 2. HCl, 70 ℃, 0.5h; 3. NaOH, Diethylether, 0.25h.

However, this method could not be applied for the synthesis of the remaining aminopyridine analogues, due to the presence of a several side products and the arduous purification process. Therefore, an alternative method for the preparation of the remaining amines was utilised that successfully provided the required aminopyridines **19a-g**.

The other selected procedure employs sodium borohydride and catalytic amounts of nickel chloride for the reduction of the nitrile-substituted pyridine starting material.^3^ Although NaBH_4_ is generally not strong enough for the reduction of nitrile groups, it was found to have enhanced properties in the presence of transition metal salts.^4^ The formed primary amines required protection to prevent their reaction with the imine intermediate produced, resulting in dimerisation.^4^ Consequently, Di-*tert*-butyl dicarbonate (Boc) was added to the reaction mixture to afford the Boc-protected amines **18a-g.**

Deprotection was then performed using trifluoroacetic acid (TFA) yielding the amine salts **19a-g** (**Scheme S4**, **Table S2**).^3, 5^ All the prepared analogues gave acceptable to excellent yields (41-100 %) in both steps and required no further purification, therefore were used for target preparation directly. The amine salts required the addition of an extra equivalent of the triethylamine used in the final SQA amination, in order to generate the primary amine.

**Scheme S4.** Preparation of the RHS pyridines **19a-g**

*Reagent and conditions:* (a) 1. NiCl_2_.6H_2_O, di*tert*-butyl dicarbonate, NaBH_4_, MeOH, 0 ℃ then rt, 1h; 2. Diethylenetriamine, 0.5h (b) TFA, dry DCM, rt, 3h.

**Table S2.** Yields for the synthesis of compounds **19a-g**

| **Compound** | **R** | **% Yield 18** | **% Yield 19** |
| --- | --- | --- | --- |
| **19a** | 6-F | 41 | 100 |
| **19b** | 5-F | 99 | 99 |
| **19c** | 4-F | 54 | 100 |
| **19d** | 3-F | 100 | 98 |
| **19e** | 6-OMe | 54 | 100 |
| **19f** | 6-Cl | 99 | 99 |
| **19g** | 3-Me, 6-Cl | 71 | 100 |

1. **Synthesis of Combined RHS and LHS modifications**

**Scheme S5.** Preparation of derivatives **20a-l** with LHS and RHS modifications

*Reagent and conditions:* (a) SOCl_2_, DMF, Toluene, 60 ℃, 6h; (b) Toluene, 80 ℃, 3h; (c) TEA, 1,4-Dioxane, 0 ℃ to rt, 1-3h.

1. **Computational Studies**

## **Homology Modelling**

To use in molecular docking and molecular dynamics simulations, a homology model was prepared using SwissModel^6^ using a recently published cryo-EM structure of a squaramide **1f** in complex with *Mycobacterium smegmatis* (Msm) ATP synthase (PDB: 8G07) as the user template structure.^7^ This structure was chosen due to the close homology between Msm and *Mycobacterium tuberculosis* (Mtb), and the presence of a squaramide in 8G07 places the interface between the a- and c-subunits in an appropriate conformation for molecular docking of squaramide analogues. The percentage similarity of Msm a- and c-subunits compared to their Mtb homologues was found to be 75.5% and 90.1% respectively – calculated using Clustal Omega.^8^

The model produced by SWISS-model was evaluated by two metrics, global mean quality estimate (GMQE) and QMEANDisCo Global. GMQE gives the expected accuracy of a given model based on sequence alignment, while QMEANDisCo Global is a composite scoring function based on global and per-residue model quality and a series of distance constraints comparing template and generated models – both are numbers between 0–1 with higher values being better.^6, 9^ Using this template, the GMQE values was low at 0.31 reflecting the poor coverage of the b- and b-delta subunits in the cryo-EM structure. The QMEANDisCo score was found to be good with a score of 0.73 ± 0.05. Since the squaramides are known to bind in the a-c subunit interface we determine that our homology model is of an appropriate quality to use in modelling.

To investigate the protonation state of each of these residues, models were prepared using the mutagenesis wizard in Pymol. We considered doubly deprotonated (ddp), Asp32 protonated (Y32-H) and Glu65 protonated (E65-H) protonation states – these were investigated using molecular dynamics studies.

## **Molecular Docking**

Molecular docking simulations were carried out using the molecular docking package GOLD.^10^ The binding site was defined by alignment of the homology model with the template cryo-EM structure and extracting the co-crystallised ligand – this extracted ligand also served as a reference ligand for RMSD calculations. Squaramides were docked into this structure using distance constraints inspired by the proposed binding mode from the cryo-EM structure – between one squaramide carbonyl and Arg86 of the a-subunit, and the squaramide NH with Glu61 of the c-subunit (**Figure S1**). Poses were scored using the PLP scoring function and default settings were used otherwise.

Docking the reference compound into the ddp protonated model produced docking poses with an average RMSD of 1.293 Å compared to the reference ligand. The highest scoring pose had an RMSD of 1.292 Å. The main difference between the docked and co-crystallised ligand conformations is a rotation around the terminal pyridine linker torsion (**Figure S1, C**). Docking into Y32-H and E65-H protonated models afforded average RMSD values of 1.249 and 1.321 Å, respectively, and top scoring RMSD values of 1.273 and 1.321 Å, respectively. Considering the lower resolution of the cryo-EM structure (2.80 Å), these values were considered acceptable and highest scoring poses were used in molecular dynamics simulations.

## **Molecular Dynamics**

The produced homology models were embedded into pre-equilibrated POPC bilayer containing 512 lipid molecules^11^ and pore waters were generated using the CHARMM-GUI webserver.^12, 13^ The protein chains were parameterised using the pdb2gmx module in GROMACS version 2022.0 using the Amber99sb forcefield with Slipids force-field parameters for POPC lipids.^11, 14^ Ligands were parameterised using Acpype as implemented in AmberTools22 using the GAFF force-field and AM1-BCC charges.^14^ After concatenation of ligand and bilayer-system topologies, the system was solvated with TIP3P water molecules in a cubic-box with dimensions 13.1x13.1x12.5 nm and neutralised by addition of Na^+^ and Cl^-^ ions at a concentration of 0.15 M. The system was energy minimised over 50000 steps using steepest descent minimisation. The complex was warmed to 310 K by sequential NVT ensemble with positional restraints on protein, ligand and lipid bilayer for 100 ps, followed by NVT and NPT ensembles of 100ps each with positional restraints on the protein and ligand heavy atoms. We used the leap-frog algorithm for integrating Newton’s equations of motion in simulations in the NVT and NPT ensembles. Temperature and pressure were set to 310K and 1 bar controlled by the V-rescale modified Berendsen thermostat and the Parrinello-Rahman barostat, respectively.^15, 16^ We used the LINCS algorithm to constrain bonds to hydrogen, the Verlet cutoff-scheme for non-bonded interactions and the Particle Mesh Ewald (PME) scheme for electrostatic interactions. PME settings for FFT grid spacing was 0.16 nm and the interpolation order was 4.

Production MD runs of 150 ns were carried out using a timestep of 2 fs saving frames at every 10 ps – sufficient to observe convergence of bilayer, protein and ligand by RMSD metrics. Single runs were used to choose an appropriate protonation state, and for the chosen E39-H protonation state, runs were performed in triplicate. All MD simulations were undertaken on Nvidia V100 GPU nodes on Barkla, part of the High-Performance Computing facilities at the University of Liverpool.

Trajectories were clustered using the MDtraj package to calculate the distance matrix between frames, and Scipy using agglomerative clustering with a distance cut-off of 2 Å.^17, 18^ Central structures of each cluster were analysed for binding interactions using PLIP.^19^ Binding interactions identified in the top-5 most populated clusters were measured over the course of the simulation – excluding the first 25 ns to allow for equilibration – to identify stable binding interactions. For analysis of hydrogen-bonding interactions we used the Baker-Hubbard definition of donor-H-acceptor distance of < 2.5 Å and Donor-H-acceptor angle of > 120°.^20^ Otherwise, we used the same interaction definitions as default in PLIP.

Binding free energy was calculated using the Molecular Mechanics/ Poisson-Boltzman Surface Area (MM/PBSA) approach.^21^ Simply, ligand binding energy (ΔG_binding_) can be denoted as the difference in free energy between the *a* protein-ligand complex (ΔG_Complex_), and the sum of its components; protein and ligand free energy (ΔG_protein_, ΔG_ligand_, respectively).

$$\Delta G_{binding}=\Delta G_{complex}-\Delta G_{protein}-\Delta G_{Ligand}$$

The MM/PBSA approach approximates binding free energy as the change in gas-phase interaction energy (ΔE_MM,_ sum of bonded, Van der Waal’s (VdW) and electrostatic contributions) and the free energy change of solvation upon ligand binding (ΔG_solv_), which in turn can be approximated from polar and apolar contributions to solvation free energy (ΔG_polar_ and ΔG_apolar_ respectively). In line with similar studies, we neglect the entropic correction to ΔG_binding_ as it has been shown to have limited effect on the accuracy of free energy calculations.^22^

$$\Delta G_{binding}= \Delta G_{MM}-\Delta G_{solv}$$

$$\Delta G_{solv}=\Delta G_{polar}+\Delta G_{apolar}$$

MM/PBSA binding energy was calculated using the g_MMPBSA package.^23^ Calculations were carried out for every 50 frames of the final 20 ns of the trajectory. We used the Poisson-Boltzman equation to calculate the polar component of solvation free energy and the solvent-accessible surface area method to calculate the apolar component. Calculation of binding free energy and per-residue decomposition of binding energies was achieved using the MmPbSaStat.py and MmPbSaDecomp.py scripts provided with g_MMPBSA.^23^


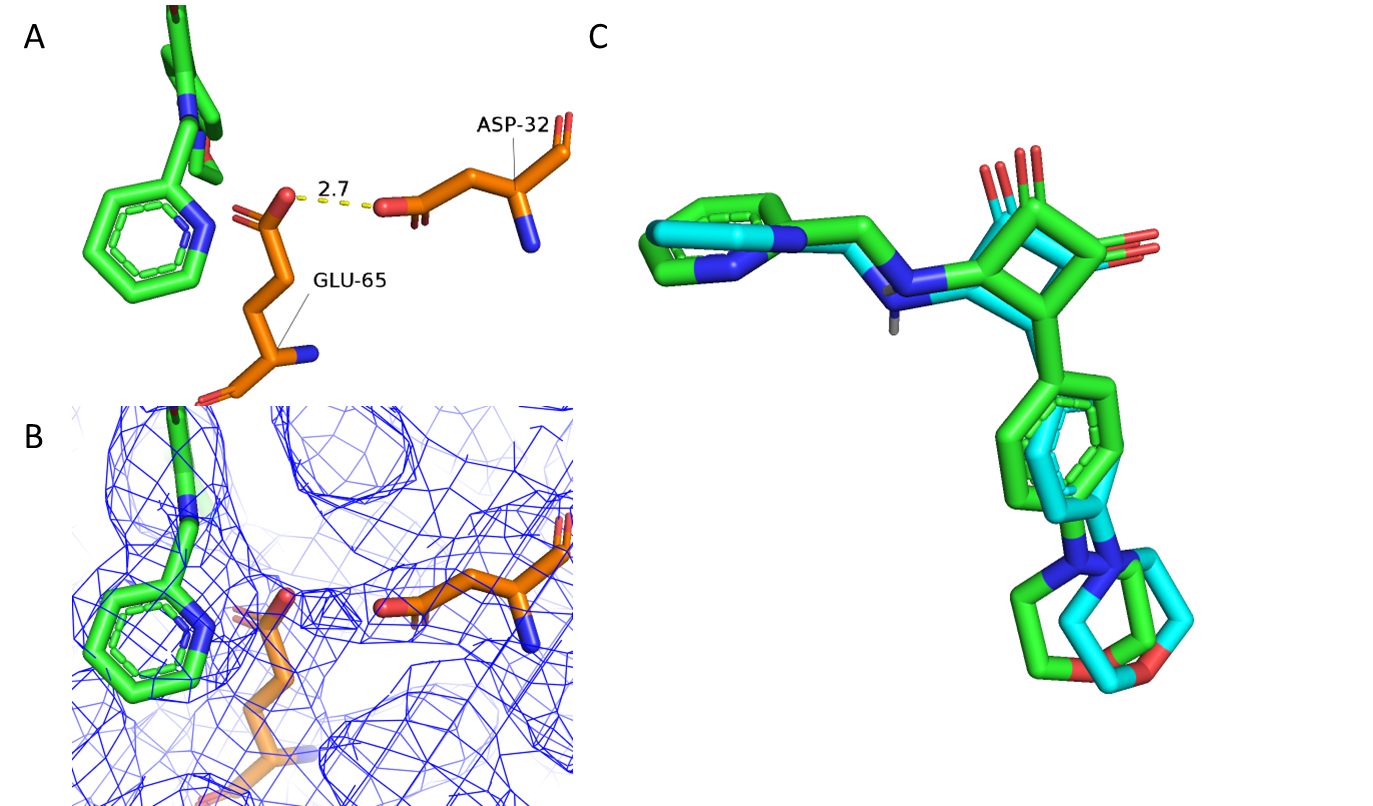


**Figure S1.** A) Pymol representation of c-subunit Asp32 and Glu65 residues of *Msm* ATP synthase (PDB: 8G07, orange sticks) and proximity to the complexed squaramide (green sticks). Distance between the carboxylate groups is indicated by a yellow-dashed line. B) Cryo-EM density isosurface of *Msm* ATP synthase (PDB: 8G07) rendered in Pymol at contour level 5. C) Superposition of the docked pose of **1f** into the *Mtb* ATP synthase homology model with the aligned, extracted reference ligand


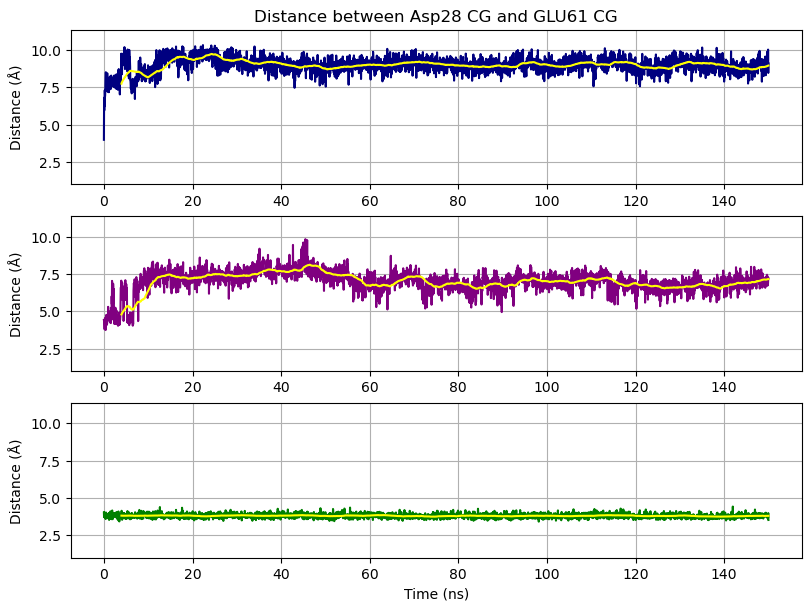


**Figure S2.** Plot of distance between the carboxylate groups of Asp28 and Glu61 of the c-subunit of *Mtb* ATP synthase over the course of each simulation with different protonation states – ddp (top), D28H (middle) and E61H (bottom), the moving average is shown as a yellow line.


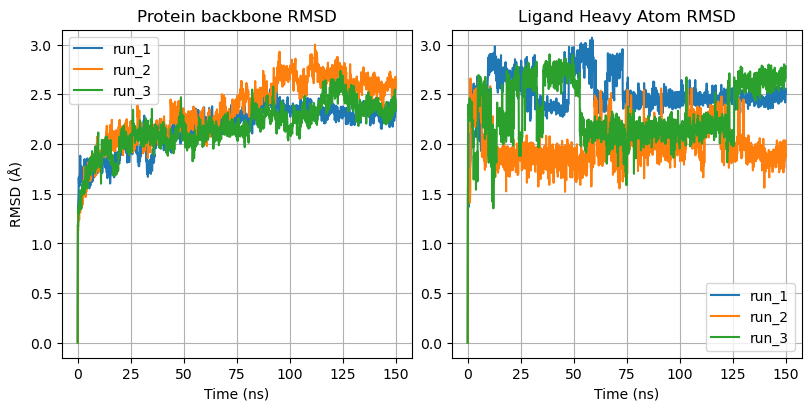
**Figure S3**. Plots of RMSD of the protein (left) and ligand (right) heavy atoms in each of the three simulations compared to the conformation at time 0 ns.


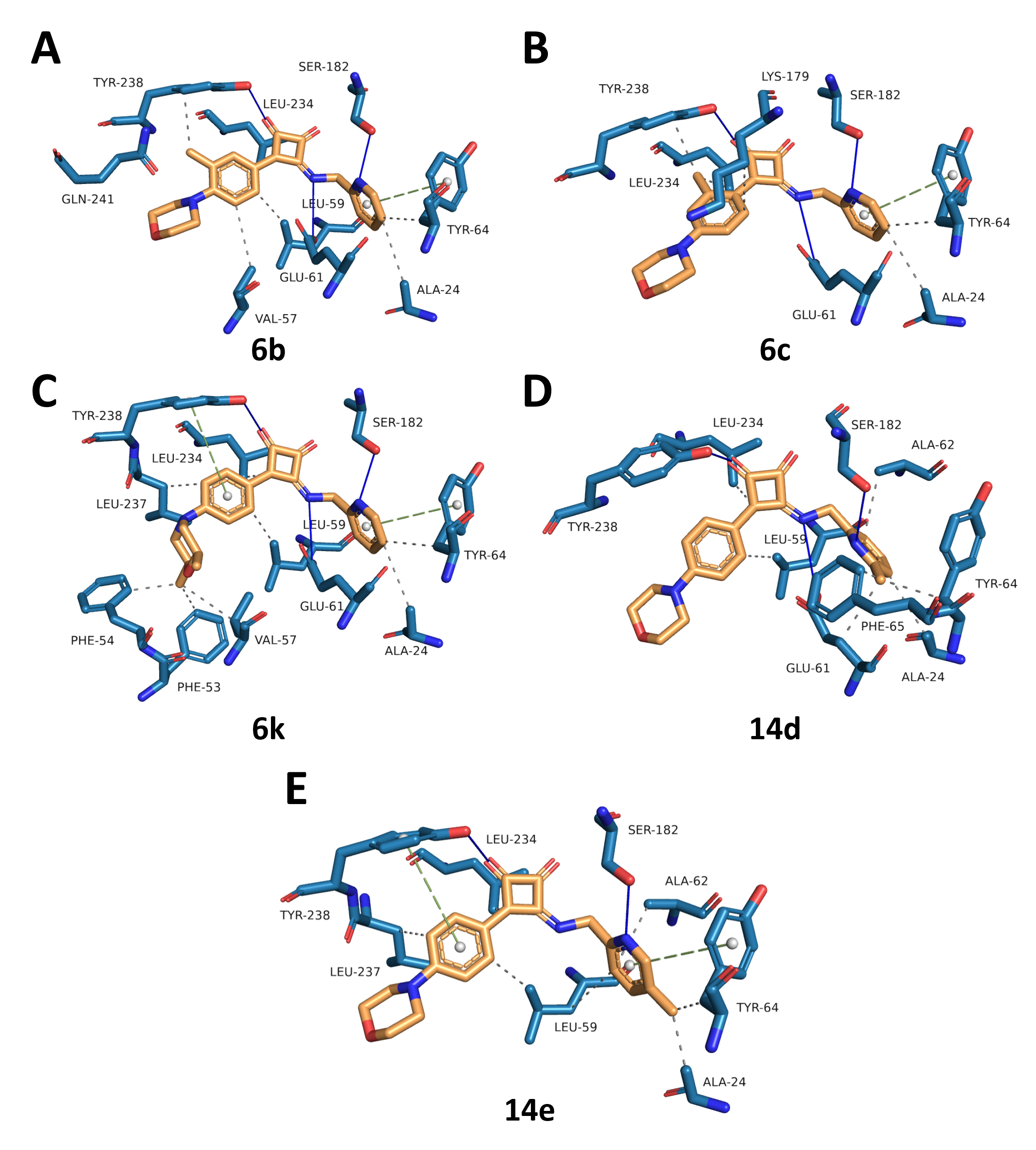
**Figure S4.** Docked poses of squaramide compounds **6b** (A), **6c** (B), **6k** (C), **14d** (D) and **14e** (E), based on PDB: 8G07. Binding site residues are shown as blue sticks, hydrogen bonds are shown as blue lines, hydrophobic interactions are shown as dotted grey lines, halogen bonding interactions are shown as green lines, and π-stacking interactions are shown as olive dashed lines. Interactions were detected using the Protein Ligand Interaction Profiler tool.^19^

**Table S3**. Summary of contributions and total MM/PBSA predicted binding energies derived from the last 20 ns of each simulation.

| **Compound** | **Run** | **ΔG_VdW_ (kJ/mol)** | **ΔG_electrostatics_ (kJ/mol)** | **ΔG_polar_ (kJ/mol)** | **ΔG_apolar_ (kJ/mol)** | **ΔG_binding_ (kJ/mol)** |
| --- | --- | --- | --- | --- | --- | --- |
| **A** | 1 | -212.027 ±11.351 | -102.859 ±6.817 | 230.955 ±11.657 | -20.028 ±0.65 | -103.958 ±12.997 |
|  | 2 | -250.117 ±11.103 | -78.193 ±11.937 | 200.732 ±9.946 | -19.888 ±0.646 | -147.467 ±12.557 |
|  | 3 | -240.062 ±9.017 | -58.001 ±6.422 | 172.134 ±10.348 | -20.985 ±0.627 | -146.913 ±11.875 |

**Table S4.** Summary of presence of protein-ligand interactions during each of the three simulations. The first 25 ns of simulation for each run were discarded.

| **Residue/ Chain** | **Interaction Type** | **Run_1** | **Run_2** | **Run_3** |
| --- | --- | --- | --- | --- |
| **ARG186_J** | Hydrogen Bond | 99.68% | 52.73% | 0.00% |
| **GLN241_J** | Hydrogen Bond | 0.00% | 19.92% | 0.00% |
| **GLU61_I** | Hydrogen Bond | 99.78% | 99.95% | 91.79% |
| **HIS164_J** | Hydrogen Bond | 0.78% | 0.00% | 0.00% |
| **LEU183_J** | Hydrogen Bond | 0.00% | 13.89% | 0.00% |
| **SER182_J** | Hydrogen Bond | 0.00% | 23.31% | 57.86% |
| **TYR238_J** | Hydrogen Bond | 99.82% | 90.69% | 0.00% |
| **ALA178_J** | Hydrophobic interaction | 0.00% | 0.00% | 99.74% |
| **ALA24_I** | Hydrophobic interaction | 41.23% | 0.00% | 66.76% |
| **ALA62_A** | Hydrophobic interaction | 98.94% | 84.85% | 95.76% |
| **ALA63_A** | Hydrophobic interaction | 0.00% | 51.62% | 92.80% |
| **GLU175_J** | Hydrophobic interaction | 0.00% | 0.00% | 86.11% |
| **GLU61_I** | Hydrophobic interaction | 92.39% | 90.95% | 55.64% |
| **ILE66_A** | Hydrophobic interaction | 68.49% | 0.00% | 0.00% |
| **LEU234_J** | Hydrophobic interaction | 62.82% | 0.00% | 0.00% |
| **LEU237_J** | Hydrophobic interaction | 87.54% | 55.26% | 0.00% |
| **LEU59_A** | Hydrophobic interaction | 94.32% | 98.46% | 0.00% |
| **LYS179_J** | Hydrophobic interaction | 99.94% | 96.93% | 83.55% |
| **PHE54_I** | Hydrophobic interaction | 1.10% | 0.00% | 39.34% |
| **PHE65_I** | Hydrophobic interaction | 86.93% | 0.00% | 98.80% |
| **TYR238_J** | Hydrophobic interaction | 0.00% | 97.84% | 0.00% |
| **TYR64_I** | Hydrophobic interaction | 99.63% | 76.26% | 98.67% |
| **VAL57_I** | Hydrophobic interaction | 88.91% | 97.55% | 90.31% |

1. **Measured permeability data**

**Table S5.** Measured permeability results for **6k**

| **Compound** | **Mean P_app_**  **(10^-6^ cm/s)** | | **Efflux Ratio** | **Mean %Solution Recovery** | | **Rank** | |
| --- | --- | --- | --- | --- | --- | --- | --- |
|  | **A to B** | **B to A** |  | **A to B** | **B to A** | **P_app_** | **Efflux Transporter Substrate** |
| **6k** | 9.83 | 13.9 | 1.42 | 50.2 | 69.6 | High | Poor or non |

1. ***In vivo* Pharmacokinetic data**

**Table S6.** Mean Plasma Pharmacokinetic parameters of **1f**, **6k** and **20j** after single IV dosing at 1 mg/kg (4 in 1 cassette) in male CD1 mice

| **PK parameters**  **(unit)** | **1f** | **6k** | **20j** |
| --- | --- | --- | --- |
|  |  |  |  |
| **CL (L/hr/kg)** | 6.26 | 3.14 | 3.88 |
| **V_ss_ (L/kg)** | 0.796 | 1.04 | 1.20 |
| **T_1/2_ (hr)** | 0.113 | 0.264 | 0.257 |
| **AUC_last_ (hr*ng/mL)** | 159 | 317 | 257 |
| **AUC_INF_ (hr*ng/mL)** | 160 | 319 | 258 |
| **MRT_INF_ (hr)** | 0.127 | 0.330 | 0.308 |

**
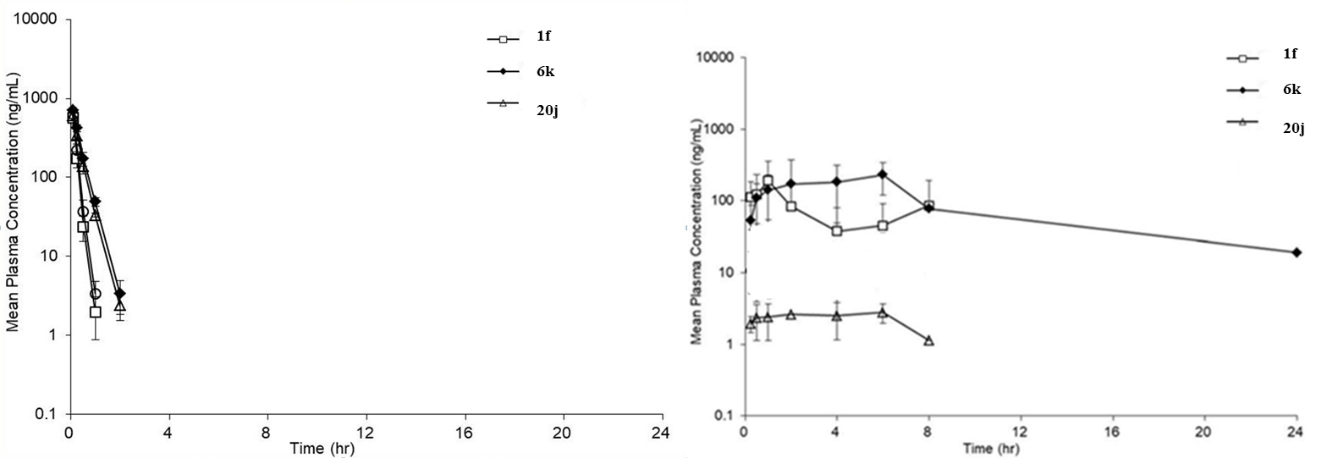
**

**Figure S5. A.** Mean plasma concentration-time profiles of **1f**, **6k** and **20j** after single dosing at 1 mg/kg (4 in 1 cassette) (left), Mean plasma concentration-time profiles of **1f**, **6k** and **20j** after single dosing at 20 mg/kg (4 in 1 cassette) (right)

**Table S7.** Mean Plasma Pharmacokinetic parameters of **1f**, **6k** and **20j** after single PO dosing at 20 mg/kg (4 in 1 cassette) in male CD1 mice

| **PK parameters**  **(unit)** | **1f** | **6k** | **20j** |
| --- | --- | --- | --- |
|  |  |  |  |
| **Tmax (hr)** | 1.00 | 6.00 | 6.00 |
| **Cmax (ng/mL)** | 191 | 231 | 2.80 |
| **T_1/2_ (hr)** | NA | NA | NA |
| **AUClast**  **(hr*ng/mL)** | 594 | 2092 | 18.9 |
| **AUC_INF_**  **(hr*ng/mL)** | NA | NA | NA |
| **F (%)** | 18.6 | 33.0 | 0.367 |


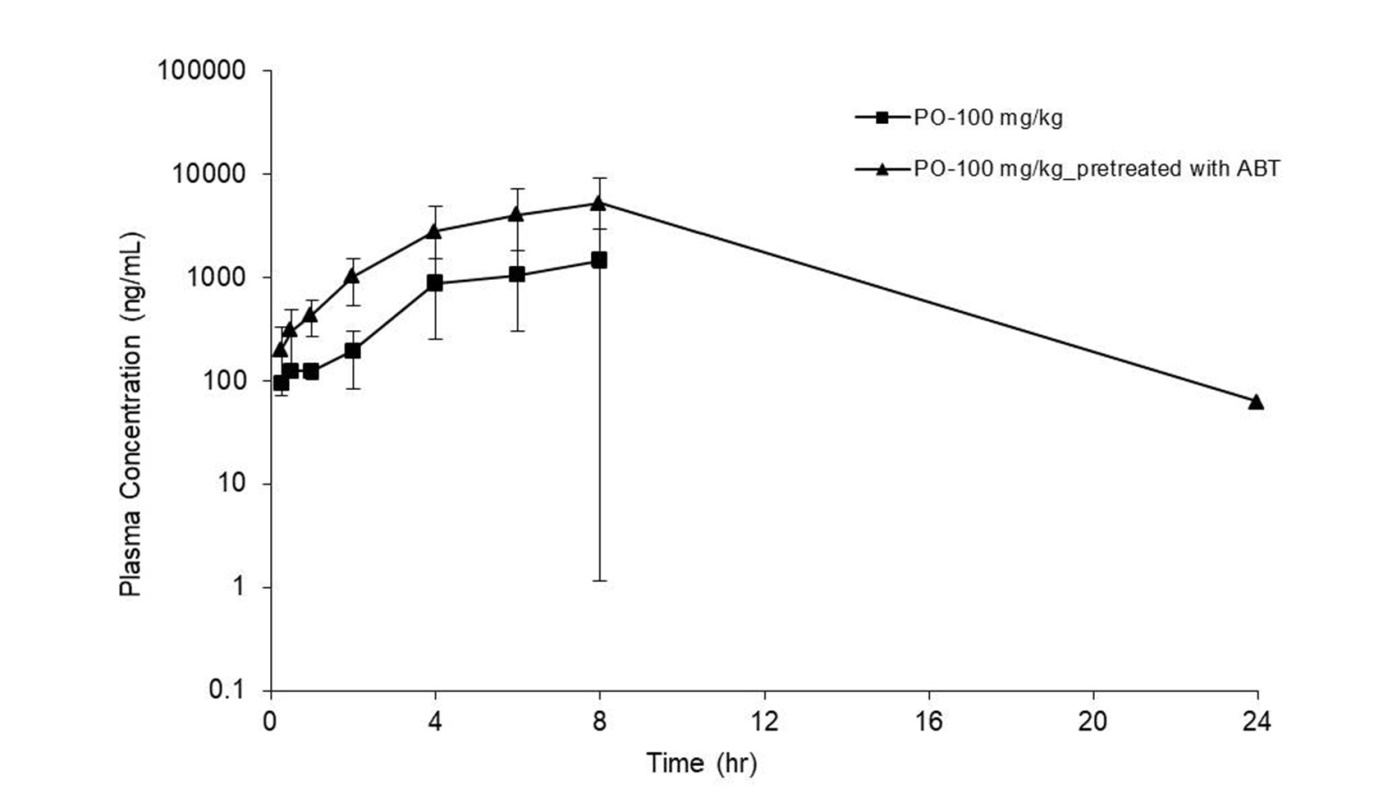


**Figure S6.** Mean plasma concentration-time profile of **6k** single oral dosing at 100 mg/kg in male CD1 mice with and without ABT

**Table S8.** PK parameters of **6k** at 100 mg/kg dose with and without ABT pre-treatment

| **Mean plasma concentration-time data of 6k after a PO dose at 100 mg/kg in male CD1 mice** | | | | | **Mean plasma concentration-time data of 6k after a PO dose at 100 mg/kg in male CD1 mice_pretreated with ABT** | | | | | |
| --- | --- | --- | --- | --- | --- | --- | --- | --- | --- | --- |
| **PK parameters** | Unit | **Mean** | SD | CV (%) | **PK parameters** | Unit | **Mean** | SD | CV (%) |  |
| **T_max_** | hr | **6.67** | 2.31 | 34.6 | **T_max_** | hr | **8.00** | 0.00 | 0.00 | |
| **C_max_** | ng/mL | **1691** | 1377 | 81.5 | **C_max_** | ng/mL | **5267** | 4034 | 76.6 | |
| **Regression time** | hr | **NA** | NA | NA | **Regression time** | hr | **NA** | NA | NA | |
| **T_1/2_** | hr | **NA** | NA | NA | **T_1/2_** | hr | **NA** | NA | NA | |
| **AUC_last_** | hr*ng/mL | **5818** | 3758 | 64.6 | **AUC_last_** | hr*ng/mL | **59679** | 53707 | 90.0 | |
| **AUC_INF_** | hr*ng/mL | **NA** | NA | NA | **AUC_INF_** | hr*ng/mL | **NA** | NA | NA | |


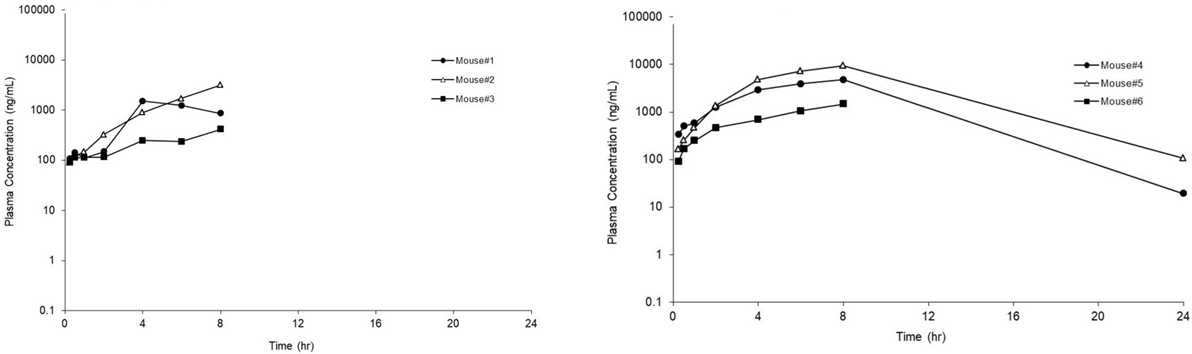


**Figure S7.** Individual Plasma concentration-time profiles of **6k** after single PO dosing at 100 mg/kg in male CD1 mice without ABT (left) and with ABT (right)

In conclusion, novel squaramides were designed and prepared. SQAs with LHS morpholine modifications offered the best metabolic stability with good potency. While the RHS 6-fluoro pyridine squaramide exhibited significant improvement in activity. Compounds with both LHS and RHS modifications displayed good pharmacokinetic profiles and optimum potency. The most stable analogue prepared was **6k** which has been tested *in vivo* for its pharmacokinetic properties and display enhanced bioavailability.

1. ***In vivo* Pharmacodynamic data**

Initially, we evaluated the *in vivo* activity of **6k**, combined with ABT, and compared it to that of BDQ in a lethal subacute mouse infection model of tuberculosis (TB to help understand its potential for further development. Mice were infected by the aerosol route 14 days prior (D-14) to the initiation of treatment (D0). In the first week of treatment, all untreated mice, and some of the treated mice reached a humane endpoint and required euthanasia to avoid undue suffering of mice and in accordance with our animal care and use committee guidelines. The lungs were plated for CFU wherever possible. At the end of 1 month of treatment (M1), all surviving mice were sacrificed and their lungs plated for CFU. The details of CFU counts for all regimens are given in **Figure S8** and **Table S9**. In mice infected with H37Rv wild type strain, linezolid (LZD) treatment resulted in an approximately ½-log reduction in CFU after 1 month of treatment (**Figure S8** and **Table S9**). Telacebec (Q203) treatment had bacteriostatic activity in both infection groups, with similar CFU counts after 1 month of treatment and at baseline D0 (**Figure S8** and **Table S9**).

In mice treated with **6k_100_ + ABT**, 3 of the 5 mice reached a humane endpoint and required euthanasia. However, the remaining 2 mice survived to complete treatment. The mean CFU count from the mice sacrificed early was 7.93 ± 0.18 log_10_ CFU/lung, similar to the CFU counts at D0, but lower than the untreated controls indicating that **6k** had at least early bacteriostatic activity against Mtb. The mean CFU count after 1 month of treatment in the surviving 2 mice was 7.68 ± 0.19 log_10_ CFU/lung indicating that **6k** may have weak bactericidal activity at this dose (**Figure S8** and **Table S9**). Thus, **6k** displayed weak bactericidal or bacteriostatic *in vivo* activity although it was not as effective as BDQ. A follow-up experiment using chronic mouse infection model of TB was then performed to further confirm the activity of **6k** and compare it to that of the lead SQA **1f** (**Figure 7**, **Table S10**).


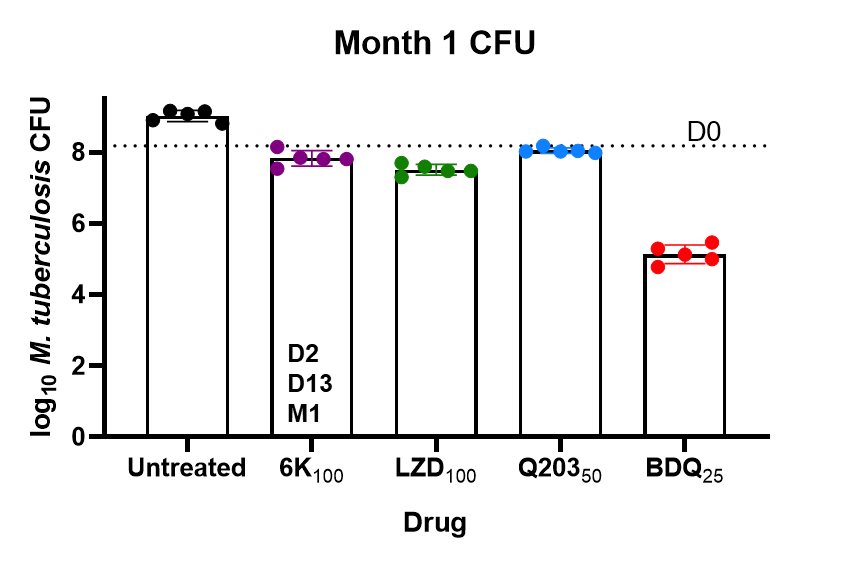


**Figure S8.** Log_10_ CFU/lung after 1 month of treatment in H37Rv infected mice. The baseline D0 counts at the start of treatment are shown by dotted line. Mice sacrificed early are indicated by D followed by a number indicating the day of sacrifice after starting treatment, D2= 8.15, D3= 7.85 and 7.81, versus mice completing treatment at M1= 7.81 and 7.54. One month of treatment with **LZD_100_** resulted in reduction of 0.64 logs. In **6k_100_** treated group, 3 mice had to be sacrificed early while remaining 2 mice recovered and were sacrificed at month 1. The overall CFU count in **6k** treated groups was slightly lower than D0 counts indicating that it was bacteriostatic or weakly bactericidal. **Q203_50_** was mostly bacteriostatic.

**Table S9.** Mean (± SD) lung log_10_ CFU counts in acute mouse infection model of TB

| **Regimen** | | **Mean log10 (±SD) lung CFU counts and other outcomes by time point** | | | | |
| --- | --- | --- | --- | --- | --- | --- |
|  | | **D-14** | **D0** | **Mice Sac’d Early**  **(Day/ Number)** | **M1** | |
| **Untreated** | | 4.72±0.03 | 8.18±0.18 | 9.12±0.06 (D4/ n =5) | All Sac’d Early | |
| **LZD_100_** | |  |  |  | 7.54±0.09 | |
| **Q203_50_** | |  |  |  | 8.10±0.11 | |
| **6k_100_+ABT_100_** | |  |  | 7.93±0.18 (D2 & D3/ n =3) | 7.68±0.19 (n= 2) | |
| **BDQ_25_** | |  |  |  | 5.13±0.27 | |
| *Subscripts indicate dose in mg/kg administered daily 5 days per week.*  *ABT was given 2 hrs before* ***6k****.*  In mice sacrificed earlier than scheduled, D = day of death | | | | | | |

**Table S10.** Mean (± SD) lung log_10_ CFU counts following 4 weeks of treatment in chronic mouse infection model of TB

| **Regimen** | **D-41** | **D0** | **M1** |
| --- | --- | --- | --- |
| **Untreated** | 1.38±0.03 | 6.36±0.27 | 5.72±0.20 |
| **6k_100_ only** |  |  | 5.97±0.09 |
| **6k _100_+ABT_100_** |  |  | 5.18±0.17 |
| **1f_100_+ABT_100_** |  |  | 5.22±0.15 |
| **BDQ_25_** |  |  | 3.76±0.20 |

1. **Predicted DMPK data**

Since one of the main goals of our investigation is to enhance the squaramides metabolic stability, it was essential to study the metabolism of squaramides. This is in order to gain more in-depth understanding of the biodegradative pathways and to guide us in our endeavor to develop more metabolically stable analogues. The metabolism prediction software (StarDrop)^24^ was utilised to investigate whether our strategies to mitigate the molecules’ metabolism were successful. In addition to providing insights into other possible liable soft spots that could guide us in designing new targets with reduced biodegradation rates. The module also provides knowledge of the possible metabolic routes for the developed squaramides.

The program uses quantum mechanical simulations to predict the sites of metabolism in the molecule and the CYP450 enzyme isoforms that are likely to mediate metabolism. Additionally, the module also predicts the possible metabolites. There are seven major CYP450 isoforms that the module examines are 3A4, 2D6, 1A2, 2C9, 2C8, 2C19, 2E1. Upon analysis of the squaramide analogues, it has been found that the enzymes 3A4, 2D6 and 1A2 are the major isoforms involved in the compounds’ metabolism. The enzyme CYP3A4 is the most abundant isoform in the liver and is responsible for the metabolism of at least 50% of pharmaceutical drugs.^25^ The active site for 3A4 is also very promiscuous in terms of its substrate specificity and binding.^26^ Although 2D6 and 1A2 are less abundant, but they mediate the metabolism of diverse drugs.^25^ However, we mostly focused on the metabolism of 3A4 as it accounts for at least 70% of our squaramide compounds’ metabolism as predicted by StarDrop.

We first looked at the metabolic sites of the initial lead compound **1f**, where the morpholine ring was found to be the most prone to metabolism with the 2 carbons next to oxygen being the most vulnerable ones (**Figure S9**). The 2 CH_2_ carbons next to the morpholine nitrogen were ranked second in metabolic susceptibility (**Figure S9**).


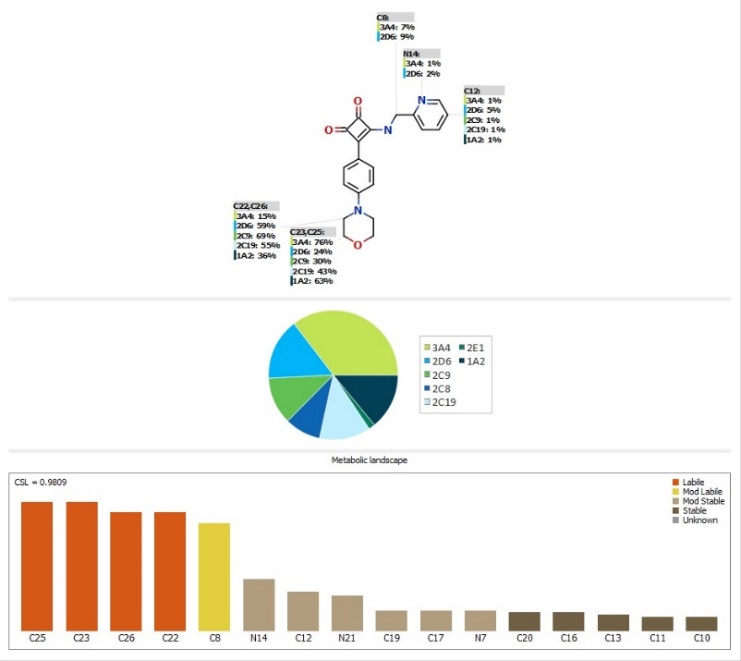

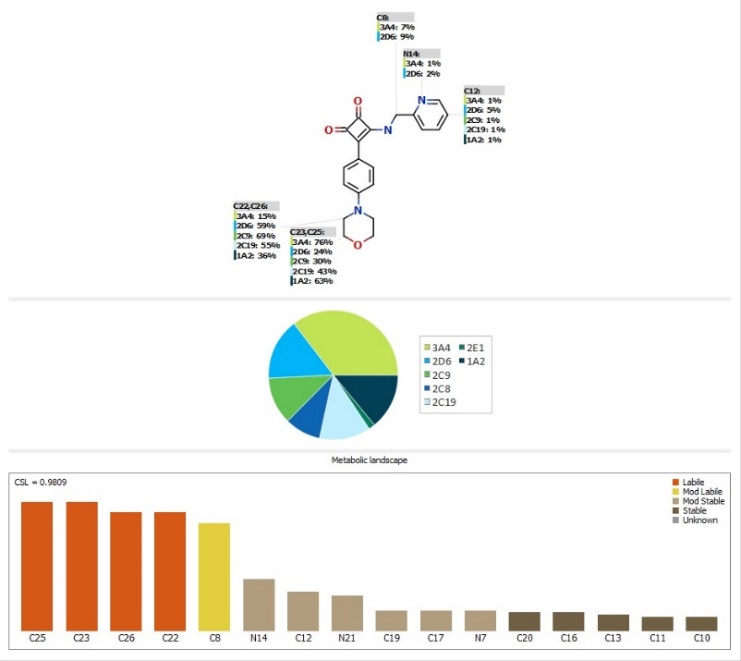

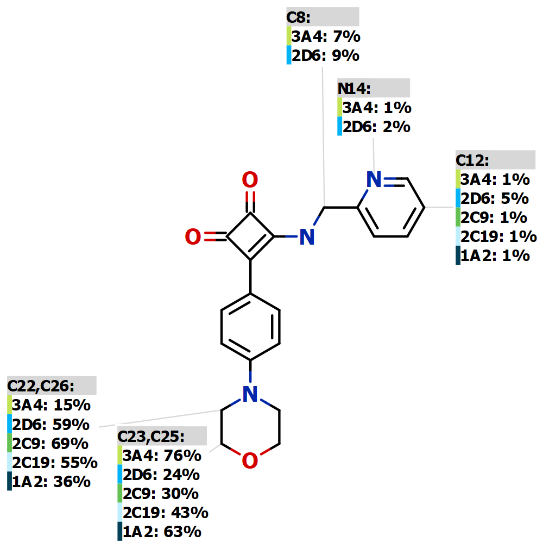


**Figure S9.** StarDrop metabolism prediction of the lead compound **1f** (left) and its overall metabolic analysis (right)

These findings confirmed our hypothesis about morpholine ring being the most liable to metabolism. This agrees with the reported metabolic fate of the morpholine moiety, which is predominantly metabolised by CYP3A4 enzyme *via* oxidation, leading to a morpholine lactone or lactam.^27^ Ring opening can then take place forming the corresponding amine or hydroxyl carboxylic acid (**Figure S10**). ^27^

**Figure S10.** Suggested metabolic pathways for the SQA morpholine moiety

Upon actual DMPK testing, it has been found that these StarDrop metabolic predictions match our findings with the relatively high clearance rates observed in rat hepatocytes and human microsomes for **1f**. Therefore, modification or bioisosteric replacement of the morpholine ring was justified.


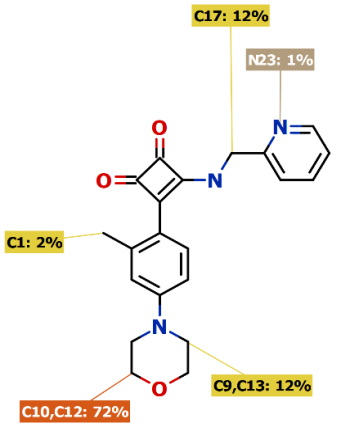

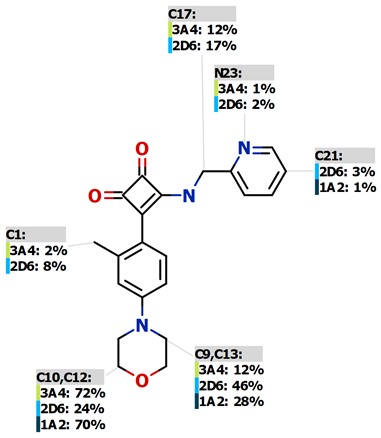
As noted below, modifications of the phenyl ring at both *ortho* and *meta* positions (**6b-c**) to the morpholine moiety displayed no improvement neither against 3A4 nor in the overall metabolism of the target SQAs (**Figure S11**).


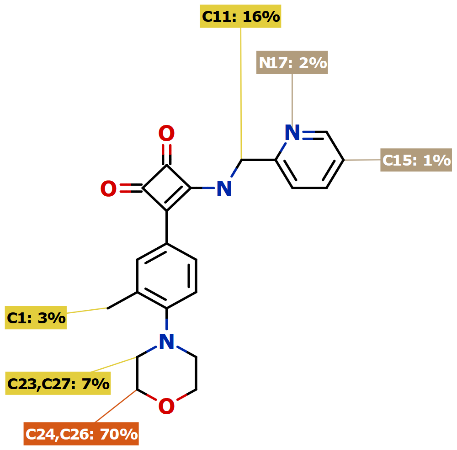


**Figure S11.** StarDrop metabolism prediction of the squaramide **6b** (left) against 3A4, predictions for **6c** (middle) and its overall metabolic analysis (right)

On the other hand, morpholine ring methylation (**6g-h**) showed more stability, particularly against 3A4 enzyme, possibly due to topographic changes of the morpholine ring which led to weaker binding affinity to the CYP450 enzyme active site (**Figure S12**).


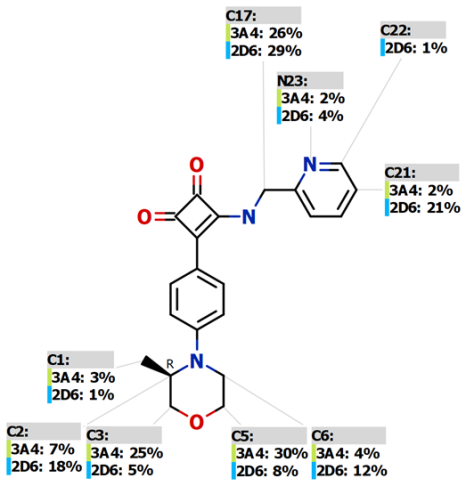

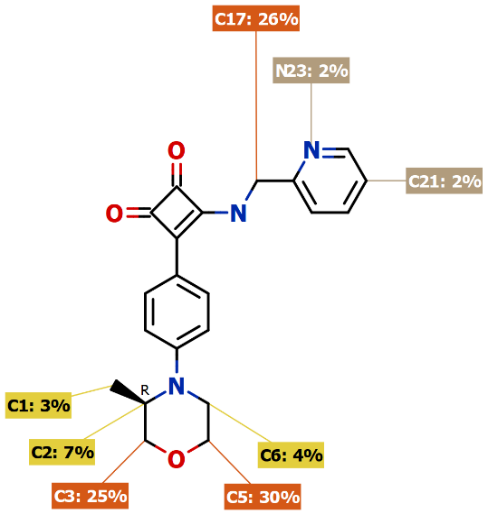


**Figure S12.** StarDrop metabolism prediction of the squaramide **6h** against 3A4 (left) and its overall metabolic analysis (right)

The 2,6-dimethyl morpholine squaramide **6j** was suggested in order to block the metabolism at the 2 CH_2_ carbons adjacent to the morpholine oxygen, hoping to enhance the compound’s stability. Although no significant metabolic reduction was predicted by StarDrop^24^, the measured clearance rates for **6j** were low to moderate. These findings confirm the positive correlation observed between morpholine ring methylation and metabolic stability (**Figure S13**). Similarly, the *cis* isomer **6k** displayed the same metabolic liabilities as **6j**, despite showing low-to-moderate metabolic clearance rates (**Figure S13**).


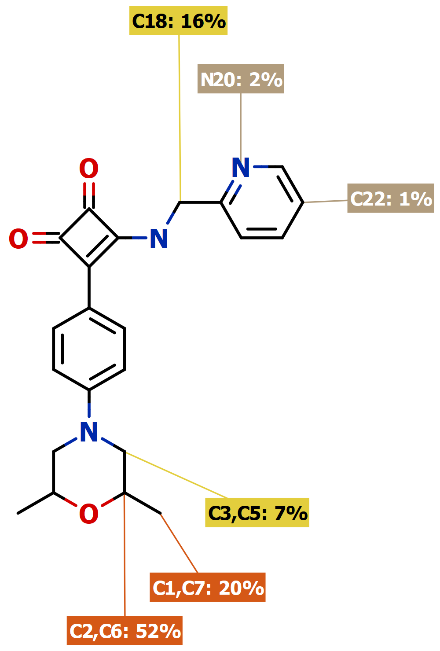

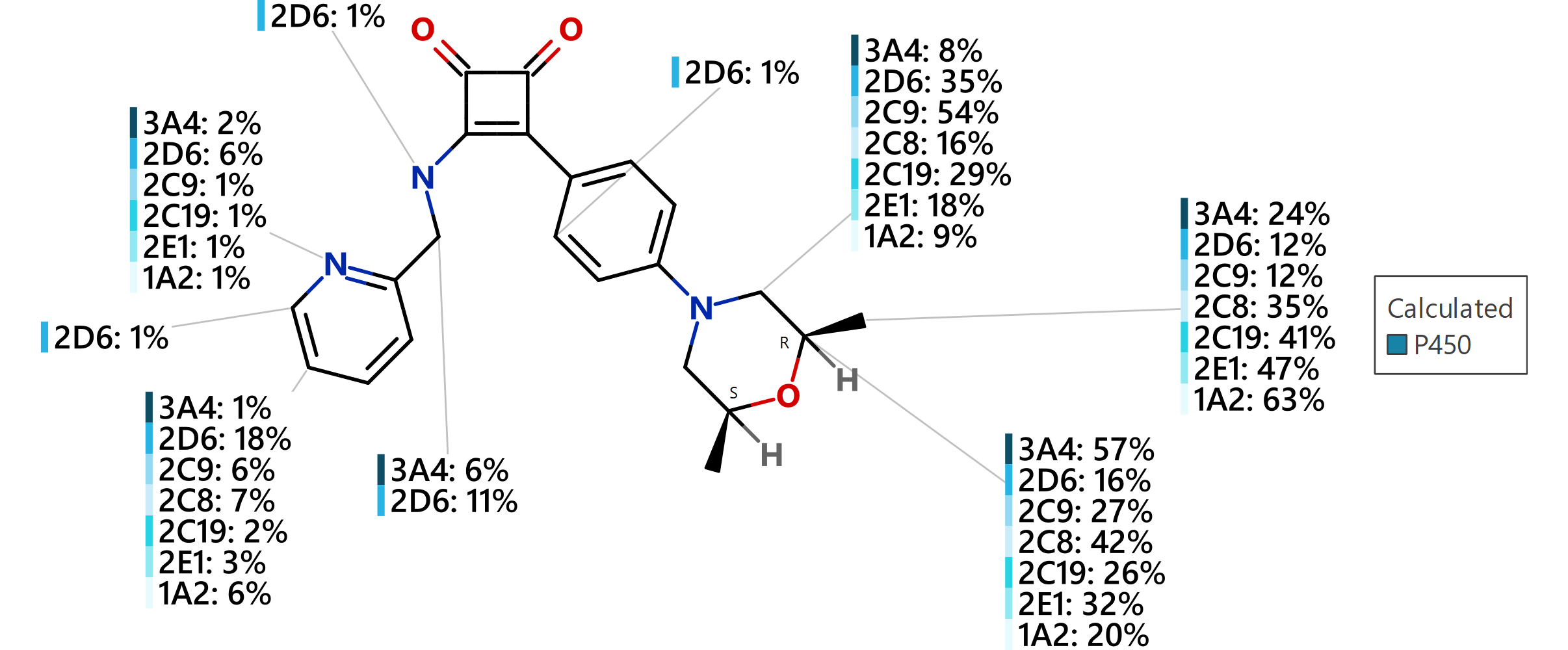


**Figure S13.** StarDrop metabolism prediction of the squaramide **6j** against 3A4 (left) and the overall metabolic analysis of **6k** (right)

Replacement of the morpholine ring with piperidine derivatives in general was not successful, none of the analogues predicted any significant enhancement of metabolic resistance except for **6o**. This is due to the lack of a tertiary CH-F bond in **6o**, which is the most susceptible site of metabolism in **6n**. These findings come in agreement with the measured DMPK, although the values for **6n** and **6p** were much higher than their morpholine counterparts, with exception of **6o** which showed good stability in human microsomes. The bridged morpholine analogues **6q-r** were expected to exhibit higher stability particularly on the heterocyclic ring. But they appear to have increased the susceptibility of the aminomethyl linker to metabolism *via* *N*-dealkylation, followed by oxidation. This could potentially be linked to the altered compound orientation that may have allowed increased binding to CYP3A4. Replacement of the 6-membered morpholine with an 8-membered bioisostere did not provide satisfying metabolic resistance as seen for the 8-oxa-3-azabicyclo[3.2.1]octane **6s**, both in StarDrop predictions and in our DMPK.

In terms of right-hand side modifications, pyridine ring substitution with methyl, fluoro, chloro or methoxy groups **13a-e** and **14d-i** was predicted to show comparable metabolism to the lead compound **1f** (**Figure S14**). A similar pattern was observed for shifting the position of pyridine nitrogen, incorporating an extra nitrogen as in pyrimidine rings, or replacing the pyridine with pyrrolidine or thiazole heterocycles **13f-g, 14a-c** and **14j** (**Figure S14**). This further highlights our theory that morpholine ring modifications can enhance the metabolic stability, while pyridine ring alterations can improve the potency.

The predictions showed some correlation to our measured *in vitro* DMPK data. However, some compounds displayed better metabolic stability while others exhibited high clearance rates.


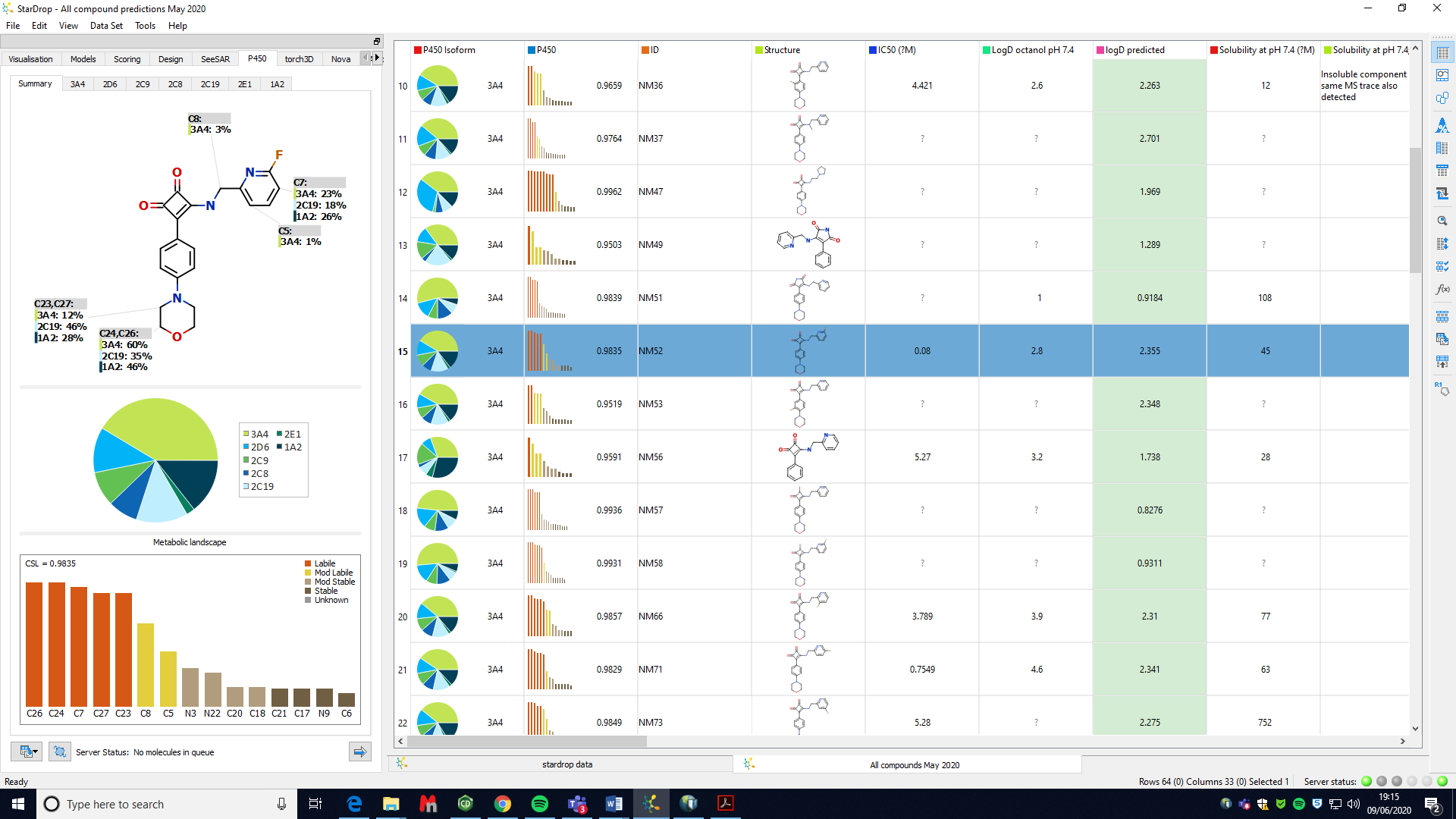

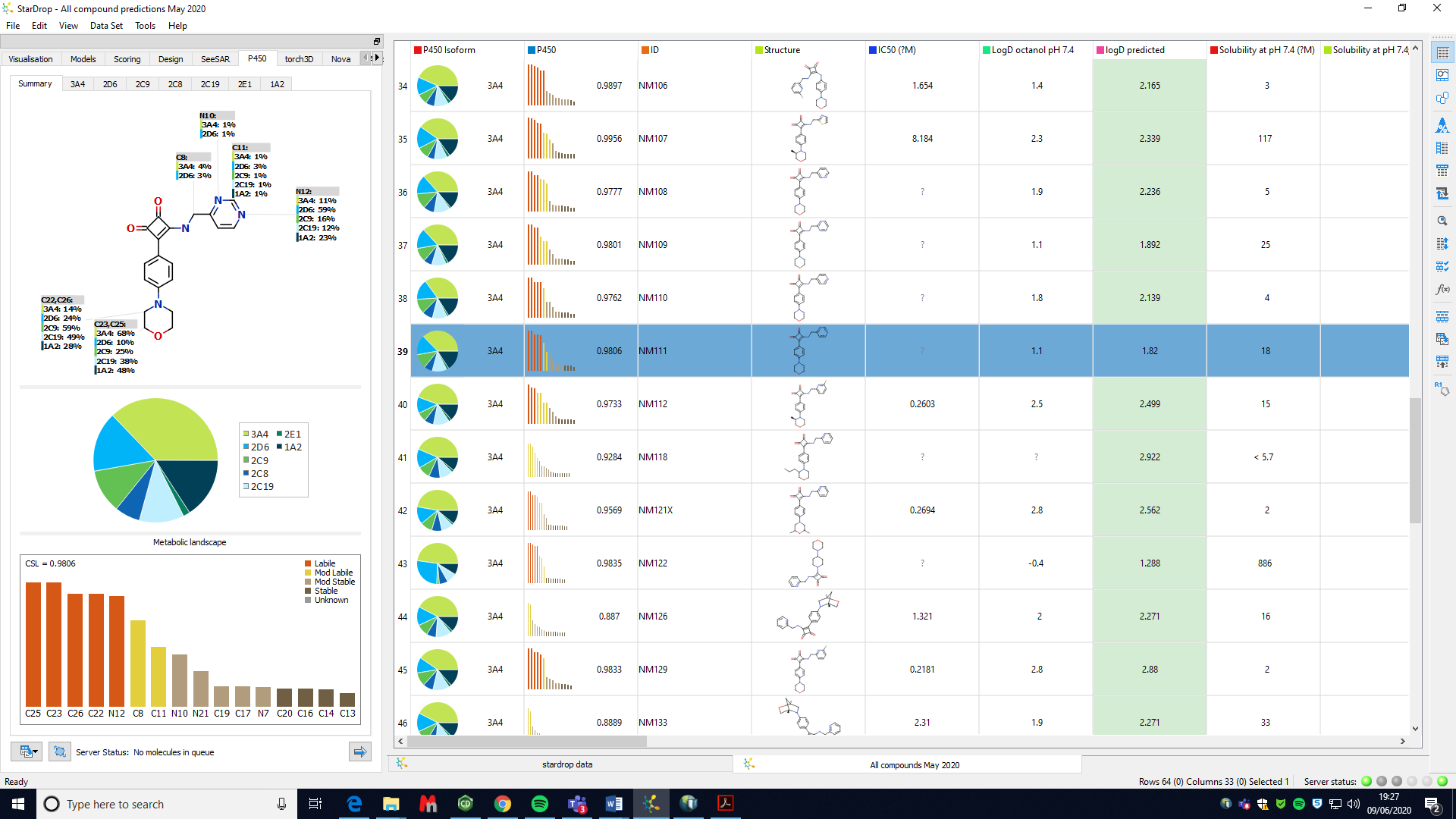


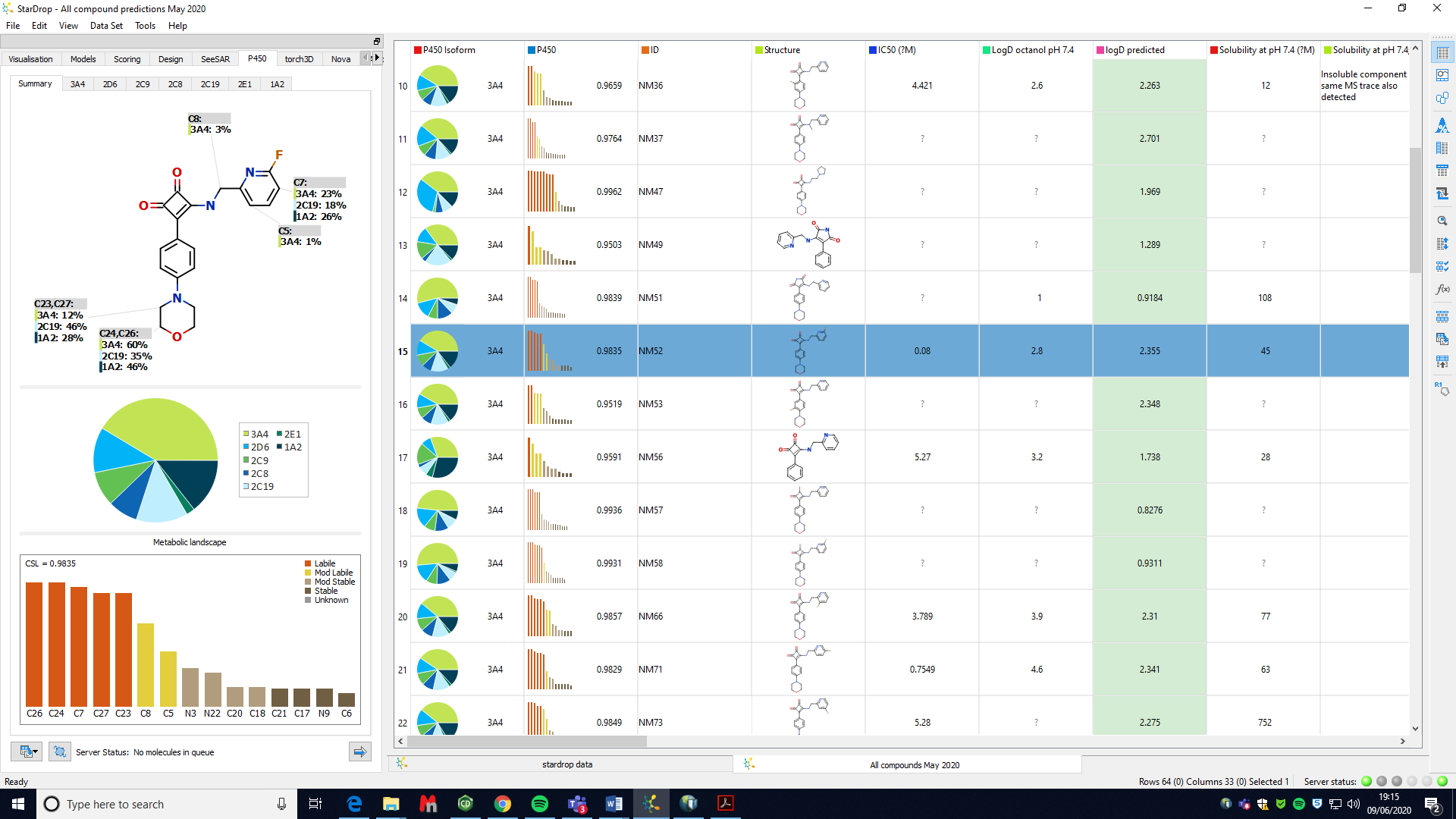

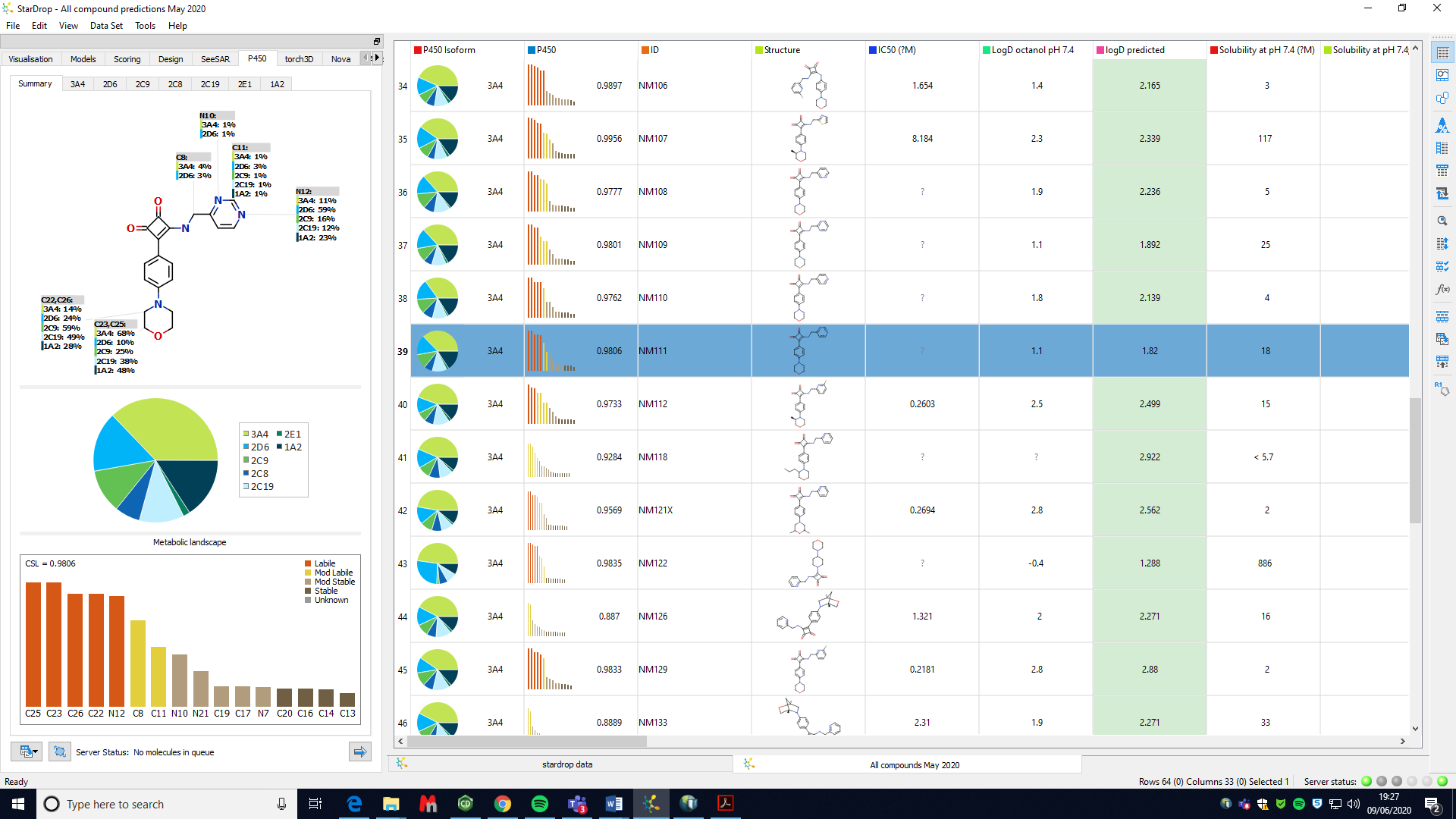


**Figure S14.** StarDrop metabolism prediction of the squaramide **13a** (left) and **13g** (right)

We assumed then that combining the stable morpholine bioisoteres with the potent 6-fluoropyridine RHS could enhance the metabolic stability of the resulting derivatives while achieving improved TB inhibitory activity. The morpholine bioisosteric heterocycles are expected to block the metabolism on the heterocyclic ring either by ring alkylation or by halogenation.

Indeed, a general trend of increased metabolic stability was observed for the squaramides with both RHS and LHS modifications, particularly for the modified heterocyclic rings.

Predictions of the combined fluoropyridine and both 3-*S* and 3-*R* Me-morpholines **20a-b** were similar. The analogue **20a** showed significant reduction in biodegradation of the morpholine ring against the enzyme 3A4 (**Figure S15**), while it displayed higher metabolism *via* aromatic hydroxylation on the carbon *ortho* to the fluoro group on the pyridine ring. Although fluorination is a well-adopted strategy for blocking metabolism soft spots, it is not always the case as seen with our squaramide derivatives.^28^ Aromatic hydroxylation by CYP450 could still occur at the *ortho* and *para* positions to the C-F bond, with significantly more prominent effects for the *ortho* position. A suggested explanation for the observed findings is the altered ring conformation of the fluoro pyridine RHS that could have led to enhanced binding with the metabolising cytochrome P450 enzyme resulting in increased biodegradation.

Nevertheless, the developed analogues were still more stable than the lead compound **1f**, therefore, these predictions help verify their *in vitro* metabolic rates which were moderate in both human microsomes and rat hepatocytes.


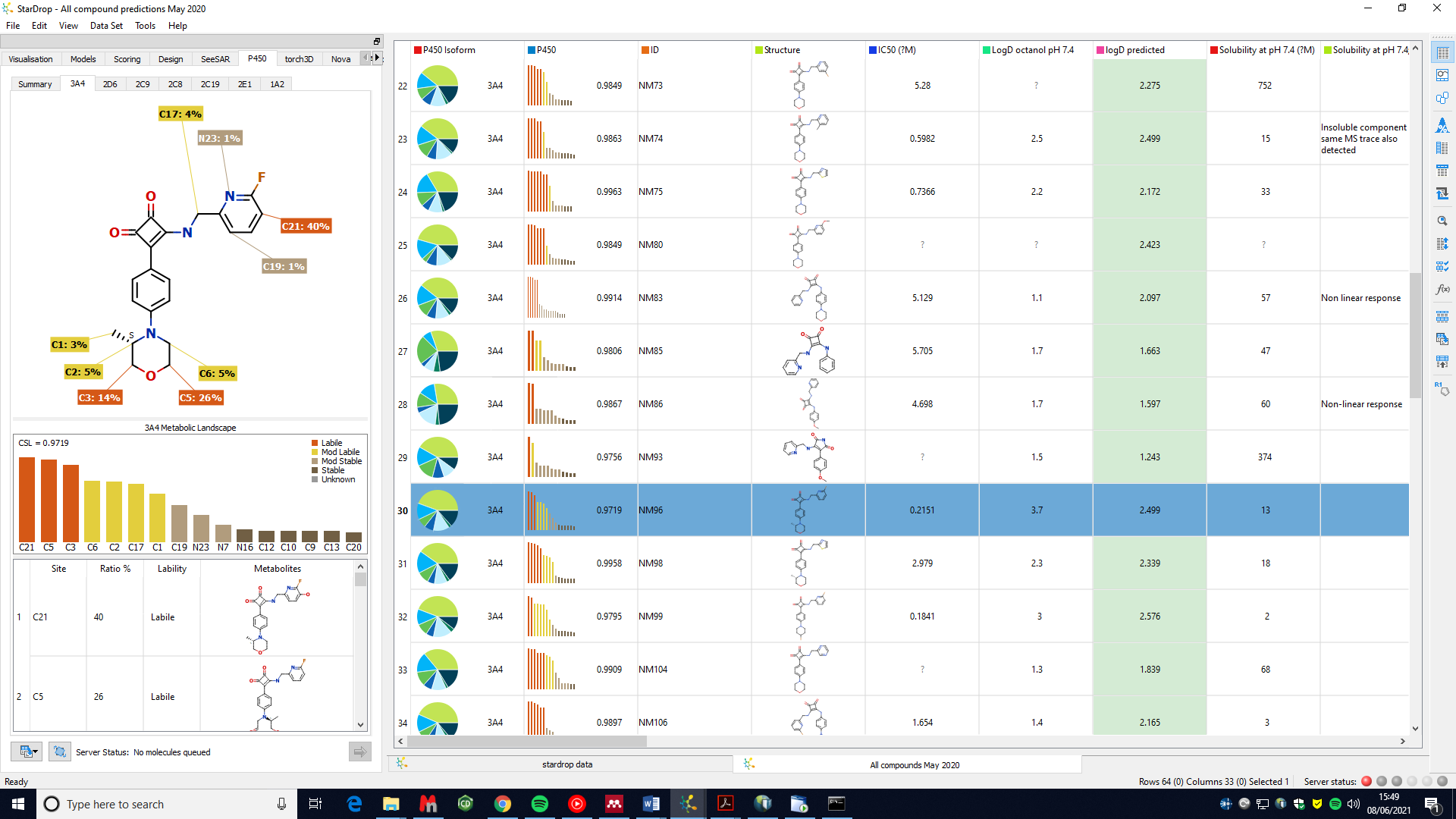

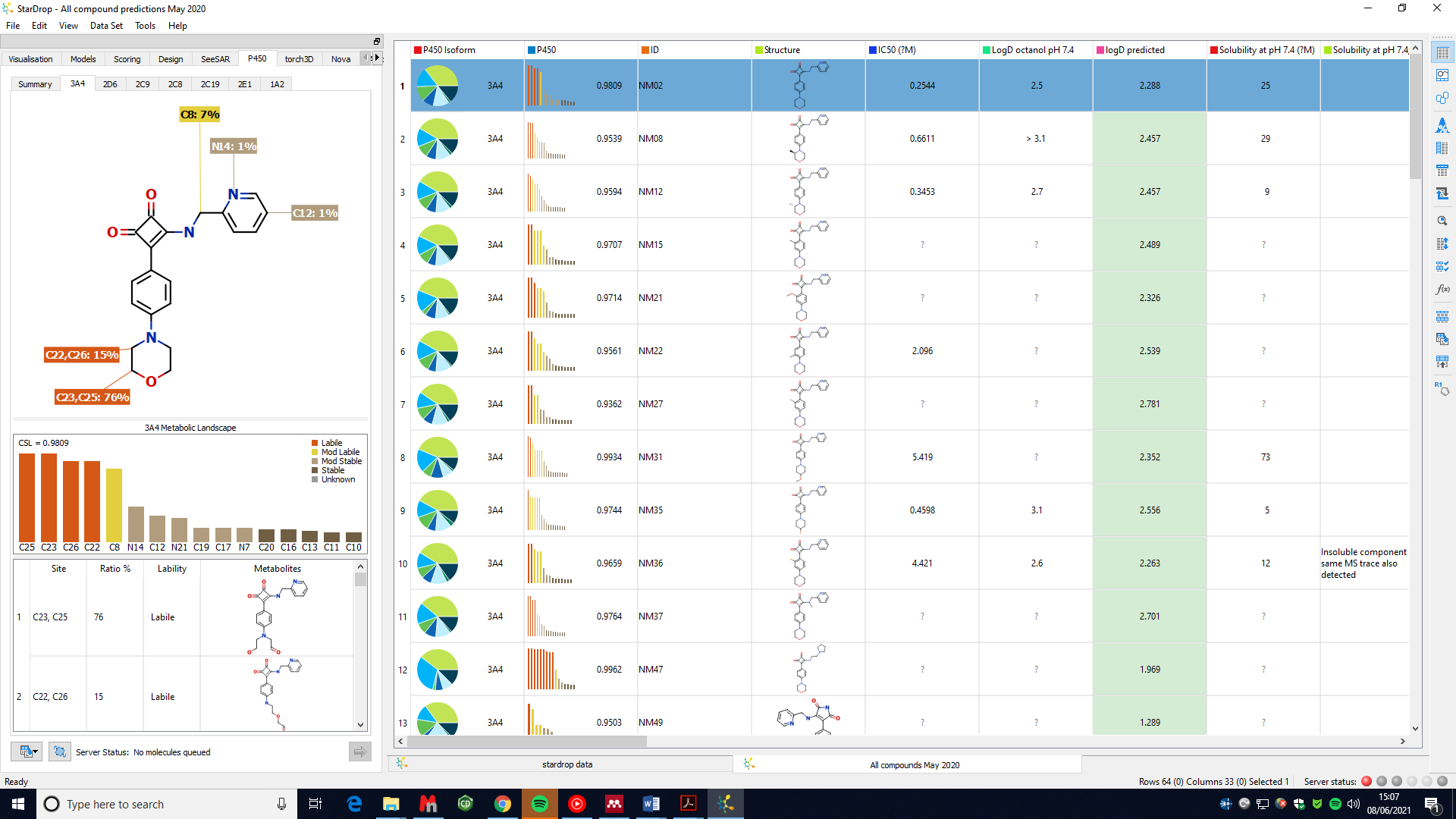


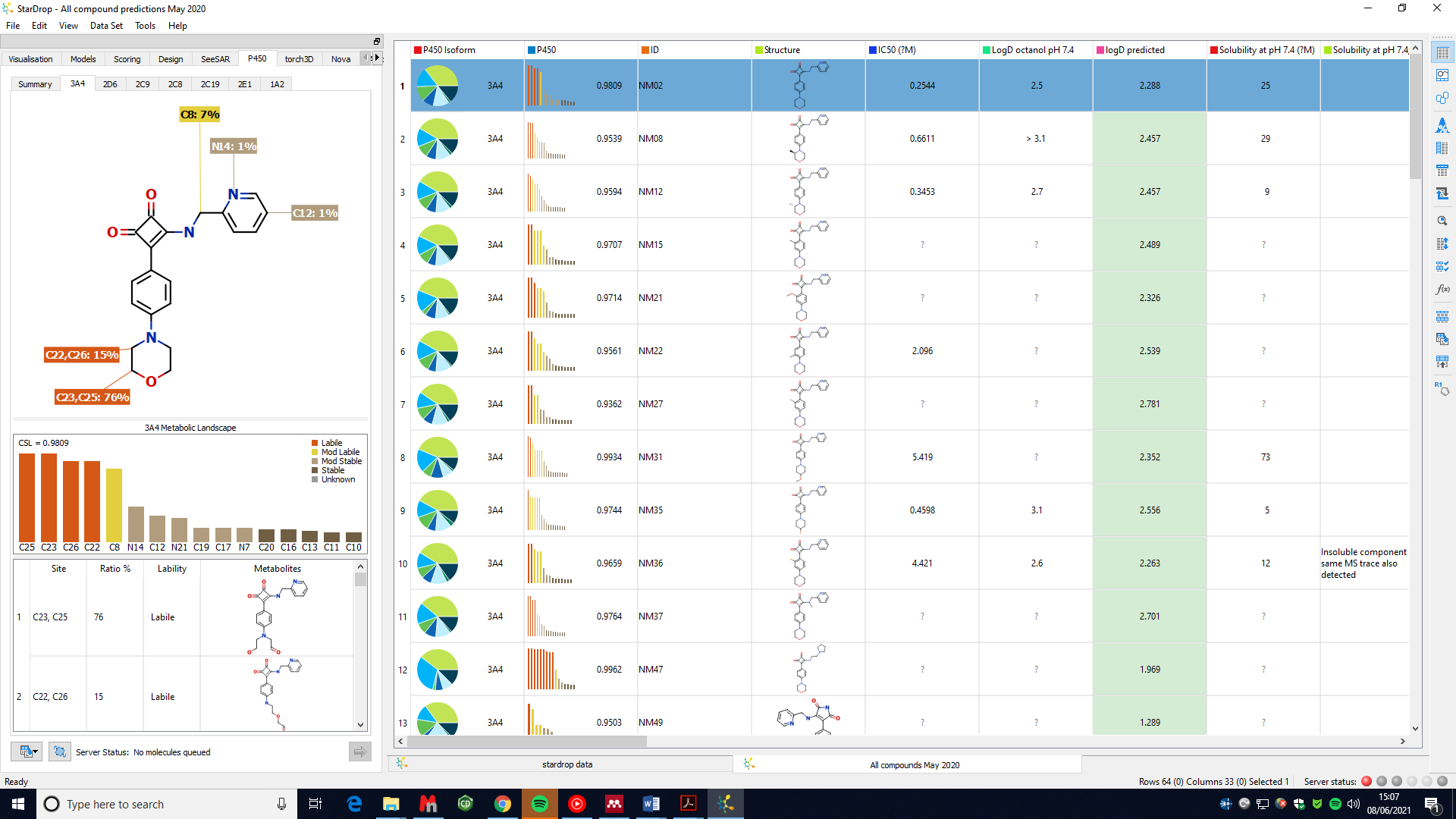

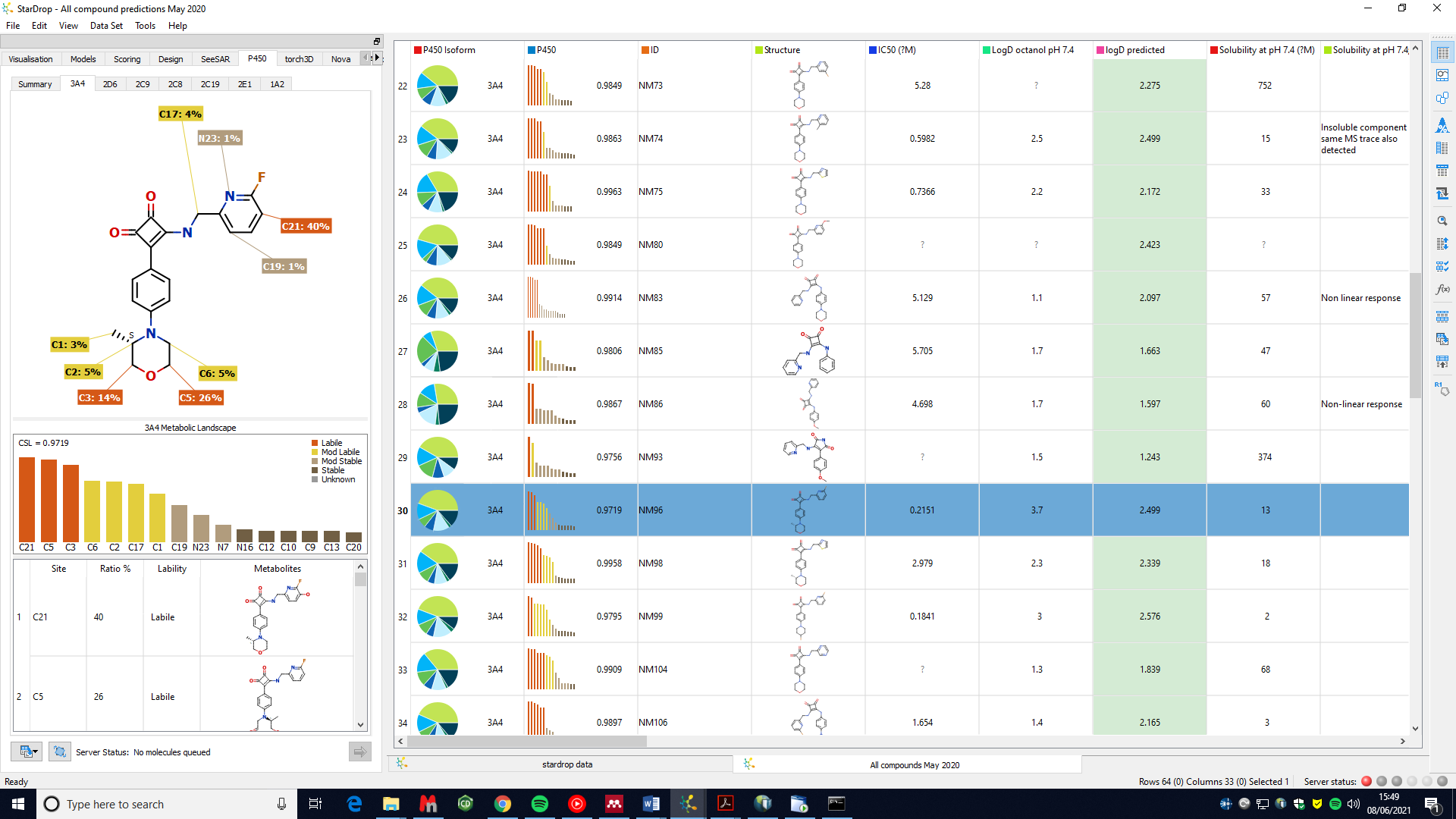


**Figure S15.** StarDrop metabolism prediction of the lead squaramide **1f** (left) and compound **20a** (right) against 3A4 enzyme.

1. **Additional experimental**

**Synthesis of 3,4-dichlorocyclobut-3-ene-1,2-dione (2b)**^29^. A mixture of squaric acid (1.14 g, 10 mmol, 1.0 eq.), thionyl chloride (1.8 mL, 25 mmol, 2.5 eq.) and DMF (0.2 mL) in dry toluene (10 mL) was allowed to heat to reflux at 60 °C for 6 hours. The solvent was then evaporated *in vacuo* and hexane (20 mL) was added. The soluble product was separated from the gummy residue by decantation. The pure product was then concentrated under reduced pressure and cooled to 0 °C to form **2b** as an orange-brown amorphous crystals. The product was stored in the freezer. Yield: 37-76% (0.56-2.28 g). ^13^C NMR (101 MHz, CDCl_3_) δ 189.41, 188.08; HRMS (CI+) *m/z*: calculated for C_4_H^35^Cl_2_O_2_ [M+H]^+^: 150.9348; found: 150.9349 (Diff: -0.66 ppm).

**Synthesis of LHS derivatives 3b-s**

**4-(*o*-tolyl)morpholine (3b).** In a screw-cap vial equipped with a magnetic stir bar, 2-bromotoluene (1.05 mmol, 1.05 eq.), morpholine (1 mmol, 1 eq.), Pd(OAc)_2_ (2.2 mg, 0.002g, 0.01 mmol), RuPhos (9.3 mg, 0.009 g, 0.02 mmol), and powdered NaO*t*Bu (0.115g, 1.2 mmol) were added. The vial was transferred to an aluminium heating block (110 °C). The reaction mixture was allowed to heat at 110 °C for 24 hours, and then it was cooled and dissolved in DCM/H_2_O mixture (1:1). The organic phase was collected, and the solvent was evaporated *in vacuo*. The product was purified using flash column chromatography on silica gel using 40 % EtOAc:hexane as an eluent system o afford **3b** a colorless oily liquid in a 78 % yield (0.685 g). ^1^H NMR (400 MHz, CDCl_3_) δ 7.31 – 7.13 (m, 2H), 7.07-6.93 (m, 2H), 3.90 – 3.79 (m, 4H), 2.94 – 2.83 (m, 4H), 2.31 (s, 3H). ^13^C NMR (101 MHz, CDCl_3_) δ 151.31, 132.65, 131.19, 126.68, 123.43, 118.98, 67.48, 52.28, 17.88.

**4-(*m*-tolyl)morpholine (3c)** prepared according to general procedure for **3b** using 3-bromotoluene (1 eq.) and morpholine (1 eq.) as a pink oily liquid in a 78 % yield (1.58 g). The product was purified using flash column chromatography on silica gel using 35 % EtOAc:hexane as an eluent system. ^1^H NMR (400 MHz, CDCl_3_) δ 7.16 (t, *J* = 7.8 Hz, 1H), 6.81 – 6.60 (m, 3H), 3.98 – 3.77 (m, 4H), 3.29 – 2.98 (m, 4H), 2.32 (s, 3H). ^13^C NMR (101 MHz, CDCl_3_) δ 151.39, 138.90, 129.04, 120.98, 116.57, 112.89, 67.00, 49.50, 21.79. HRMS (CI+) *m/z*: calculated for C_11_H_16_NO [M+H]^+^: 178.1226; found: 178.1226 (Diff: -0.01 ppm).

**4-(3-chlorophenyl)morpholine (3d)** prepared according to the general procedure for **3b** using 1,3-dichlorobenzene (1 eq.) and morpholine (1 eq.) as a colorless oily liquid in an 84 % yield (0.95 g). The product was purified using flash column chromatography on silica gel using 20 % EtOAc:hexane as an eluent system then the polarity was increased to 30 % EtOAc:hexane. ^1^H NMR (400 MHz, CDCl_3_) δ 7.17 (t, *J* = 8.3 Hz, 1H), 6.90 – 6.80 (m, 2H), 6.77 (ddd, *J* = 8.3, 2.4, 0.6 Hz, 1H), 3.88 – 3.78 (m, 4H), 3.17 – 3.08 (m, 4H). ^13^C NMR (101 MHz, CDCl_3_) δ 152.36, 135.06, 130.10, 119.70, 115.52, 113.60, 66.74, 48.89. HRMS (CI+) *m/z*: calculated for C_10_H_13_^35^ClNO [M+H]^+^: 198.0680; found: 198.0678 (Diff: 1.01 ppm).

**4-(3-fluorophenyl)morpholine (3e)** Prepared according to the general procedure for **3b** using 1-fluoro-3-iodobenzene (1 eq.) and morpholine (1 eq.) as a colorless oily liquid in a 73 % yield (1.22 g). The product was purified using flash column chromatography on silica gel using 10 % EtOAc:hexane as an eluent system. ^1^H NMR (400 MHz, CDCl_3_) δ 7.26 – 7.11 (m, 1H), 6.65 (dd, *J* = 8.2, 2.1 Hz, 1H), 6.59 – 6.50 (m, 2H), 3.88 – 3.80 (m, 4H), 3.17 – 3.10 (m, 4H). ^13^C NMR (101 MHz, CDCl_3_) δ 163.89 (d, *J* = 243.5 Hz), 153.00 (d, *J* = 9.5 Hz), 130.21 (d, *J* = 10.0 Hz), 110.80 (d, *J* = 2.6 Hz), 106.21 (d, *J* = 21.4 Hz), 102.41 (d, *J* = 25.3 Hz), 66.73, 48.82. HRMS (CI+) *m/z*: calculated for C_10_H_13_FNO [M+H]^+^: 182.0976; found: 182.0977 (Diff: -0.55 ppm).

**4-(3-methoxyphenyl)morpholine (3f)** prepared according to the general procedure for **3b** using 3-bromoanisole (1 eq.) and morpholine (1 eq.) as light pink crystals upon cooling in a 78 % yield (0.30 g). The product was purified using flash column chromatography on silica gel using 30 % EtOAc:hexane as an eluent system. ^1^H NMR (400 MHz, CDCl_3_) δ 7.21 – 7.15 (m, 1H), 6.65 – 6.45 (m, 1H), 6.46 – 6.42 (m, 2H), 3.91 – 3.81 (m, 4H), 3.79 (s, 3H), 3.25 – 3.07 (m, 4H). ^13^C NMR (101 MHz, CDCl_3_) δ 160.67, 152.73, 129.88, 108.48, 104.74, 102.24, 66.90, 55.20, 49.30. HRMS (CI+) *m/z*: calculated for C_11_H_16_NO_2_ [M+H]^+^: 194.1176; found: 194.1177 (Diff: -0.52 ppm).

**(*S*)-3-methyl-4-phenylmorpholine (3g)** prepared according to the general procedure for **3b** using iodobenzene (1 eq.) and (*S*)-3-methyl morpholine (1 eq.) as a colorless oily liquid in a 45-64 % yield (0.33-0.45 g). The product was purified using flash column chromatography on silica gel using 20 % EtOAc:hexane as an eluent system. ^1^H NMR (400 MHz, CDCl_3_) δ 7.30 – 7.21 (m, 2H), 6.94 – 6.84 (m, 3H), 3.97 (dt, *J* = 11.2, 3.4 Hz, 1H), 3.89 – 3.83 (m, 1H), 3.77 – 3.64 (m, 3H), 3.21 – 3.01 (m, 2H), 1.07 (d, *J* = 6.5 Hz, 3H). ^13^C NMR (101 MHz, CDCl_3_) δ 149.99, 129.20, 119.85, 116.77, 72.06, 67.22, 51.03, 44.49, 11.67. HRMS (ES+) *m/z*: calculated for C_11_H_16_NO [M+H]^+^: 178.1226; found: 178.1227 (Diff: -0.56 ppm).

**(*R*)-3-methyl-4-phenylmorpholine (3h)** prepared according to the general procedure for **3b** using iodobenzene (1 eq.) and (*R*)-3-methyl morpholine (1 eq.) as a colourless oily liquid in a 45-79 % yield (0.12-0.55 g). The product was purified using flash column chromatography on silica gel using 20 % EtOAc:hexane as an eluent system. ^1^H NMR (400 MHz, CDCl_3_) δ 7.36 – 7.17 (m, 2H), 6.99 – 6.77 (m, 3H), 3.97 (dt, *J* = 11.2, 3.2 Hz, 1H), 3.90 – 3.82 (m, 1H), 3.78 – 3.62 (m, 3H), 3.25 – 3.00 (m, 2H), 1.07 (d, *J* = 6.4 Hz, 3H). ^13^C NMR (101 MHz, CDCl_3_) δ 149.80, 129.20, 119.86, 116.77, 72.06, 67.22, 51.03, 44.50, 11.67. HRMS (ES+) *m/z*: calculated for C_11_H_16_NO [M+H]^+^: 178.1226; found: 178.1226 (Diff: 0.32 ppm).

**4-phenyl-3-propylmorpholine (3i)** prepared according to the general procedure for **3b** using iodobenzene (1 eq.) and 3-propyl morpholine (1 eq.). **7c** was collected as a pink oil in a 36 % yield (0.113 g). The crude product was purified using flash column chromatography on silica gel using 10 % EtOAc:hexane as an eluent system. ^1^H NMR (400 MHz, CDCl_3_) δ 7.32 – 7.18 (m, 2H), 6.87 – 6.80 (m, 3H), 3.96 (dt, *J* = 10.9, 2.7 Hz, 1H), 3.89 (dd, *J* = 11.3, 1.1 Hz, 1H), 3.77 (dd, *J* = 11.3, 2.7 Hz, 1H), 3.74 – 3.66 (m, 1H), 3.58 – 3.47 (m, 1H), 3.23 – 3.07 (m, 2H), 1.85 – 1.73 (m, 1H), 1.43 – 1.30 (m, 2H), 1.29 – 1.12 (m, 1H), 0.87 (t, *J* = 7.0 Hz, 3H). ^13^C NMR (101 MHz, CDCl_3_) δ 149.87, 129.25, 118.99, 115.59, 68.68, 66.99, 55.42, 43.56, 27.28, 20.10, 14.07.

**2,6-dimethyl-4-phenylmorpholine (3j)** prepared according to the general procedure for **3b** using iodobenzene (1 eq.) and 2,6-dimethylmorpholine (1 eq.). **3j** was collected as a mixture of *cis* and *trans* isomers as a dark red oil in a 75 % yield (0.25 g). The crude product was purified using flash column chromatography on silica gel using 5 % EtOAc:hexane as an eluent system. ^1^H NMR (400 MHz, CDCl_3_) δ 7.31 – 7.22 (m, 3H), 6.95 – 6.81 (m, 4H), 4.21 – 4.03 (m, 1H), 3.90 – 3.73 (m, 2H), 3.48 – 3.42 (m, 2H), 3.20 (dd, *J* = 11.7, 3.2 Hz, 1H), 2.88 (dd, *J* = 11.5, 6.0 Hz, 1H), 2.41 (dd, *J* = 11.7, 10.7 Hz, 2H), 1.31 (d, *J* = 6.4 Hz, 2H), 1.26 (d, *J* = 6.3 Hz, 3H). ^13^C NMR (101 MHz, CDCl_3_) δ 151.71, 150.99, 129.19, 119.79, 115.89, 115.80, 71.67, 66.49, 54.88, 54.33, 19.09, 18.08. (NMR Shows the 2 isomers). HRMS (ES+) *m/z*: calculated for C_12_H_18_NO [M+H]^+^ 192.1383; found: 192.1388 (Diff: -2.60 ppm).

***Cis* 2,6-dimethyl-4-phenylmorpholine (3k)** prepared according to the general procedure for **3b** using iodobenzene (1 eq.) and *cis* 2,6-dimethylmorpholine (1 eq.) as a colorless oil in an 81-88 % yield (0.65-0.67 g). The crude product was purified using flash column chromatography on silica gel using 5 % EtOAc:hexane as an eluent system. ^1^H NMR (400 MHz, CDCl_3_) δ 7.31 (t, *J* = 7.9 Hz, 2H), 6.95 (d, *J* = 7.9 Hz, 2H), 6.90 (t, *J* = 7.3 Hz, 1H), 3.91 – 3.80 (m, 2H), 3.49 (d, *J* = 11.5 Hz, 2H), 2.45 (t, *J* = 11.5 Hz, 2H), 1.30 (d, *J* = 6.3 Hz, 6H). ^13^C NMR (101 MHz, CDCl_3_) δ 151.01, 129.20, 119.80, 115.81, 71.68, 54.89, 19.11. HRMS (ES+) *m/z*: calculated for C_12_H_18_NO [M+H]^+^ 192.1383; found: 192.1390 (Diff: -3.64 ppm).

***Trans* (*2S*, *6S*)-dimethyl-4-phenylmorpholine (3l)** was prepared according to the general procedure for **3b** using iodobenzene (1 eq.) and *Trans* (2*S*,6*S*)-dimethylmorpholine (1 eq.) as a yellow oil in an 87 % yield (0.58 g). The crude product was purified using flash column chromatography on silica gel using 5 % EtOAc:hexane as an eluent system. ^1^H NMR (400 MHz, CDCl_3_) δ 7.18 (t, *J* = 7.9 Hz, 2H), 6.78 (dd, *J* = 16.8, 7.9 Hz, 3H), 4.13 – 4.00 (m, 2H), 3.12 (dd, *J* = 11.7, 2.9 Hz, 2H), 2.80 (dd, *J* = 11.7, 6.3 Hz, 2H), 1.23 (d, *J* = 6.3 Hz, 6H). ^13^C NMR (101 MHz, CDCl_3_) δ 151.72, 129.18, 119.60, 115.90, 66.50, 54.34, 18.08.

***Trans* (*2R*, *6R*)-dimethyl-4-phenylmorpholine (3m)** was prepared according to the general procedure for **3b** using iodobenzene (1 eq.) and *Trans* (2*R*,6*R*)-dimethylmorpholine (1 eq.) as a dark pink oil in a 92 % yield (0.76 g). The crude product was purified using flash column chromatography on silica gel using 5 % EtOAc:hexane as an eluent system. ^1^H NMR (400 MHz, CDCl_3_) δ 7.31 (t, *J* = 7.9 Hz, 2H), 6.91 (dd, *J* = 16.8, 7.9 Hz, 3H), 4.28 – 4.11 (m, 2H), 3.24 (dd, *J* = 11.7, 2.9 Hz, 2H), 2.92 (dd, *J* = 11.7, 6.3 Hz, 2H), 1.36 (d, *J* = 6.3 Hz, 6H). ^13^C NMR (101 MHz, CDCl_3_) δ 151.72, 129.18, 119.61, 115.91, 66.50, 54.34, 18.09.

**4-fluoro-1-phenylpiperidine (3n)** was prepared according to the general procedure for **3b** using iodobenzene (1 eq.), 4-fluoropiperidine hydrochloride (1 eq.) and NaO*t*Bu (2.2 eq.) as a colorless oily liquid in a 72 % yield (0.37 g). The product was purified using flash column chromatography on silica gel using 10 % EtOAc:hexane as an eluent system. ^1^H NMR (400 MHz, CDCl_3_) δ 7.32 – 7.19 (m, 2H), 6.95 (d, *J* = 8.0 Hz, 2H), 6.85 (t, *J* = 7.3 Hz, 1H), 4.86 (tt, *J* = 6.7, 3.5 Hz, 0.5H), 4.78 – 4.62 (m, 0.5H), 3.42 – 3.32 (m, 2H), 3.22 – 3.06 (m, 2H), 2.16 – 1.85 (m, 4H). ^13^C NMR (101 MHz, CDCl_3_) δ 45.99 (d, *J* = 6.2 Hz), 31.18 (d, *J* = 19.4 Hz). ^13^C NMR (101 MHz, CDCl_3_) δ 151.18, 129.17, 119.72, 116.65, 88.45 (d, *J* = 171.0 Hz), 45.99 (d, *J* = 6.2 Hz), 31.18 (d, *J* = 19.4 Hz).

**4,4-difluoro-1-phenylpiperidine (3o)** was prepared according to the general procedure for **3b** using iodobenzene (1 eq.), 4,4-difluoropiperidine hydrochloride (1 eq.) and NaO*t*Bu (2.2 eq) as a pink-orange oily liquid in a 61 % yield (0.15 g). The crude product was purified using flash column chromatography on silica gel using 5 % EtOAc:hexane as an eluent system. ^1^H NMR (400 MHz, CDCl_3_) δ 7.29 – 7.23 (m, 2H), 6.96 – 6.90 (m, 2H), 6.90 – 6.83 (m, 1H), 3.35 – 3.29 (m, 4H), 2.13 – 2.01 (m, 4H). ^13^C NMR (101 MHz, CDCl_3_) δ 150.23, 129.32, 122.01, 120.19, 116.85, 46.78 (t, *J* = 5.2 Hz), 33.65 (t, *J* = 22.8 Hz).

**4-methoxy-1-phenylpiperidine (3p)** was prepared according to the general procedure for **3b** using iodobenzene (1 eq.) and 4-methoxypiperidine (1 eq.) as a colorless oily liquid in a 60 % yield (0.19 g). The product was purified using flash column chromatography on silica gel using 40 % EtOAc:hexane as an eluent system. ^1^H NMR (400 MHz, CDCl_3_) δ 7.36 – 7.14 (m, 2H), 6.96 – 6.92 (m, 2H), 6.85 – 6.80 (m, 1H), 3.60 – 3.41 (m, 2H), 3.38 (s, 3H), 3.37 – 3.30 (m, 1H), 2.92 (ddd, *J* = 12.6, 9.6, 3.1 Hz, 2H), 2.06 – 1.85 (m, 2H), 1.77 – 1.55 (m, 2H).^13^C NMR (101 MHz, CDCl_3_) δ 151.43, 129.07, 119.41, 116.53, 76.18, 55.58, 47.30, 30.68.

**(*1S, 4S*)-5-phenyl-2-oxa-5-azabicyclo[2.2.1]heptane (3q)** was prepared according to the general procedure for **3b** using iodobenzene (1 eq.), (1*S*, 4*S*)-2-oxa-5-azabicyclo[2.2.1]heptane hydrochloride (1 eq.) and NaO*t*Bu (2.2 eq) as faint orange crystals in a 31-45 % yield (0.12-0.178 g). The crude product was purified using flash column chromatography on silica gel using 20 % EtOAc:hexane as an eluent system that was increased to 30 % EtOAc:hexane. ^1^H NMR (400 MHz, CDCl_3_) δ 7.26 (t, *J* = 7.9 Hz, 2H), 6.75 (t, *J* = 7.3 Hz, 1H), 6.62 (d, *J* = 7.9 Hz, 2H), 4.67 (s, 1H), 4.43 (s, 1H), 3.96 (d, *J* = 7.3 Hz, 1H), 3.88 (d, *J* = 7.3 Hz, 1H), 3.59 (d, *J* = 9.2 Hz, 1H), 3.19 (d, *J* = 9.2 Hz, 1H), 2.05 (d, *J* = 9.6 Hz, 1H), 1.97 (d, *J* = 9.6 Hz, 1H). ^13^C NMR (101 MHz, CDCl_3_) δ 147.10, 129.28, 116.81, 112.92, 76.46, 71.85, 58.05, 57.08, 36.92. HRMS (ES+) *m/z*: calculated for C_11_H_14_NO [M+H]^+^: 176.1070; found: 176.1074 (Diff: -2.27 ppm).

**(*1R*, *4R*)-5-phenyl-2-oxa-5-azabicyclo[2.2.1]heptane (3r)** was prepared according to the general procedure for **3b** using iodobenzene (1 eq.), (1*R*, 4*R*)-2-oxa-5-azabicyclo[2.2.1]heptane hydrochloride (1 eq.) and NaO*t*Bu (2.2 eq) as yellow needle crystals in a 67-78 % yield (0.2-0.26 g). The crude product was purified using flash column chromatography on silica gel using 20 % EtOAc:hexane as an eluent system. ^1^H NMR (400 MHz, CDCl_3_) δ 7.26 (t, *J* = 7.9 Hz, 2H), 6.75 (t, *J* = 7.3 Hz, 1H), 6.62 (d, *J* = 7.9 Hz, 2H), 4.67 (s, 1H), 4.43 (s, 1H), 3.96 (d, *J* = 7.3 Hz, 1H), 3.88 (d, *J* = 7.3 Hz, 1H), 3.59 (d, *J* = 9.2 Hz, 1H), 3.20 (d, *J* = 9.2 Hz, 1H), 2.05 (d, *J* = 9.6 Hz, 1H), 1.98 (d, *J* = 9.6 Hz, 1H). ^13^C NMR (101 MHz, CDCl_3_) δ 147.10, 129.28, 116.82, 112.92, 76.46, 71.85, 58.06, 57.09, 36.92. HRMS (CI+) *m/z*: calculated for C_11_H_14_NO [M+H]^+^: 176.1070; found: 176.1077 (Diff: -3.97 ppm).

**3-phenyl-8-oxa-3-azabicyclo[3.2.1]octane (3s)** was prepared according to the general procedure for **3b** using iodobenzene (1 eq.), 8-oxa-3-azabicyclo[3.2.1]octane hydrochloride (1 eq.) and NaO*t*Bu (2.2 eq) as a dark pink oily liquid in a 48 % yield (0.12 g). The crude product was purified using flash column chromatography on silica gel using 20 % EtOAc:hexane as an eluent system. ^1^H NMR (400 MHz, CDCl_3_) δ 7.29 – 7.22 (m, 2H), 6.84 – 6.77 (m, 3H), 4.50 – 4.46 (m, 2H), 3.33 (d, *J* = 11.4 Hz, 2H), 3.01 (dd, *J* = 11.4, 2.4 Hz, 2H), 2.01 – 1.88 (m, 4H). ^13^C NMR (101 MHz, CDCl_3_) δ 151.07, 129.13, 118.68, 113.86, 74.08, 53.36, 28.30. The compound was patented by Peng *et* *al*.^30^

**Synthesis of intermediates 4a-s**

**General procedure A for the synthesis of analogues** **4a, 4c, 4e-s**

A mixture of derivatives **3b-t** (1.0 eq.) and **2b** (1.0 eq.) in dry toluene (13 mL) was allowed to heat to reflux at 80 °C for 3 hours. The reaction mixture was then diluted with toluene and distilled water. The insoluble black precipitate was filtered off, and the organic layer was washed with water (2x30 mL) and brine (30 mL). It was then dried over anhydrous MgSO_4_, filtered and concentrated *in vacuo*. The crude product was purified using flash column chromatography on silica gel to afford **4a, 4c, 4e-s**.

**General procedure B for the synthesis of analogues 4b and 4d**

Morpholine derivatives **3b** or **3d** (1.0 eq.) were added to a solution of **2b** (1.0 eq.) in anhydrous chloroform (7 mL). The mixture was cooled to 10 °C. Anhydrous AlCl_3_ (1.0 eq.) was added, and the suspension was allowed to warm to room temperature and allowed to stir for 3 hours. The reaction mixture was then poured onto crushed ice and extracted with chloroform (3x20 mL). Then, it was dried over anhydrous MgSO_4_, filtered and concentrated *in vacuo*. The crude product was purified by flash column chromatography on silica gel (10-30 % EtOAc in hexane) to afford **4b** and **4d.**

**3-chloro-4-(4-morpholinophenyl)cyclobut-3-ene-1,2-dione (4a).** Prepared following general procedure **A** using 4-phenyl morpholine (1.0 eq.) and **2b** (1.0 eq.) in dry toluene (13 mL) was allowed to heat to reflux at 80 °C for 3 hours. The reaction mixture was then diluted with toluene and distilled water. The insoluble black precipitate was filtered off and the organic layer was washed with water (2x30 mL) and brine (30 mL). It was then dried over anhydrous MgSO_4_, filtered and concentrated *in vacuo.* The crude product was purified using flash column chromatography on silica gel using 40% EtOAc:hexane as an eluent system to afford **4a** as a dark maroon solid in 21% yield (0.42 g). ^1^H NMR (400 MHz, CDCl_3_) δ 8.17 (d, *J* = 9.2 Hz, 1H), 6.95 (d, *J* = 9.2 Hz, 1H), 3.89 – 3.85 (m, 2H), 3.46 – 3.42 (m, 2H). ^13^C NMR (101 MHz, CDCl_3_) δ 195.51, 190.39, 186.60, 174.20, 155.02, 131.40, 116.53, 113.36, 66.34, 46.68. HRMS (ES+) *m/z*: calculated for C_14_H_13_^35^ClNO_3_ [M+H]^+^: 278.0578; found: 278.0578 (Diff: 0.32 ppm).

**3-chloro-4-(3-methyl-4-morpholinophenyl)cyclobut-3-ene-1,2-dione (4b).** Prepared according to general procedure **B** using **3b** (1 eq.) and **2b** (1 eq.). The crude product was purified using flash column chromatography on silica gel using 30% EtOAc:hexane as an eluent system to afford **4b** as a brown sticky solid in an 11% yield (0.09 g). ^1^H NMR (400 MHz, CDCl_3_) δ 8.28 – 7.89 (m, 2H), 7.10 (d, *J* = 8.4 Hz, 1H), 3.90 – 3.86 (m, 4H), 3.10 – 3.06 (m, 4H), 2.39 (s, 3H). ^13^C NMR (101 MHz, CDCl_3_) δ 195.10, 190.54, 157.78, 132.34, 128.53, 118.86, 77.32, 77.01, 76.69, 66.91, 51.24, 18.93.

**3-chloro-4-(2-methyl-4-morpholinophenyl)cyclobut-3-ene-1,2-dione (4c).** Prepared according to general procedure **A** using **2b** (1 eq.) and **3c** (1 eq.). The crude product was purified using flash column chromatography on silica gel using 50% EtOAc:hexane as an eluent system to afford **4c** as a dark orange solid in a 13% yield (0.12 g). ^1^H NMR (400 MHz, CDCl_3_) δ 7.98 (d, *J* = 8.7 Hz, 1H), 6.80 – 6.74 (m, 2H), 3.94 – 3.76 (m, 4H), 3.53 – 3.30 (m, 4H), 2.60 (s, 3H). ^13^C NMR (101 MHz, CDCl_3_) δ 194.25, 192.05, 191.24, 176.41, 154.52, 142.50, 131.96, 116.69, 115.85, 110.87, 77.33, 77.01, 76.69, 66.42, 46.86, 23.09. HRMS (ES+) *m/z*: calculated for C_15_H_15_^35^ClNO_3_ [M+H]^+^: 292.0735; found: 292.0733 (Diff: 0.68 ppm).

**3-chloro-4-(2-chloro-4-morpholinophenyl)cyclobut-3-ene-1,2-dione (4d).** Prepared according to general procedure **B** using **2b** (1 eq.) and **3d** (1 eq.). The crude product was purified using flash column chromatography on silica gel using 40% EtOAc:hexane as an eluent system to afford **4d** as an orange solid in a 10% yield (0.12 g). ^1^H NMR (400 MHz, CDCl_3_) δ 7.99 (d, *J* = 9.0 Hz, 1H), 6.97 (d, *J* = 2.6 Hz, 1H), 6.83 (dd, *J* = 9.0, 2.6 Hz, 1H), 3.89 – 3.80 (m, 4H), 3.44 – 3.32 (m, 4H).^13^C NMR (101 MHz, CDCl_3_) δ 168.54, 154.84, 136.96, 132.26, 115.15, 111.36, 66.26, 46.82. HRMS (ES+) *m/z*: calculated for C_14_H_12_^35^Cl_2_NO_3_ [M+H]^+^: 312.0189; found: 312.0191 (Diff: -0.64 ppm).

**3-chloro-4-(2-fluoro-4-morpholinophenyl)cyclobut-3-ene-1,2-dione (4e).** Prepared according to general procedure **A** using **2b** (1 eq.) and **3e** (1 eq.). The crude product was purified using flash column chromatography on silica gel using 40% EtOAc:hexane as an eluent system to afford **4e** as an orange solid in a 7-14 % yield (0.048- 0.06 g). ^1^H NMR (400 MHz, CDCl_3_) δ 8.25 (dd, *J* = 9.0, 8.0 Hz, 1H), 6.71 (dd, *J* = 9.0, 2.5 Hz, 1H), 6.63 – 6.57 (m, 1H), 3.89 – 3.84 (m, 4H), 3.47 – 3.40 (m, 4H). ^13^C NMR (101 MHz, CDCl_3_) δ 192.77 (d, *J* = 336.4 Hz), 184.10, 176.00, 164.24, 161.67, 156.98 (d, *J* = 11.5 Hz), 131.55 (d, *J* = 5.4 Hz), 106.28 (d, *J* = 21.4 Hz), 104.81 (d, *J* = 17.1 Hz), 100.28 (d, *J* = 25.4 Hz), 66.19, 46.74. HRMS (ES+) *m/z*: calculated for C_14_H_12_ClFNO_3_ [M+H]^+^: 292.0735; found: 292.0749 (Diff: -4.79 ppm). Anal. Calcd. for C_14_H_12_^35^ClFNO_3_: C, 56.87; H, 3.75; N, 4.74. Found C, 56.76; H, 3.90; N, 4.81.

**3-chloro-4-(2-methoxy-4-morpholinophenyl)cyclobut-3-ene-1,2-dione (4f).** Prepared according to general procedure **A** using **2b** (1 eq.) and **3f** (1 eq.). The crude product was purified using flash column chromatography on silica gel using 40% EtOAc:hexane as an eluent system to afford **4f** as an orange solid in a 16% yield (0.16 g). ^1^H NMR (400 MHz, CDCl_3_) δ 8.27 (d, *J* = 9.0 Hz, 1H), 6.51 (dd, *J* = 9.0, 2.3 Hz, 1H), 6.27 (d, *J* = 2.3 Hz, 1H), 3.93 (s, 3H), 3.89 – 3.84 (m, 4H), 3.51 – 3.39 (m, 4H). ^13^C NMR (101 MHz, CDCl_3_) δ 202.41, 195.79, 185.06, 170.26, 168.40, 161.34, 157.39, 132.48, 106.10, 96.20, 95.22, 66.34, 54.84, 46.96. HRMS (ES+) *m/z*: calculated for C_15_H_15_^35^ClNO_4_ [M+H]^+^: 308.0684; found: 308.0683 (Diff: 0.21 ppm).

**(*S*)-3-chloro-4-(4-(3-methylmorpholino)phenyl)cyclobut-3-ene-1,2-dione (4g).** Prepared according to general procedure **A** using **2b** (1 eq.) and **3g** (1 eq.). The crude product was purified using flash column chromatography on silica gel using 30-35 % EtOAc:hexane as an eluent system to afford **4g** as an orange solid in a 34-44 % yield (0.184-0.312 g). ^1^H NMR (400 MHz, CDCl_3_) δ 8.17 (d, *J* = 9.2 Hz, 2H), 6.91 (d, *J* = 9.2 Hz, 2H), 4.17 – 4.01 (m, 2H), 3.92 – 3.77 (m, 2H), 3.67 (td, *J* = 11.9, 3.6 Hz, 1H), 3.51 (dd, *J* = 12.6, 2.7 Hz, 1H), 3.33 (td, *J* = 12.6, 3.6 Hz, 1H), 1.30 (d, *J* = 6.8 Hz, 3H). ^13^C NMR (101 MHz, CDCl_3_) δ 195.59, 189.83, 186.66, 173.67, 154.09, 131.56, 115.93, 112.95, 70.99, 66.52, 49.14, 41.23, 12.91. HRMS (ES+) *m/z*: calculated for C_15_H_15_^35^ClNO_3_ [M+H]^+^: 292.0735; found: 292.0749 (Diff: -4.79 ppm).

**(*R*)-3-chloro-4-(4-(3-methylmorpholino)phenyl)cyclobut-3-ene-1,2-dione (4h).** Prepared according to general procedure **A** using **2b** (1 eq.) and **3h** (1 eq.). The crude product was purified using flash column chromatography on silica gel using 40% EtOAc:hexane as an eluent system to provide **4h** as a dark orange solid in a 10-32 % yield (0.03-0.26 g). ^1^H NMR (400 MHz, CDCl_3_) δ 8.16 (d, *J* = 9.0 Hz, 2H), 6.91 (d, *J* = 9.0 Hz, 2H), 4.24 – 3.92 (m, 2H), 3.88 – 3.78 (m, 2H), 3.72 – 3.62 (m, 1H), 3.57 – 3.46 (m, 1H), 3.38 – 3.28 (m, 1H), 1.30 (d, *J* = 6.7 Hz, 3H). ^13^C NMR (101 MHz, CDCl_3_) δ 206.92, 198.42, 195.64, 186.64, 173.62, 154.09, 131.55, 115.91, 112.94, 70.97, 66.51, 49.14, 41.23, 30.91, 12.91. HRMS (ES+) *m/z*: calculated for C_15_H_15_ClNO_3_ [M+H]^+^: 292.0735; found: 292.0737 (Diff: -0.68 ppm). Anal. Calcd. for C_15_H_14_^35^ClNO_3_: C, 61.76; H, 4.84; N, 4.80. Found C, 61.29; H, 4.93; N, 4.66.

**3-chloro-4-(4-(3-propylmorpholino)phenyl)cyclobut-3-ene-1,2-dione (4i).** Prepared according to general procedure **A** using **2b** (1 eq.) and **3i** (1 eq.). The crude product was purified using flash column chromatography on silica gel using 30% EtOAc:hexane as an eluent system to afford **4i** as a dark red solid in a 19% yield (0.033 g). ^1^H NMR (400 MHz, CDCl_3_) δ 8.16 (d, *J* = 9.2 Hz, 2H), 6.88 (d, *J* = 9.2 Hz, 2H), 4.04 (dd, *J* = 11.6, 3.9 Hz, 1H), 3.98 (d, *J* = 11.6 Hz, 1H), 3.82 (dd, *J* = 8.9, 4.3 Hz, 1H), 3.75 – 3.67 (m, 1H), 3.65 (dd, *J* = 11.6, 3.0 Hz, 1H), 3.54 (dd, *J* = 12.6, 3.0 Hz, 1H), 3.35 (td, *J* = 12.6, 3.9 Hz, 1H), 2.02 – 1.87 (m, 1H), 1.55 – 1.38 (m, 2H), 1.38 – 1.27 (m, 2H), 0.95 (t, *J* = 7.2 Hz, 3H). ^13^C NMR (101 MHz, CDCl_3_) δ 195.71, 190.29, 186.50, 173.34, 154.27, 131.63, 115.60, 112.83, 68.05, 66.47, 53.83, 41.75, 29.43, 19.92, 13.99. HRMS (ES+) *m/z*: calculated for C_17_H_19_^35^ClNO_3_ [M+H]^+^: 320.1048; found: 320.1063 (Diff -6.79 ppm).

**3-chloro-4-(4-(2,6-dimethylmorpholino)phenyl)cyclobut-3-ene-1,2-dione (4j).** Prepared according to general procedure **A** using **2b** (1 eq.) and **3j** (1 eq.) as 2 isomers the ***Cis* product** was prepared as an orange red solid in a 20% yield (0.054 g). The crude product was purified using flash column chromatography on silica gel using 30% EtOAc:hexane as an eluent system. ^1^H NMR (400 MHz, CDCl_3_) δ 8.15 (d, *J* = 9.2 Hz, 2H), 6.93 (d, *J* = 9.2 Hz, 2H), 3.81 – 3.70 (m, 4H), 2.71 – 2.60 (m, 2H), 1.30 (d, *J* = 6.1 Hz, 6H). ^13^C NMR (101 MHz, CDCl_3_) δ 195.35, 186.66, 173.61, 154.60, 131.47, 115.93, 113.35, 71.30, 51.92, 18.95. HRMS (ES+) *m/z*: calculated for C_16_H_17_^35^ClNO_3_ [M+H]^+^: 306.0891; found: 306.0893 (Diff -0.85 ppm)

The ***Trans* product** was collected as a red-orange solid in a 4% yield (0.01 g). ^1^H NMR (400 MHz, CDCl_3_) δ 8.15 (d, *J* = 9.2 Hz, 2H), 6.87 (d, *J* = 9.2 Hz, 2H), 4.19 (pd, *J* = 6.4, 3.4 Hz, 2H), 3.53 (dd, *J* = 12.7, 3.4 Hz, 2H), 3.25 (dd, *J* = 12.7, 7.1 Hz, 2H), 1.29 (d, *J* = 6.4 Hz, 6H). ^13^C NMR (101 MHz, CDCl_3_) δ 195.70, 190.06, 186.50, 173.13, 154.91, 131.59, 115.65, 112.77, 66.36, 51.27, 18.15. HRMS (ES+) *m/z*: calculated for C_16_H_17_^35^ClNO_3_ [M+H]^+^: 306.0891; found: 306.0895 (Diff: -1.30 ppm).

**3-chloro-4-(4-(*Cis*-2,6-dimethylmorpholino)phenyl)cyclobut-3-ene-1,2-dione (4k).** Prepared according to general procedure **A** using **2b** (1 eq.) and **3k** (1 eq.). The crude product was purified using flash column chromatography on silica gel using 30-100% EtOAc:hexane as an eluent system to afford **4k** as an orange-red solid in an 11-27 % yield (0.11-0.28 g). ^1^H NMR (400 MHz, CDCl_3_) δ 8.07 (d, *J* = 9.0 Hz, 2H), 6.85 (d, *J* = 9.0 Hz, 2H), 3.74 – 3.63 (m, 4H), 2.60 (t, *J* = 11.9 Hz, 2H), 1.23 (d, *J* = 6.1 Hz, 6H) (^1^H NMR shows 2 isomers). ^13^C NMR (101 MHz, CDCl_3_) δ 195.62, 190.29, 186.64, 173.75, 154.59, 131.46, 116.11, 113.32, 71.38, 71.29, 51.89, 19.00, 18.95. HRMS (ES+) *m/z*: calculated for C_16_H_17_^35^ClNO_3_ [M+H]^+^: 306.0891; found: 306.0892 (Diff: -0.32 ppm).

**3-chloro-4-(4-((*Trans*-2*S*,6*S*)-2,6-dimethylmorpholino)phenyl)cyclobut-3-ene-1,2-dione (4l).** Prepared according to general procedure **A** using **2b** (1 eq.) and **3l** (1 eq.). The crude product was purified using flash column chromatography on silica gel using 30% EtOAc:hexane as an eluent system to afford **4l** as an orange solid in a 37 % yield (0.39 g). ^1^H NMR (400 MHz, CDCl_3_) δ 8.16 (d, *J* = 9.1 Hz, 2H), 6.89 (d, *J* = 9.1 Hz, 2H), 4.27 – 4.14 (m, 2H), 3.55 (dd, *J* = 12.7, 3.1 Hz, 2H), 3.27 (dd, *J* = 12.7, 7.1 Hz, 2H), 1.32 (d, *J* = 6.4 Hz, 6H). ^13^C NMR (101 MHz, CDCl_3_) δ 195.69, 190.23, 186.47, 173.32, 154.90, 131.59, 115.62, 112.76, 66.36, 51.26, 18.16. HRMS (ES+) *m/z*: calculated for C_16_H_17_^35^ClNO_3_ [M+H]^+^: 306.0891; found: 306.0892 (Diff: -0.32 ppm).

**3-chloro-4-(4-((*Trans*-2*R*,6*R*)-2,6-dimethylmorpholino)phenyl)cyclobut-3-ene-1,2-dione (4m).** Prepared according to general procedure **A** using **2b** (1 eq.) and **3m** (1 eq.). The crude product was purified using flash column chromatography on silica gel using 30% EtOAc:hexane as an eluent system to afford **4m** as a dark orange solid in a 47 % yield (0.49 g). ^1^H NMR (400 MHz, CDCl_3_) δ 8.13 (d, *J* = 8.9 Hz, 2H), 6.87 (d, *J* = 8.9 Hz, 2H), 4.26 – 4.16 (m, 2H), 3.55 (dd, *J* = 12.7, 3.0 Hz, 2H), 3.26 (dd, *J* = 12.7, 7.1 Hz, 2H), 1.31 (d, *J* = 6.4 Hz, 6H). ^13^C NMR (101 MHz, CDCl_3_) δ 195.67, 190.21, 186.41, 173.19, 154.89, 131.57, 115.54, 112.73, 66.37, 51.23, 18.17. HRMS (ES+) *m/z*: calculated for C_16_H_17_^35^ClNO_3_ [M+H]^+^: 306.0891; found: 306.0891 (Diff: -0.01 ppm).

**3-chloro-4-(4-(4-fluoropiperidin-1-yl)phenyl)cyclobut-3-ene-1,2-dione (4n).** Prepared according to general procedure **A** using **2b** (1 eq.) and **3n** (1 eq.). The crude product was purified using flash column chromatography on silica gel using 30-35 % EtOAc:hexane as an eluent system to afford **4n** as an orange solid in a 16-42 % yield (0.1-0.11 g). ^1^H NMR (400 MHz, CDCl_3_) δ 8.13 (d, *J* = 9.2 Hz, 2H), 6.95 (d, *J* = 9.2 Hz, 2H), 5.07 – 4.96 (m, 0.5H), 4.92 – 4.78 (m, 0.5H), 3.68 – 3.54 (m, 4H), 2.07 – 1.87 (m, 4H). ^13^C NMR (101 MHz, CDCl_3_) δ 195.68, 190.26, 186.51, 173.38, 154.33, 131.65, 115.65, 113.42, 87.32 (d, *J* = 171.8 Hz), 43.10 (d, *J* = 4.9 Hz), 30.60 (d, *J* = 20.3 Hz). HRMS (ES+) *m/z*: calculated for C_15_H_14_^35^ClFNO_2_ [M+H]^+^: 294.0692; found: 294.0691 (Diff: 0.01 ppm).

**3-chloro-4-(4-(4,4-difluoropiperidin-1-yl)phenyl)cyclobut-3-ene-1,2-dione (4o).** Prepared according to general procedure **A** using **2b** (1 eq.) and **3o** (1 eq.) and the reaction mixture was allowed to heat for 6 hours. The crude product was purified using flash column chromatography on silica gel using 30% EtOAc:hexane as an eluent system to afford **4o** as a light orange solid in a 22-28% yield (0.14-0.35 g). ^1^H NMR (400 MHz, CDCl_3_) δ 8.17 (d, *J* = 9.0 Hz, 2H), 6.98 (d, *J* = 9.0 Hz, 2H), 3.73 – 3.56 (m, 4H), 2.11 (ddd, *J* = 19.2, 13.2, 5.7 Hz, 4H). ^13^C NMR (101 MHz, CDCl_3_) δ 195.47, 190.31, 186.72, 174.39, 153.71, 131.61, 121.26, 116.46, 113.88, 44.25 (t, *J* = 5.3 Hz), 33.45 (t, *J* = 23.5 Hz). HRMS (ES+) *m/z*: calculated for C_15_H_13_^35^ClF_2_NO_2_ [M+H]^+^: 312.0597; found: 312.0596 (Diff: 0.41 ppm).

**3-chloro-4-(4-(4-methoxypiperidin-1-yl)phenyl)cyclobut-3-ene-1,2-dione (4p).** Prepared according to general procedure **A** using **2b** (1 eq.) and **3p** (1 eq.). The crude product was purified using flash column chromatography on silica gel using 40% EtOAc:hexane as an eluent system to afford **4p** as an orange solid in a 28% yield (0.085 g). ^1^H NMR (400 MHz, CDCl_3_) δ 8.13 (d, *J* = 9.2 Hz, 2H), 6.93 (d, *J* = 9.2 Hz, 2H), 3.85 – 3.64 (m, 2H), 3.56 – 3.48 (m, 1H), 3.40 (s, 3H), 3.39 – 3.31 (m, 4H), 2.06 – 1.90 (m, 4H). ^13^C NMR (101 MHz, CDCl_3_) δ 190.24, 186.41, 172.96, 154.56, 131.65, 115.22, 113.29, 74.96, 55.84, 44.12, 30.00.

**3-(4-((1*S*,4*S*)-2-oxa-5-azabicyclo[2.2.1]heptan-5-yl)phenyl)-4-chloro cyclobut-3-ene-1,2-dione (4q).** Prepared according to general procedure **A** using **2b** (1 eq.) and **3q** (1 eq.). The crude product was purified using flash column chromatography on silica gel using 50-70 % EtOAc:hexane as an eluent system to afford **4q** as an orange solid in a 41-61% yield (0.11-0.12 g). ^1^H NMR (400 MHz, CDCl_3_) δ 8.15 (d, *J* = 8.7 Hz, 2H), 6.67 (d, *J* = 8.7 Hz, 2H), 4.79 (s, 1H), 4.65 (s, 1H), 3.96 (d, *J* = 7.6 Hz, 1H), 3.90 (d, *J* = 7.6 Hz, 1H), 3.61 (d, *J* = 9.9 Hz, 1H), 3.40 (d, *J* = 9.9 Hz, 1H), 2.13 – 2.05 (m, 2H). ^13^C NMR (101 MHz, CDCl_3_) δ 195.84, 190.10, 186.35, 172.46, 151.54, 131.78, 114.99, 112.29, 76.04, 72.96, 57.68, 57.41, 36.93. HRMS (ES+) *m/z*: calculated for C_15_H_12_^35^ClNO_3_Na [M+Na]^+^: 312.0398; found: 312.0395 (Diff: 0.96 ppm).

**3-(4-((1*R*,4*R*)-2-oxa-5-azabicyclo[2.2.1]heptan-5-yl)phenyl)-4-chloro cyclobut-3-ene-1,2-dione (4r).** Prepared according to general procedure **A** using **2** b(1 eq.) and **3r** (1 eq.). The crude product was purified using flash column chromatography on silica gel using 40% EtOAc:hexane as an eluent system to afford **4r** as an orange-red solid in a 39-48% yield (0.13-0.2 g). ^1^H NMR (400 MHz, CDCl_3_) δ 8.14 (d, *J* = 9.0 Hz, 2H), 6.65 (d, *J* = 9.0 Hz, 2H), 4.77 (s, 1H), 4.65 – 4.61 (m, 1H), 3.94 (dd, *J* = 7.6, 1.4 Hz, 1H), 3.88 (d, *J* = 7.6 Hz, 1H), 3.59 (dd, *J* = 9.9, 1.4 Hz, 1H), 3.40 – 3.36 (m, 1H), 2.08 – 2.05 (m, 2H). ^13^C NMR (101 MHz, CDCl_3_) δ 195.84, 190.10, 186.38, 172.50, 151.54, 131.78, 128.98, 115.02, 112.29, 76.03, 72.96, 57.67, 57.41, 36.93. HRMS (ES+) *m/z*: calculated for C_15_H_12_^35^ClNO_3_Na [M+Na]^+^: 312.0398; found: 312.0396 (Diff: 0.64 ppm).

**3-(4-(8-oxa-3-azabicyclo[3.2.1]octan-3-yl)phenyl)-4-chlorocyclobut-3-ene-1,2-dione (4s).** Prepared according to general procedure **A** using **2b** (1 eq.) and **3s** (1 eq.). The crude product was purified using flash column chromatography on silica gel using 40% EtOAc:hexane as an eluent system to afford **4s** as an orange solid in a 32% yield (0.06 g). ^1^H NMR (400 MHz, CDCl_3_) δ 8.15 (d, *J* = 9.1 Hz, 2H), 6.87 (d, *J* = 9.1 Hz, 2H), 4.56 (d, *J* = 2.6 Hz, 2H), 3.54 (d, *J* = 12.0 Hz, 2H), 3.25 (dd, *J* = 12.0, 2.6 Hz, 2H), 2.07 – 2.01 (m, 2H), 1.91 – 1.83 (m, 2H). ^13^C NMR (101 MHz, CDCl_3_) δ 195.63, 190.27, 186.71, 173.73, 155.72, 131.32, 116.08, 112.55, 73.34, 51.86, 28.07. HRMS (ES+) *m/z*: calculated for C_16_H_14_^35^ClNO_3_Na [M+Na]^+^: 326.0554; found: 326.0554 (Diff: 0.12 ppm).

**Synthesis of derivatives 8-10 and 16**

**Synthesis of 4-hydroxy-2,3-diisopropoxy-4-phenylcyclobut-2-en-1-one (8).** A solution of diisopropyl squarate (1.08 mmol, 1 eq.) under N_2_ in 3 mL of anhydrous THF was cooled to -78 °C and 0.1 mL (1eq.) of phenyl lithium **7** (1.9 M in dibutyl ether) was added dropwise. The reaction was kept at -78 °C and monitored by TLC for disappearance of starting material. After 1 hour, the reaction was quenched with 10 mL of H_2_O at -78 °C for 15 minutes. The mixture was diluted with 40 mL of ethyl acetate and the layers were separated. The aqueous layer was extracted with ethyl acetate (3X50 mL); the combined organic layers were dried over MgSO_4_, and the solvents were removed under vacuum to afford **8** as a colourless liquid in 68% yield (0.19 g) and was taken to the next step directly without further purification. ^1^H NMR (400 MHz, CDCl_3_) δ 7.55 – 7.51 (m, 2H), 7.37 (t, *J* = 7.4 Hz, 2H), 7.31 (d, *J* = 7.4 Hz, 1H), 5.52 (s, 1H), 4.98 – 4.85 (m, 1H), 1.87 – 1.83 (m, 12H). ^13^C NMR (101 MHz, CDCl_3_) δ 206.95, 183.70, 165.44, 137.70, 133.27, 128.48, 128.10, 125.83, 67.93, 53.42, 30.87, 25.58. HRMS (ES+) *m/z*: calculated for C_16_H_20_O_4_Na [M+Na]^+^: 299.1254; found: 299.1256 (Diff: -0.66 ppm).

**Synthesis of 3-isopropoxy-4-phenylcyclobut-3-ene-1,2-dione (9)**. 4-Hydroxy-2,3-diisopropoxy-4-phenylcyclobut-2-en-1-one (**8**) (0.1 g, 0.687 mmol) was dissolved in 5 mL of DCM at room temperature and 4 drops of concentrated HCl were added. After 1 hour, the reaction mixture was diluted with 10 mL of DCM and dried with MgSO_4_. The solvent was then evaporated under reduced pressure to give **9** as yellow crystals in 81% yield (0.12 g) and no further purification was required as observed from TLC and ^1^H NMR analysis. ^1^H NMR (400 MHz, CDCl_3_) δ 8.08 – 8.04 (m, 2H), 7.55 – 7.49 (m, 3H), 5.69 – 5.57 (m, 1H), 1.57 (d, *J* = 6.2 Hz, 6H). ^13^C NMR (101 MHz, CDCl_3_) δ 194.27, 192.85, 192.50, 174.02, 132.63, 129.09, 127.92, 127.67, 80.14, 23.02. The spectral data matches the reported compound data.

**Synthesis of 3-phenyl-4-((pyridin-2-ylmethyl)amino)cyclobut-3-ene-1,2-dione (10)**. A mixture of 3-isopropoxy-4-phenylcyclobut-3-ene-1,2-dione (**9**) (0.12 g, 0.55 mmol, 1 eq.) and 2-picolyl amine (0.057 mL, 0.55 mmol, 1 eq.) in methanol (3 mL) was allowed to stir for 30 min (monitored by TLC). Then it was poured into water (20 mL), the crude product was obtained as a light-yellow solid that was filtered off. The residue was air-dried to give the pure product **10** as a light-yellow solid in 41% yield (0.06 g) with no further purification required as observed from TLC and ^1^H NMR analysis. ^1^H NMR (400 MHz, DMSO) δ 9.66 (s, 1H), 8.56 (d, *J* = 4.8 Hz, 1H), 8.04 (d, *J* = 7.7 Hz, 2H), 7.83 (td, *J* = 7.7, 1.6 Hz, 1H), 7.59 – 7.50 (m, 3H), 7.47 (d, *J* = 7.8 Hz, 1H), 7.33 (dd, *J* = 7.2, 4.8 Hz, 1H), 5.03 (s, 2H). ^13^C NMR (101 MHz, DMSO) δ 200.49, 196.05, 189.37, 179.78, 162.12, 157.54, 149.75, 137.55, 131.05, 129.51, 126.65, 123.21, 122.15, 49.43. HRMS (ES+) *m/z*: calculated for C_16_H_13_N_2_O_2_ [M + H]^+^: 265.0972; found 265.0975 (Diff: -1.13 ppm). M.p.: 200-202 °C. Anal. Calcd. for C_16_H_12_N_2_O_2_: C, 72.72; H, 4.58; N, 10.60. Found C, 72.76; H, 4.50; N, 10.55.

**Synthesis of (4-methylpyridin-2-yl)methanamine (16).** NaBH_4_ (2.3 eq.) and 4-methylpicolinonitrile (1.0 eq.) were suspended in dry THF (3 mL/mmol) and cooled to 0 °C. Iodine (1.0 eq.) in dry THF (2 mL/mmol) was added under inert atmosphere at 0 °C for 2.5 hours. The reaction mixture was heated to reflux at 70 °C for 3 hours. It was then cooled to 0 °C, HCl (0.8 mL/mmol, 6M) was added slowly, and the contents were refluxed again for 30 minutes. The mixture was cooled to 0 °C and NaOH (7.5 eq.) was added. Diethyl ether (7 mL/mmol) was added, and the organic layer was separated. The amine left in the aqueous layer was then extracted with DCM (10x5 mL/mmol). The combined DCM extracts were dried over anhydrous MgSO4, filtered and concentrated *in vacuo* to afford **16** as a brown oil in a 34% yield (0.176 g). ^1^H NMR (400 MHz, DMSO) δ 8.38 (d, *J* = 5.0 Hz, 1H), 7.31 (s, 1H), 7.10 (d, *J* = 4.7 Hz, 1H), 3.82 (s, 2H), 2.36 (s, 33). ^13^C NMR (101 MHz, DMSO) δ 162.90, 148.82, 147.39, 122.89, 122.11, 47.59, 21.02. LRMS (CI) *m/z*: calculated for C7H11N2 [M+H]+: 123.2; found: 123.1.

**Synthesis of RHS derivatives 18a-g and 19a-g**

**Synthesis of derivatives 18a-g**

***tert-*butyl ((6-fluoropyridin-2-yl)methyl)carbamate (18a).** Di-*tert*-butyl dicarbonate (2.0 eq.) and NiCl_2_·6H_2_O (0.1 eq.) were added to a solution of 6-fluoropicolinonitrile (1.0 eq.) in dry methanol (30 mL) at 0 °C. NaBH_4_ (7.0 eq.) was then added in small portions over 30 minutes. The reaction mixture was allowed to warm to room temperature and allowed to stir for an hour. Diethylenetriamine (1.0 eq.) was added, and the mixture was allowed to stir for 30 minutes. It was then concentrated *in vacuo*, dissolved in Ethyl acetate (50 mL) and extracted with saturated NaHCO_3_ (2x50 mL). The organic layer was dried over anhydrous MgSO_4_, filtered and concentrated *in vacuo* to yield **18a** as a colourless oil in a 41-96% yield (0.30-0.35 g). The product was taken to the next step directly without further purification. ^1^H NMR (400 MHz, CDCl_3_) δ 7.80 – 7.71 (m, 1H), 7.17 (dd, *J* = 7.4, 2.0 Hz, 1H), 6.84 – 6.76 (m, 1H), 5.42 (s, 1H), 4.39 (d, *J* = 5.5 Hz, 2H), 1.46 (s, 9H). ^13^C NMR (101 MHz, CDCl_3_) δ 163.21 (d, *J* = 240.1 Hz), 155.90, 141.67 (d, *J* = 7.6 Hz), 118.69 (d, *J* = 2.6 Hz), 118.25 (d, *J* = 4.0 Hz), 107.76 (d, *J* = 36.6 Hz), 79.74, 45.11, 28.36. HRMS (ES+) *m/z*: calculated for C_11_H_15_FN_2_O_2_Na [M+Na]^+^: 249.101; found: 249.1009 (Diff: 0.40 ppm).

***tert*-butyl ((5-fluoropyridin-2-yl)methyl)carbamate (18b).** Prepared according to the general procedure for **18a** using 5-fluoropicolinonitrile (1 eq.) as a yellow oil in a 99% yield (0.35 g). The product was taken to the next step directly without further purification. ^1^H NMR (400 MHz, CDCl_3_) δ 8.39 (d, *J* = 2.2 Hz, 1H), 7.38 (td, *J* = 8.3, 2.2 Hz, 1H), 7.29 (dd, *J* = 8.3, 3.9 Hz, 1H), 5.49 (s, 1H), 4.42 (d, *J* = 5.0 Hz, 2H), 1.46 (s, 9H). ^13^C NMR (101 MHz, CDCl_3_) δ 171.12, 158.65 (d, *J* = 254.8 Hz), 153.67 (d, *J* = 3.2 Hz), 137.19 (d, *J* = 23.8 Hz), 123.51 (d, *J* = 18.5 Hz), 122.55 (d, *J* = 4.0 Hz), 79.63, 60.37, 28.37. HRMS (ES+) *m/z*: calculated for C_11_H_16_FN_2_O_2_ [M+H]^+^: 227.1190; found: 227.1192 (Diff: -0.88 ppm).

***tert*-butyl ((4-fluoropyridin-2-yl)methyl)carbamate (18c).** Prepared according to the general procedure for **18a** using 4-fluoropicolinonitrile (1 eq.) as a yellow oil in a 54% yield (0.20 g). The product and was taken to the next step directly without further purification. ^1^H NMR (400 MHz, CDCl_3_) δ 8.55 – 8.44 (m, 1H), 7.02 (d, *J* = 9.5 Hz, 1H), 6.97 – 6.86 (m, 1H), 5.48 (s, 1H), 4.44 (d, *J* = 4.9 Hz, 2H), 1.46 (s, 9H). ^13^C NMR (101 MHz, CDCl_3_) δ 169.17 (d, *J* = 262.9 Hz), 155.97 (d, *J* = 5.3 Hz), 151.54 (d, *J* = 7.1 Hz), 150.32, 110.28 (d, *J* = 16.6 Hz), 109.24 (d, *J* = 16.7 Hz), 79.79, 55.13, 28.38. HRMS (ES+) *m/z*: calculated for C_11_H_16_FN_2_O_2_ [M+H]^+^: 227.1190; found: 227.1195 (Diff: -2.20 ppm).

***tert*-butyl ((3-fluoropyridin-2-yl)methyl)carbamate (18d).** Prepared according to the general procedure for **18a** using 3-fluoropicolinonitrile (1 eq.) as a reddish yellow oil in 95% yield (0.35 g). The product was to the next step directly without further purification. ^1^H NMR (400 MHz, CDCl_3_) δ 8.36 (d, *J* = 3.8 Hz, 1H), 7.38 (t, *J* = 8.8 Hz, 1H), 7.26 – 7.20 (m, 1H), 5.76 (s, 1H), 4.53 (s, 2H), 1.47 (s, 9H). ^13^C NMR (101 MHz, CDCl_3_) δ 161.26 (d, *J* = 270.3 Hz), 155.64, 145.15 (d, *J* = 15.6 Hz), 144.61 (d, *J* = 5.3 Hz), 123.48 (d, *J* = 3.6 Hz), 122.69 (d, *J* = 18.1 Hz), 79.49, 39.98, 28.38.

***tert*-butyl ((6-methoxypyridin-2-yl)methyl)carbamate (18e).** Prepared according to the general procedure for **18a** using 6-methoxypicolinonitrile (1 eq.) to as a yellow oil in 54% yield (0.19 g). The final product was taken to the next step directly without further purification. ^1^H NMR (400 MHz, CDCl_3_) δ 7.52 (t, *J* = 8.0 Hz, 1H), 6.81 (d, *J* = 7.1 Hz, 1H), 6.61 (d, *J* = 8.0 Hz, 1H), 5.40 (s, 1H), 4.34 (d, *J* = 4.1 Hz, 2H), 3.93 (s, 3H), 1.47 (s, 9H). ^13^C NMR (101 MHz, CDCl_3_) δ 163.75, 155.97, 155.20, 139.09, 113.82, 108.86, 79.38, 53.29, 28.40. HRMS (ES+) *m/z*: calculated for C_12_H_19_N_2_O_3_ [M+H]^+^: 239.1390; found: 239.1395 (Diff: -2.09 ppm).

***tert*-butyl ((6-chloropyridin-2-yl)methyl)carbamate (18f).** Prepared according to the general procedure for **18a** using 6-chloropicolinonitrile (1 eq.) as a yellow oil in 99% yield (0.35g). The product was taken to the next step directly without further purification. ^1^H NMR (400 MHz, CDCl_3_) δ 7.63 (t, *J* = 7.8 Hz, 1H), 7.28 – 7.18 (m, 2H), 5.40 (s, 1H), 4.40 (d, *J* = 5.7 Hz, 2H), 1.46 (s, 9H). ^13^C NMR (101 MHz, CDCl_3_) δ 158.90, 156.18, 150.90, 139.33, 122.76, 120.10, 79.78, 48.82, 28.38. HRMS (ES+) *m/z*: calculated for C_11_H_15_^35^ClN_2_O_2_Na [M+Na]^+^: 265.0714; found: 265.0721 (Diff: -2.64 ppm).

***tert*-butyl ((6-chloro-3-methylpyridin-2-yl)methyl)carbamate (18g).** Prepared according to the general procedure for **18a** using 6-chloro-3-methylpicolinonitrile (1 eq.) as a yellow oil in 71 % yield (0.24 g). The product was and was taken to the next step directly without further purification. ^1^H NMR (400 MHz, CDCl_3_) δ 7.42 (d, *J* = 7.9 Hz, 1H), 7.14 (d, *J* = 7.9 Hz, 1H), 5.90 (s, 1H), 4.38 (d, *J* = 4.3 Hz, 2H), 2.27 (s, 3H), 1.48 (s, 9H). ^13^C NMR (101 MHz, CDCl_3_) δ 155.96, 155.04, 147.84, 140.54, 129.40, 122.45, 79.52, 42.92, 28.42, 16.95.

**Synthesis of pyridine amine salts 19a-g**

**(6-fluoropyridin-2-yl)methanamine salt (19a).** Trifluoroacetic acid (4.0 eq.) was added to a solution of *tert-*butyl ((6-fluoropyridin-2-yl)methyl)carbamate **18a** (1.0 eq.) in dry DCM (5 mL) under nitrogen atmosphere. The reaction mixture was allowed to stir for 3 hours. It was then concentrated *in vacuo* to afford **19a** as a sticky amorphous orange-yellow solid in a 99% yield (0.4 g). The product was taken to the next step directly without further purification. ^1^H NMR (400 MHz, DMSO) δ 8.45 (s, 3H), 8.06 (q, *J* = 8.1 Hz, 1H), 7.45 (d, *J* = 7.1 Hz, 1H), 7.19 (d, *J* = 8.1 Hz, 1H), 4.20 (s, 2H). ^13^C NMR (101 MHz, DMSO) δ 162.98 (d, *J* = 238.2 Hz), 159.10 (q, *J* = 35.1 Hz), 152.86 (d, *J* = 12.8 Hz), 143.62 (d, *J* = 7.9 Hz), 120.97 (d, *J* = 4.0 Hz), 116.50 (q, *J* = 293.1 Hz), 109.40 (d, *J* = 36.2 Hz), 42.54. HRMS (ES+) *m/z*: calculated for C_6_H_8_FN_2_ [M]^+^: 127.0666; found: 127.0669 (Diff: -2.36 ppm).

**Synthesis of (5-fluoropyridin-2-yl)methanamine salt (19b).** Prepared according to the general procedure for **19a** using *tert*-butyl ((5-fluoropyridin-2-yl)methyl)carbamate **18b** (1 eq.) as a yellow oil in 99% yield (0.37 g). The product was taken to the next step directly without further purification. ^1^H NMR (400 MHz, DMSO) δ 8.64 (d, *J* = 2.4 Hz, 1H), 8.36 (s, 3H), 7.84 (td, *J* = 8.7, 2.4 Hz, 1H), 7.59 (dd, *J* = 8.7, 4.3 Hz, 1H), 4.21 (d, *J* = 4.9 Hz, 2H). ^13^C NMR (101 MHz, DMSO) δ 159.30 (d, *J* = 253.4 Hz), 158.86 (q, *J* = 35.2 Hz), 150.16 (d, *J* = 3.7 Hz), 137.44 (d, *J* = 24.1 Hz), 124.81 (d, *J* = 4.0 Hz), 124.64 (d, *J* = 4.3 Hz), 116.49 (q, *J* = 293.2 Hz), 42.68. HRMS (ES+) *m/z*: calculated for C_6_H_8_FN_2_ [M]^+^: 127.0666; found: 127.0666 (Diff: -0.16 ppm).

**(4-fluoropyridin-2-yl)methanamine salt (19c).** Prepared according to the general procedure for **19a** using *tert*-butyl ((4-fluoropyridin-2-yl)methyl)carbamate **18c** (1 eq.) as a yellow oil in a 100% yield (0.21 g). The product was taken to the next step directly without further purification. ^1^H NMR (400 MHz, DMSO) δ 8.70 – 8.64 (m, 1H), 8.35 (s, 3H), 7.46 (dd, *J* = 10.0, 2.0 Hz, 1H), 7.41 – 7.35 (m, 1H), 4.24 (d, *J* = 5.4 Hz, 2H). ^13^C NMR (101 MHz, DMSO) δ 172.01, 158.92 (q, *J* = 35.7 Hz), 152.38 (d, *J* = 7.7 Hz), 117.81, 114.90, 110.82, 109.48, 42.63. HRMS (ES+) *m/z*: calculated for C_6_H_8_FN_2_ [M]^+^: 127.0666; found: 127.0668 (Diff: -1.57 ppm).

**(3-fluoropyridin-2-yl)methanamine salt (19d).** Prepared according to the general procedure for **19a** using *tert*-butyl ((3-fluoropyridin-2-yl)methyl)carbamate **18d** (1 eq.) as an amorphous red solid in a 98% yield (0.47 g). The product was taken to the next step directly without further purification. ^1^H NMR (400 MHz, DMSO) δ 8.52 – 8.49 (m, 1H), 8.41 (s, 3H), 7.87 – 7.81 (m, 1H), 7.61 – 7.50 (m, 1H), 4.30 (s, 2H). ^13^C NMR (101 MHz, DMSO) δ 167.98, 158.40 (q, *J* = 44.0, 21.8 Hz), 145.47 (d, *J* = 5.2 Hz), 141.88 (d, *J* = 14.8 Hz), 125.85 (d, *J* = 3.9 Hz), 124.17 (d, *J* = 17.8 Hz), 115.73, 37.95. HRMS (ES+) *m/z*: calculated for C_6_H_8_FN_2_ [M]^+^: 127.0666; found: 127.0666 (Diff: -0.16 ppm).

**(6-methoxypyridin-2-yl)methanamine salt (19e).** Prepared according to the general procedure for **19a** using *tert*-butyl ((6-methoxypyridin-2-yl)methyl)carbamate **18e** (1 eq.) as a yellow oil in a 100% yield (0.20 g). The product was taken to the next step directly without further purification. ^1^H NMR (400 MHz, DMSO) δ 8.29 (s, 2H), 7.76 (t, *J* = 7.8 Hz, 1H), 7.05 (d, *J* = 7.3 Hz, 1H), 6.81 (d, *J* = 8.3 Hz, 1H), 4.12 (q, *J* = 5.6 Hz, 2H), 3.92 (s, 3H). ^13^C NMR (101 MHz, DMSO) δ 163.69, 158.94 (q, *J* = 35.2 Hz), 151.35, 140.54, 117.92, 115.58, 110.24, 53.85, 42.85. HRMS (ES+) *m/z*: calculated for C_7_H_11_N_2_O [M]^+^: 139.0866; found: 139.0867 (Diff: -0.71 ppm).

**(6-chloropyridin-2-yl)methanamine salt (19f).** Prepared according to the general procedure for **19a** using *tert*-butyl ((6-chloropyridin-2-yl)methyl)carbamate **18a** (1 eq.) as a sticky yellowish white solid in a 100% yield (0.58 g). The product was taken to the next step directly without further purification. ^1^H NMR (400 MHz, CDCl_3_) δ 8.18 (s, 3H), 7.74 (t, *J* = 7.9 Hz, 1H), 7.36 (d, *J* = 7.9 Hz, 1H), 7.28 – 7.24 (m, 1H), 4.37 (s, 2H). ^13^C NMR (101 MHz, CDCl_3_) δ 161.37, 160.98, 151.40, 140.37, 124.80, 120.92, 114.16, 42.73. HRMS (ES+) *m/z*: calculated for C_6_H_8_^35^Cl N_2_ [M]^+^: 143.037; found: 143.0371 (Diff: -0.4 ppm).

**(6-chloro-3-methylpyridin-2-yl)methanamine salt (19g).** Prepared according to the general procedure for **19a** using *tert*-butyl ((6-chloro-3-methylpyridin-2-yl)methyl)carbamate **18g** (1 eq.) as a sticky yellowish white solid in a 100% yield (0.25 g). The product was taken to the next step directly without further purification. ^1^H NMR (400 MHz, DMSO) δ 8.33 (s, 3H), 7.76 (d, *J* = 8.0 Hz, 1H), 7.46 (d, *J* = 8.0 Hz, 1H), 4.22 – 4.17 (m, 2H), 2.28 (s, 3H). ^13^C NMR (101 MHz, DMSO) δ 158.86 (q, *J* = 35.0 Hz), 152.91, 147.11, 142.08, 141.71, 131.00, 123.80, 43.48, 16.70. HRMS (ES+) *m/z*: calculated for C_7_H_10_^35^ClN_2_ [M]^+^: 157.0527; found: 157.0523 (Diff: 2.55 ppm).

1. **References**

(1) Topchiy, M. A.; Asachenko, A. F.; Nechaev, M. S. Solvent-Free Buchwald-Hartwig Reaction of Aryl and Heteroaryl Halides with Secondary Amines. *European J. Org. Chem.* **2014**, *2014* (16), 3319–3322. https://doi.org/10.1002/ejoc.201402077.

(2) Saavedra, J. Z.; Resendez, A.; Rovira, A.; Eagon, S.; Haddenham, D.; Singaram, B. Reaction of InCl 3 with Various Reducing Agents: InCl 3-NaBH 4-Mediated Reduction of Aromatic and Aliphatic Nitriles to Primary Amines. *Journal of Organic Chemistry* **2012**, *77* (1), 221–228. https://doi.org/10.1021/jo201809a.

(3) Caddick, S.; Judd, D. B.; Lewis, A. K. D. K.; Reich, M. T.; Williams, M. R. V. A Generic Approach for the Catalytic Reduction of Nitriles. *Tetrahedron* **2003**, *59* (29), 5417–5423. https://doi.org/10.1016/S0040-4020(03)00858-5.

(4) Caddick, S.; Alexandra, A. K.; Judd, D. B.; Williams, M. R. V. Convenient Synthesis of Protected Primary Amines Form Nitriles. *Tetrahedron Lett.* **2000**, *41* (18), 3513–3516. https://doi.org/10.1016/S0040-4039(00)00410-X.

(5) Claffey, M. M.; Helal, C. J.; Verhoest, P. R.; Kang, Z.; Fors, K. S.; Jung, S.; Zhong, J.; Bundesmann, M. W.; Hou, X.; Lui, S.; Kleiman, R. J.; Vanase-Frawley, M.; Schmidt, A. W.; Menniti, F.; Schmidt, C. J.; Hoffman, W. E.; Hajos, M.; McDowell, L.; Oconnor, R. E.; MacDougall-Murphy, M.; Fonseca, K. R.; Becker, S. L.; Nelson, F. R.; Liras, S. Application of Structure-Based Drug Design and Parallel Chemistry to Identify Selective, Brain Penetrant, in Vivo Active Phosphodiesterase 9A Inhibitors. *J. Med. Chem.* **2012**, *55* (21), 9055–9068. https://doi.org/10.1021/jm3009635.

(6) Waterhouse, A.; Bertoni, M.; Bienert, S.; Studer, G.; Tauriello, G.; Gumienny, R.; Heer, F. T.; De Beer, T. A. P.; Rempfer, C.; Bordoli, L.; Lepore, R.; Schwede, T. SWISS-MODEL: Homology Modelling of Protein Structures and Complexes. *Nucleic Acids Res.* **2018**, *46* (W1), W296–W303. https://doi.org/10.1093/nar/gky427.

(7) Courbon, G. M.; Palme, P. R.; Mann, L.; Richter, A.; Imming, P.; Rubinstein, J. L. Mechanism of Mycobacterial ATP Synthase Inhibition by Squaramides and Second Generation Diarylquinolines . *EMBO J.* **2023**, *42* (15), 1–12. https://doi.org/10.15252/embj.2023113687.

(8) Madeira, F.; Pearce, M.; Tivey, A. R. N.; Basutkar, P.; Lee, J.; Edbali, O.; Madhusoodanan, N.; Kolesnikov, A.; Lopez, R. Search and Sequence Analysis Tools Services from EMBL-EBI in 2022. *Nucleic Acids Res.* **2022**, *50* (W1), W276–W279. https://doi.org/10.1093/nar/gkac240.

(9) Studer, G.; Rempfer, C.; Waterhouse, A. M.; Gumienny, R.; Haas, J.; Schwede, T. QMEANDisCo—Distance Constraints Applied on Model Quality Estimation. *Bioinformatics* **2020**, *36* (6), 1765–1771. https://doi.org/10.1093/bioinformatics/btz828.

(10) Jones, G.; Willett, P.; Glen, R. C.; Leach, A. R.; Taylor, R. Development and Validation of a Genetic Algorithm for Flexible Docking. *J. Mol. Biol.* **1997**, *267* (3), 727–748. https://doi.org/10.1006/jmbi.1996.0897.

(11) Jämbeck, J. P. M.; Lyubartsev, A. P. An Extension and Further Validation of an All-Atomistic Force Field for Biological Membranes. *J. Chem. Theory Comput.* **2012**, *8* (8), 2938–2948. https://doi.org/10.1021/ct300342n.

(12) Allouche, A. Software News and Updates Gabedit — A Graphical User Interface for Computational Chemistry Softwares. *J. Comput. Chem.* **2012**, *32*, 174–182. https://doi.org/10.1002/jcc.

(13) Wu, E. L.; Cheng, X.; Jo, S.; Rui, H.; Song, K. C.; Dávila-Contreras, E. M.; Qi, Y.; Lee, J.; Monje-Galvan, V.; Venable, R. M.; Klauda, J. B.; Im, W. CHARMM-GUI Membrane Builder toward Realistic Biological Membrane Simulations. *J. Comput. Chem.* **2014**, *35* (27), 1997–2004. https://doi.org/10.1002/jcc.23702.

(14) Lindorff-Larsen, K.; Piana, S.; Palmo, K.; Maragakis, P.; Klepeis, J. L.; Dror, R. O.; Shaw, D. E. Improved Side-Chain Torsion Potentials for the Amber Ff99SB Protein Force Field. *Proteins: Structure, Function and Bioinformatics* **2010**, *78* (8), 1950–1958. https://doi.org/10.1002/prot.22711.

(15) Bussi, G.; Donadio, D.; Parrinello, M. Canonical Sampling through Velocity Rescaling. *J. Chem. Phys.* **2007**, *126* (1). https://doi.org/10.1063/1.2408420.

(16) Parrinello, M.; Rahman, A. Polymorphic Transitions in Single Crystals: A New Molecular Dynamics Method. *J. Appl. Phys.* **1981**, *52* (12), 7182–7190. https://doi.org/10.1063/1.328693.

(17) McGibbon, R. T.; Beauchamp, K. A.; Harrigan, M. P.; Klein, C.; Swails, J. M.; Hernández, C. X.; Schwantes, C. R.; Wang, L. P.; Lane, T. J.; Pande, V. S. MDTraj: A Modern Open Library for the Analysis of Molecular Dynamics Trajectories. *Biophys. J.* **2015**, *109* (8), 1528–1532. https://doi.org/10.1016/j.bpj.2015.08.015.

(18) Virtanen, P.; Gommers, R.; Oliphant, T. E.; Haberland, M.; Reddy, T.; Cournapeau, D.; Burovski, E.; Peterson, P.; Weckesser, W.; Bright, J.; van der Walt, S. J.; Brett, M.; Wilson, J.; Millman, K. J.; Mayorov, N.; Nelson, A. R. J.; Jones, E.; Kern, R.; Larson, E.; Carey, C. J.; Polat, İ.; Feng, Y.; Moore, E. W.; VanderPlas, J.; Laxalde, D.; Perktold, J.; Cimrman, R.; Henriksen, I.; Quintero, E. A.; Harris, C. R.; Archibald, A. M.; Ribeiro, A. H.; Pedregosa, F.; van Mulbregt, P.; Vijaykumar, A.; Bardelli, A. Pietro; Rothberg, A.; Hilboll, A.; Kloeckner, A.; Scopatz, A.; Lee, A.; Rokem, A.; Woods, C. N.; Fulton, C.; Masson, C.; Häggström, C.; Fitzgerald, C.; Nicholson, D. A.; Hagen, D. R.; Pasechnik, D. V.; Olivetti, E.; Martin, E.; Wieser, E.; Silva, F.; Lenders, F.; Wilhelm, F.; Young, G.; Price, G. A.; Ingold, G. L.; Allen, G. E.; Lee, G. R.; Audren, H.; Probst, I.; Dietrich, J. P.; Silterra, J.; Webber, J. T.; Slavič, J.; Nothman, J.; Buchner, J.; Kulick, J.; Schönberger, J. L.; de Miranda Cardoso, J. V.; Reimer, J.; Harrington, J.; Rodríguez, J. L. C.; Nunez-Iglesias, J.; Kuczynski, J.; Tritz, K.; Thoma, M.; Newville, M.; Kümmerer, M.; Bolingbroke, M.; Tartre, M.; Pak, M.; Smith, N. J.; Nowaczyk, N.; Shebanov, N.; Pavlyk, O.; Brodtkorb, P. A.; Lee, P.; McGibbon, R. T.; Feldbauer, R.; Lewis, S.; Tygier, S.; Sievert, S.; Vigna, S.; Peterson, S.; More, S.; Pudlik, T.; Oshima, T.; Pingel, T. J.; Robitaille, T. P.; Spura, T.; Jones, T. R.; Cera, T.; Leslie, T.; Zito, T.; Krauss, T.; Upadhyay, U.; Halchenko, Y. O.; Vázquez-Baeza, Y. SciPy 1.0: Fundamental Algorithms for Scientific Computing in Python. *Nat. Methods* **2020**, *17* (3), 261–272. https://doi.org/10.1038/s41592-019-0686-2.

(19) Salentin, S.; Schreiber, S.; Haupt, V. J.; Adasme, M. F.; Schroeder, M. PLIP: Fully Automated Protein-Ligand Interaction Profiler. *Nucleic Acids Res.* **2015**, *43* (W1), W443–W447. https://doi.org/10.1093/nar/gkv315.

(20) Baker, E. N.; Hubbard, R. E. Hydrogen Bonding in Globular Proteins. *Prog. Biophys. Mol. Biol.* **1984**, *44* (2), 97–179. https://doi.org/10.1016/0079-6107(84)90007-5.

(21) Homeyer, N.; Gohlke, H. Free Energy Calculations by the Molecular Mechanics Poisson-Boltzmann Surface Area Method. *Mol. Inform.* **2012**, *31* (2), 114–122. https://doi.org/10.1002/minf.201100135.

(22) Hou, T.; Wang, J.; Li, Y.; Wang, W. Assessing the Performance of the MM/PBSA and MM/GBSA Methods. 1. The Accuracy of Binding Free Energy Calculations Based on Molecular Dynamics Simulations. *J. Chem. Inf. Model.* **2011**, *51* (1), 69–82. https://doi.org/10.1021/ci100275a.

(23) Kumari, R.; Kumar, R.; Lynn, A. G-Mmpbsa -A GROMACS Tool for High-Throughput MM-PBSA Calculations. *J. Chem. Inf. Model.* **2014**, *54* (7), 1951–1962. https://doi.org/10.1021/ci500020m.

(24) *StarDrop, Optibrium, 2026*. https://www.optibrium.com/stardrop/.

(25) Pelkonen, O.; Turpeinen, M.; Hakkola, J.; Honkakoski, P.; Hukkanen, J.; Raunio, H. Inhibition and Induction of Human Cytochrome P450 Enzymes: Current Status. *Arch. Toxicol.* **2008**, *82* (10), 667–715. https://doi.org/10.1007/s00204-008-0332-8.

(26) Guengerich, F. P. Cytochrome P-450 3A4: Regulation and Role in Drug Metabolism. *Annu. Rev. Pharmacol. Toxicol.* **1999**, *39* (1), 1–17. https://doi.org/10.1146/annurev.pharmtox.39.1.1.

(27) Kourounakis, A. P.; Xanthopoulos, D.; Tzara, A. Morpholine as a Privileged Structure: A Review on the Medicinal Chemistry and Pharmacological Activity of Morpholine Containing Bioactive Molecules. *Med. Res. Rev.* **2020**, *40* (2), 709–752. https://doi.org/10.1002/med.21634.

(28) Pan, Y. The Dark Side of Fluorine. *ACS Med. Chem. Lett.* **2019**, *10* (7), 1016–1019. https://doi.org/10.1021/acsmedchemlett.9b00235.

(29) Xia, G.; Ruan, C.; Wang, H. Highly Sensitive Detection of Carbon Dioxide by a Pyrimido[1,2-a]Benzimidazole Derivative: Combining Experimental and Theoretical Studies. *Analyst* **2015**, *140* (15), 5099–5104. https://doi.org/10.1039/C5AN00947B.

(30) Li, Peng; Decampo, Floryan; Shi, Feng; Cui, Xinjiang; Yuan, H. Synthesis of Compounds Containing 8-Oxa-3-Azabicyclo[3.2.1]Octane Ring. WO2015109451, 2015.

1. **NMR spectra and HPLC chromatograms of target squaramide compounds**

**Figure S16.** ¹H NMR (400 MHz, DMSO), ^13^C NMR (101 MHz, DMSO) and HPLC chromatogram of compound **1f**


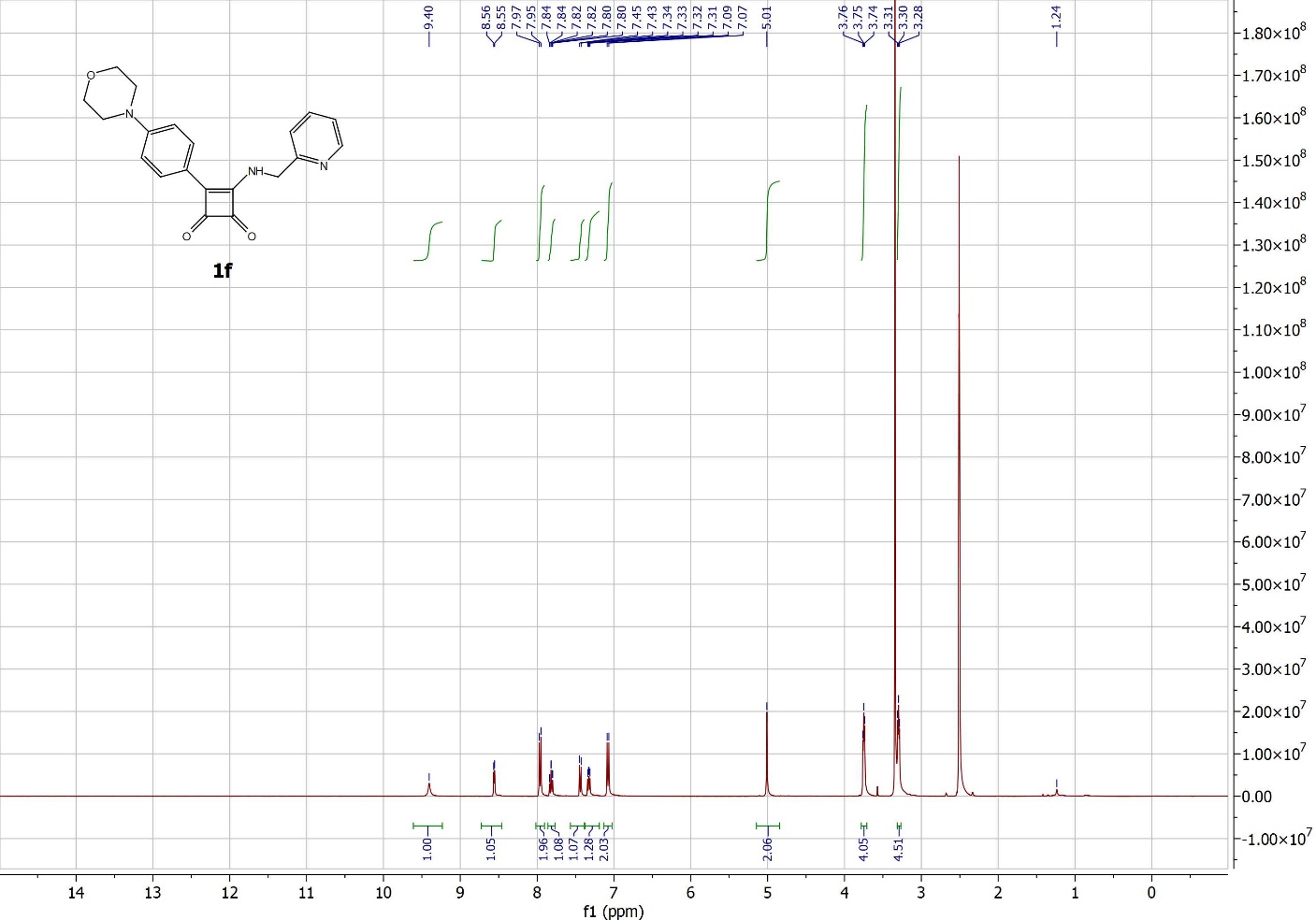


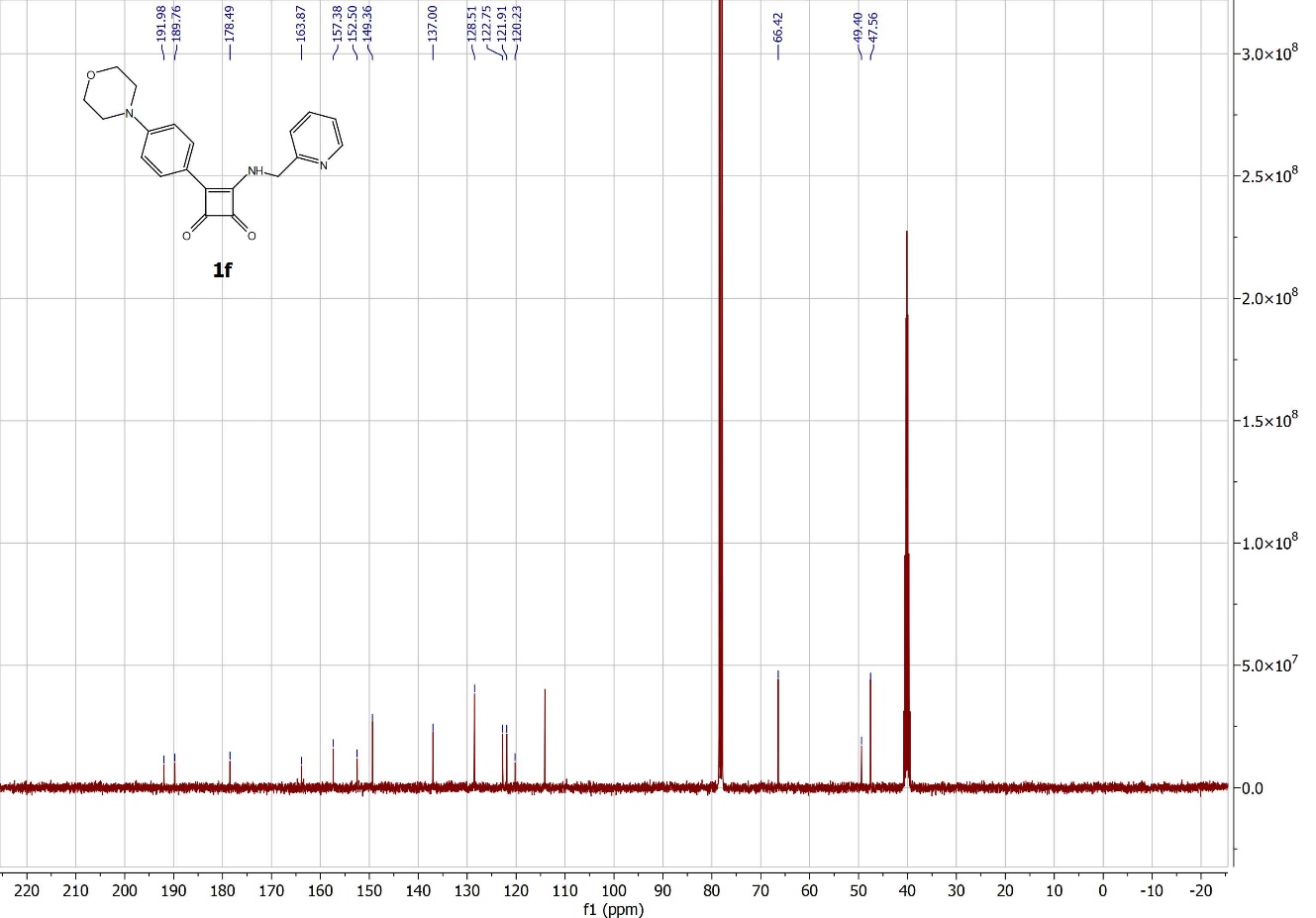


Sig= 254


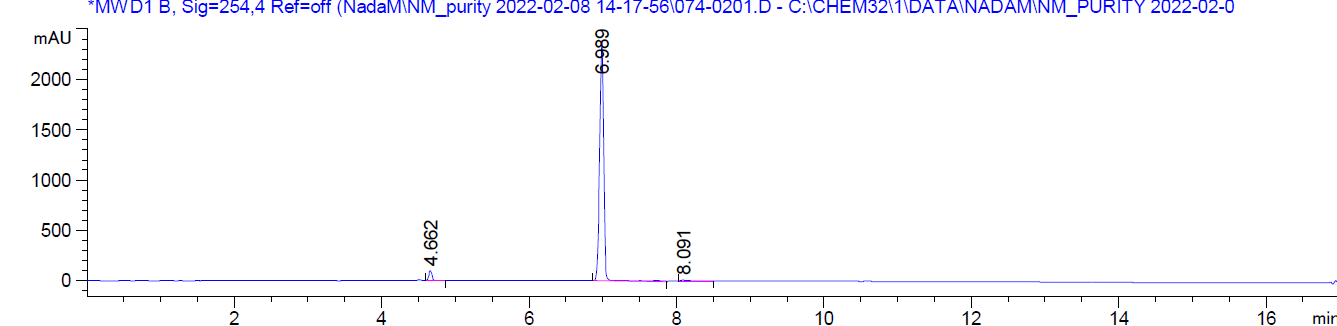


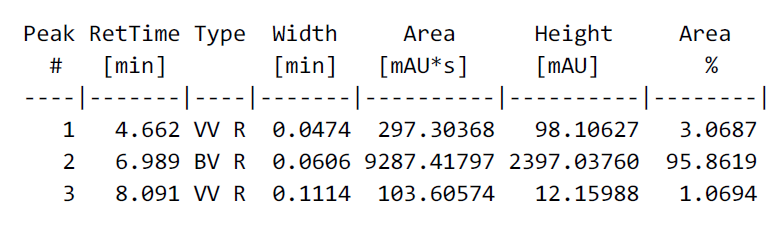


**Figure S17.** ¹H NMR (400 MHz, DMSO) and ^13^C NMR (101 MHz, DMSO) of compound **6b**


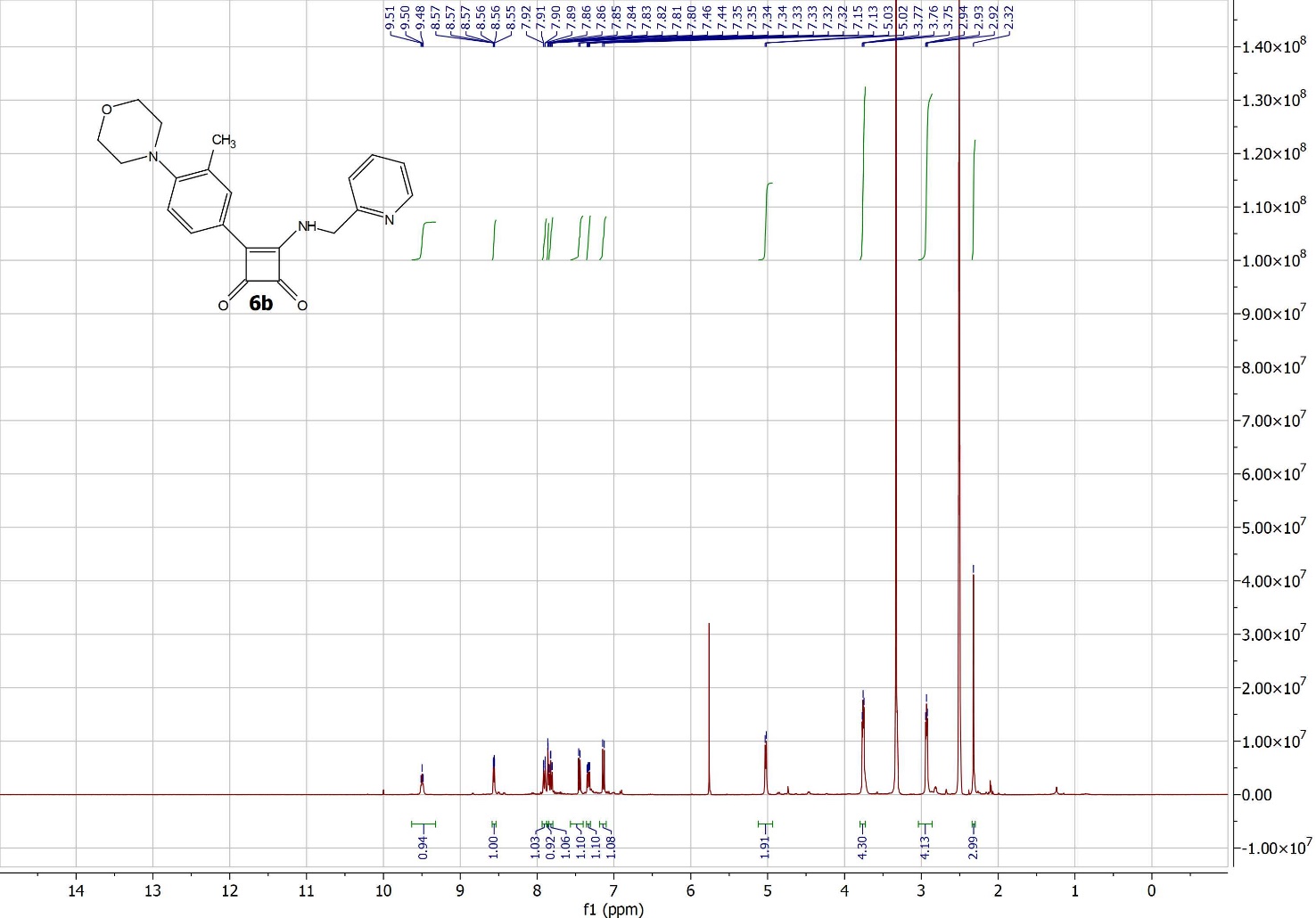

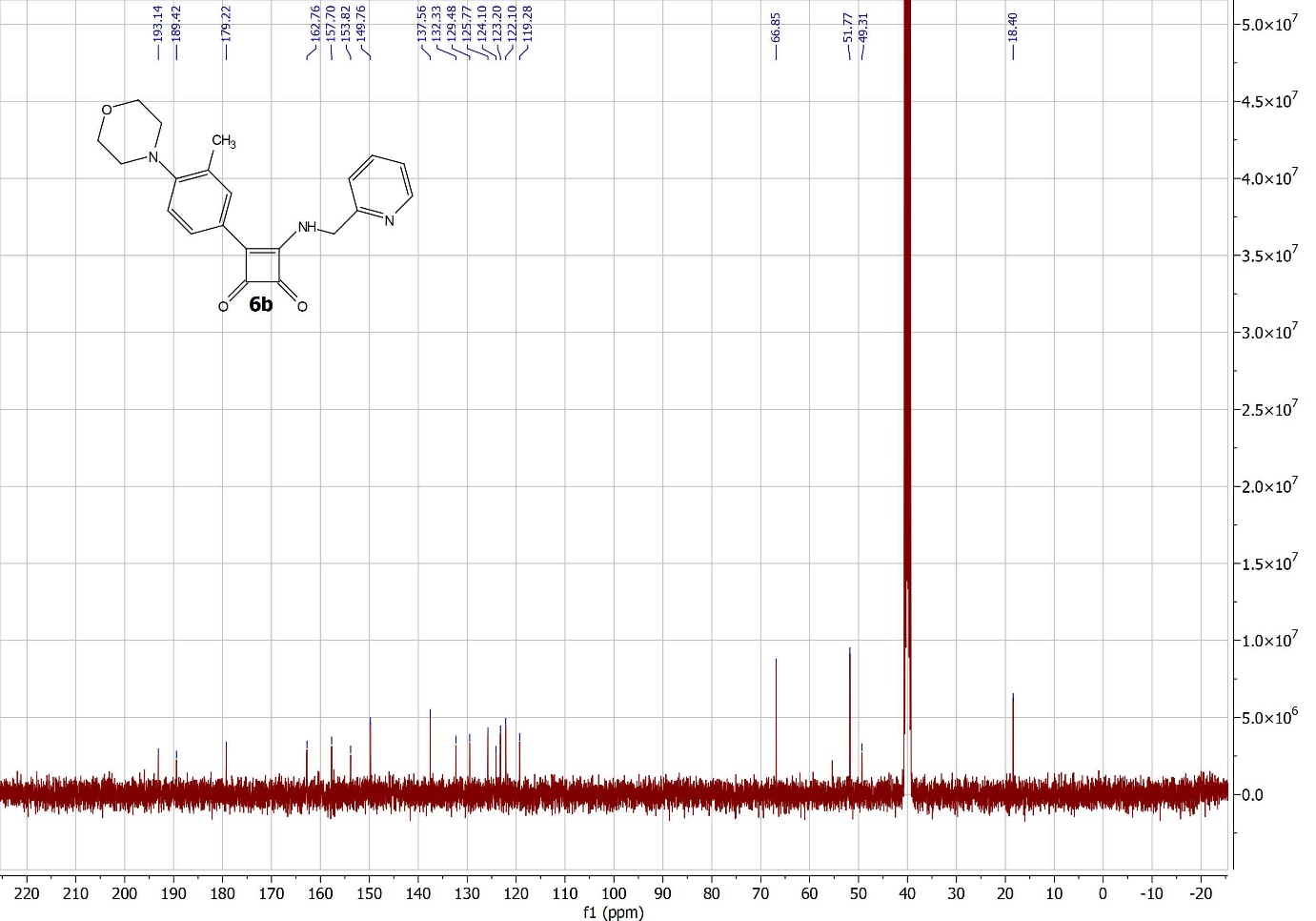


**Figure S18.** ¹H NMR (400 MHz, DMSO) and ^13^C NMR (101 MHz, DMSO) of compound **6c**


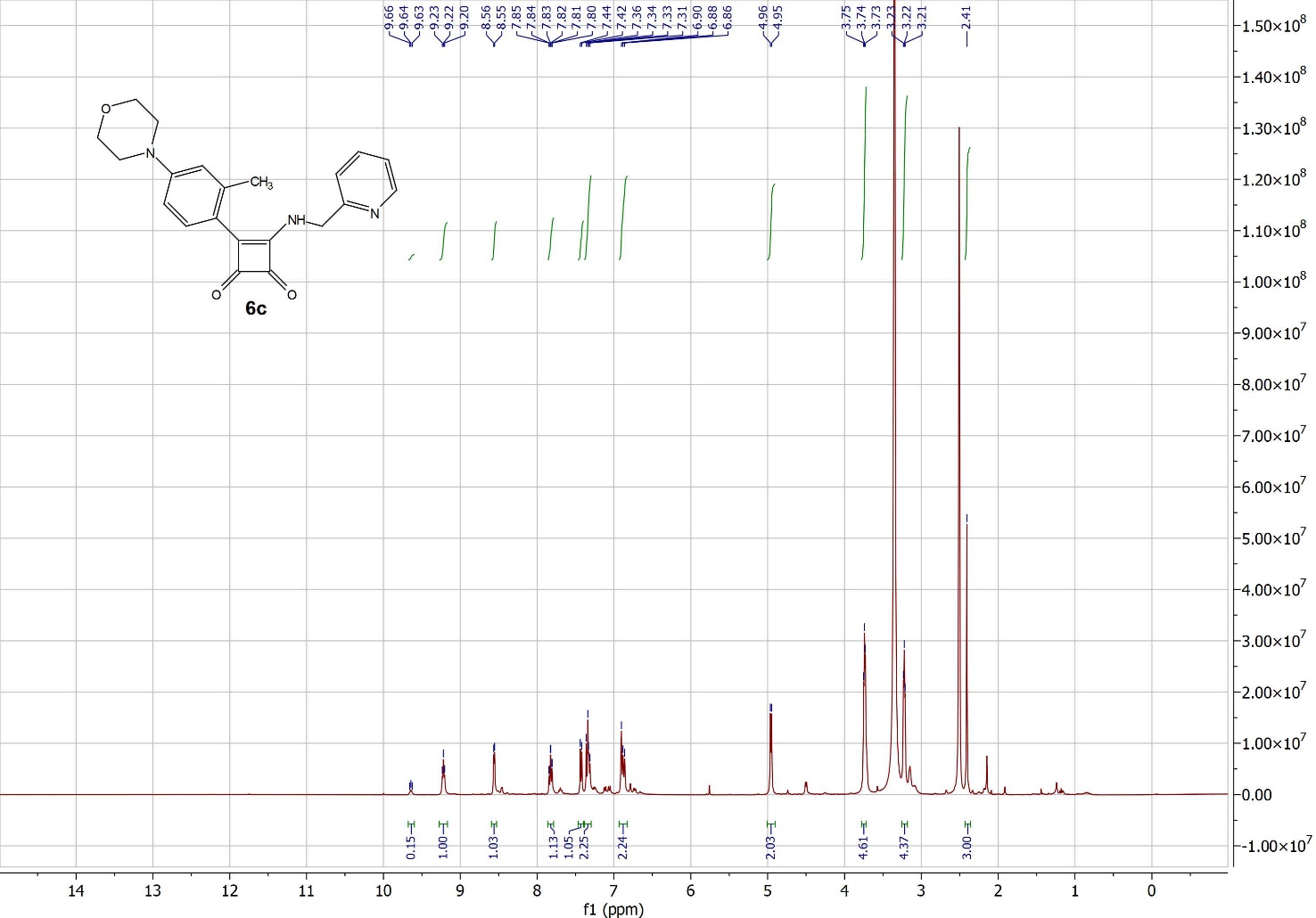

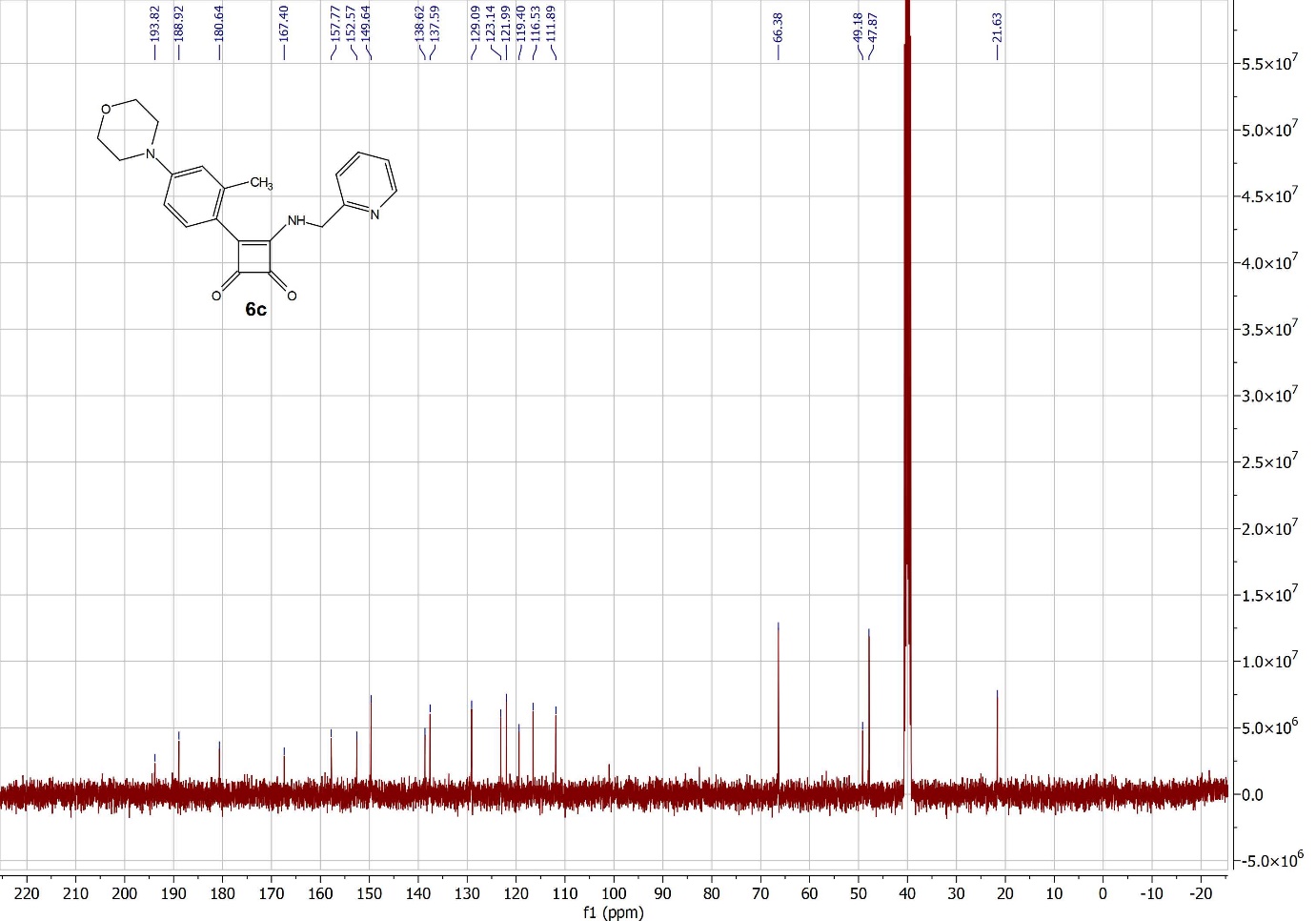


**Figure S19.** ¹H NMR (400 MHz, DMSO) and ^13^C NMR (101 MHz, DMSO) of compound **6d**


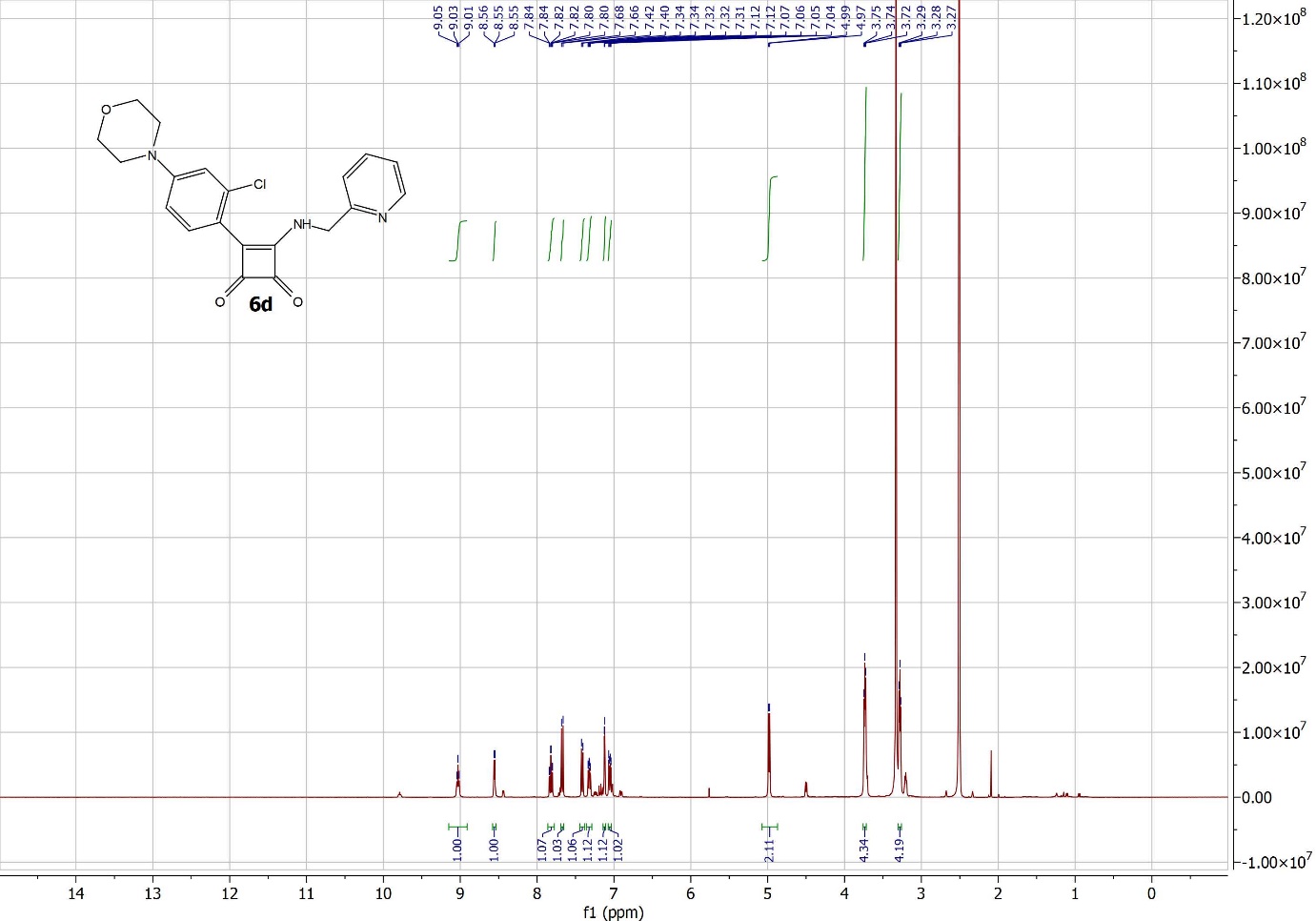

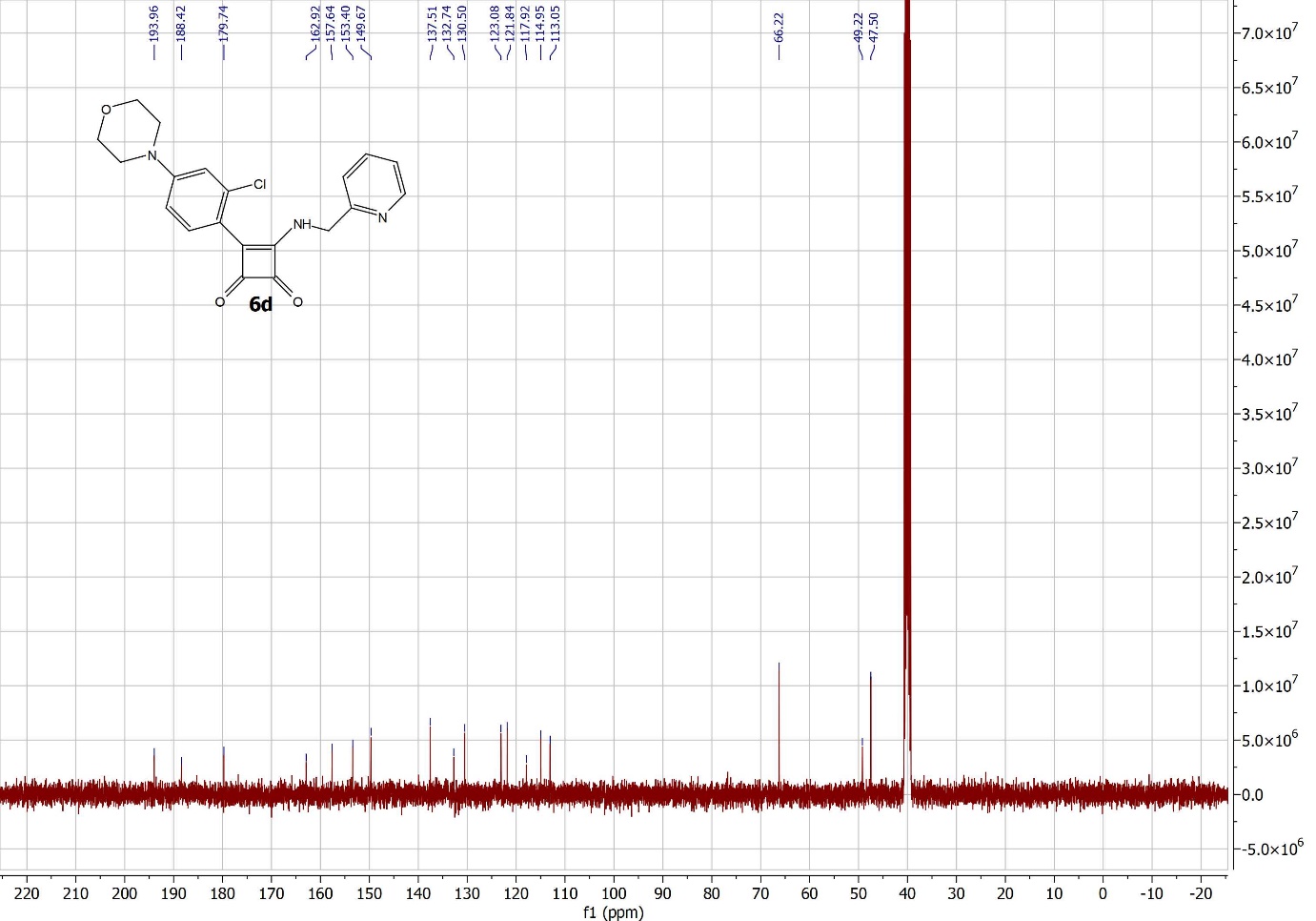


**Figure S20.** ¹H NMR (400 MHz, CDCl_3_ and ^13^C NMR (101 MHz, CDCl_3_) of compound **6e**


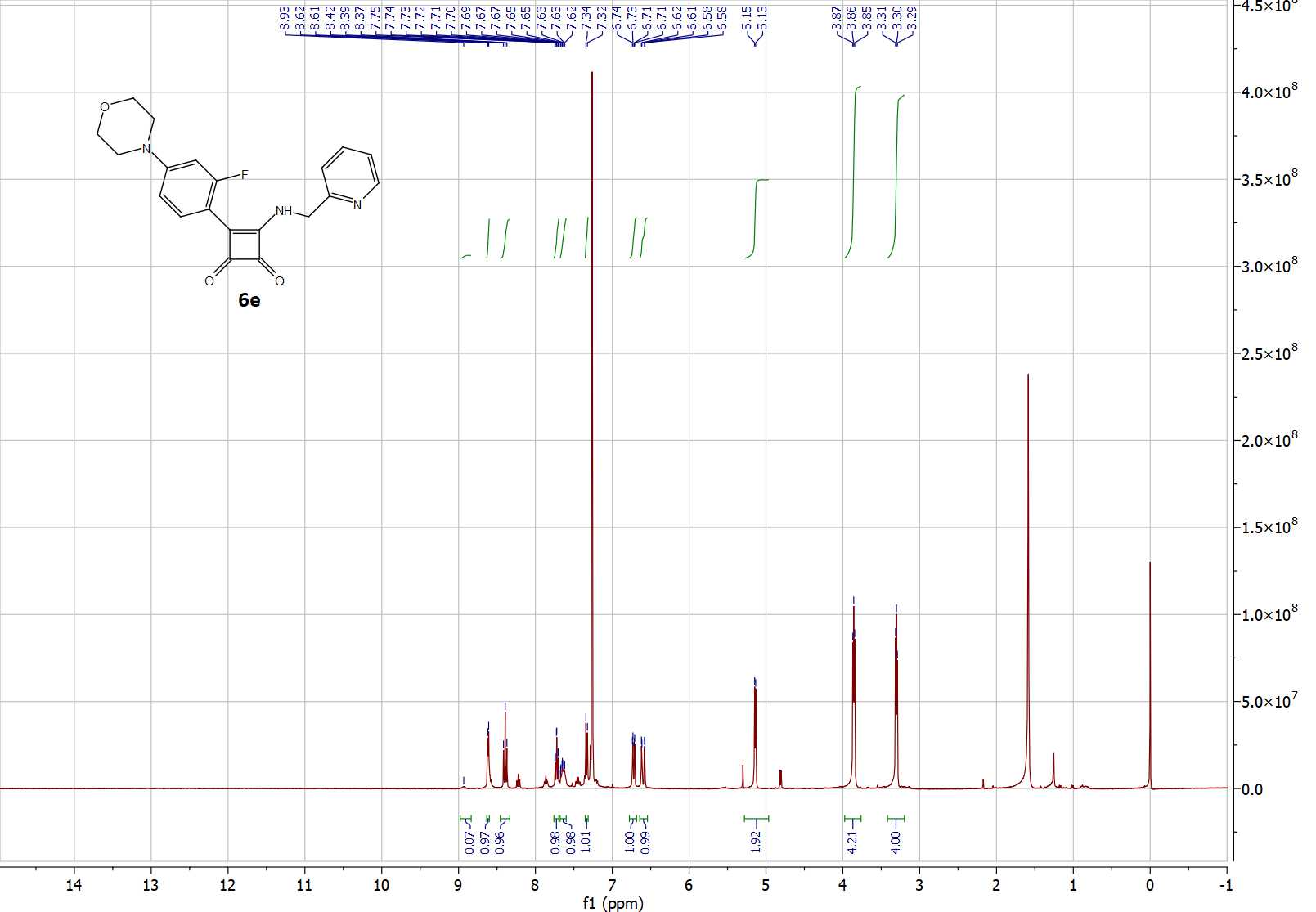

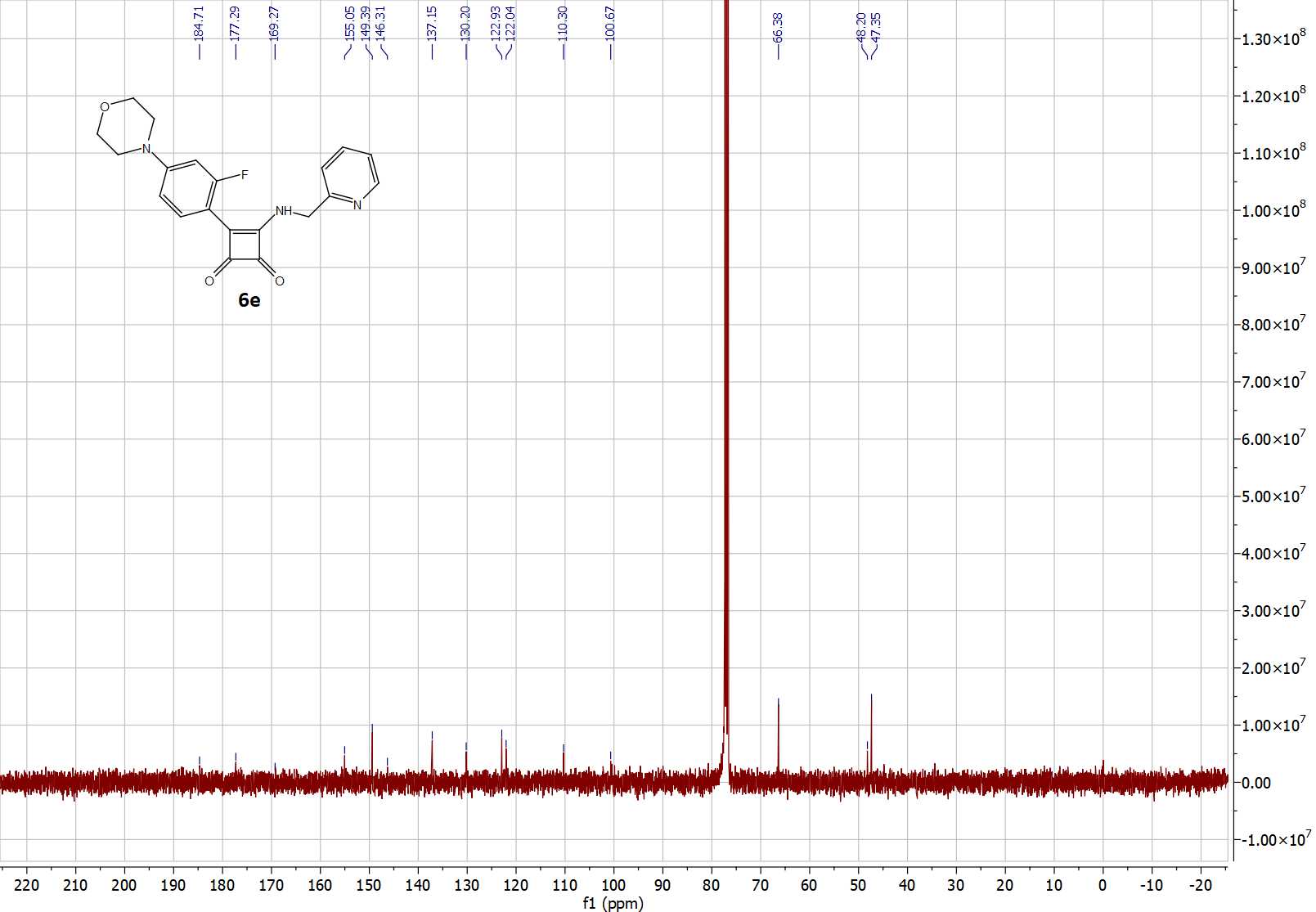


**Figure S21.** ¹H NMR (400 MHz, DMSO) and ^13^C NMR (101 MHz, DMSO) of compound **6f**


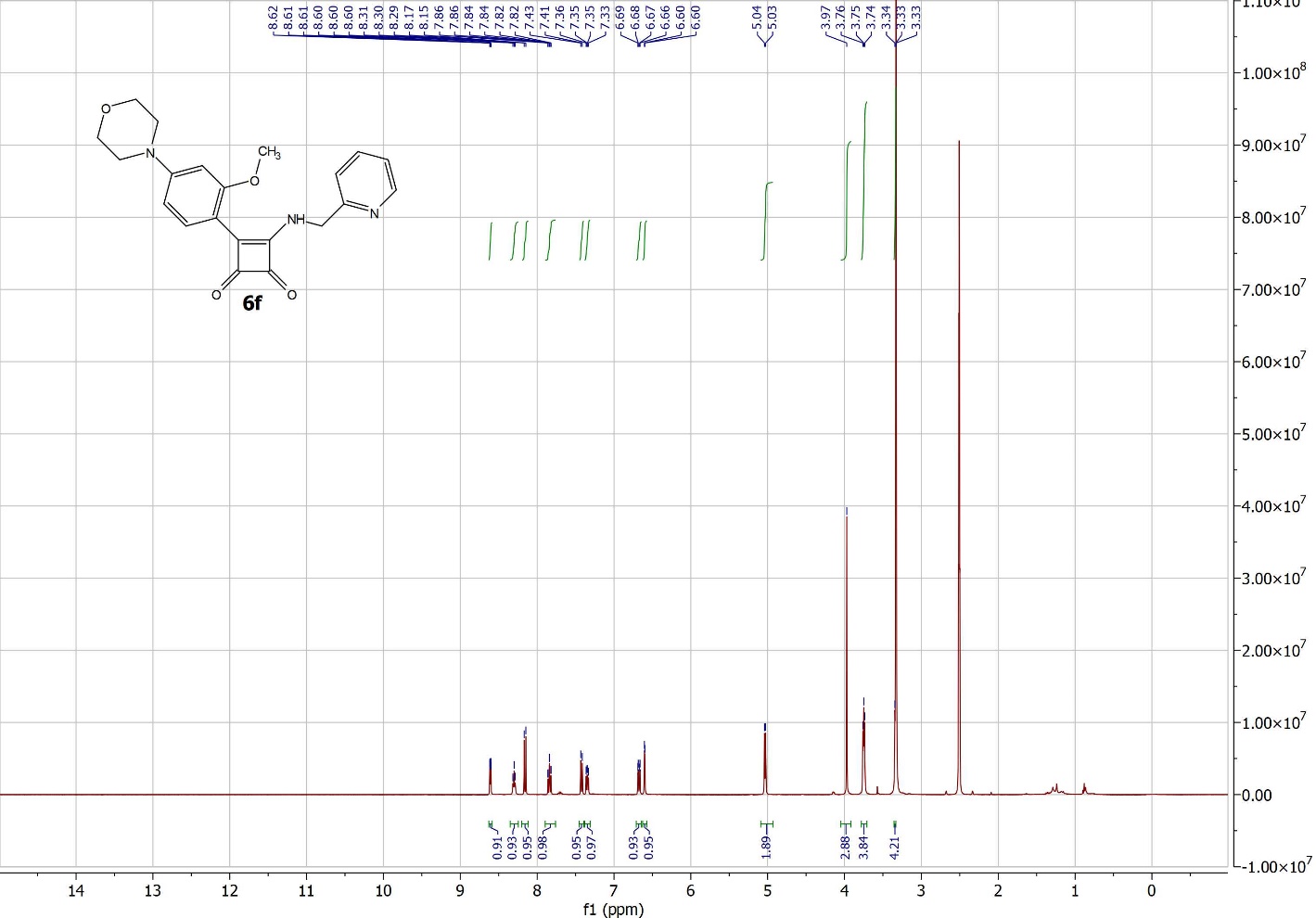

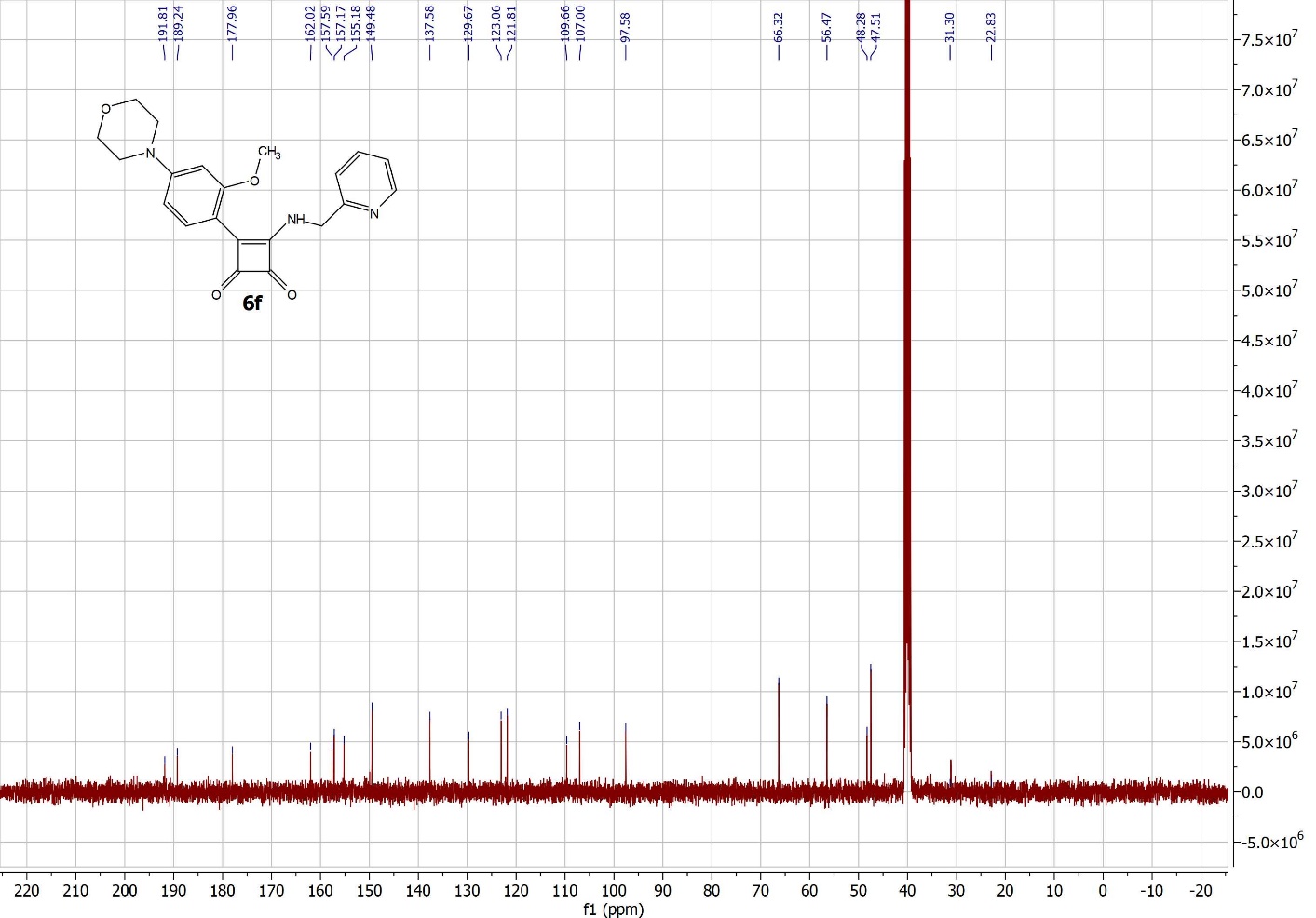


**Figure S22.** ¹H NMR (400 MHz, DMSO), ^13^C NMR (101 MHz, DMSO) and HPLC chromatogram of compound **6g**


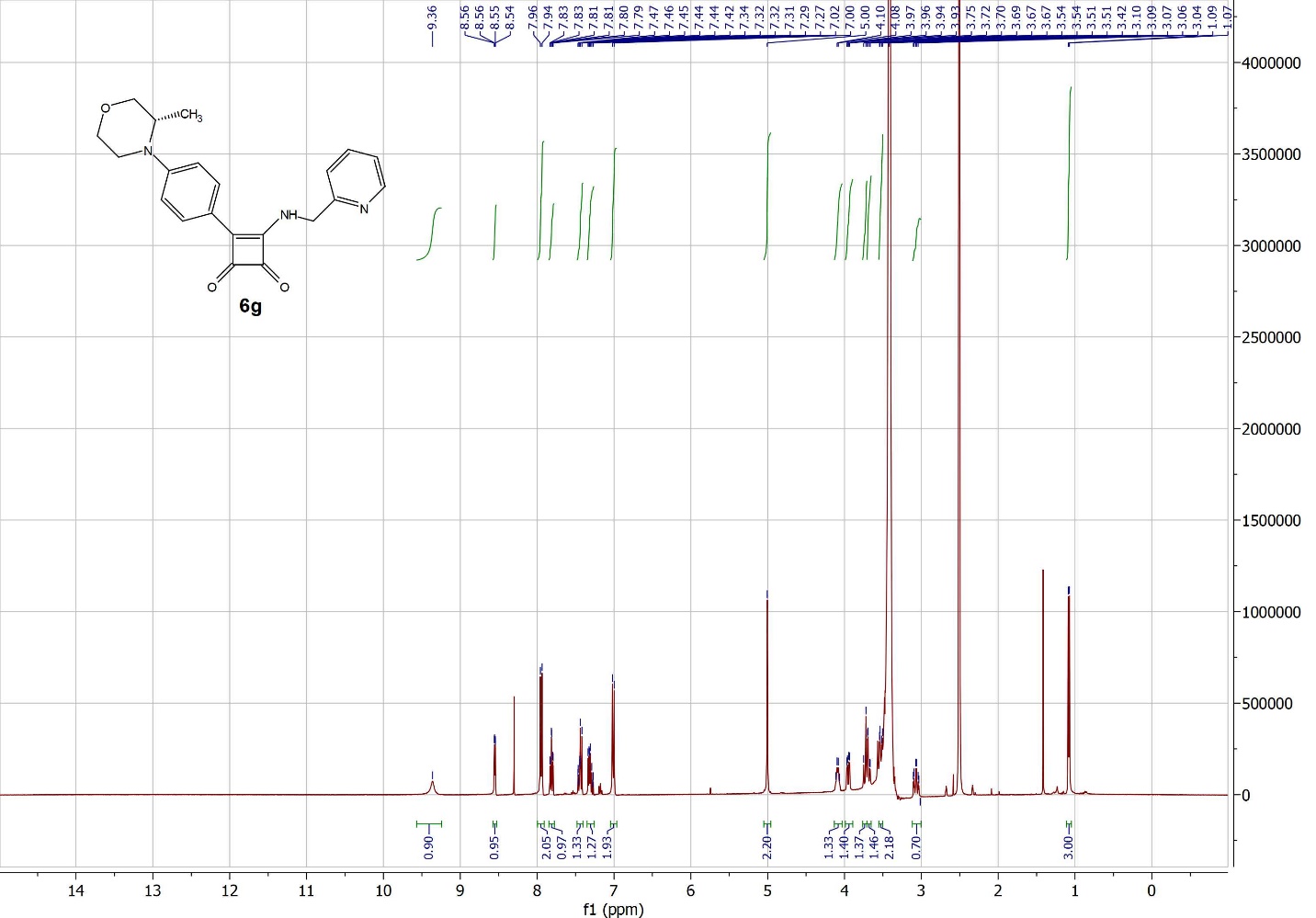

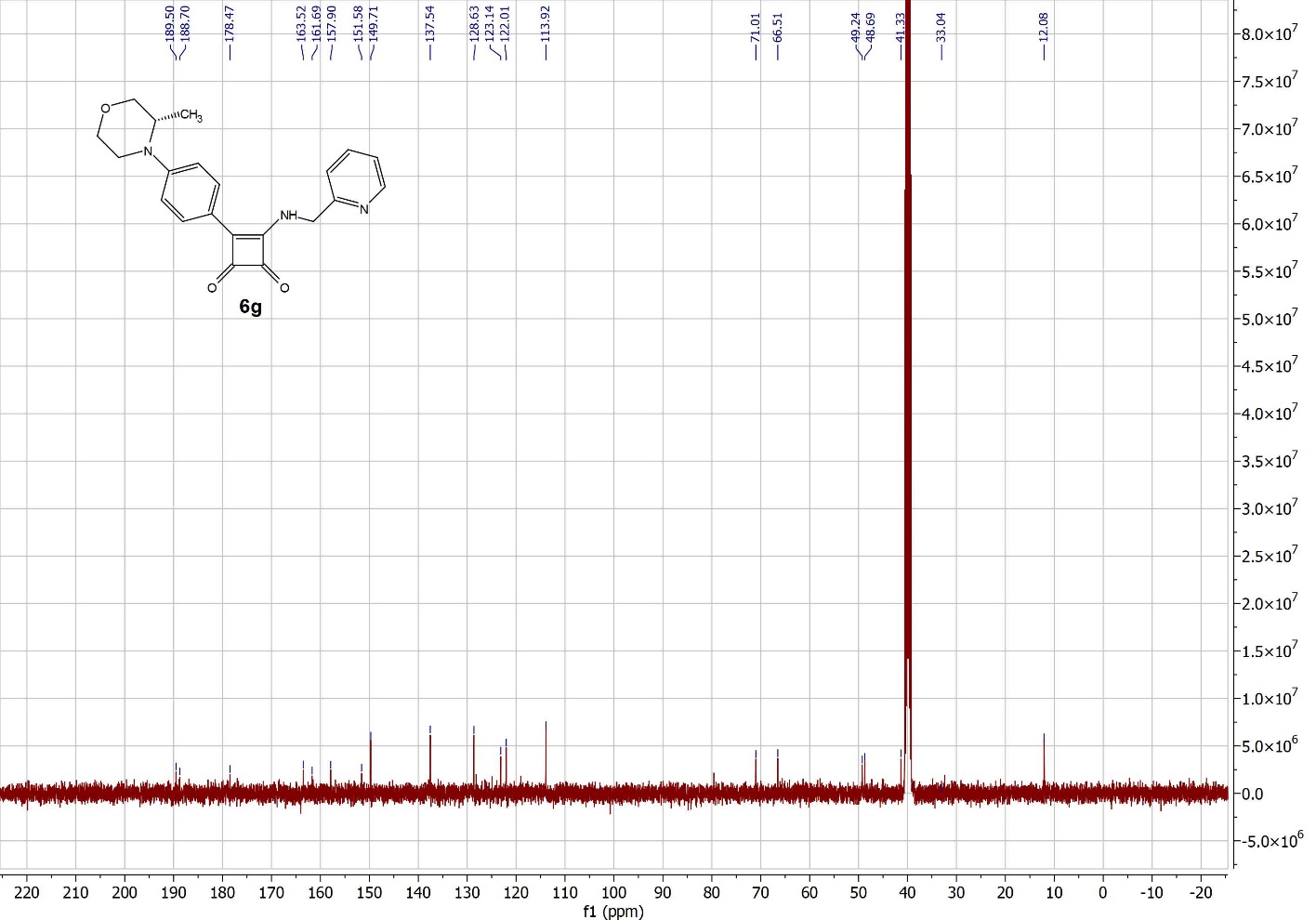


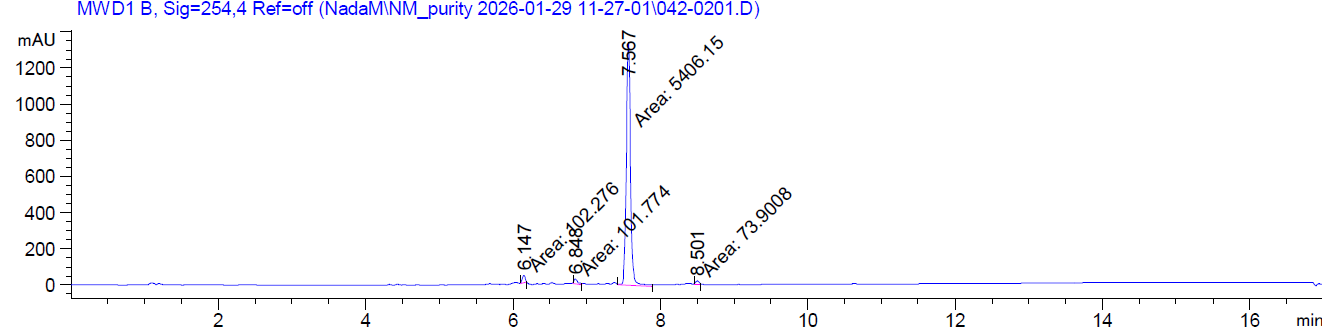


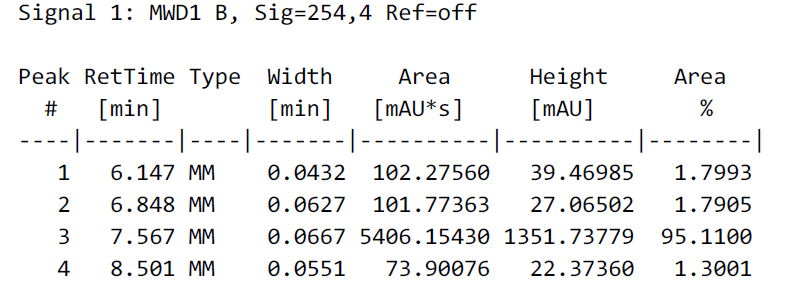


**Figure S23.** ¹H NMR (400 MHz, DMSO), ^13^C NMR (101 MHz, DMSO) and HPLC chromatogram of compound **6h**


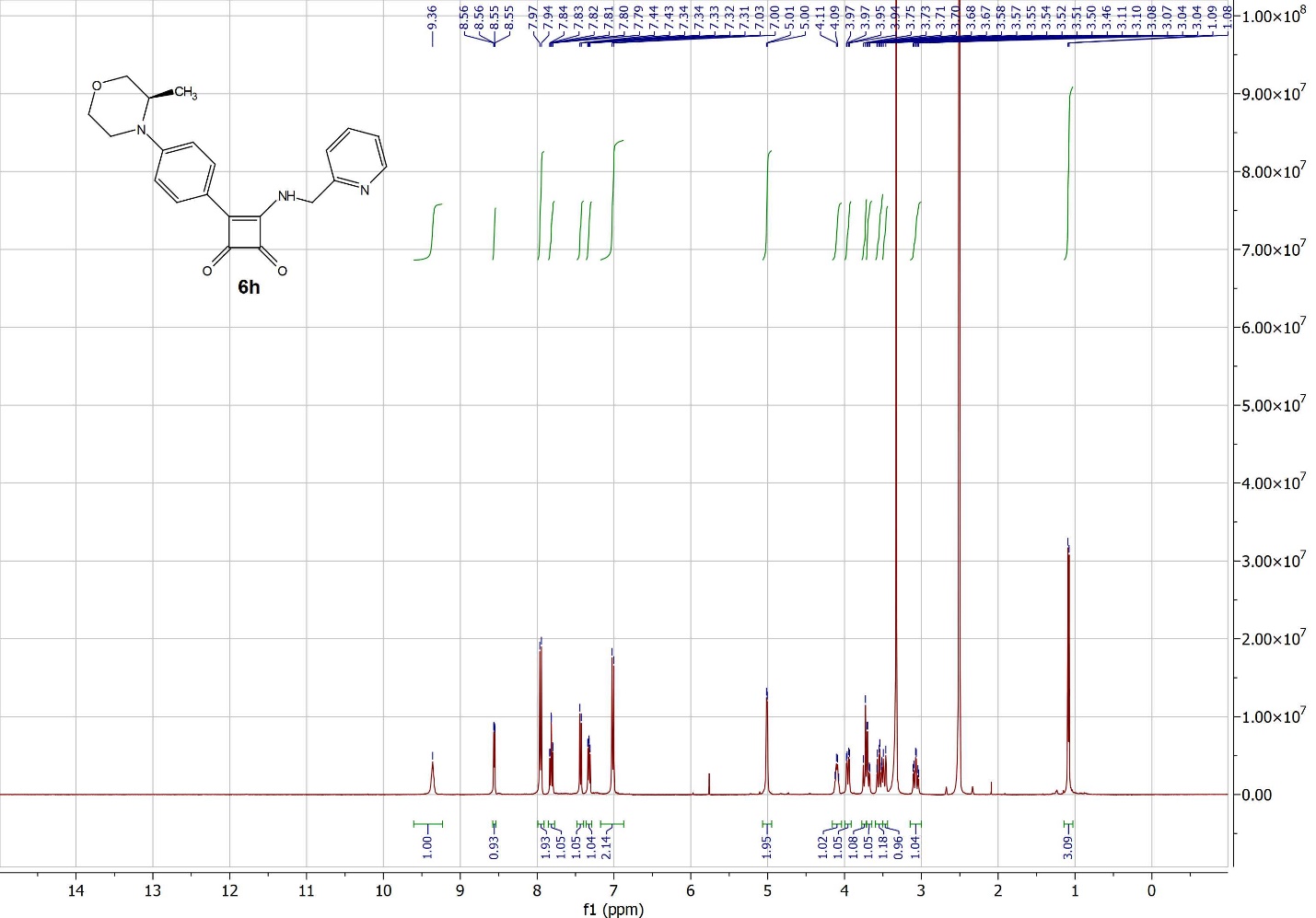
 **
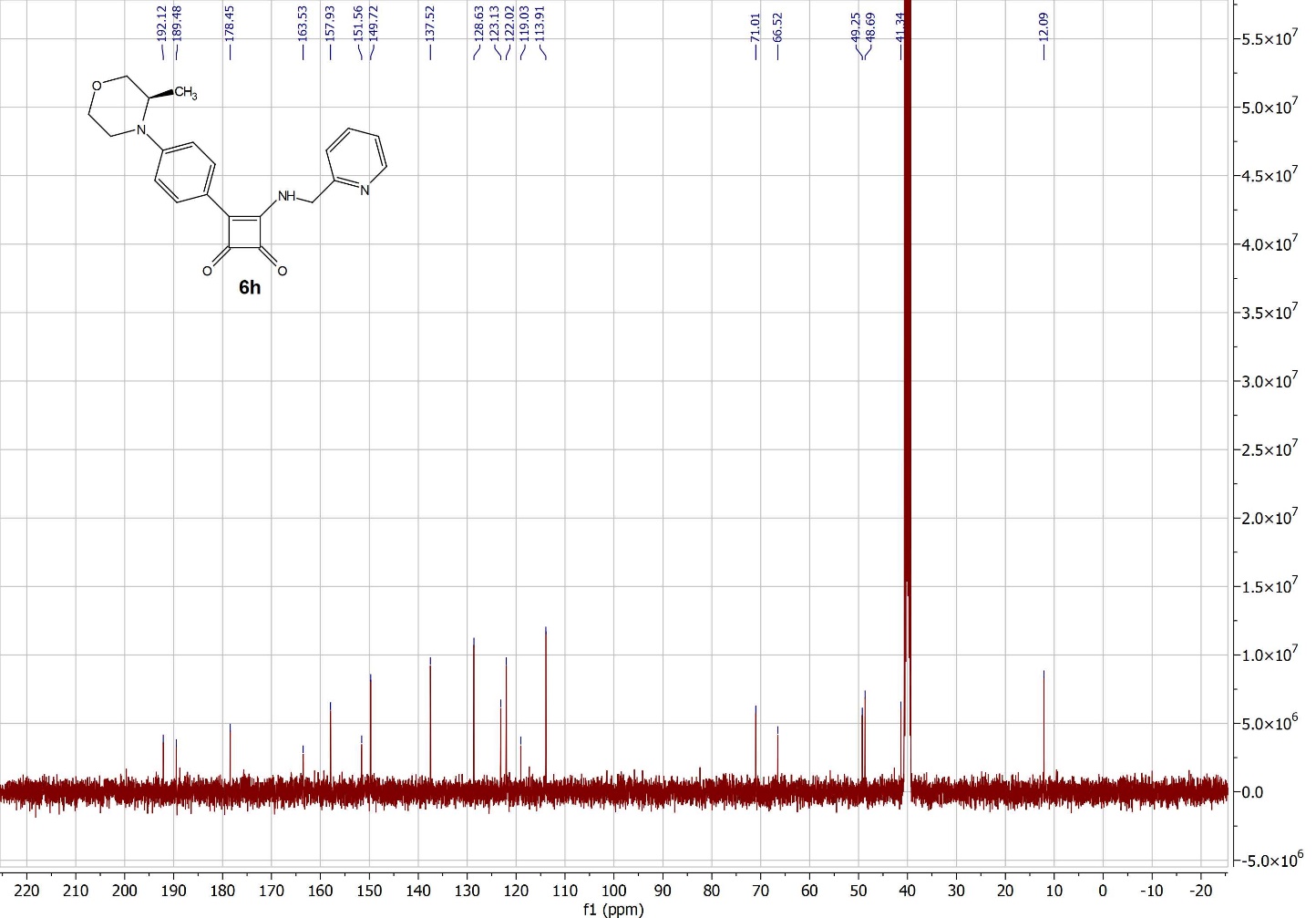
**


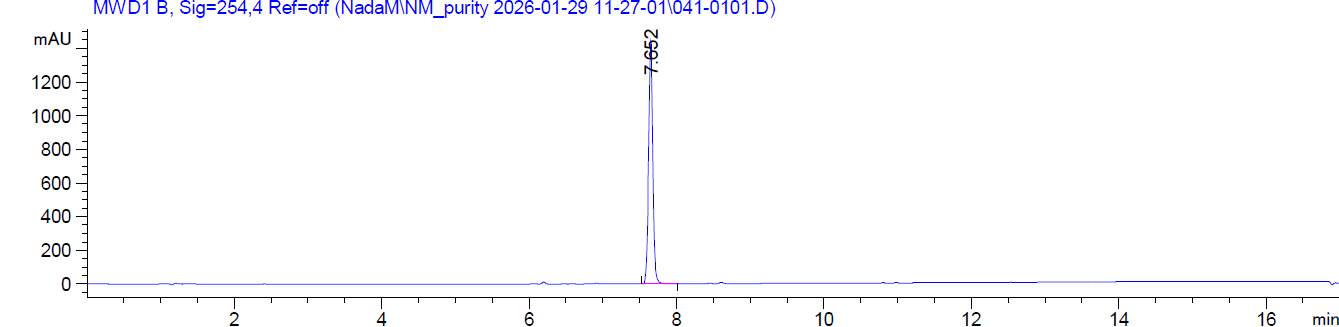


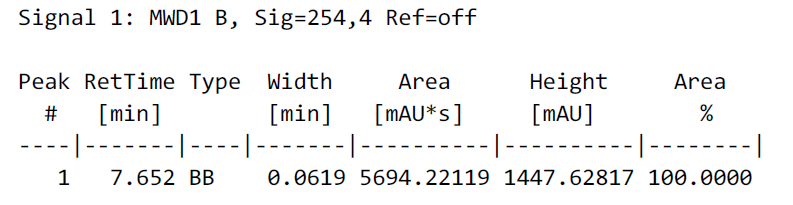


**Figure S24.** ¹H NMR (400 MHz, DMSO) and ^13^C NMR (101 MHz, DMSO) of compound **6i**


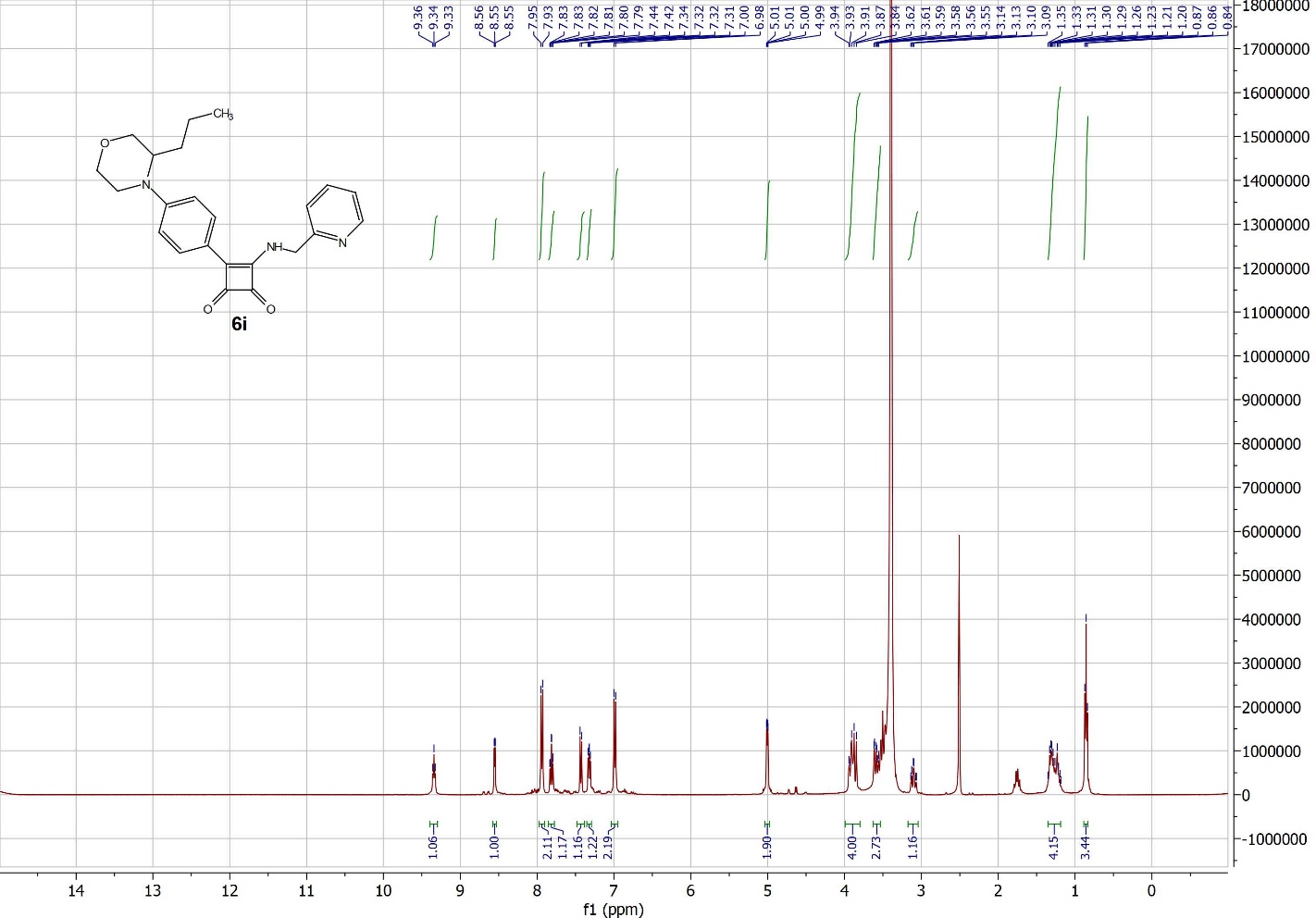

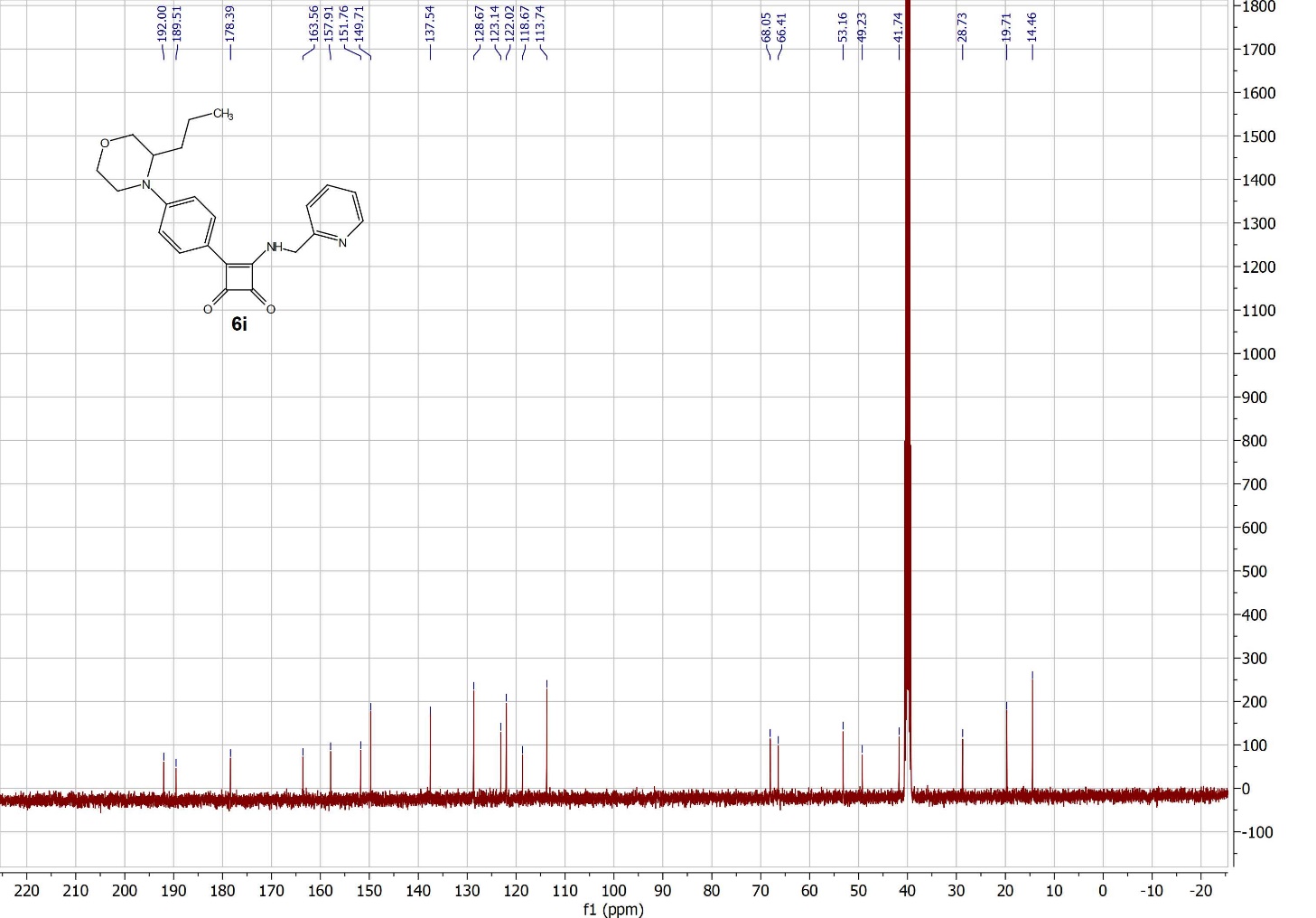


**Figure S25.** ¹H NMR (400 MHz, DMSO) and ESI-HRMS of compound **6j**


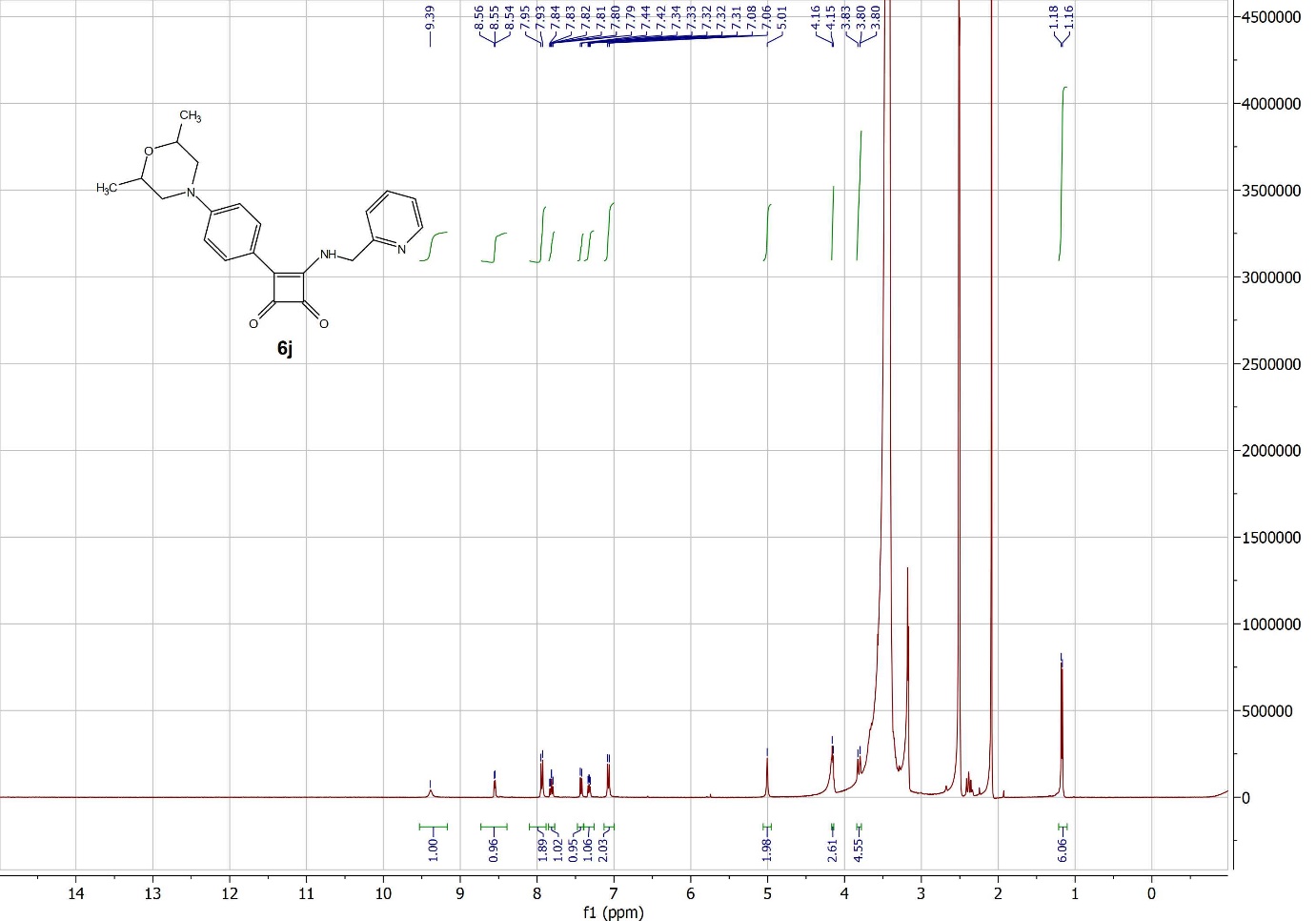


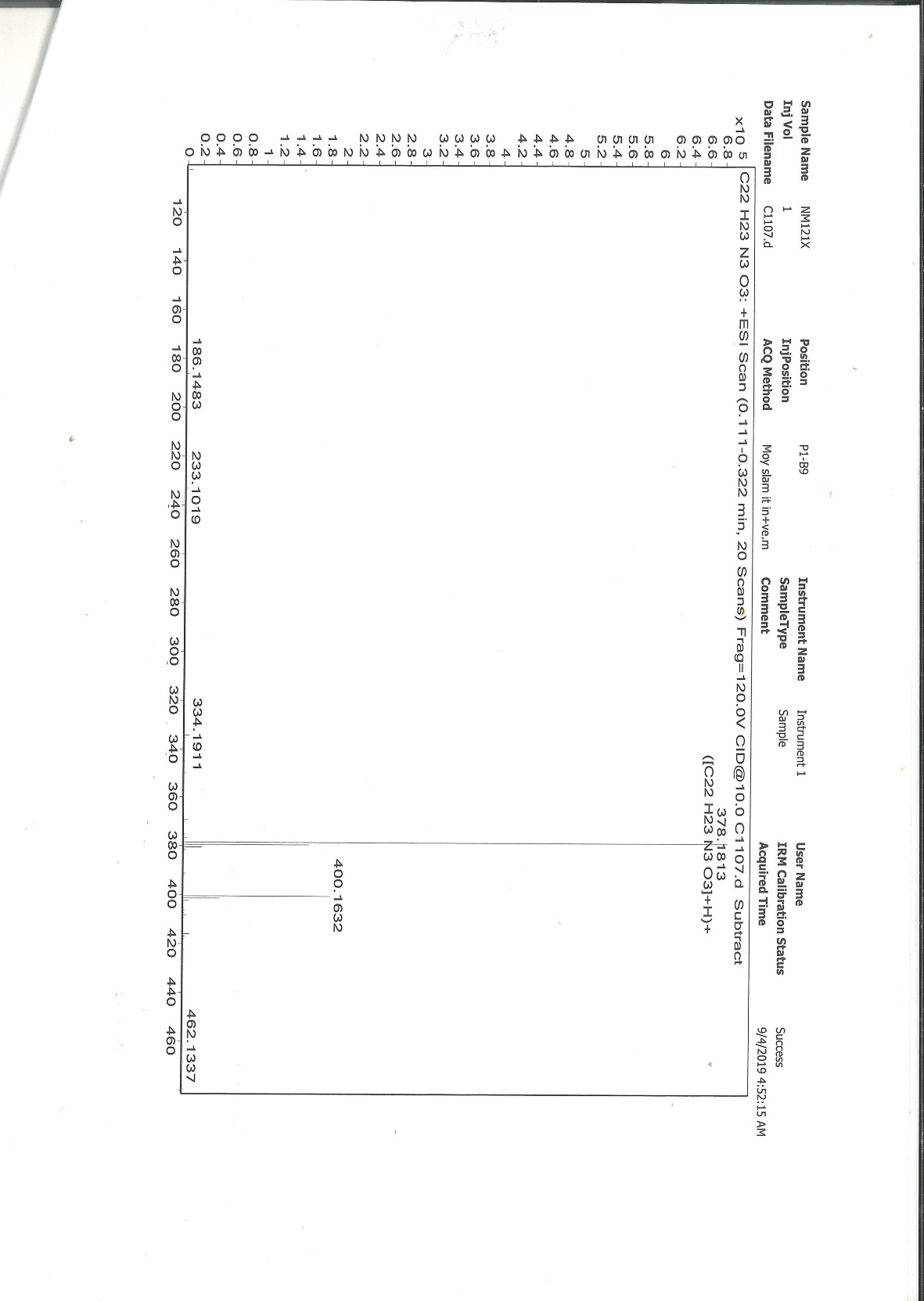


**6j**

**Figure S26.** ¹H NMR (400 MHz, DMSO), ^13^C NMR (101 MHz, DMSO) and HPLC chromatogram of compound **6k**


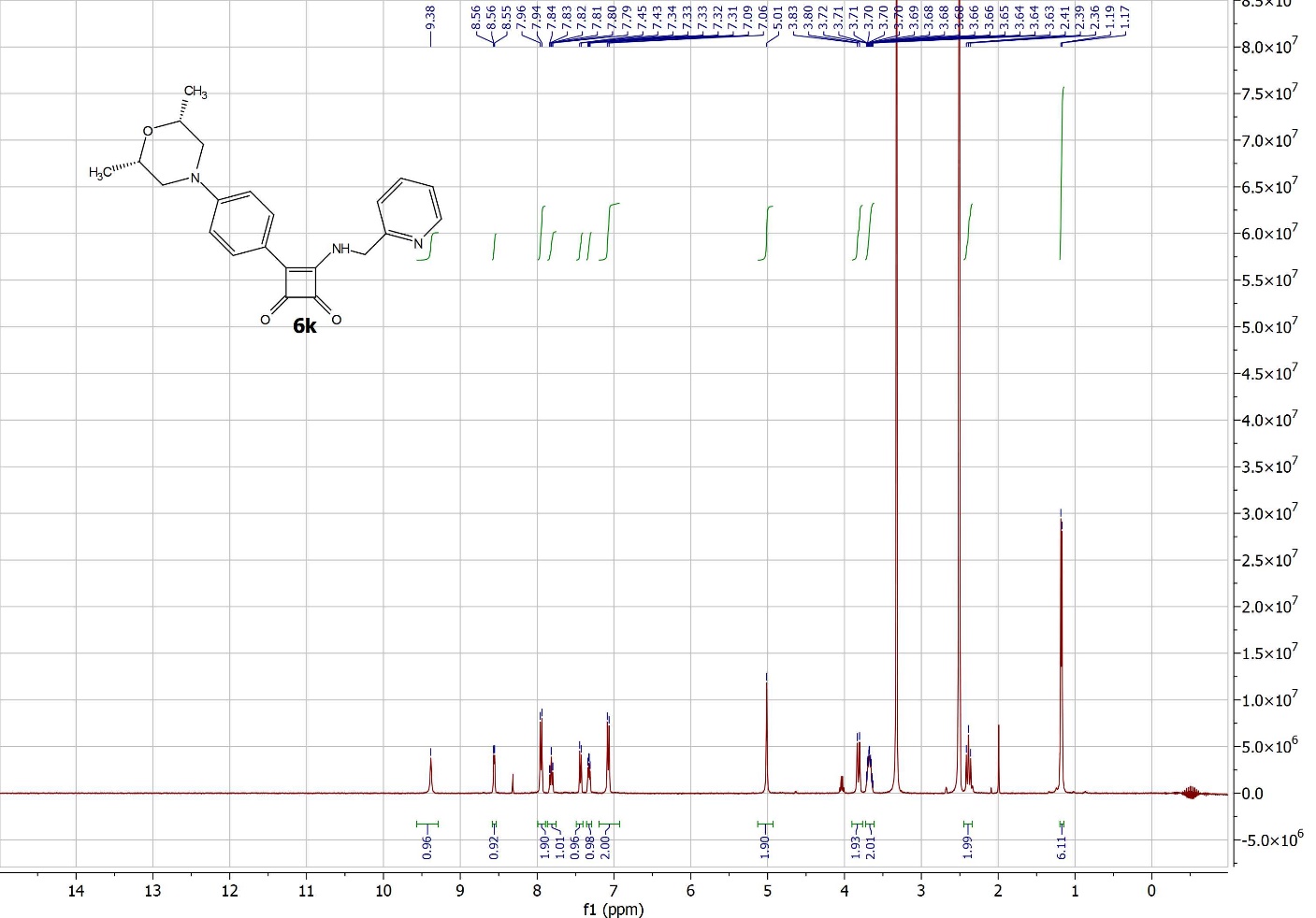

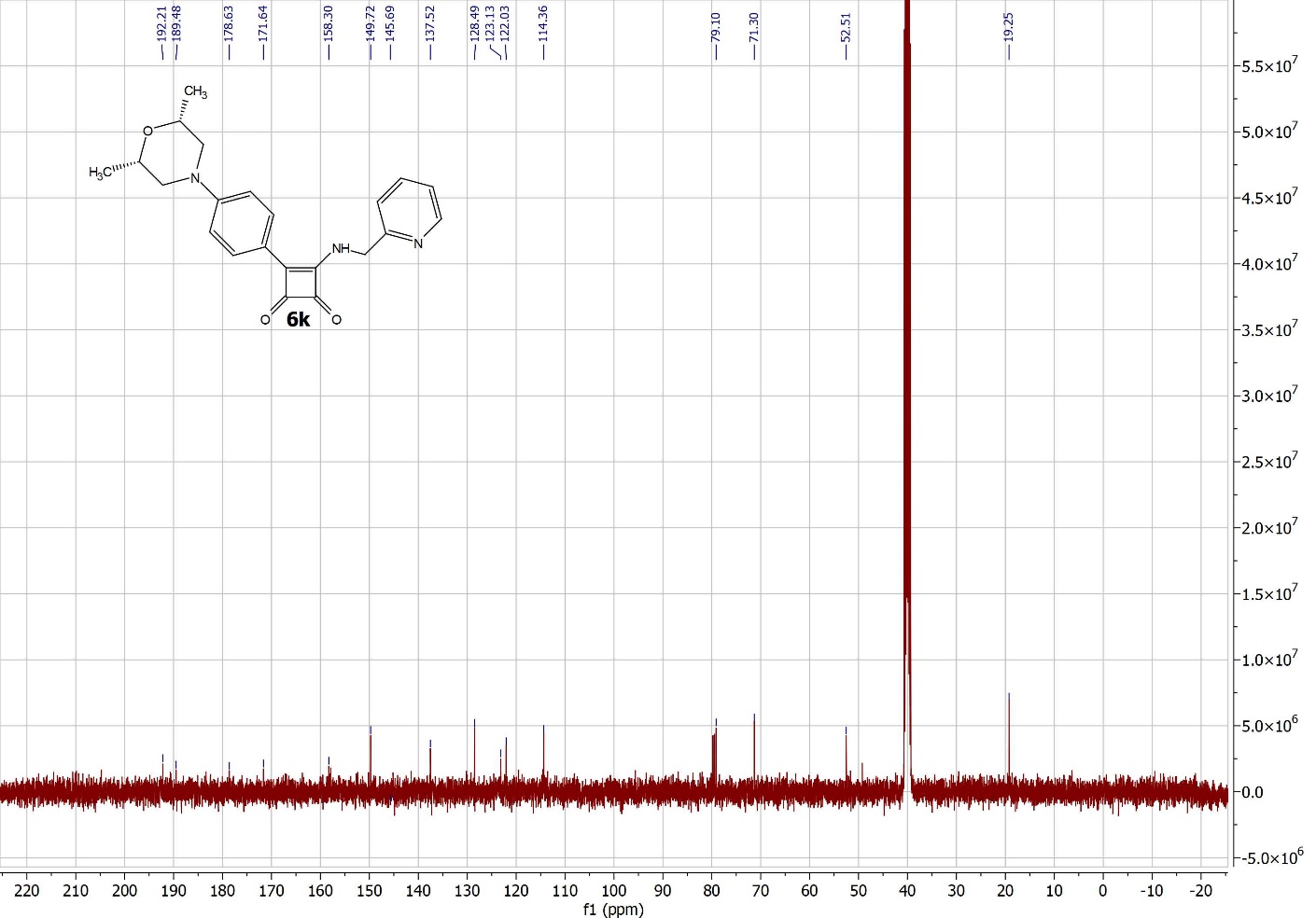


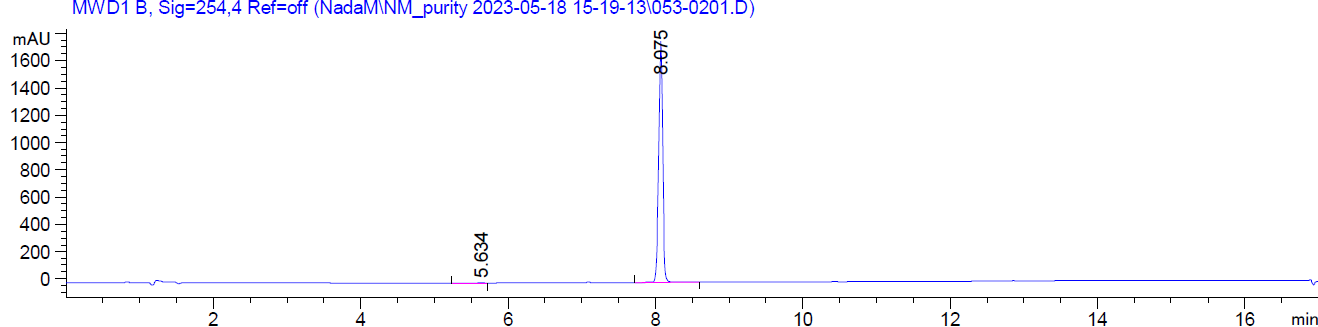


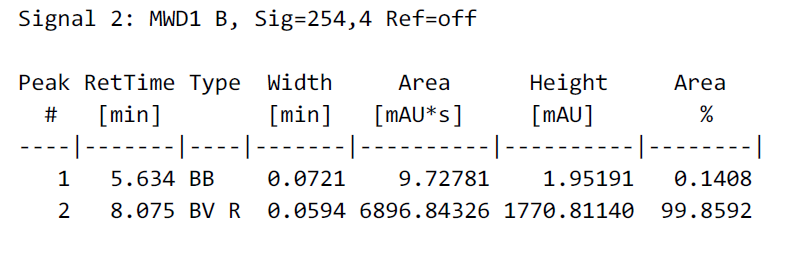


**Figure S27.** ¹H NMR (400 MHz, DMSO), ^13^C NMR (101 MHz, DMSO) and HPLC chromatogram of compound **6l**


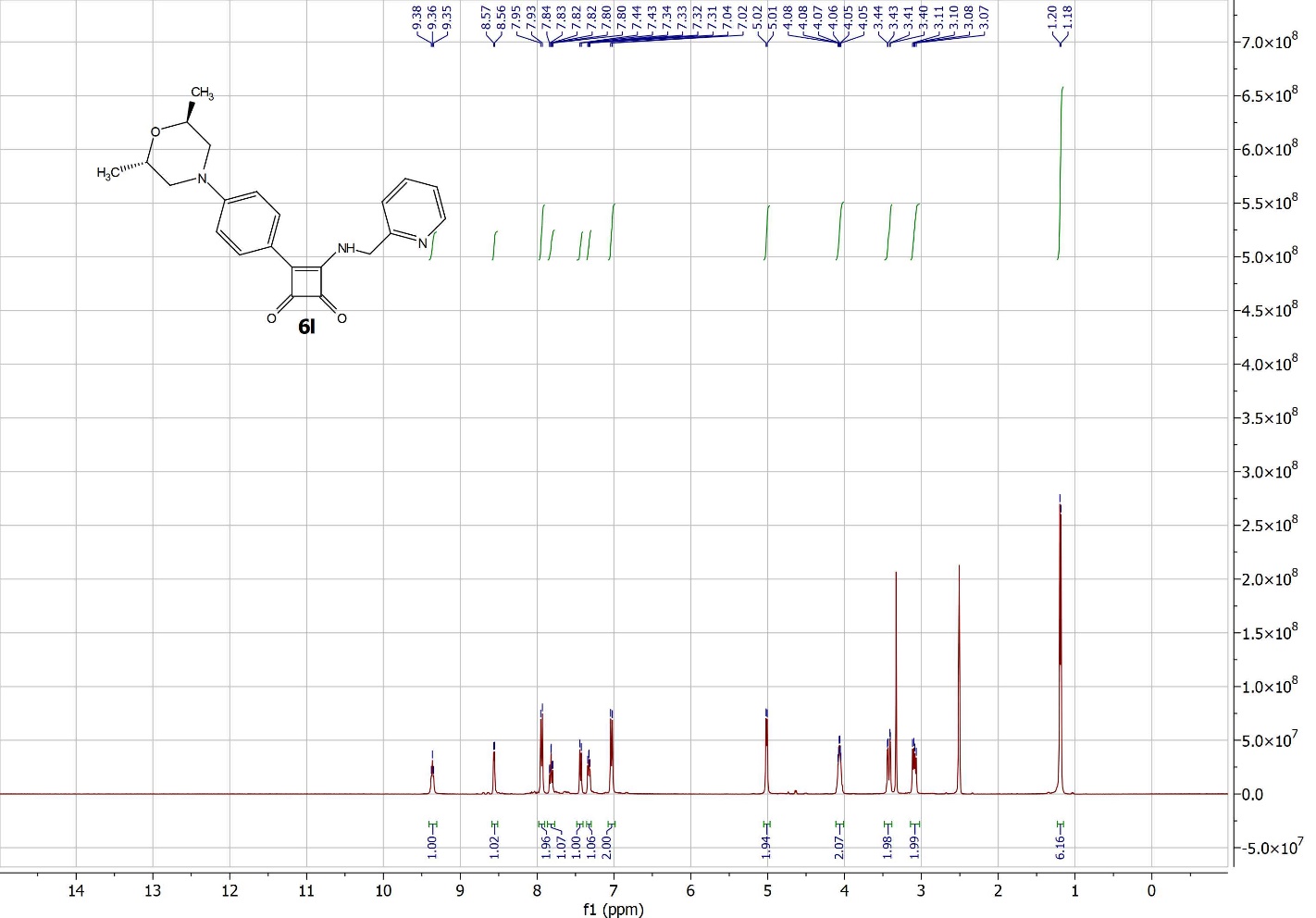

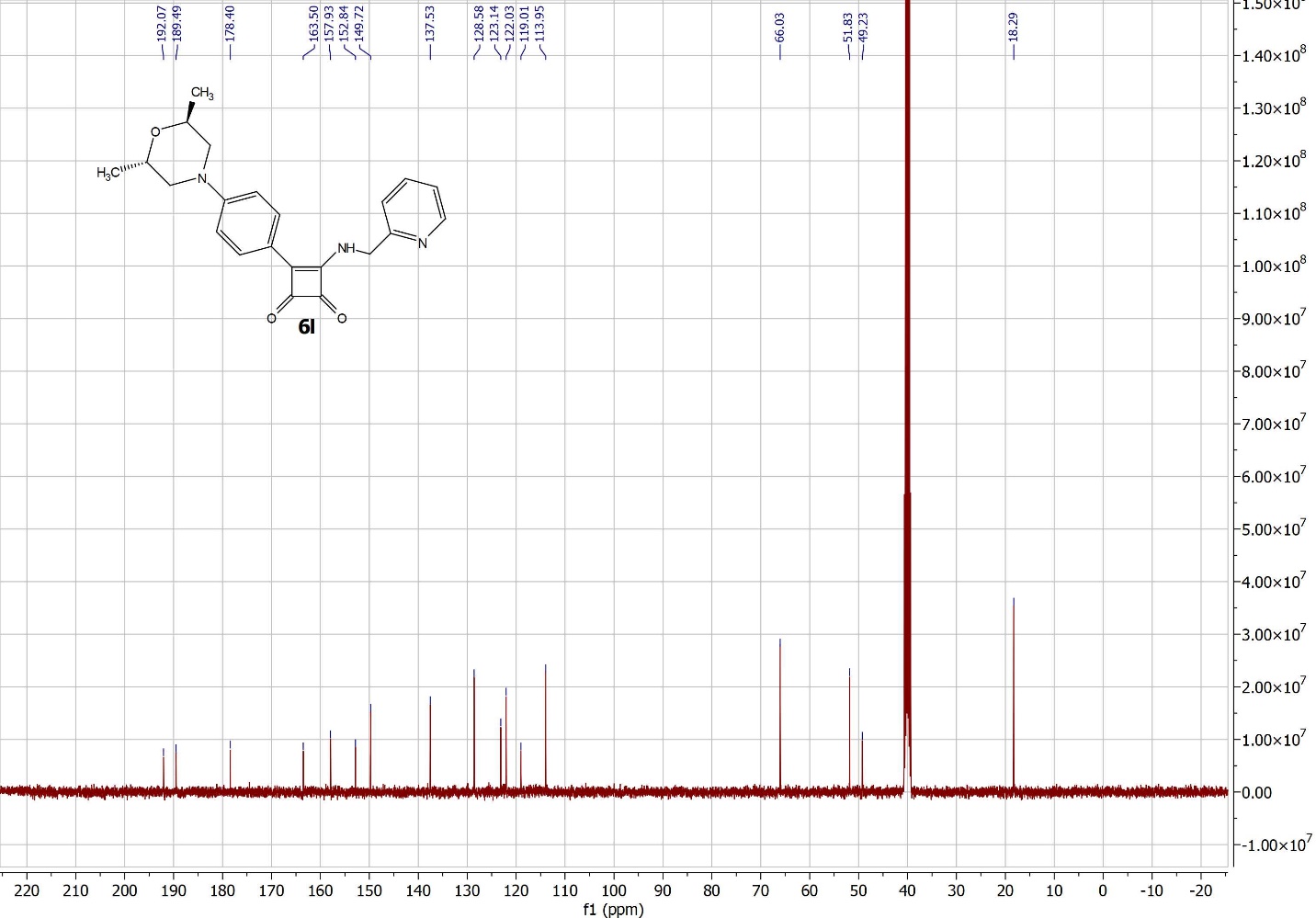


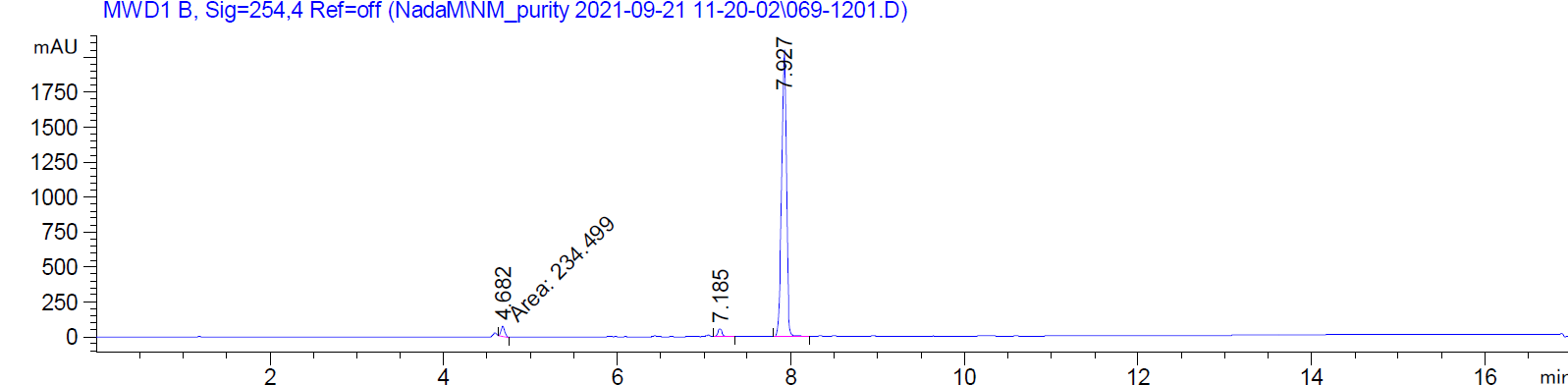


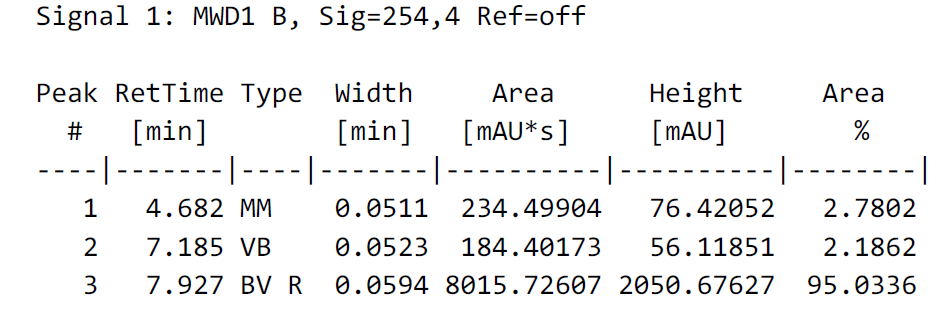


**Figure S28.** ¹H NMR (400 MHz, DMSO), ^13^C NMR (101 MHz, DMSO) and HPLC chromatogram of compound **6m**


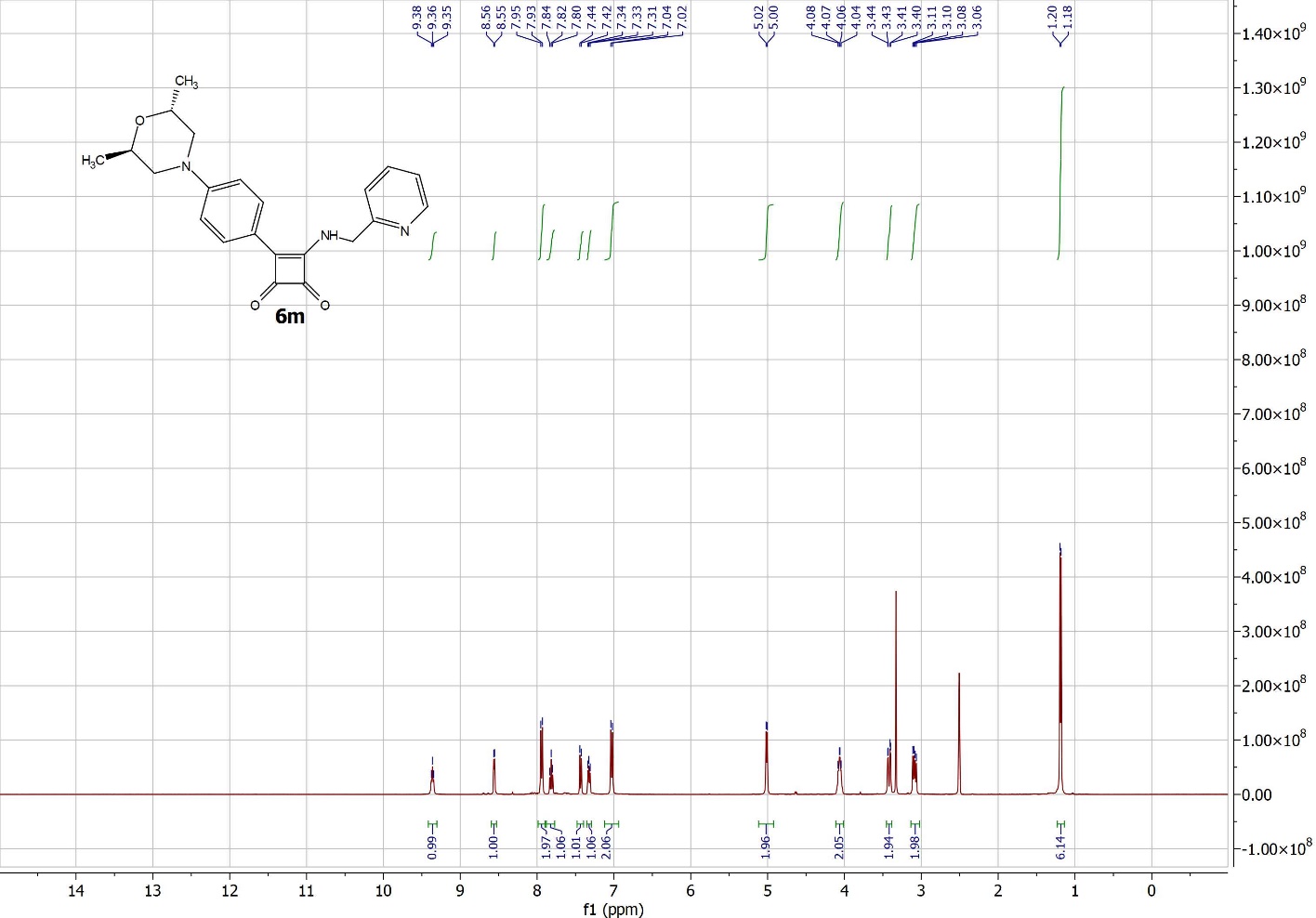

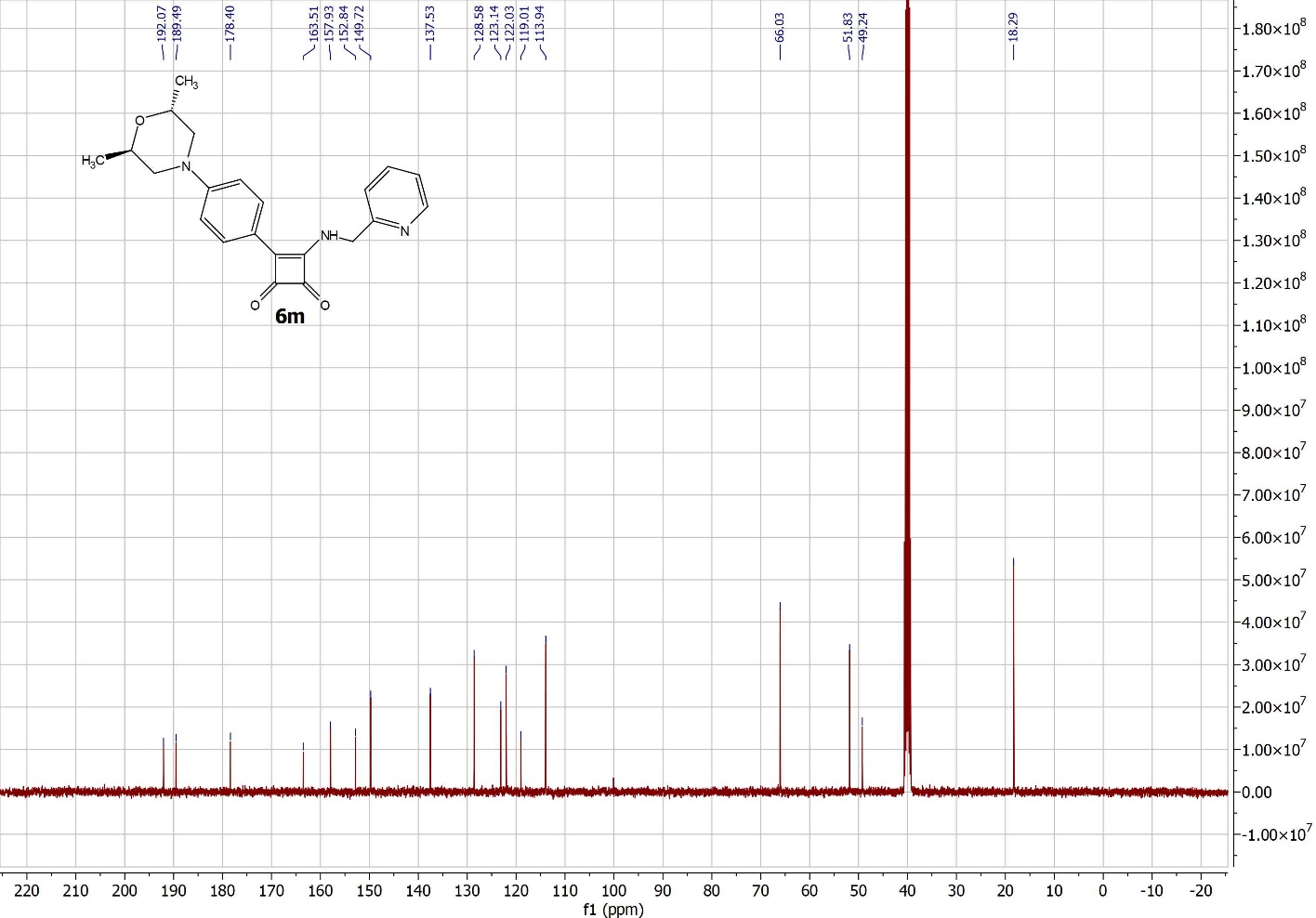


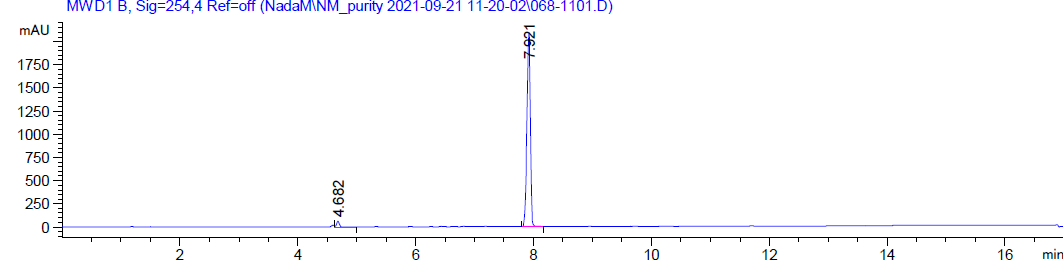


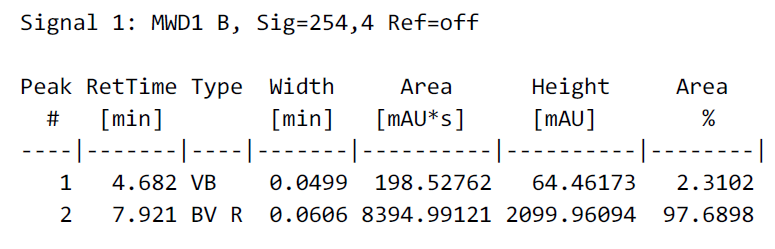


**Figure S29.** ¹H NMR (400 MHz, DMSO) and ^13^C NMR (101 MHz, DMSO) of compound **6n**


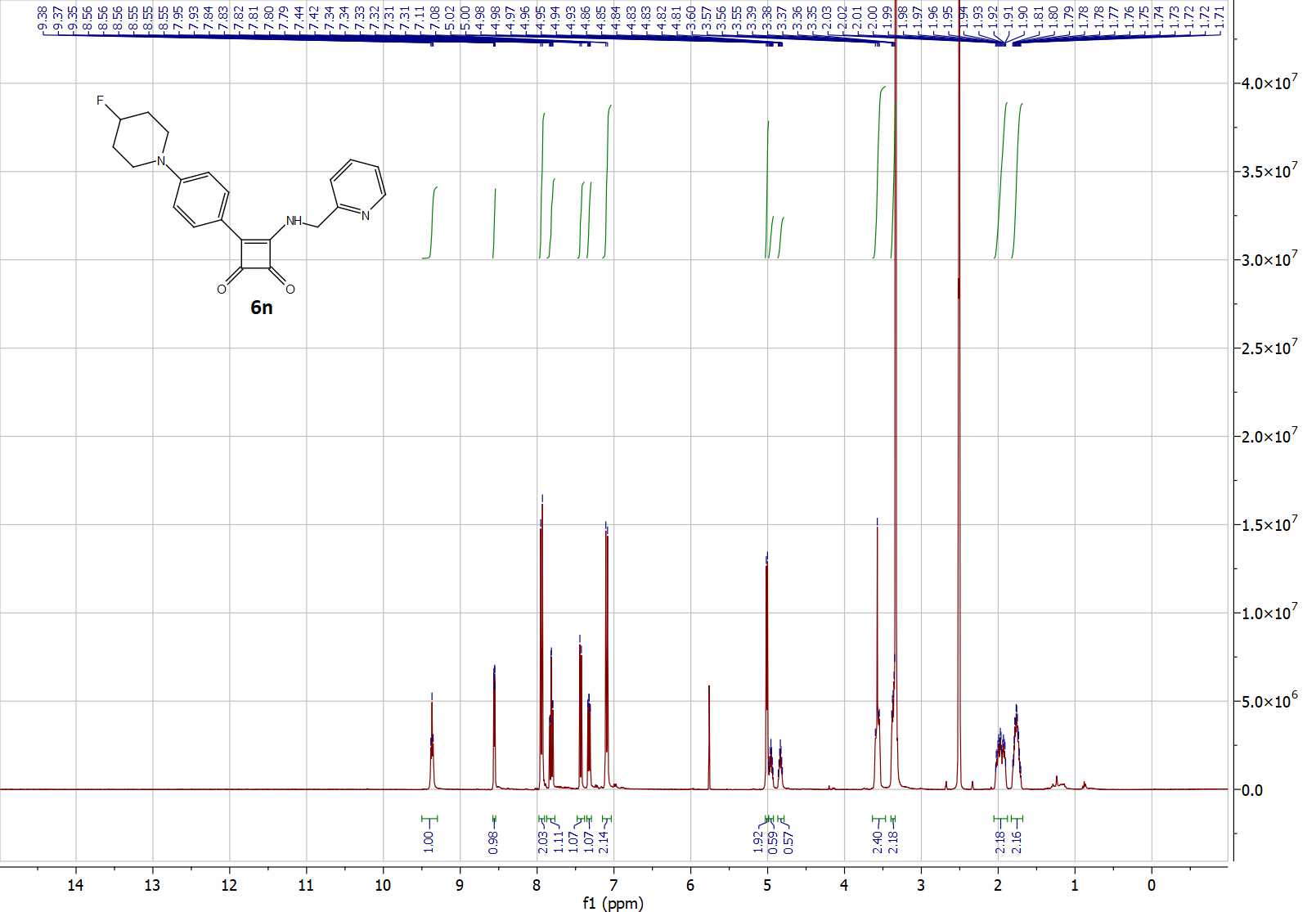

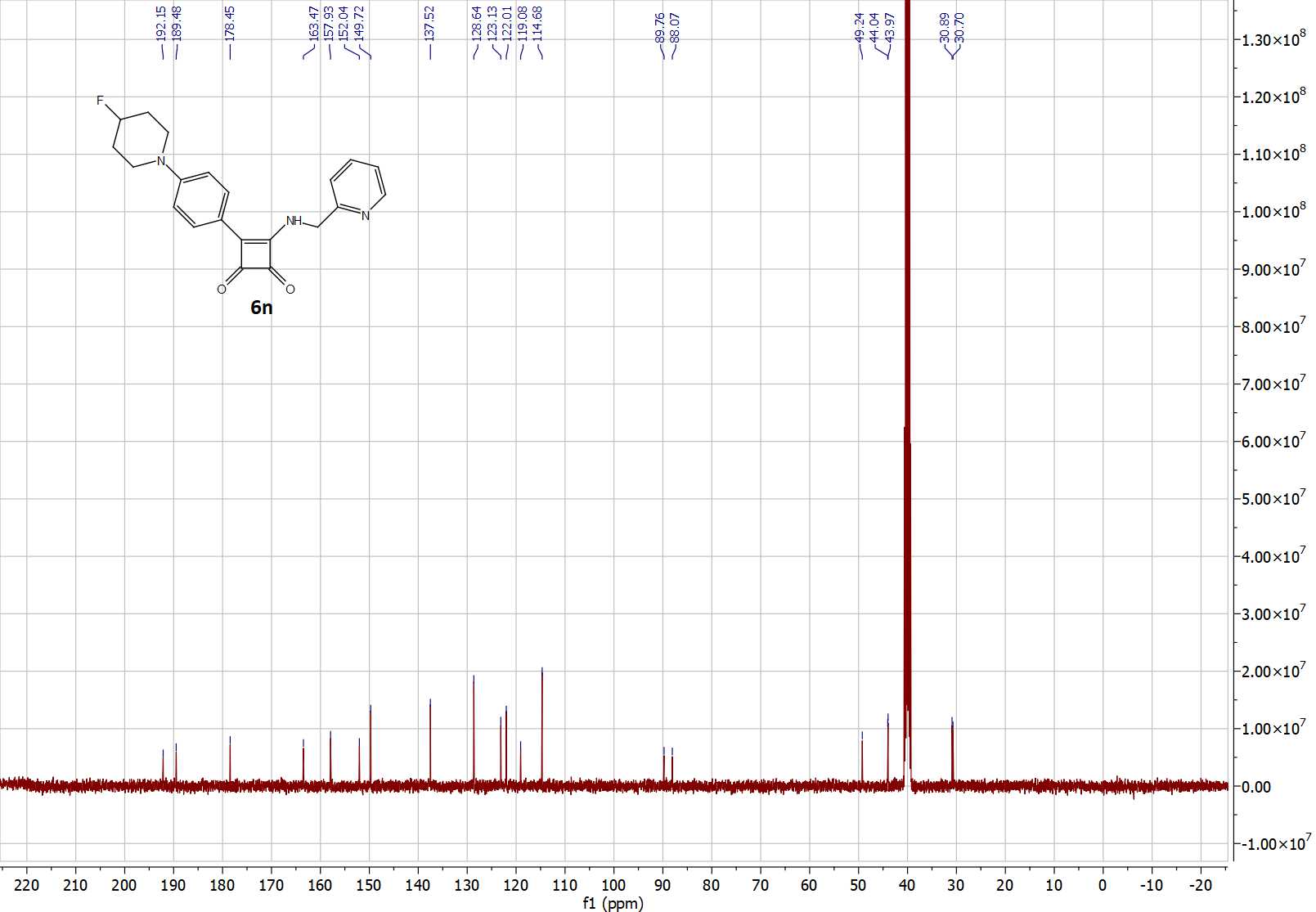


**Figure S30.** ¹H NMR (400 MHz, DMSO) and ^13^C NMR (101 MHz, DMSO) of compound **6o**


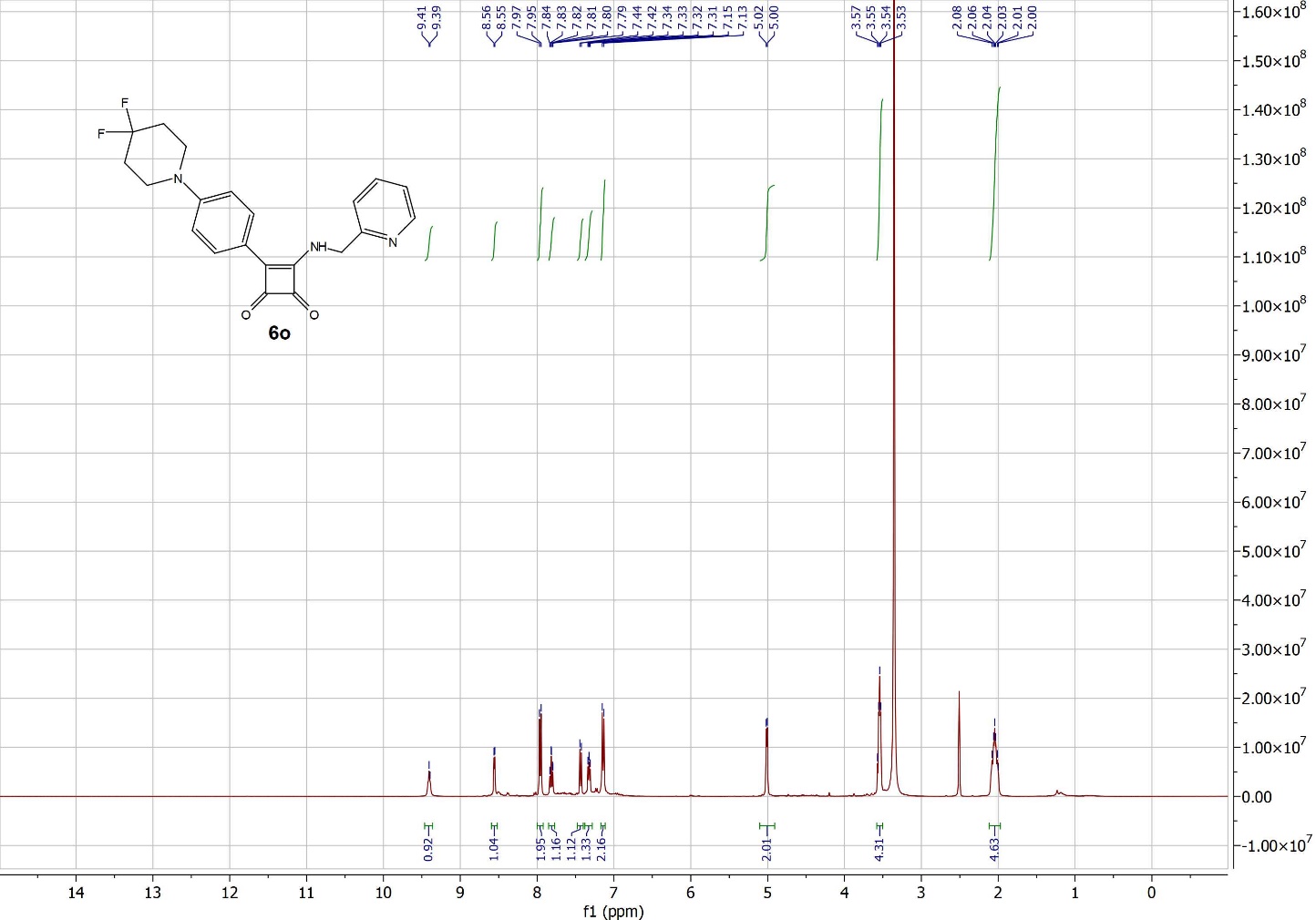

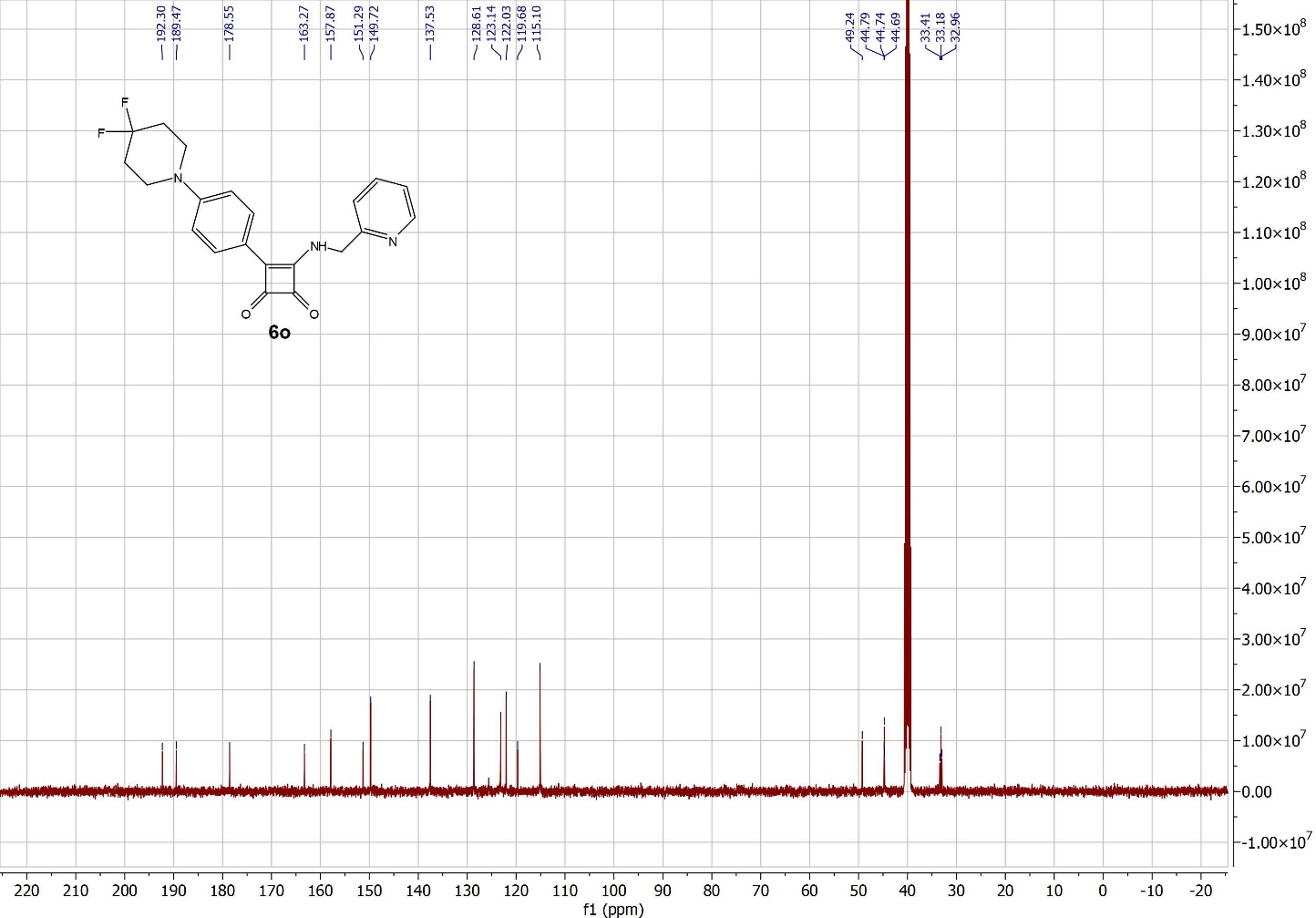


**Figure S31.** ¹H NMR (400 MHz, DMSO) and ^13^C NMR (101 MHz, DMSO) of compound **6p**


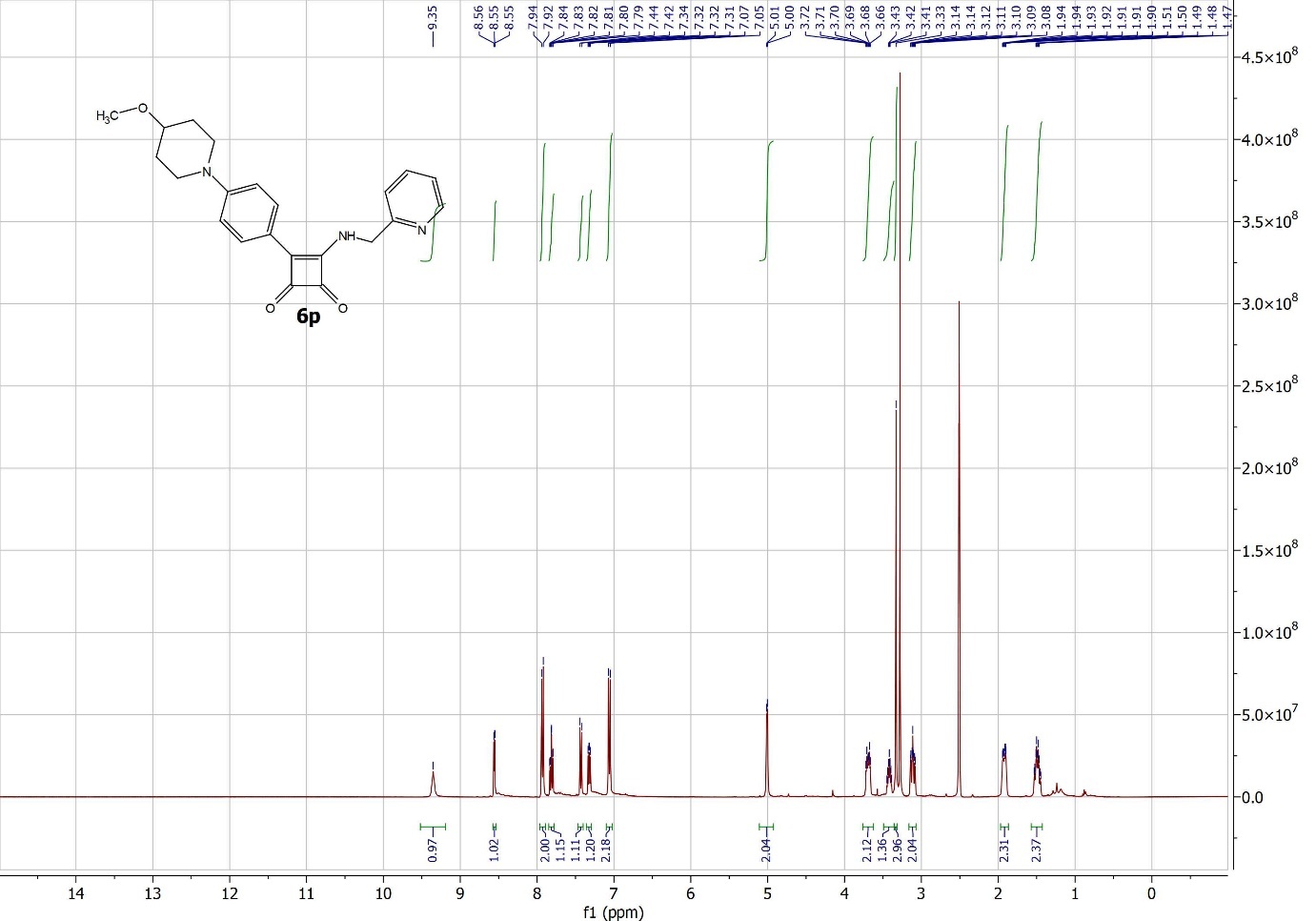


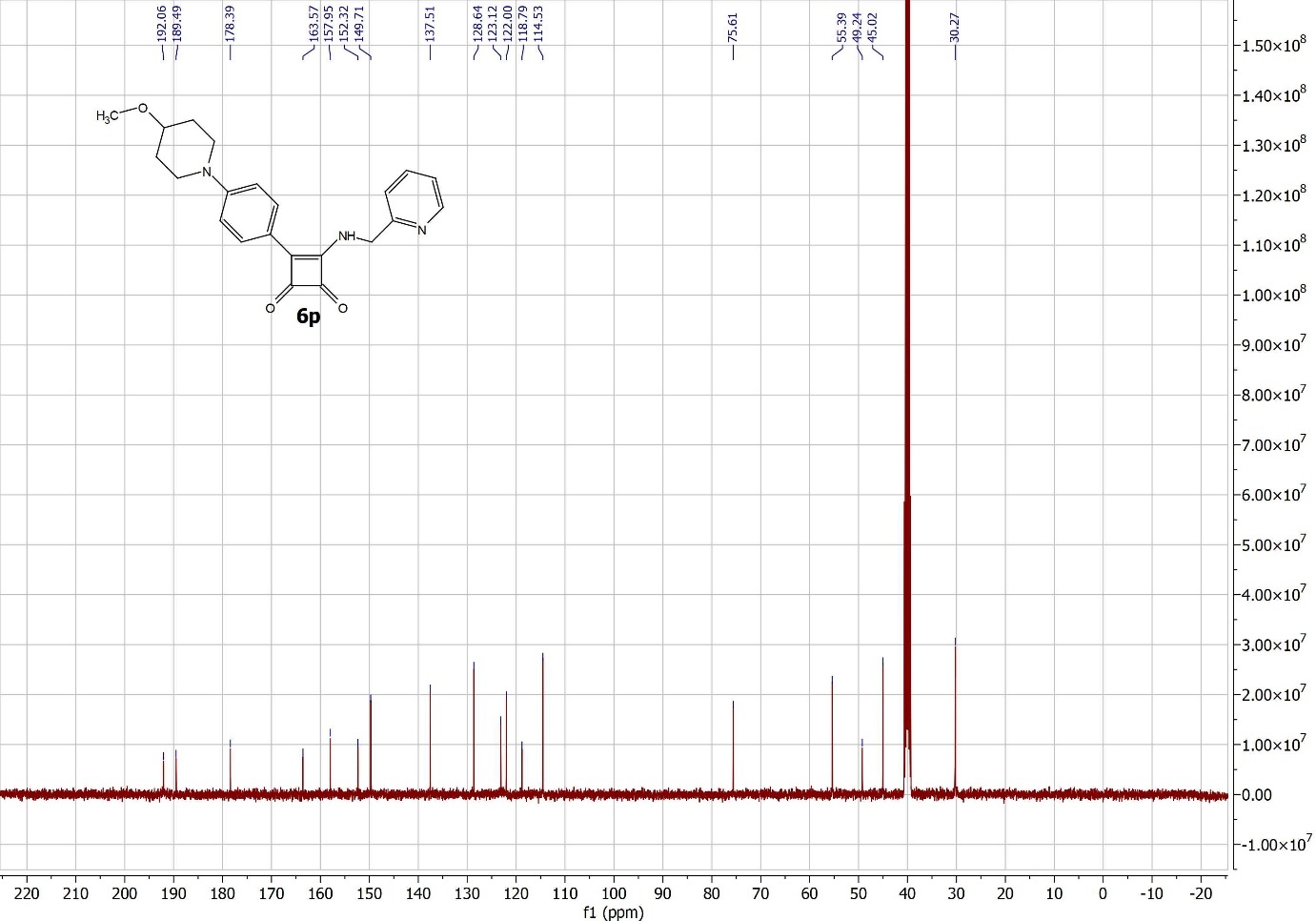


**Figure S32.** ¹H NMR (400 MHz, DMSO), ^13^C NMR (101 MHz, DMSO) and HPLC chromatogram of compound **6q**


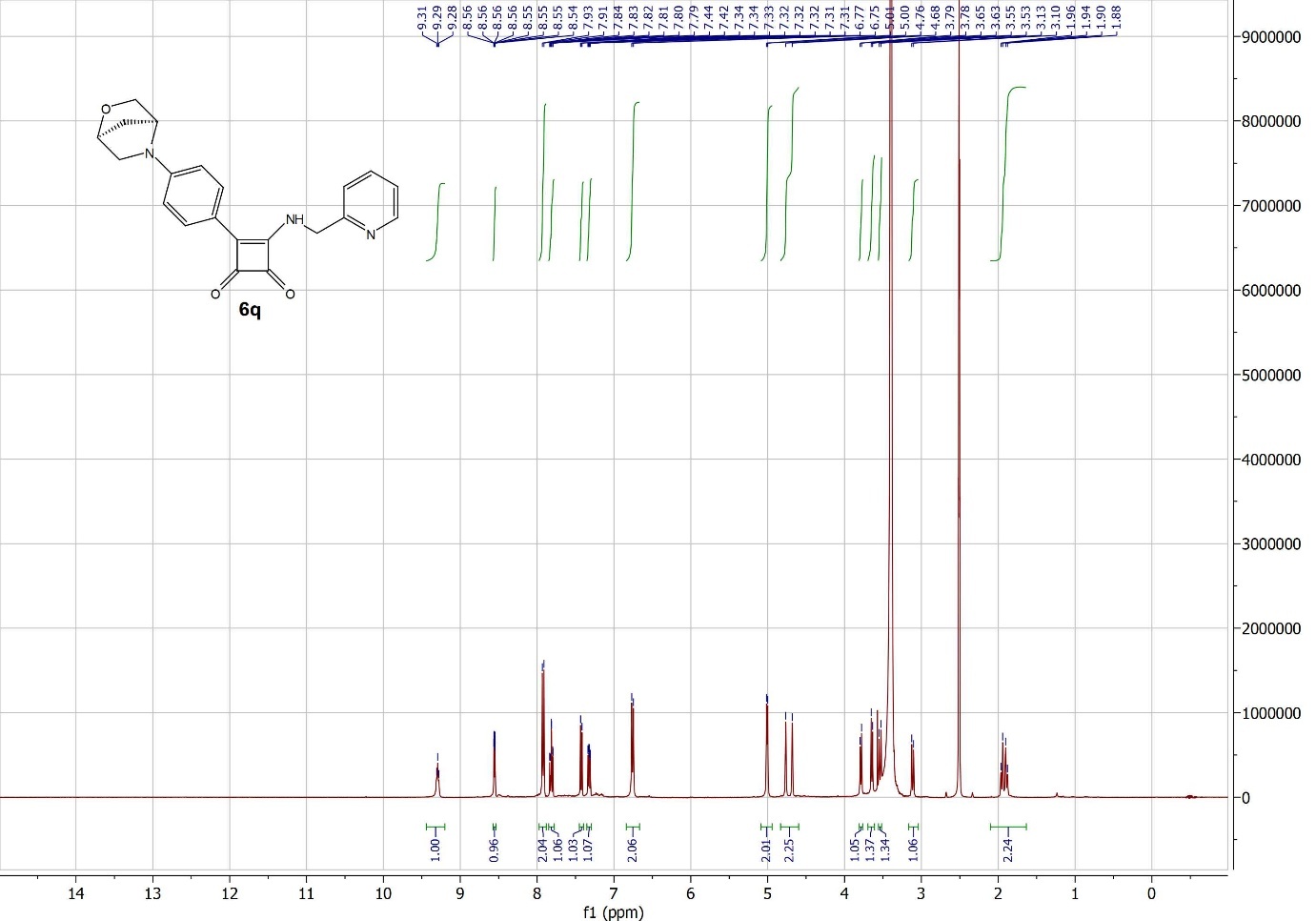


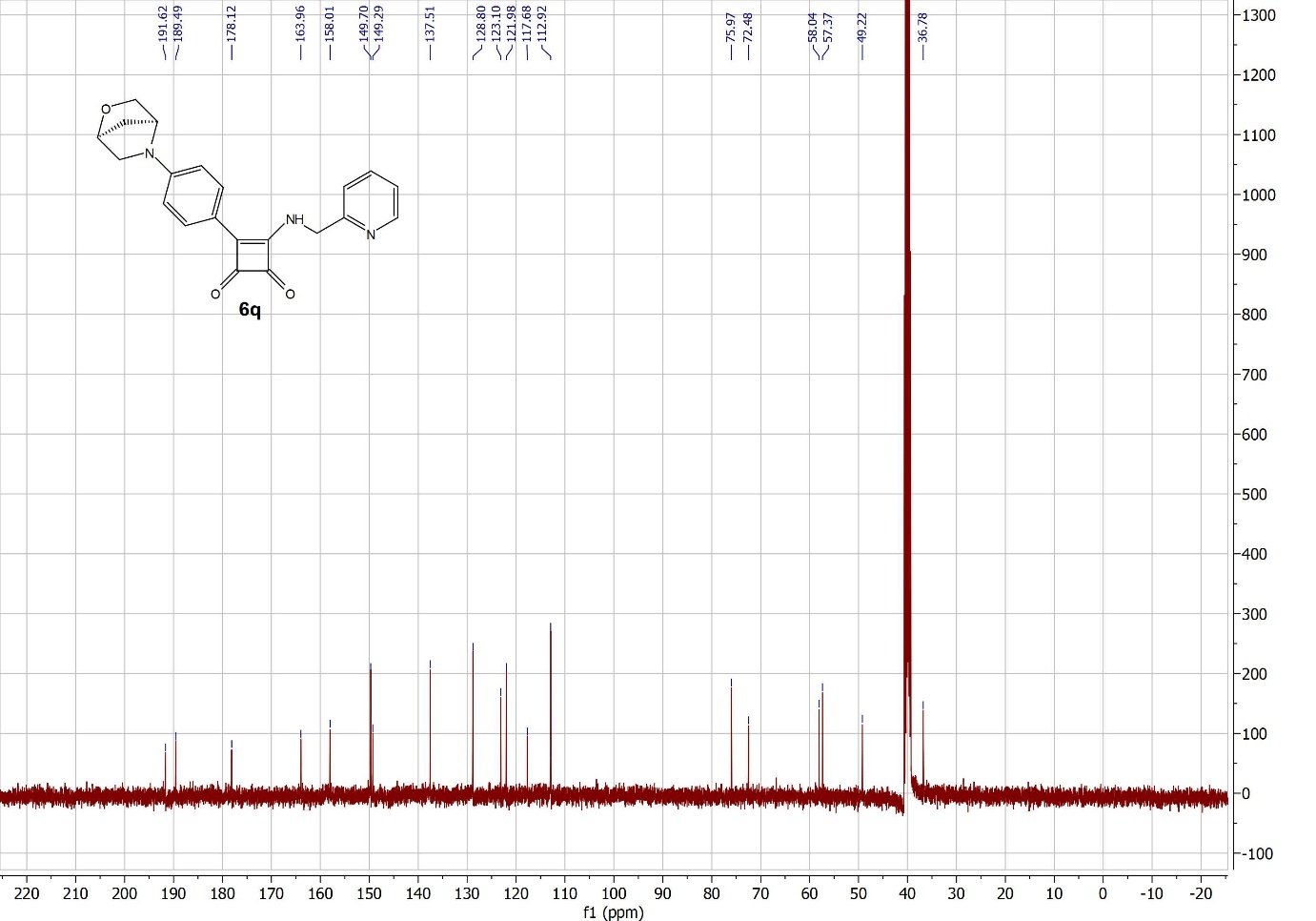


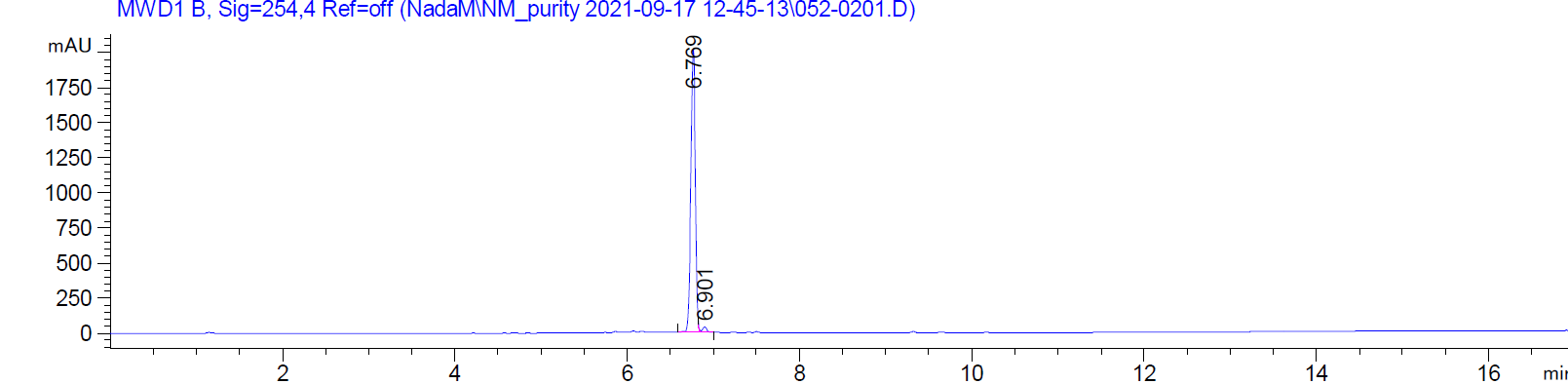


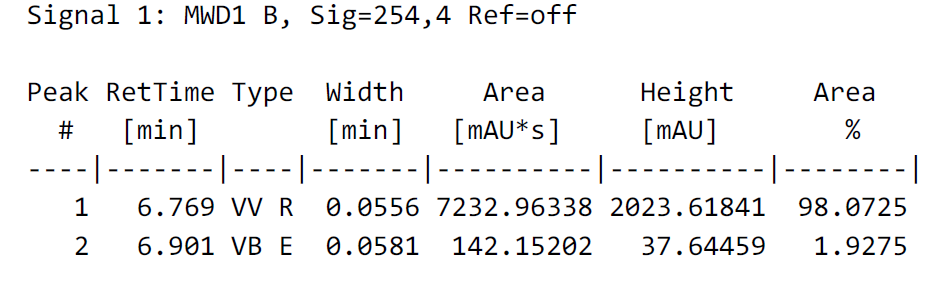


**Figure S33.** ¹H NMR (400 MHz, DMSO), ^13^C NMR (101 MHz, DMSO) and HPLC chromatogram of compound **6r**


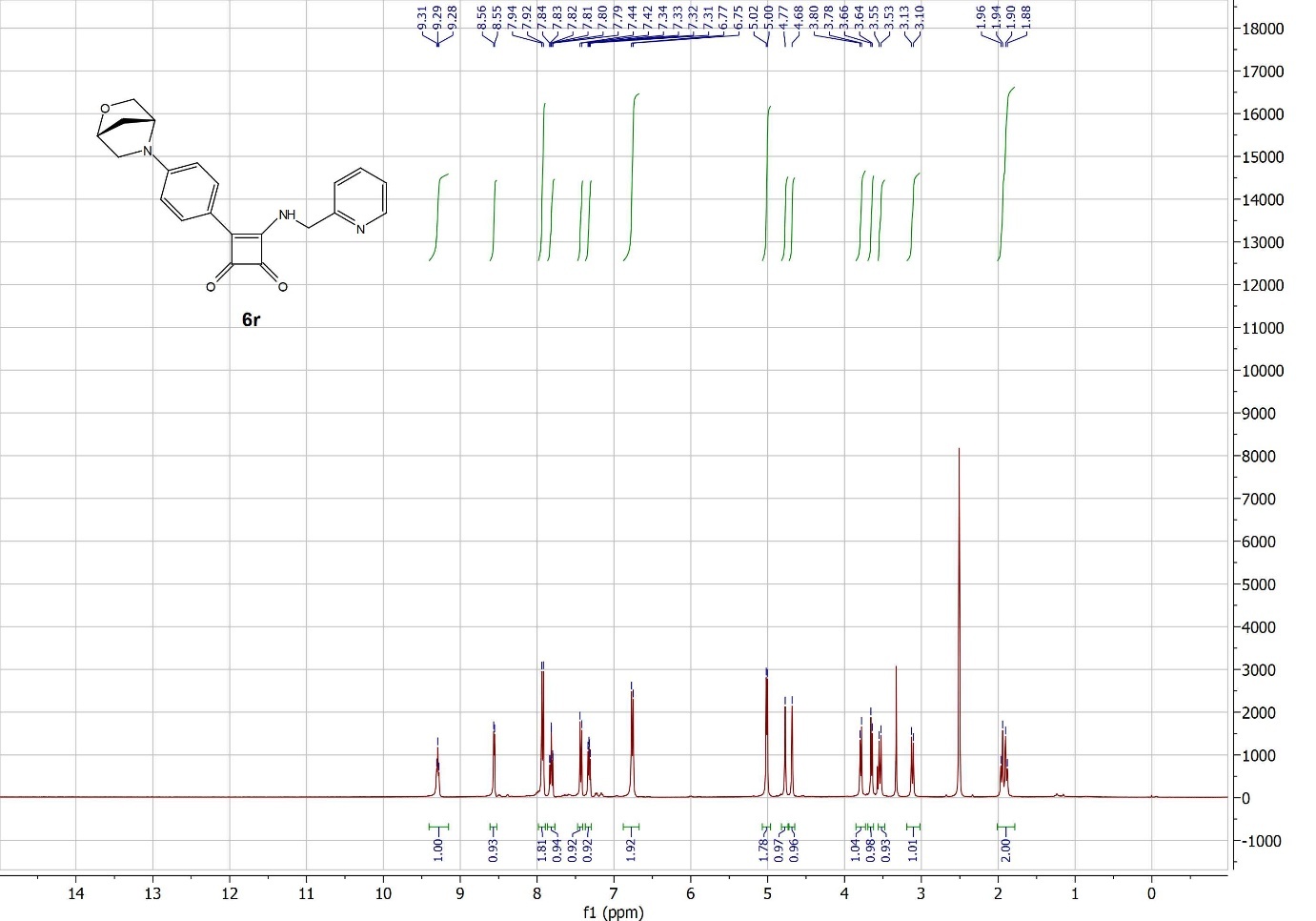


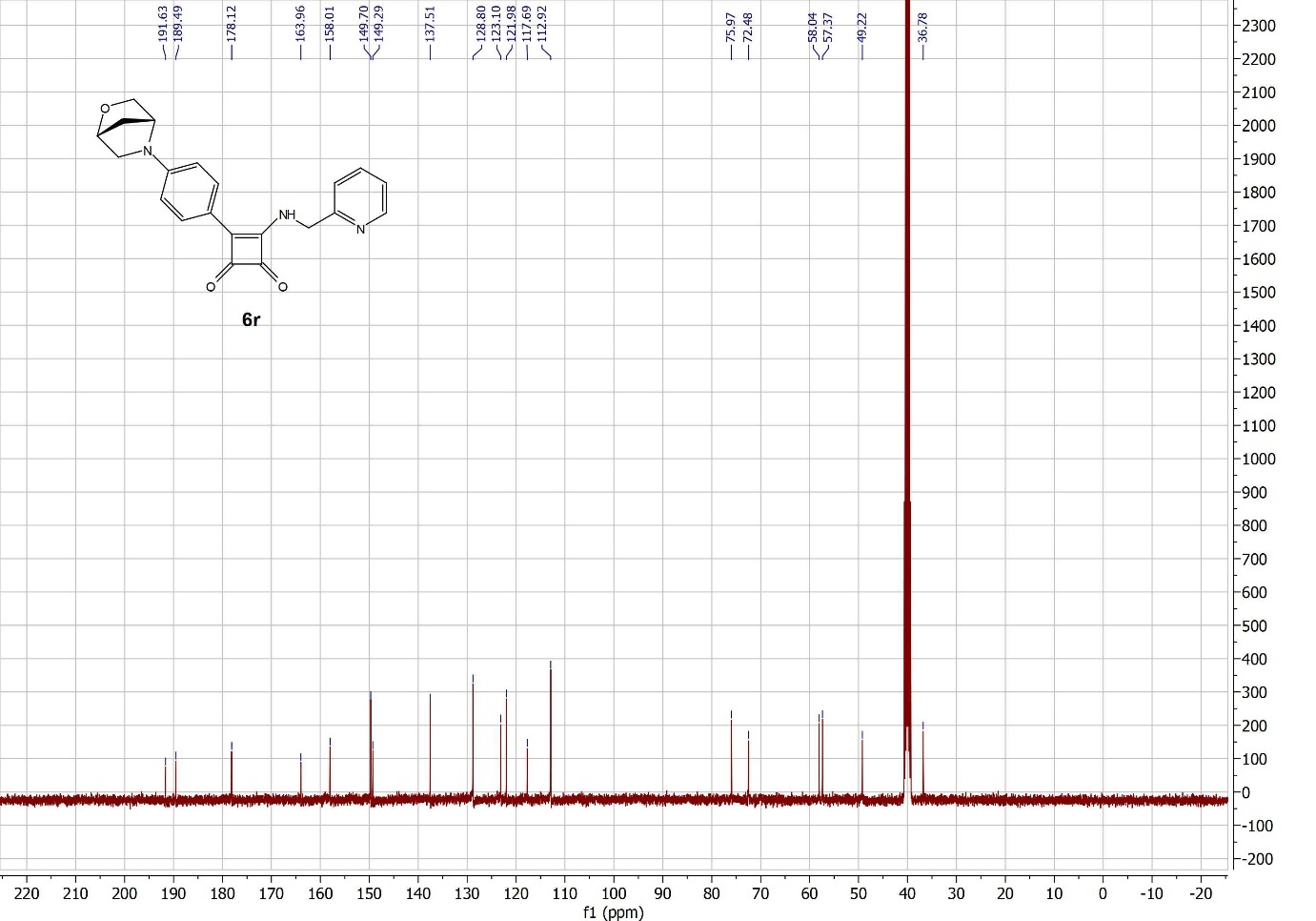


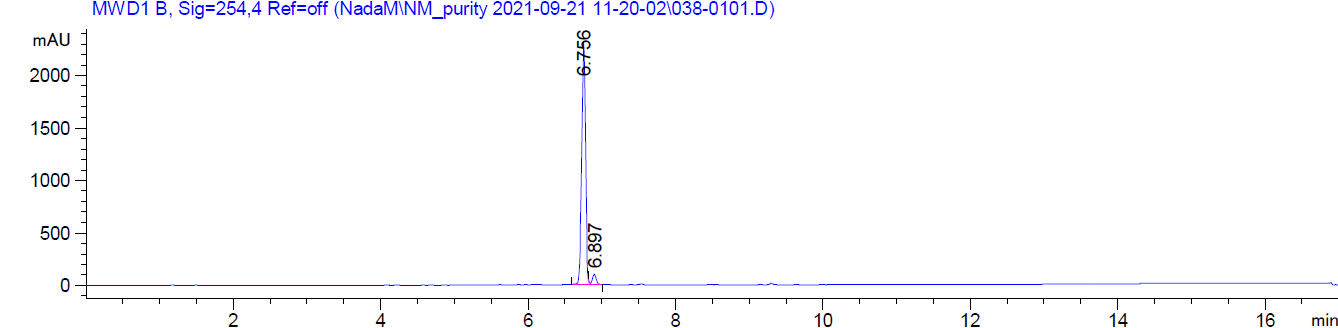


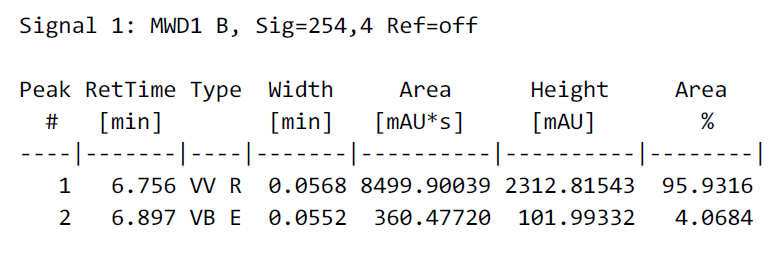


**Figure S34.** ¹H NMR (400 MHz, DMSO) and ^13^C NMR (101 MHz, DMSO) of compound **6s**

**
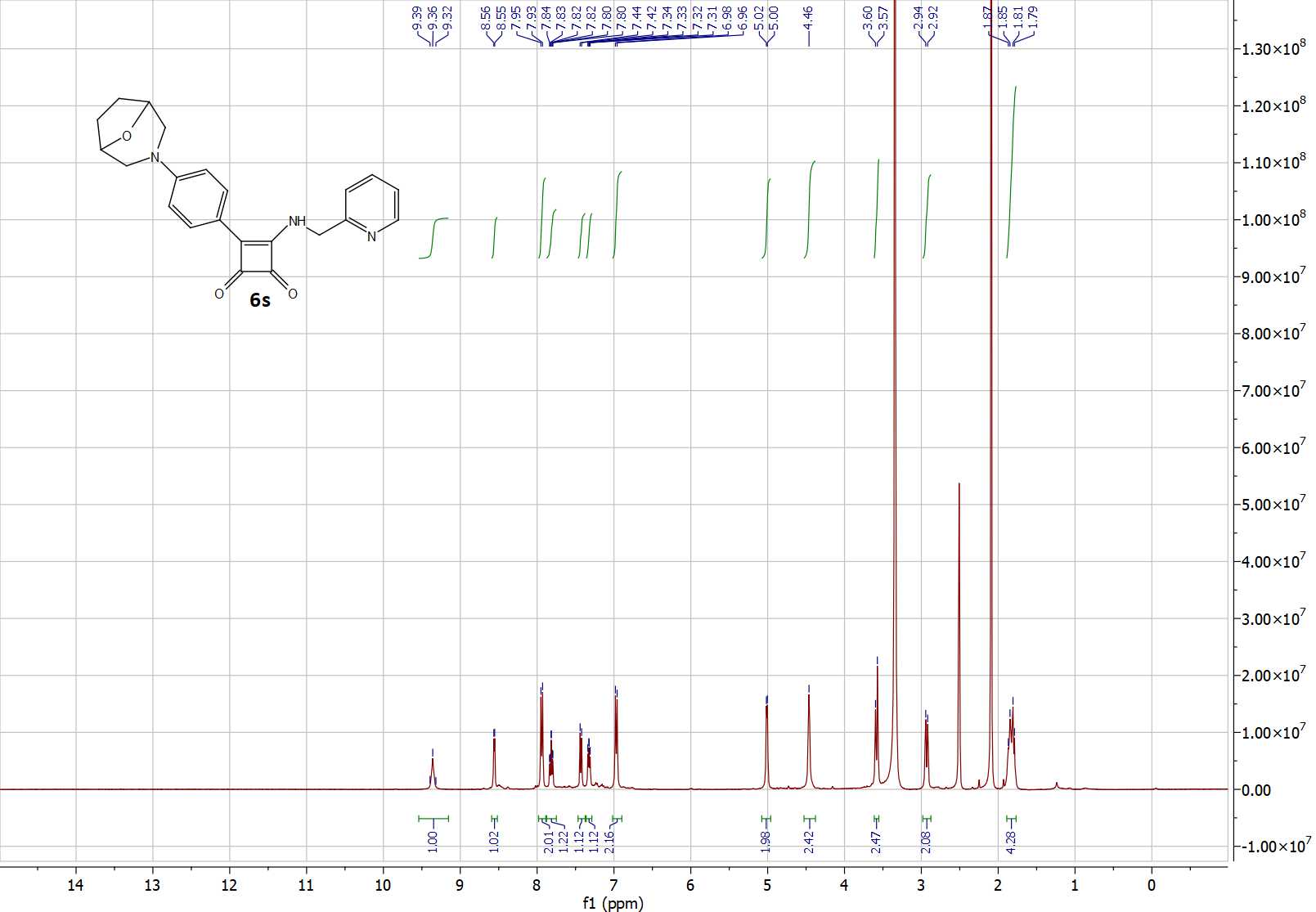
** **
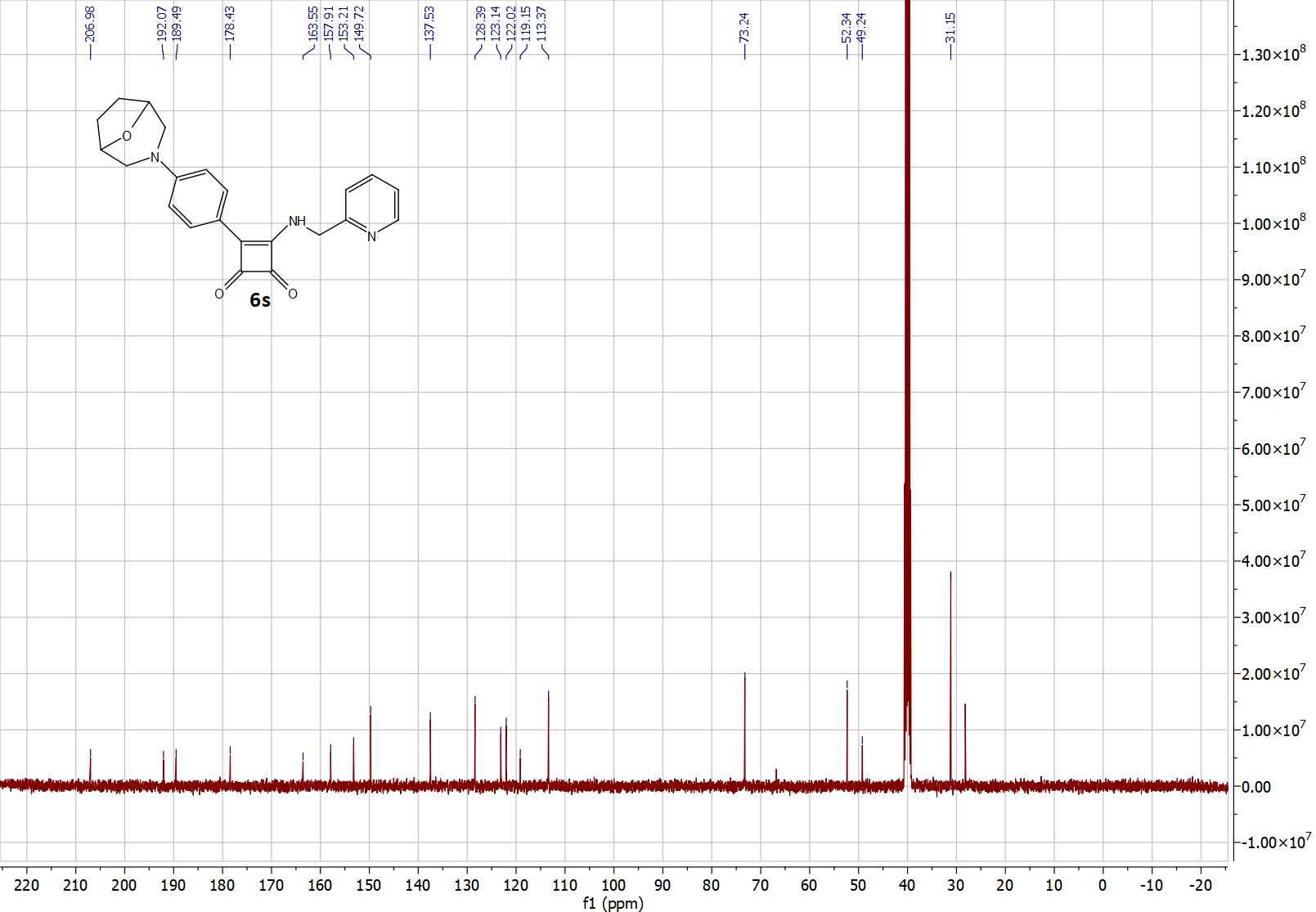
**

**Figure S35.** ¹H NMR (400 MHz, DMSO), ^13^C NMR (101 MHz, DMSO) and HPLC chromatogram of compound **13a**
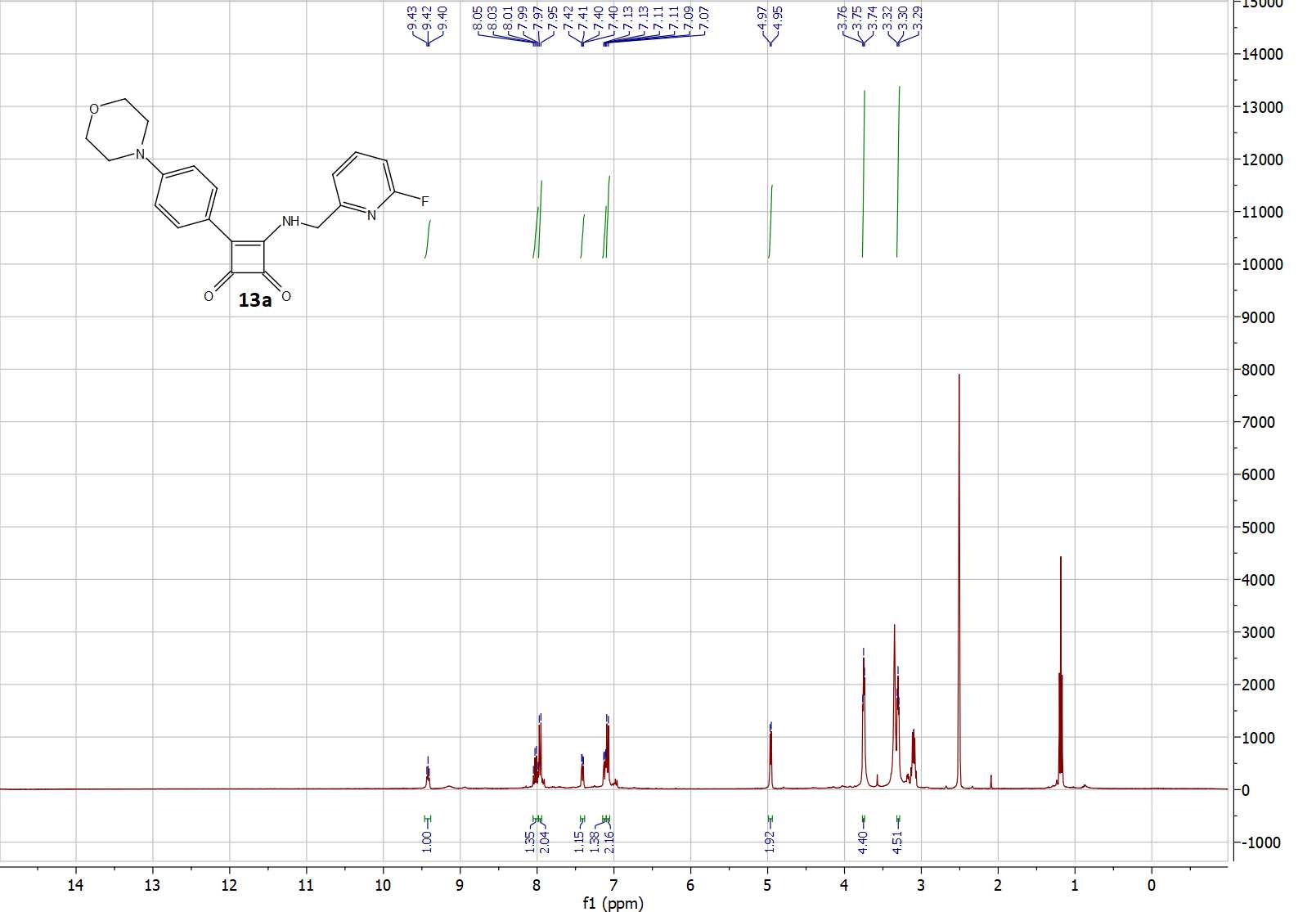


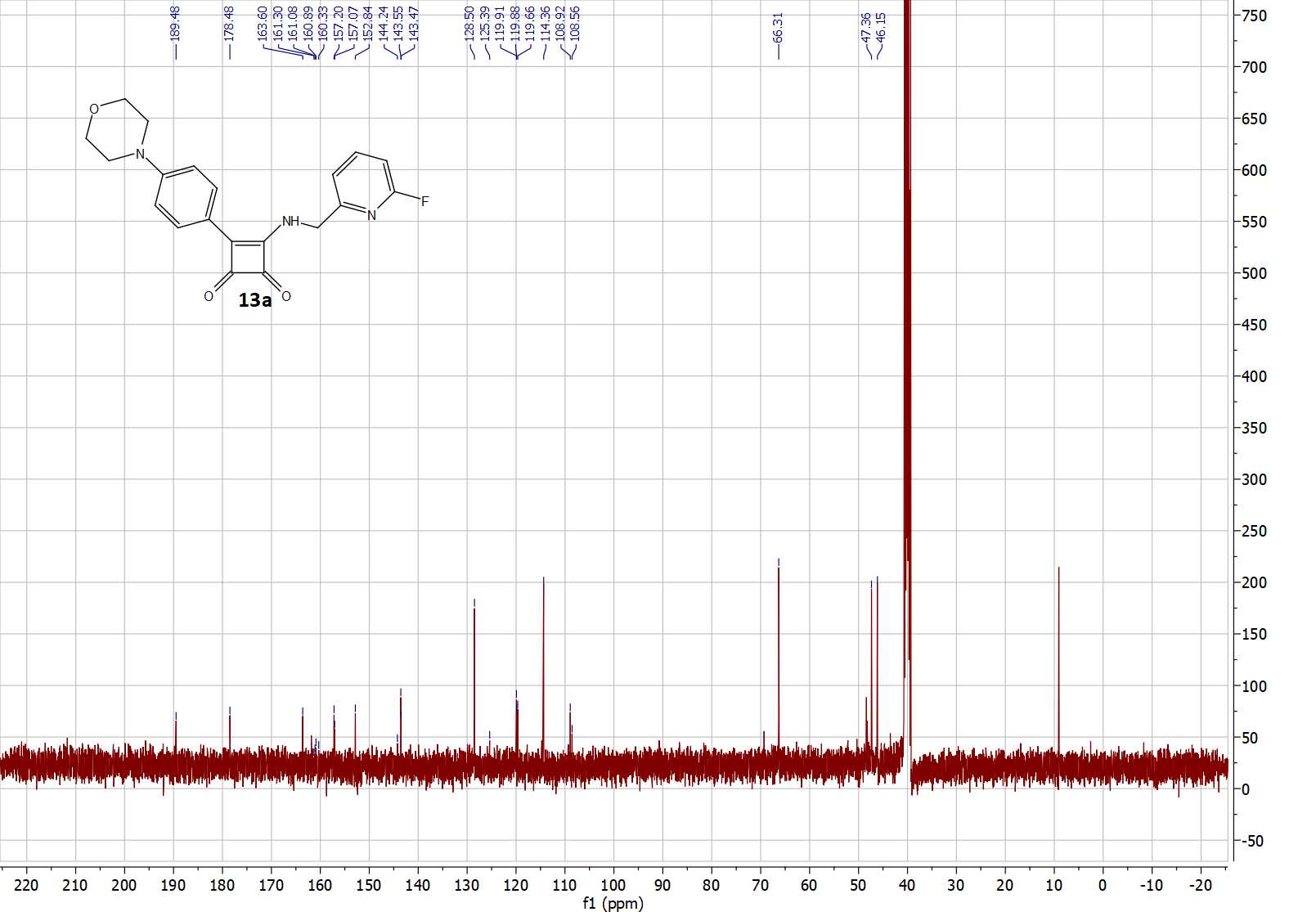


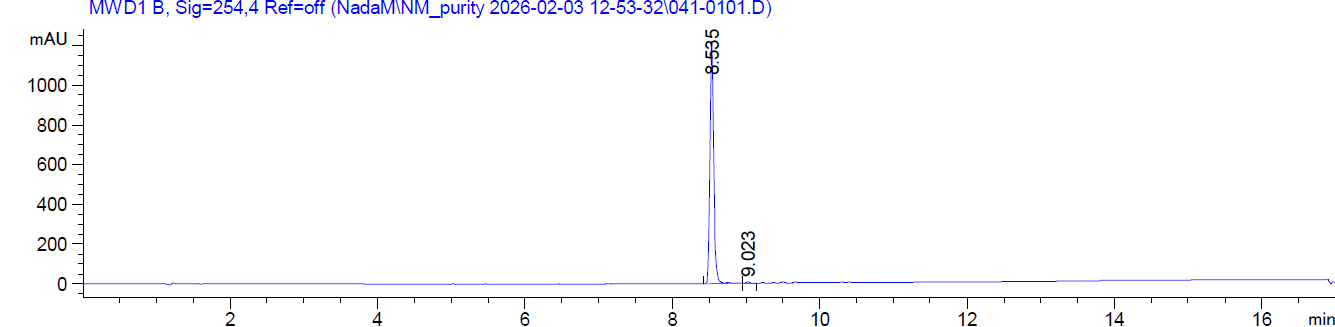


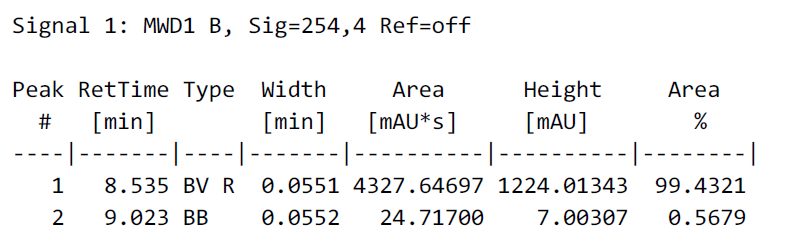


**Figure S36.** ¹H NMR (400 MHz, DMSO), ^13^C NMR (101 MHz, DMSO) and HPLC chromatogram of compound **13b**


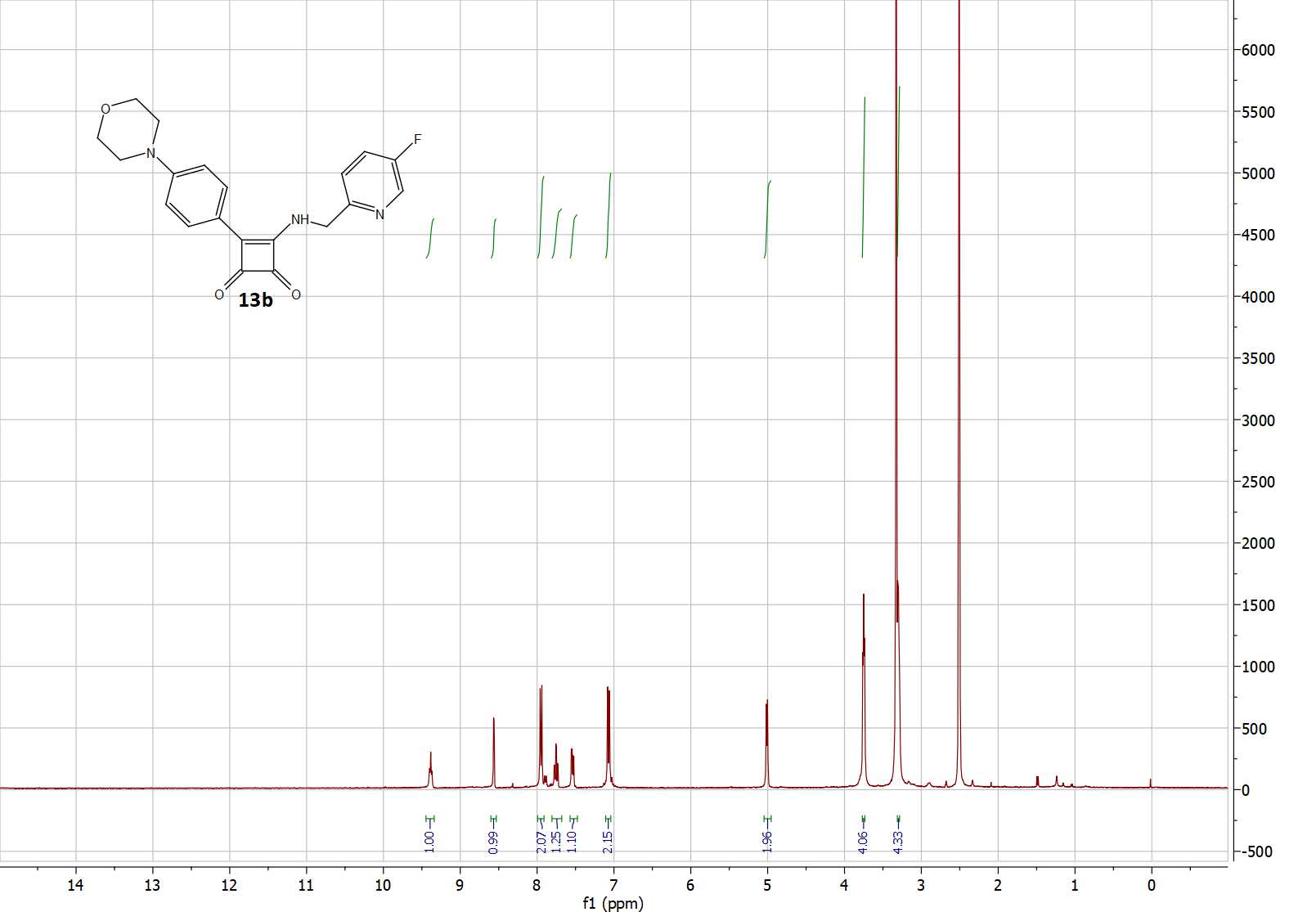


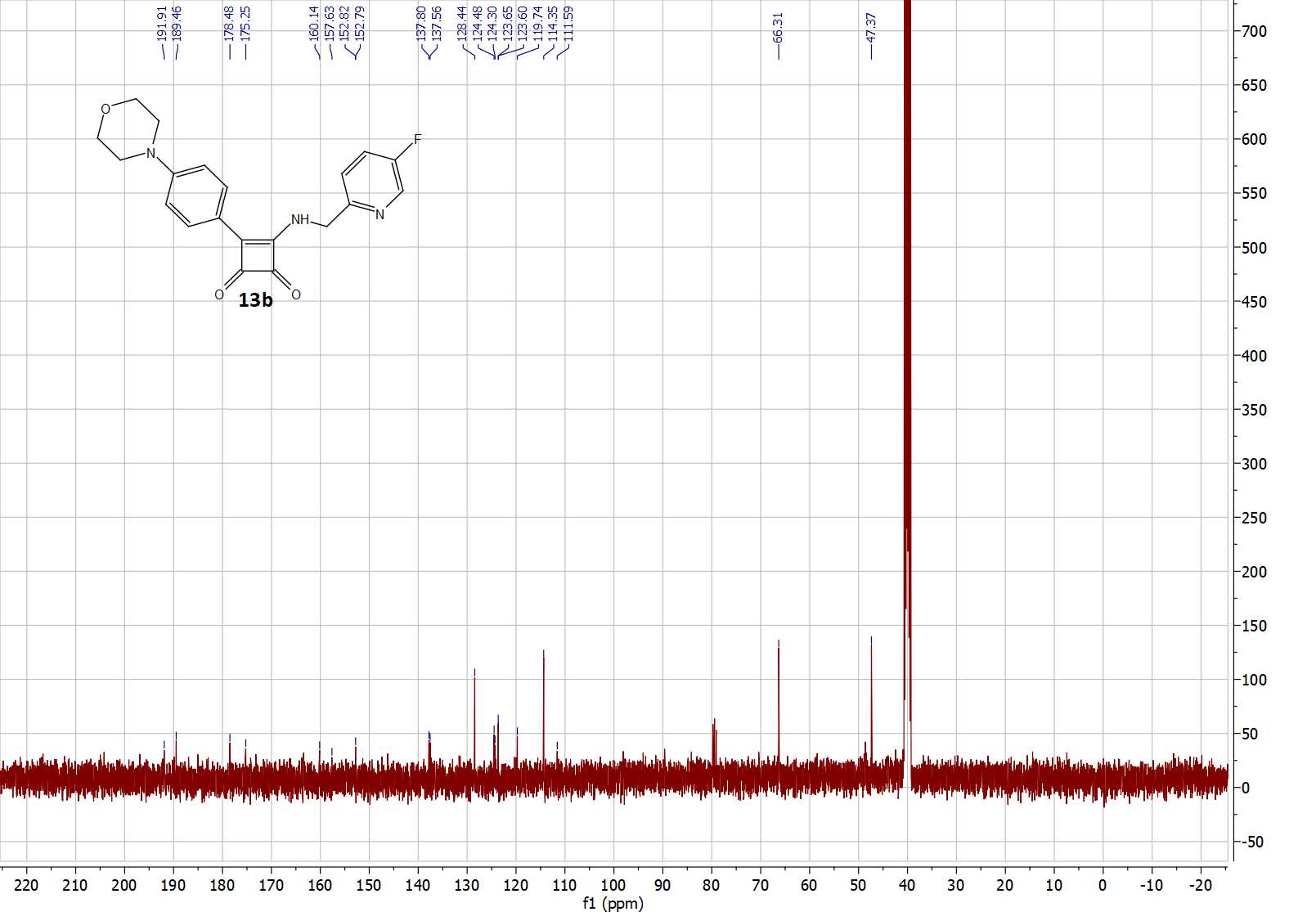


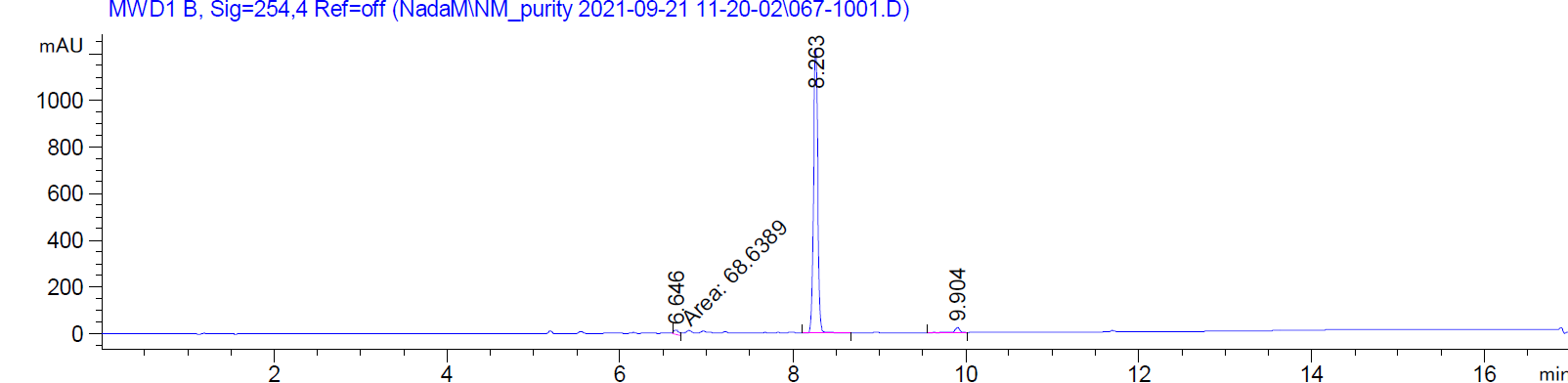


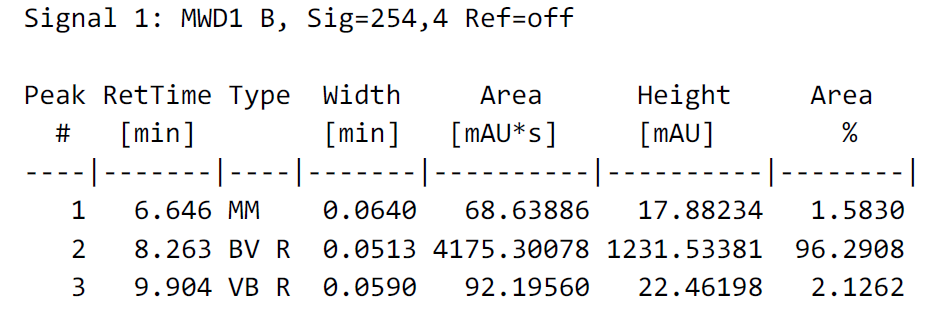


**Figure S37.** ¹H NMR (400 MHz, DMSO) and ^13^C NMR (101 MHz, DMSO) of compound **13c**


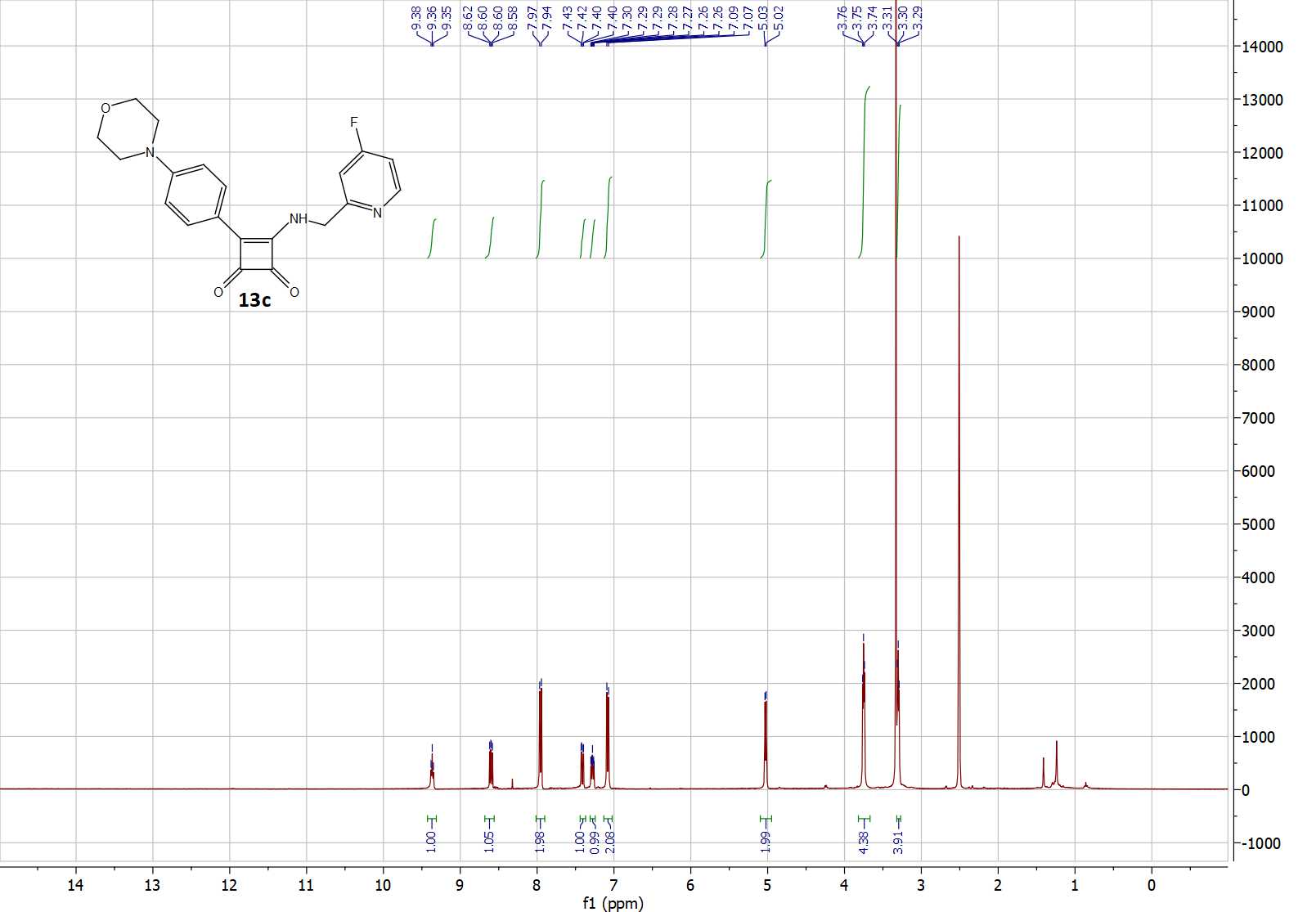


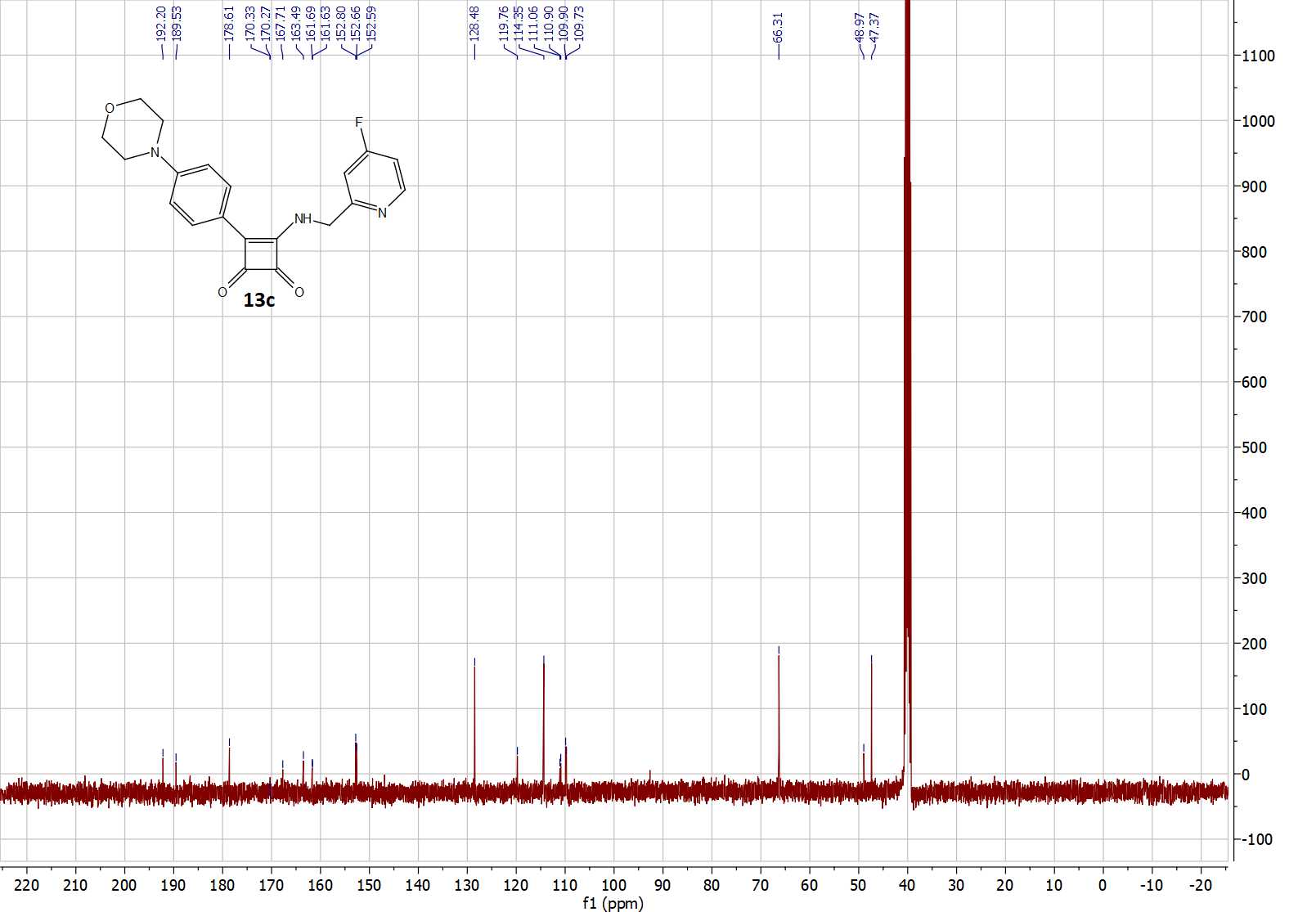
**Figure S38.** ¹H NMR (400 MHz, DMSO) and ESI-HRMS of compound **13d**


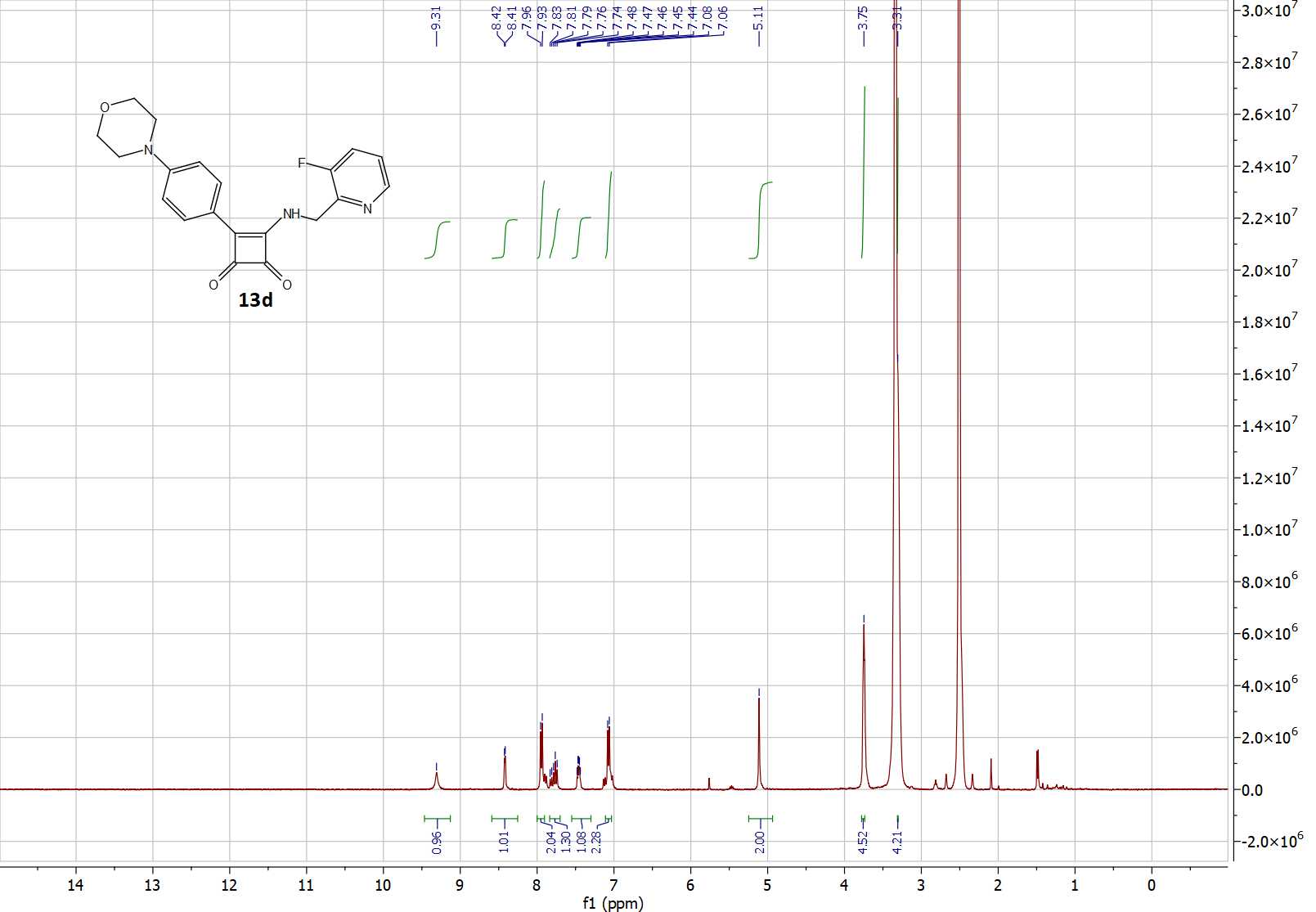


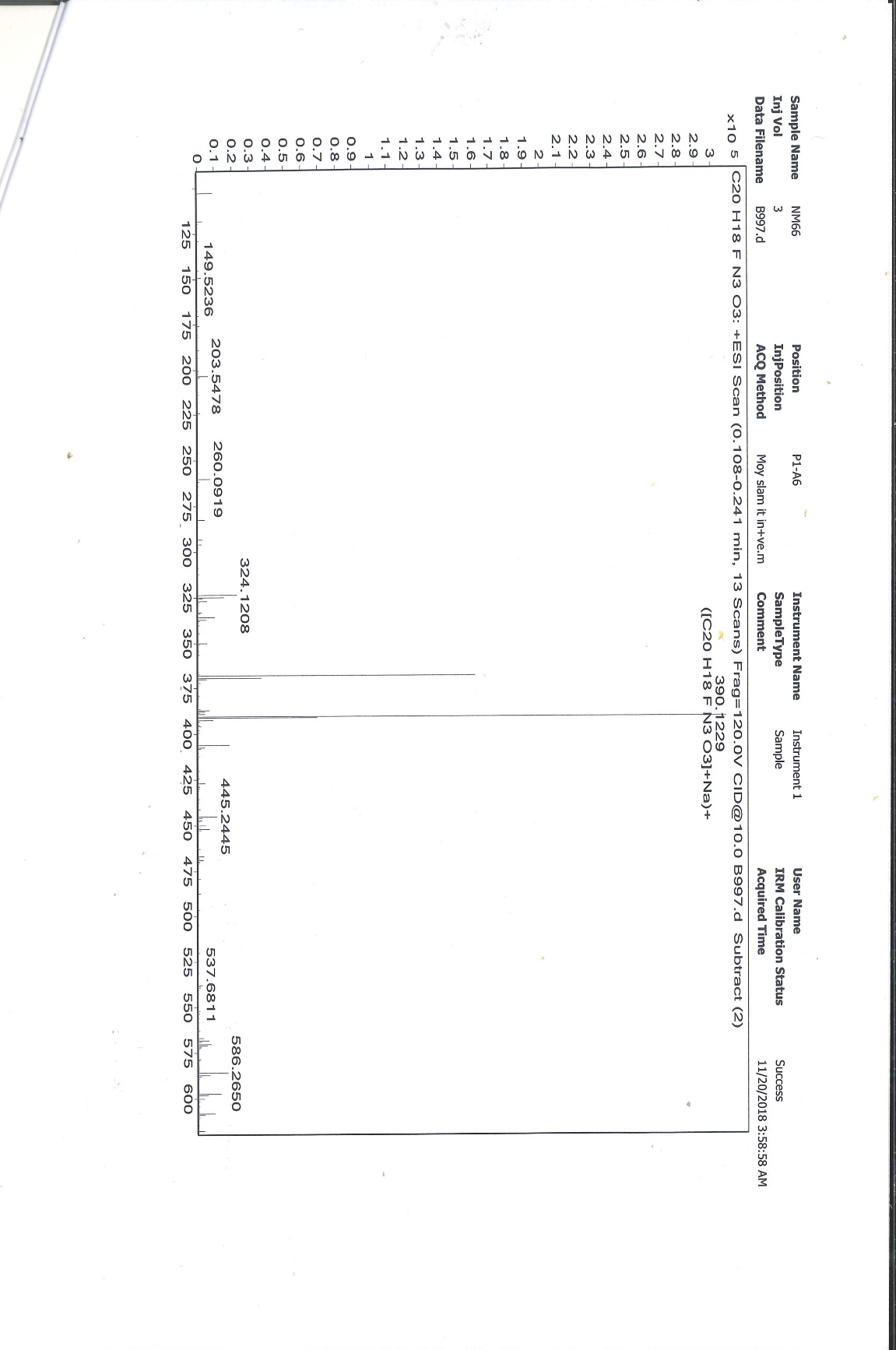


**13d**

**Figure S39.** ¹H NMR (400 MHz, DMSO) and ^13^C NMR (101 MHz, DMSO) of compound **13e**


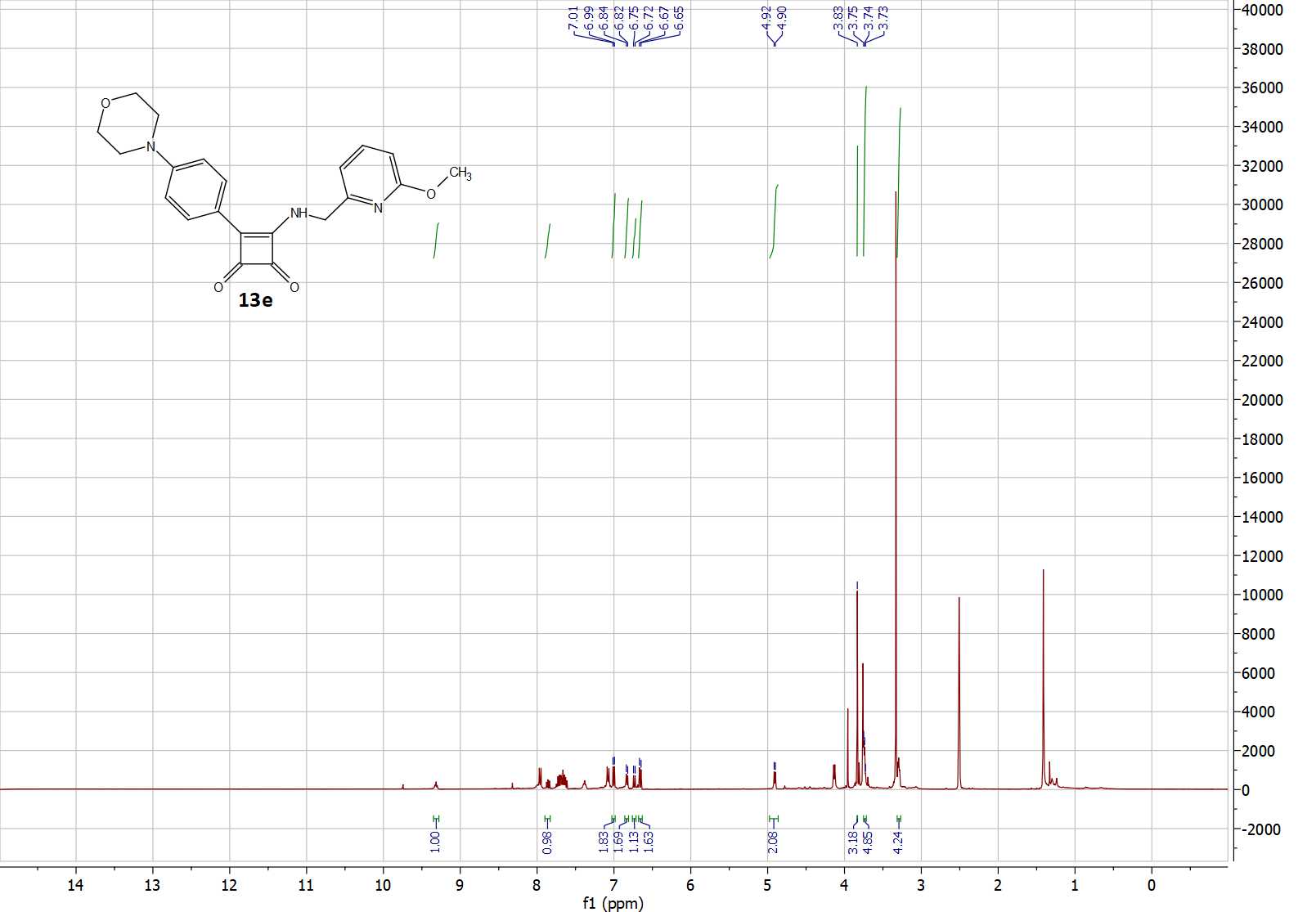

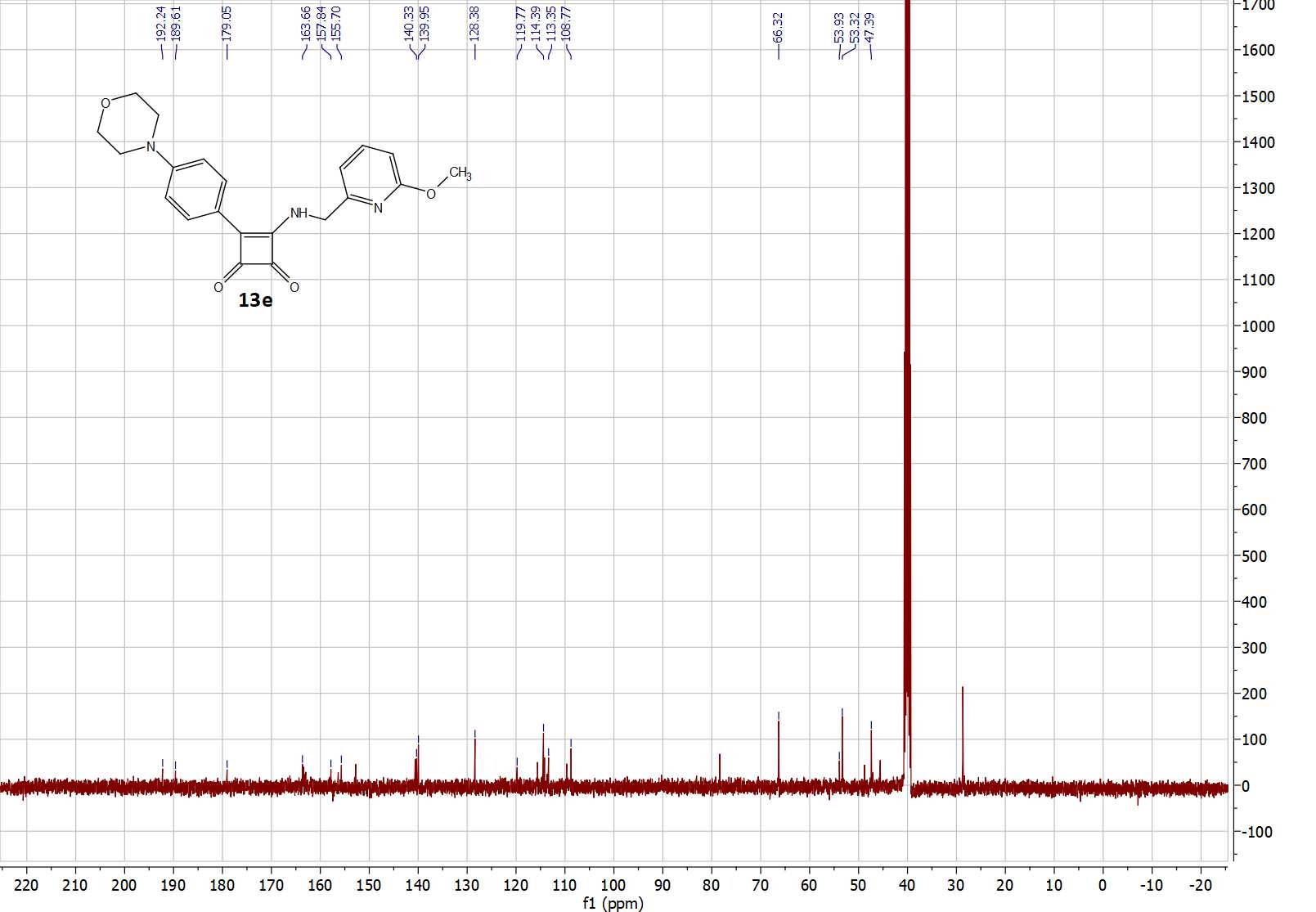


**Figure S40.** ¹H NMR (400 MHz, DMSO) and ^13^C NMR (101 MHz, DMSO) of compound **13f**


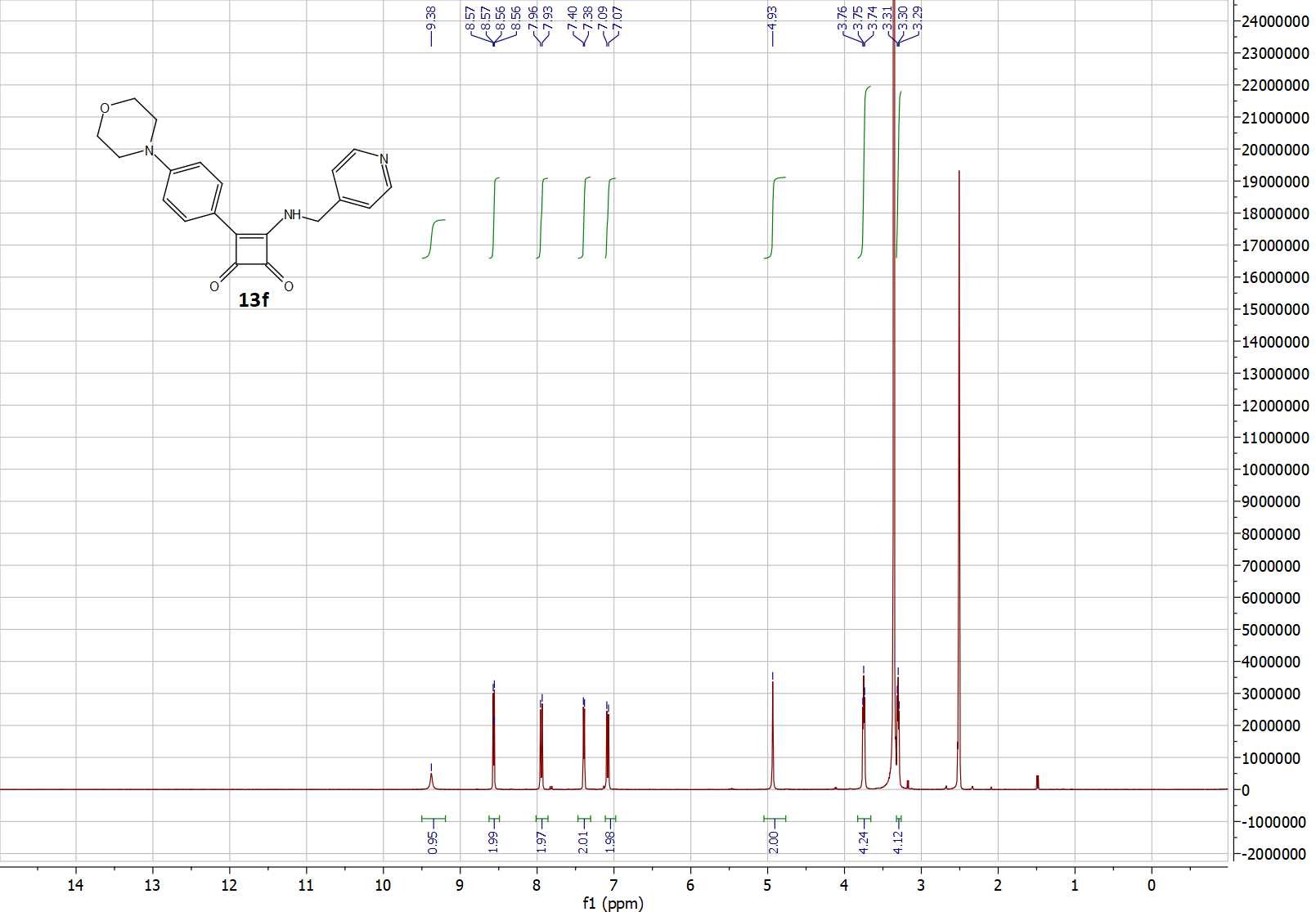

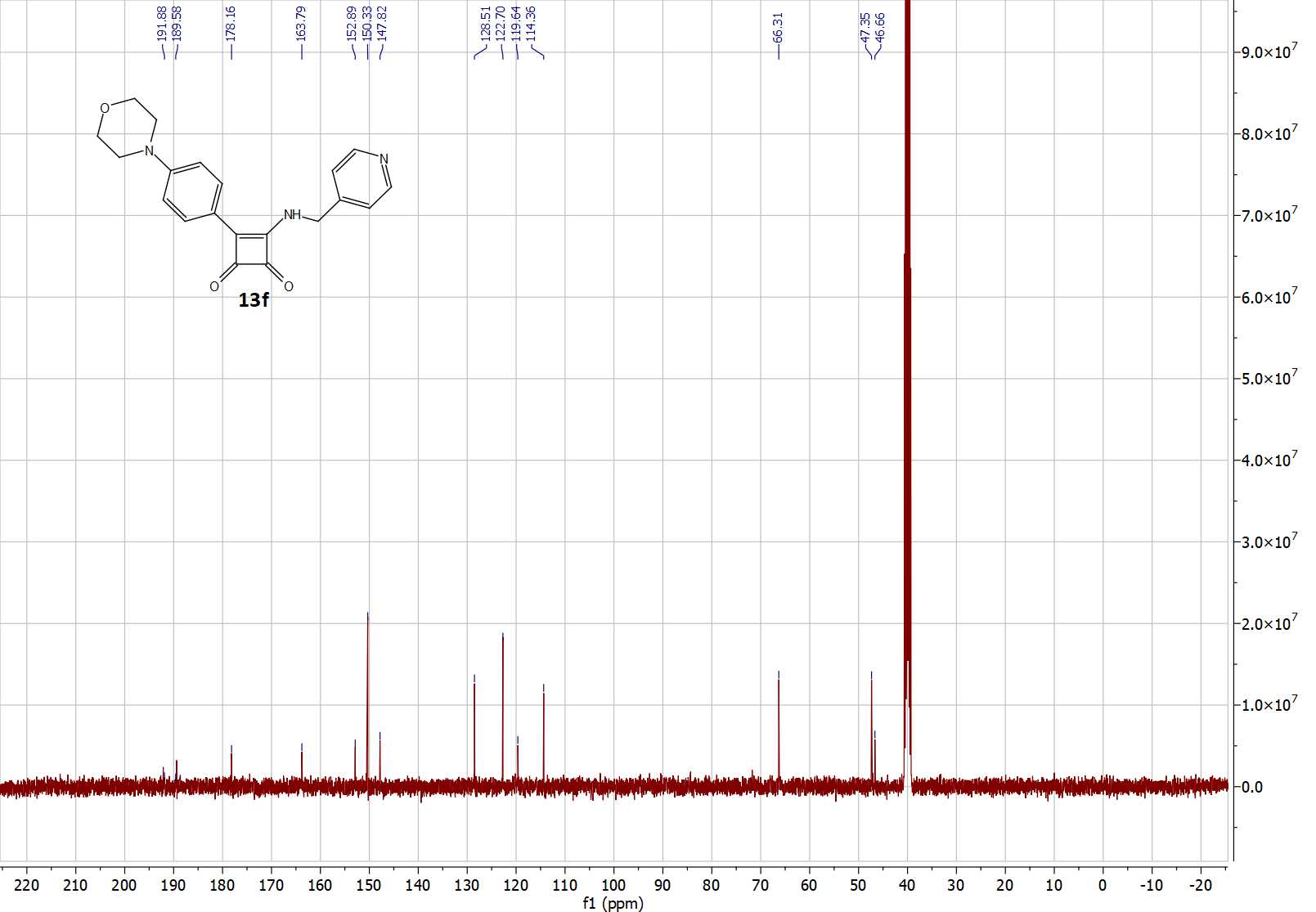
**Figure S41.** ¹H NMR (400 MHz, DMSO) and ^13^C NMR (101 MHz, DMSO) of compound **13g**

**
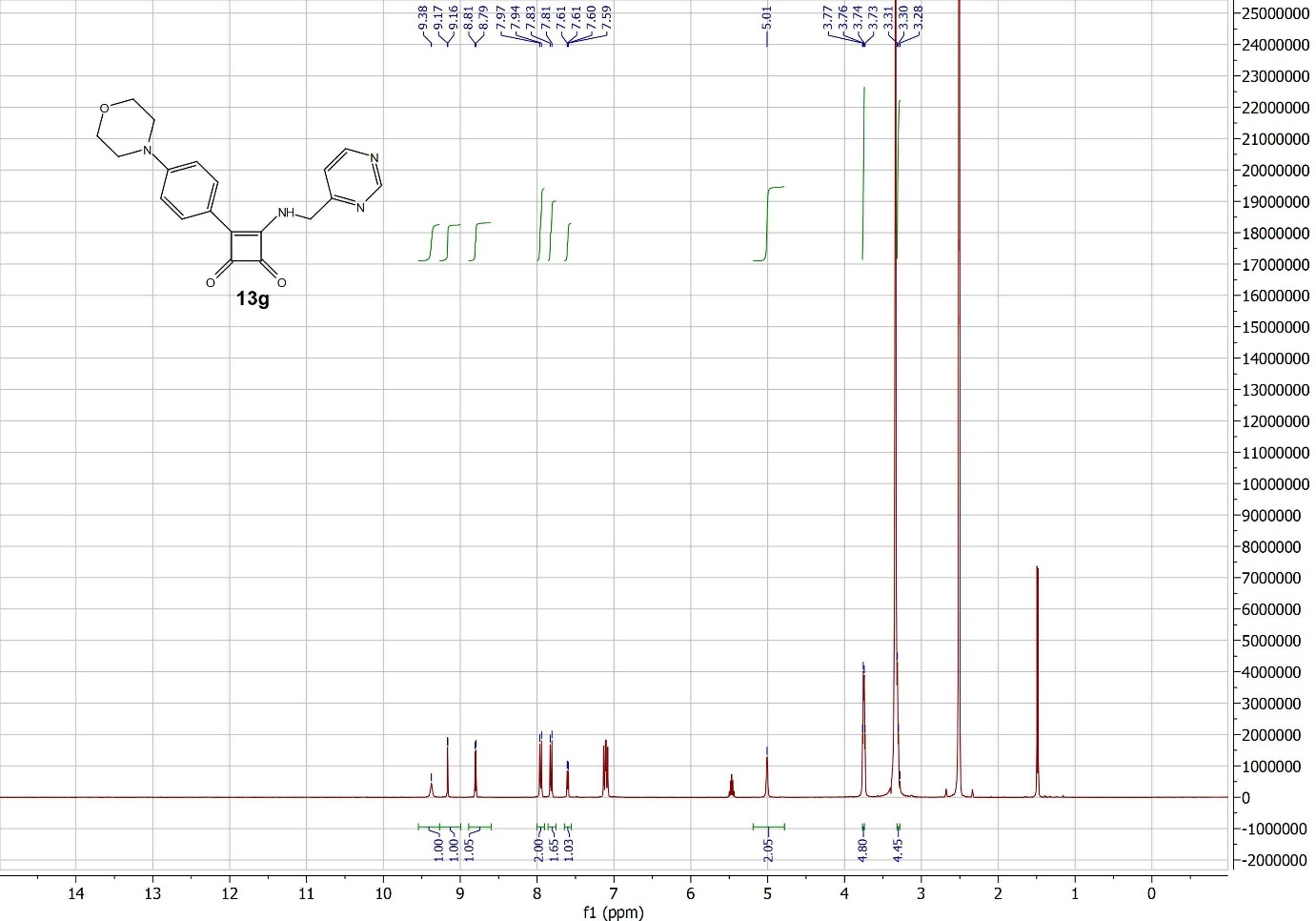
** **
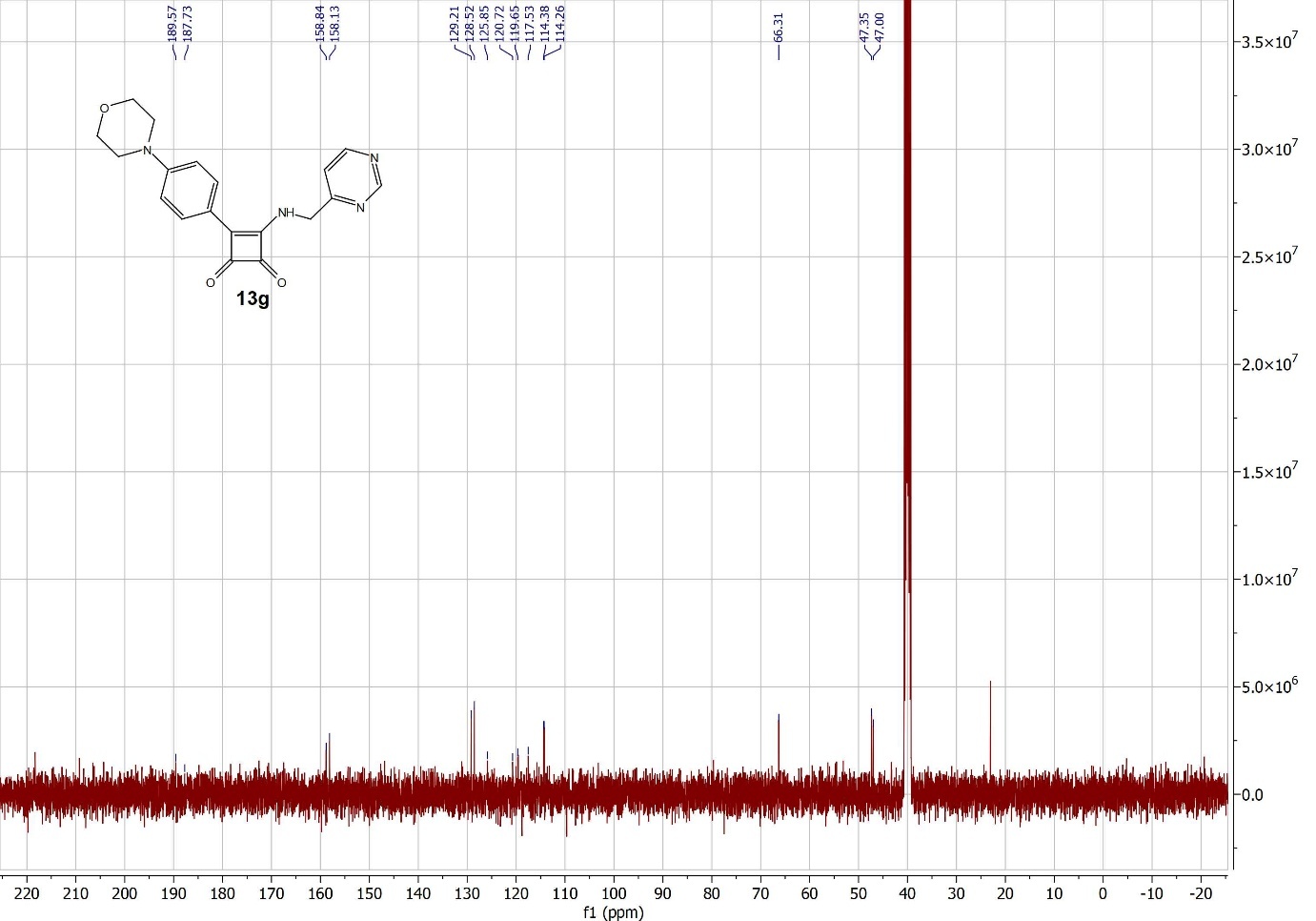
**

**Figure S42.** ¹H NMR (400 MHz, DMSO) and ^13^C NMR (101 MHz, DMSO) of compound **14a**


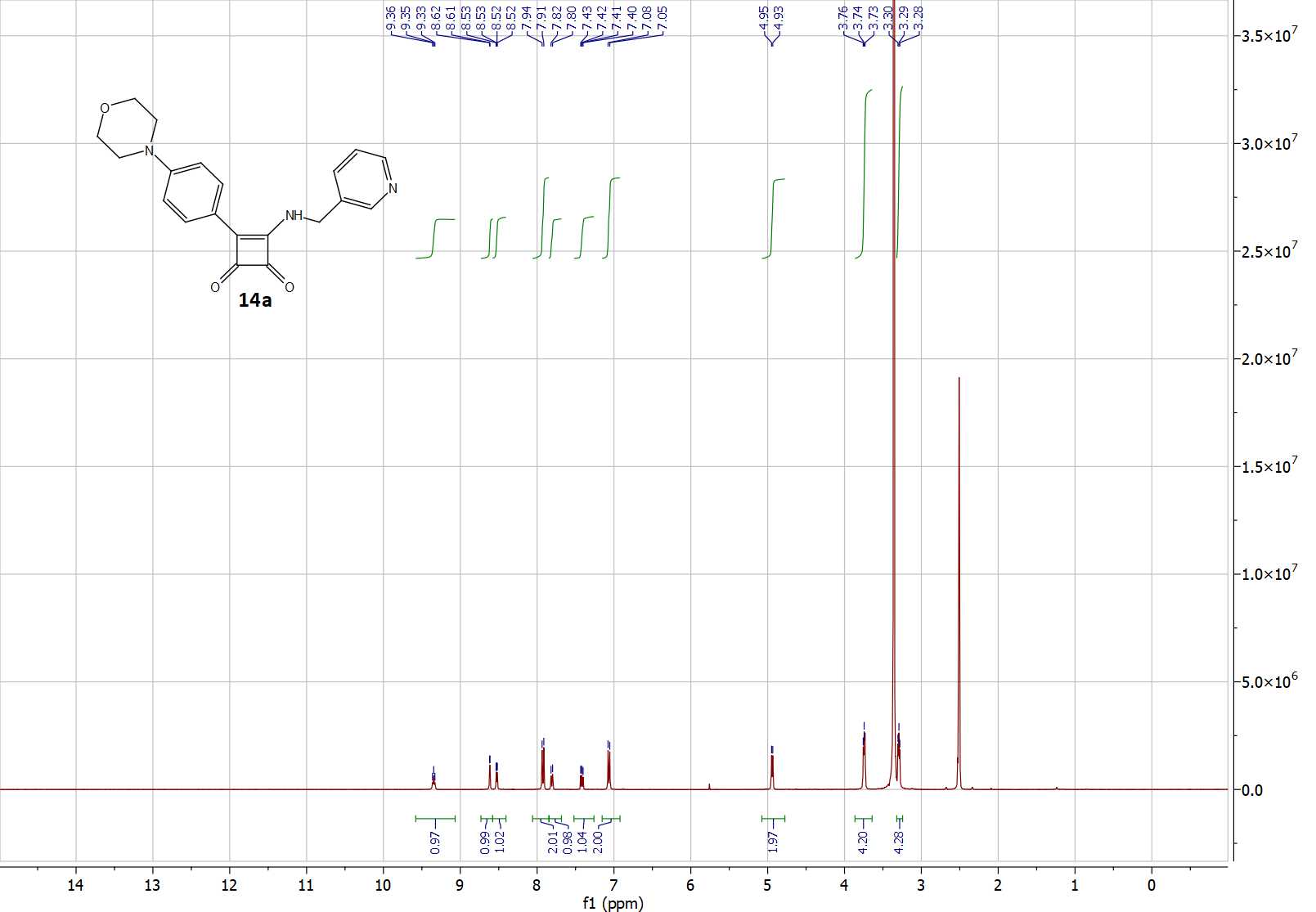

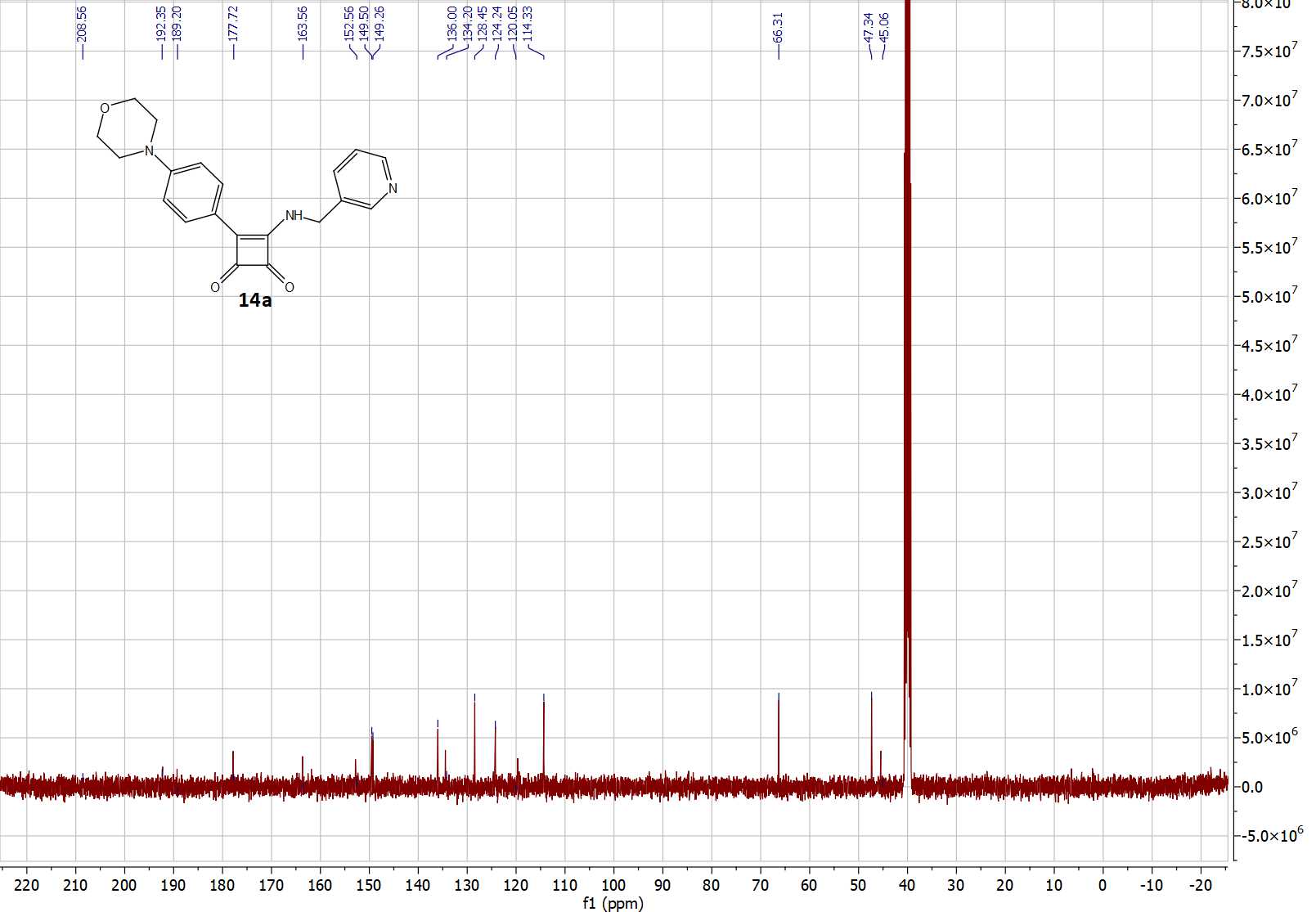


**Figure S43.** ¹H NMR (400 MHz, DMSO) and ^13^C NMR (101 MHz, DMSO) of compound **14b**

**Figure S44.** ¹H NMR (400 MHz, DMSO) and ^13^C NMR (101 MHz, DMSO) of compound **14c**

**Figure S45.** ¹H NMR (400 MHz, DMSO) and ^13^C NMR (101 MHz, DMSO) of compound **14d**

**Figure S46.** ¹H NMR (400 MHz, DMSO) and ^13^C NMR (101 MHz, DMSO) of compound **14e**

**Figure S47.** ¹H NMR (400 MHz, DMSO) and ^13^C NMR (101 MHz, DMSO) of compound **14f**

**Figure S48.** ¹H NMR (400 MHz, DMSO) and ^13^C NMR (101 MHz, DMSO) of compound **14g**

**Figure S49.** ¹H NMR (400 MHz, DMSO) and ^13^C NMR (101 MHz, DMSO) of compound **14h**

**Figure S50.** ¹H NMR (400 MHz, DMSO) and ^13^C NMR (101 MHz, DMSO) of compound **14i**

**Figure S51.** ¹H NMR (400 MHz, DMSO) and ^13^C NMR (101 MHz, DMSO) of compound **14j**

**Figure S52.** ¹H NMR (400 MHz, DMSO) and ESI-HRMS of compound **14k**

**14k**

**Figure S53.** ¹H NMR (400 MHz, DMSO), ^13^C NMR (101 MHz, DMSO) and HPLC chromatogram of compound **20a**

**Figure S54.** ¹H NMR (400 MHz, DMSO), ^13^C NMR (101 MHz, DMSO) and HPLC chromatogram of compound **20b**

**Figure S55.** ¹H NMR (400 MHz, DMSO) and ^13^C NMR (101 MHz, DMSO) of compound **20c**

**Figure S56.** ¹H NMR (400 MHz, DMSO) and ^13^C NMR (101 MHz, DMSO) of compound **20d**  **Figure S57.** ¹H NMR (400 MHz, DMSO) and ^13^C NMR (101 MHz, DMSO) of compound **20e**

**Figure S58.** ¹H NMR (400 MHz, DMSO), ^13^C NMR (101 MHz, DMSO) and HPLC chromatogram of compound **20f**

**Figure S59.** ¹H NMR (400 MHz, DMSO), ^13^C NMR (101 MHz, DMSO) and HPLC chromatogram of compound **20g**

**Figure S60.** ¹H NMR (400 MHz, DMSO) and ^13^C NMR (101 MHz, DMSO) of compound **20h**

**Figure S61.** ¹H NMR (400 MHz, DMSO) and ^13^C NMR (101 MHz, DMSO) of compound **20i**

**Figure S62.** ¹H NMR (400 MHz, DMSO), ^13^C NMR (101 MHz, DMSO) and HPLC chromatogram of compound **20j**

**Figure S63.** ¹H NMR (400 MHz, DMSO), ^13^C NMR (101 MHz, DMSO) and HPLC chromatogram of compound **20k**

**Figure S64.** ¹H NMR (400 MHz, DMSO), ^13^C NMR (101 MHz, DMSO) and HPLC chromatogram of compound **20l**
